# Mapping physical access to healthcare for older adults in sub-Saharan Africa: A cross-sectional analysis with implications for the COVID-19 response

**DOI:** 10.1101/2020.07.17.20152389

**Published:** 2020-07-21

**Authors:** Pascal Geldsetzer, Marcel Reinmuth, Paul O. Ouma, Sven Lautenbach, Emelda A. Okiro, Till Bärnighausen, Alexander Zipf

**Affiliations:** 1 Division of Primary Care and Population Health, Department of Medicine, Stanford University, Stanford, CA, USA;; 2 Heidelberg Institute of Global Health, Heidelberg University, Heidelberg, Germany;; 3 Institute of Geography, Heidelberg University, Heidelberg, Germany;; 4 HeiGIT at Heidelberg University, Heidelberg, Germany;; 5 Population Health Unit, Kenya Medical Research Institute (MFL)-Wellcome Trust Research Programme, Nairobi, Kenya;; 6 Centre for Tropical Medicine and Global Health, Nuffield Department of Medicine, University of Oxford, Oxford, United Kingdom; 7 Department of Global Health and Population, Harvard T.H. Chan School of Public Health, Boston, MA, USA;; 8 Africa Health Research Institute, Somkhele, South Africa

## Abstract

**Background:**

SARS-CoV-2, the virus causing coronavirus disease 2019 (COVID-19), is rapidly spreading across sub-Saharan Africa (SSA). Hospital-based care for COVID-19 is particularly often needed among older adults. However, a key barrier to accessing hospital care in SSA is travel time. To inform the geographic targeting of additional healthcare resources, this study aimed to determine the estimated travel time at a 1km × 1km resolution to the nearest hospital and to the nearest healthcare facility of any type for adults aged 60 years and older in SSA.

**Methods:**

We assembled a unique dataset on healthcare facilities’ geolocation, separately for hospitals and any type of healthcare facility (including primary care facilities) and including both private- and public-sector facilities, using data from the OpenStreetMap project and the KEMRI Wellcome Trust Programme. Population data at a 1km × 1km resolution was obtained from WorldPop. We estimated travel time to the nearest healthcare facility for each 1km × 1km raster using a cost-distance algorithm.

**Findings:**

9.6% (95% CI: 5.2% – 16.9%) of adults aged ≥60 years had an estimated travel time to the nearest hospital of longer than six hours, varying from 0.0% (95% CI: 0.0% – 3.7%) in Burundi and The Gambia, to 40.9% (95% CI: 31.8% – 50.7%) in Sudan. 11.2% (95% CI: 6.4% – 18.9%) of adults aged ≥60 years had an estimated travel time to the nearest healthcare facility of any type (whether primary or secondary/tertiary care) of longer than three hours, with a range of 0.1% (95% CI: 0.0% – 3.8%) in Burundi to 55.5% (95% CI: 52.8% – 64.9%) in Sudan. Most countries in SSA contained populated areas in which adults aged 60 years and older had a travel time to the nearest hospital of more than 12 hours and to the nearest healthcare facility of any type of more than six hours. The median travel time to the nearest hospital for the fifth of adults aged ≥60 years with the longest travel times was 348 minutes (IQR: 240 – 576 minutes) for the entire SSA population, ranging from 41 minutes (IQR: 34 – 54 minutes) in Burundi to 1,655 minutes (IQR: 1065 – 2440 minutes) in Gabon.

**Interpretation:**

Our high-resolution maps of estimated travel times to both hospitals and healthcare facilities of any type can be used by policymakers and non-governmental organizations to help target additional healthcare resources, such as new make-shift hospitals or transport programs to existing healthcare facilities, to older adults with the least physical access to care. In addition, this analysis shows precisely where population groups are located that are particularly likely to under-report COVID-19 symptoms because of low physical access to healthcare facilities. Beyond the COVID-19 response, this study can inform countries’ efforts to improve care for conditions that are common among older adults, such as chronic non-communicable diseases.

**Funding:**

Bill & Melinda Gates Foundation

## Introduction

Declared a pandemic by the World Health Organization on March 11 2020,^1^ SARS-CoV-2 has caused over nine million confirmed infections and the disease it can trigger - coronavirus disease 2019 (COVID-19) - has led to almost 500,000 reported deaths across the world by late June 2020.^2^ While low testing numbers do not allow for a reliable assessment of the extent of the epidemic in sub-Saharan Africa (SSA), the region had over 300,000 reported infections and almost 9,000 deaths due to SARS-CoV-2 as of June 24 2020.^2^ Epidemiological modelling suggests that COVID-19 could lead to between 300,000 and 2.5 million deaths in SSA, depending on modelling assumptions and the mitigation policies that are adopted.^3^

There are numerous barriers to receiving high-quality healthcare in SSA, including financial barriers to accessing care, weak supply chains, and understaffing of healthcare facilities.^4^ However, physical distance to the nearest healthcare facility - and the associated requirements for transport options, cost of transport, and time lost from other income-generating activities - consistently figure as one of the most important barriers to accessing both hospital-based and primary care in the region.^5–10^

Travel time to the nearest healthcare facility and the nearest hospital will likely also play an important role in the ability of health systems in SSA to respond to SARS-CoV-2 for three main reasons. First, physical access to hospitals will influence whether and how timely individuals with COVID-19 are able to seek healthcare. While many hospitals in SSA are not able to provide mechanical ventilation,^11,12^ other critical components of care for those with severe COVID-19, such as hemodynamic support, supplemental oxygen therapy, and treatment of co-infections (e.g., bacterial pneumonia), are more readily available in hospitals in SSA.^13,14,15^ Second, physical access to a healthcare facility of any type will impact whether and when during the disease course individuals with COVID-19 contact the healthcare system. These care-seeking decisions in turn have important ramifications for whether the health system is notified of COVID-19 cases and, thus, the monitoring of the epidemic, particularly in settings that are unable to conduct large-scale community-based testing for SARS-CoV-2 infections. Third, if these options become widely available in SSA in the future, physical access to healthcare facilities will likely affect to what degree individuals with COVID-19 take up effective medications against the condition and possibly also to what degree they are able to access a vaccine against SARS-CoV-2.

Having a detailed understanding of where population groups are located that are both vulnerable to COVID-19 and have long travel times to the nearest healthcare facility can inform where additional healthcare resources (e.g., the establishment of makeshift hospitals or programs to ensure availability of transport to hospitals) are most needed. In addition, such knowledge would allow for pinpointing those geographic areas that are most likely to harbour the most cases of COVID-19 that were unreported due to lack of physical access to care, which in turn can inform the geographic targeting of testing efforts. More broadly, understanding where older adults reside who have the least physical access to healthcare can inform health systems’ efforts to improve care for conditions that are common in this age group, particularly chronic non-communicable diseases and their sequelae. By assembling a unique dataset from both crowd-sourced data and official records by governments and international organizations, this study, therefore, aimed to create highly detailed maps of estimated travel time for adults aged 60 yeas and older in SSA to both the nearest hospital and the closest healthcare facility of any type.

## Methods

### Data sources for the geolocation of healthcare facilities:

We used two data sources: healthcare facility data from the OpenStreetMap (OSM) project and a geocoded inventory of healthcare facilities published by the KEMRI Wellcome Trust Research Programme.^16,17^ OSM is a collaborative online platform to map, edit, and share geospatial data globally. Started in 2004, OSM evolved from a crowd-sourced alternative for proprietary map data providers, to an important complementary data source in humanitarian settings,^18,19^ and a widely used source of information for base maps as well as for critical infrastructure in the global South.^20^ Querying the database for all objects with either “amenity” or “healthcare” as key and either “hospital”, “clinic”, or “doctors” as value, we extracted all healthcare facilities mapped in OSM with their geographic coordinates using the ohsome api.^21^ We identified 24,571 healthcare facilities of which 13,392 were tagged as hospitals.

The second data source used in this analysis was an inventory of 98,745 public-sector healthcare facilities across all countries of SSA except for five small island states (Cape Verde, Comoros, Mauritius, Sao Tomé & Principé, and the Seychelles), assembled and published by the KEMRI Wellcome Trust Programme.^17^ The primary source of data were master facility lists (MFLs) of national Ministries of Health and documentation by United Nations and non-governmental organizations. Additional sources included websites and data portals by SSA governments, health sector reports, and personal communications. We henceforth refer to this dataset as the MFL dataset. 52% of the healthcare facilities contained in the data were manually geocoded by the KEMRI Wellcome Trust Programme team. For Sudan, Guinea-Bissau, and ten out of 18 provinces in Angola, the MFL dataset contains the geographic coordinates of hospitals only. The MFL dataset included 92,245 healthcare facilities in our study countries of which 4,720 were classified as hospitals.

### Data source for the geolocation of the population:

Population counts for adults aged 60 years and older were obtained from the WorldPop project.^22^ The counts reflect projections for 2020 at a spatial resolution of 1 km^2^. The WorldPop project built this dataset using a semi-automated dasymetric mapping method that employs a Random Forest classifier to disaggregate census data at the level of national census tracks to 1 km^2^ areas.^23^ Predictors used were geographical properties, such as topography, climate, and land cover, as well as the density of human-built features, such as nightlights, roads, and buildings.

### Estimating travel time to the nearest healthcare facility:

We merged the OSM and MFL dataset such that our estimated travel times are the travel time to the nearest healthcare facility, regardless of the data source in which the facility is listed. We chose this strategy because both datasets are more likely to be missing existing healthcare facilities than to falsely list a non-existing healthcare facility. We estimated travel time to the nearest healthcare facility separately for hospitals and healthcare facilities of any type. Hospitals were chosen as one entity of interest because most healthcare interventions to care for individuals with severe COVID-19 require hospital-based care. Healthcare facilities of any type were chosen as an additional entity of interest because physical access to any healthcare facility likely influences the degree to which individuals with COVID-19 present to the healthcare system and, thus, the extent to which the healthcare system is made aware of new COVID-19 cases. In the absence of community-based screening for SARS-CoV-2 infections, areas with low physical access to healthcare facilities of any type may, thus, have a disproportionately high number of unreported COVID-19 cases.

We used the AccessMod tool (version 5.6.33) to estimate travel time.^25^ AccessMod employs a raster-based cost-distance algorithm, whereby each raster cell is associated with a cost value that determines the time required to travel through this cell. The cost for each cell was modelled using the 2018 Copernicus Global Land Cover product and the Shuttle Radar Topography Mission (SRTM v. 4) digital elevation model as basic impedance surface.^24,25^ In addition, we used OSM data to ascertain the road network and locations of rivers and open water (which were considered barriers for any kind of travel). Aligning with previous studies in SSA,^27^ we assigned a travel speed of 100km/h to motorways and primary roads, 50km/h to secondary roads, and 30 km/h to tertiary roads. Barren land and built-up areas were assigned a 5km/h and forests a 2 km/h walking speed. The model was created at a spatial resolution of 100m^2^. For both OSM and MFL data, we calculated the travel time from each cell to the nearest healthcare facility of any type and the closest hospital. These results were then aggregated to a 1km^2^ resolution to match the resolution of the WorldPop population data. Our analyses assumed that individuals were able to cross national borders to reach the nearest healthcare facility.

### Statistical analysis:

We plotted the distribution of travel time, separately for hospitals and healthcare facilities of any type, in each country. In addition, we mapped the estimated travel time at a 1km × 1km resolution both as a continuous variable and when categorizing travel time into less than two hours, two to six hours, six to 12 hours, and more than 12 hours for the nearest hospital, and less than one hour, one to two hours, two to six hours, and more than six hours for the nearest healthcare facility of any type. When summarizing our data as binomial proportions, we show two-sided 95% confidence intervals using the Wilson score interval.^26^ Other than the calculation of travel time, which was carried out using AccessMod 5, all analyses were conducted in R version 3.6.3.^27^

## Results

### Sample characteristics:

Across our two datasets, the population density of healthcare facilities varied from 0.067 facilities per 100,000 in Burkina Faso (MFL data) to 11.008 facilities per 100,000 in the Central African Republic (OSM data) for hospitals, and from 0.257 facilities per 100,000 in Somalia (OSM data) to 28.983 facilities per 100,000 in Gabon (MFL data) for healthcare facilities of any type.

### Distribution of travel time to the nearest healthcare facility:

Across SSA, the proportion of adults aged 60 years and older with an estimated travel time of greater than six hours to the nearest hospital was 9.6% (95% CI: 5.2 – 16.9) across SSA, varying from 0.0% (95% CI: 0.0 – 3.7) in Burundi and The Gambia to 40.9% (95% CI: 31.8 – 50.7) in Sudan (Figure S1). For healthcare facilities of any type and using a travel time cut-off of two hours, the corresponding proportions were 15.9% (95% CI: 10.1 – 24.4) across SSA, ranging from 0.4% (95% CI: 0.0 – 4.4) in Burundi to 59.4% (95% CI: 50.1 – 69.0) in Sudan (Figure S2).

**Figure 1.**
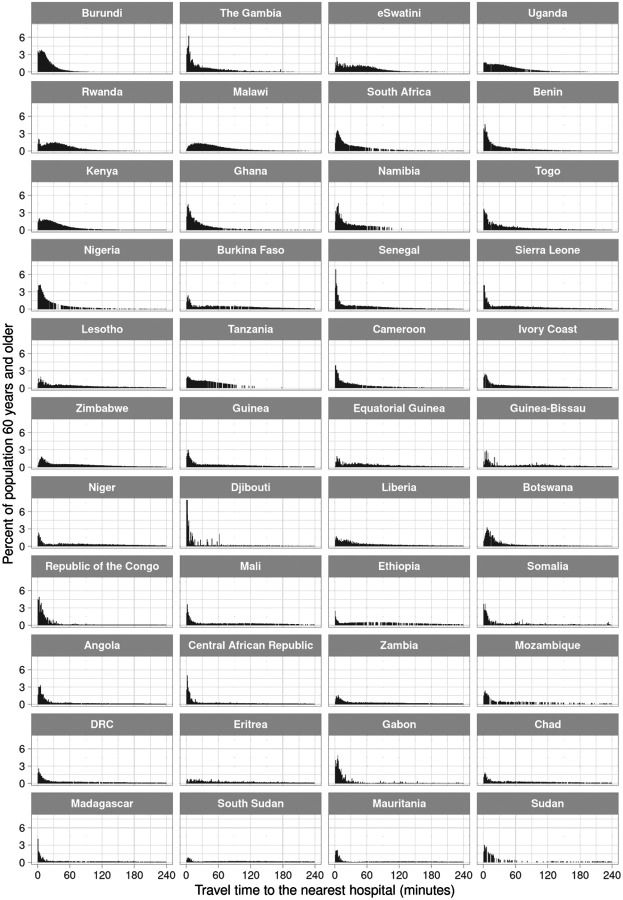
Distribution of travel time to the nearest hospital for adults aged 60 years and older, by country^1^ Abbreviations: DRC=Democratic Republic of the Congo ^1^ Countries were ordered in ascending order by the proportion of adults aged 60 years and older in their population who reside in a 1km × 1km area that has an estimated travel time >6 hours to the nearest hospital.

**Figure 2.**
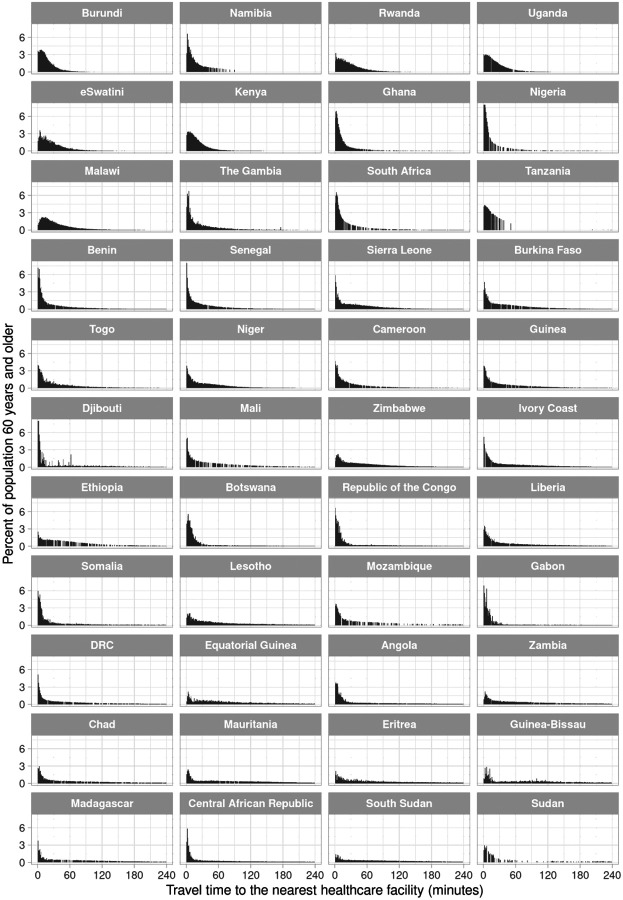
Distribution of travel time to the nearest healthcare facility of any type for adults aged 60 years and older, by country^1^ Abbreviations: DRC=Democratic Republic of the Congo ^1^ Countries were ordered in ascending order by the proportion of adults aged 60 years and older in their population who reside in a 1km × 1km area that has an estimated travel time >2 hours to the nearest healthcare facility.

The shape of the distribution of travel time to the nearest hospital for adults aged 60 years and older varied greatly across countries (Figure 1). It ranged from a distribution in which the vast majority of the population is within 60 minutes travel time and there being virtually no right tail to the distribution (e.g., in Burundi), to distributions in which the population was almost equally spread across the range of travel time from 0 to 240 minutes (e.g., in Ethiopia). For the nearest healthcare facility of any type, in contrast, the distribution was more heavily skewed towards very short travel times (Figure 2), with the proportion of adults aged 60 years and older who reside within 30 minutes of traveling to the nearest facility being at least 25% in 43 of our 44 study countries. The travel time distributions are shown separately for the MFL and the OSM dataset in Figures S3 to S6.

### Maps of travel time to the nearest hospital:

**Figure 3.**
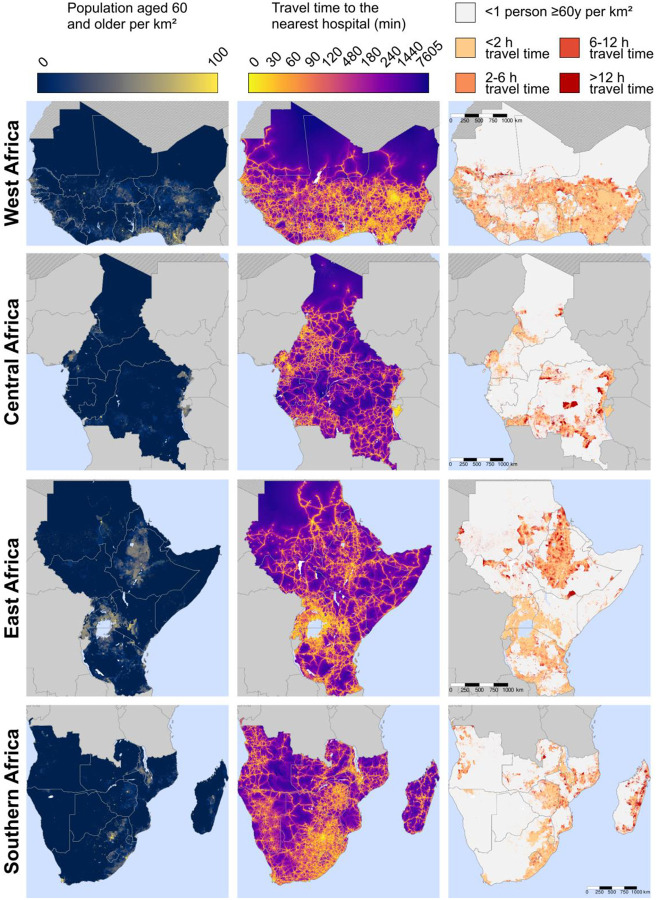
Maps of travel time to the nearest hospital for adults ≥60 years, by region

Figure 3 shows the population density of adults aged 60 years and older as well as the estimated travel time among these adults to the nearest hospital at a 1km × 1km resolution. The third column of maps focusses on areas with at least one adult aged 60 years and older per km^2^ and categorizes travel time into less than two hours, two hours to less than six hours, six hours to less than 12 hours, and more than 12 hours. This column shows that almost all countries in SSA contain areas that have both at least one person per km^2^ and an estimated travel time to the nearest hospital of greater than 12 hours (indicated as the areas in dark red). Countries with many of these 1km^2^ areas with poor physical access to hospital care included the Democratic Republic of the Congo, Madagascar, Ethiopia, Sudan, South Sudan, Mozambique, and Mauritania. More detailed maps created separately for each country are shown in Figures S7 – S50. Regional maps created using only the MFL and only the OSM data are shown in Figure S51 and Figure S52, respectively.

### Maps of travel time to the nearest healthcare facility of any type:

Figure 4 shows the same variables as Figure 3 but for healthcare facilities of any type (as opposed to hospitals only). Countries with a high number of these 1km^2^ areas with poor physical access to a healthcare facility included the Democratic Republic of the Congo, Sudan, Ethiopia, Madagascar, Mozambique, South Sudan, and Angola. Maps created separately for each country are shown in Figures S53 – S96. Regional maps created using only the MFL and only the OSM data are shown in Figure S97 and Figure S98, respectively.

## Discussion

With approximately 10% of adults aged 60 years and older across SSA having an estimated travel time to the nearest hospital of greater than six hours, physical access to healthcare will likely play a major role in whether older adults in this world region will be able to seek care for COVID-19. By precisely identifying where older adults are residing who have a particularly high estimated travel time to the nearest hospital, our high-resolution maps can inform policy makers as to where interventions to increase physical access to hospital care are needed most urgently. Such interventions could include transport programs to existing hospitals and the establishment of make-shift hospitals. In addition, our maps of estimated travel time to the nearest healthcare facility of any type could help guide policy makers as to which populations are least likely to present to the healthcare system when they suffer from COVID-19 symptoms due to a lack of physical access to healthcare. This information in turn could be helpful in interpreting monitoring data on new cases of COVID-19 from different areas within countries, and in targeting testing efforts to those populations that have the greatest need for such tests.

The usefulness and policy relevance of this analysis goes beyond informing countries’ response to the SARS-CoV-2 pandemic. Physical access - that is, the time required to travel to a healthcare facility, available transport options, and costs for transport - is thought to be one of the main barriers to accessing healthcare in SSA.^5–10^ Yet, there is currently very little detailed evidence on how physical access to healthcare varies across SSA, particularly within countries. Such evidence, however, is crucial to guide policy makers in identifying those areas that have the greatest need for community outreach programs, the establishment of new healthcare facilities, and improved transport infrastructure. Our study helps fill this important evidence gap for older adults in the region and is, thus, of high relevance for informing countries’ efforts to improve care for conditions that affect older adults, particularly chronic non-communicable diseases. Specifically, this study builds on the, to our knowledge, three existing studies that have mapped physical access to healthcare in SSA at a geographic level below countries or administrative regions within countries. Specifically, Ouma et al. have examined access to emergency hospital care in SSA.^28^ This study differs from ours in that it focussed on women of child-bearing age (15 to 49 years) rather than older adults, did not include primary healthcare facilities nor any private-sector healthcare facilities, did not use OSM data, employed a cut-off of travel time less or greater than two hours (based on a target set by the Lancet Commissions for Global Surgery 2030^29^) rather than analysing the whole distribution, analysed data from 2015, and did not provide detailed country-by-country maps. Other relevant studies have focussed on the effect of physical access to a healthcare facility on the probability of seeking care for a febrile episode in children,^30^ and estimated travel time to healthcare facilities among populations at risk of viral haemorrhagic fevers.^31^

Another key contribution of our study is the collation of a new dataset on geo-tagged healthcare facilities in SSA. By making this dataset available in the public domain and including the location of other age groups (not merely adults aged 60 years and older), we allow researchers and policy makers to run their own analyses for a variety of demographic groups and add to (or alter) the list of geo-tagged healthcare facilities in a country. There is currently no authoritative source of the location of all healthcare facilities in SSA. We have combined data from the only two existing sources of data for the geolocation of healthcare facilities in the region. We chose this approach because it is highly likely that neither dataset is complete, as is evidenced by the fact that in some countries the MFL dataset listed a higher number of healthcare facilities than the OSM data while the opposite was the case in other countries. Because the OSM project relies on volunteers to map and tag healthcare facilities, the OSM data by itself is particularly likely to underestimate the density of healthcare facilities in an area. The fact that OSM contained a higher number of healthcare facilities in many countries than did the MFL dataset, particularly for hospitals, is, however, encouraging in that OSM appears to be a useful source of information for the geolocation of healthcare facilities. Importantly, OSM data are likely to improve over time as coverage of smartphones increases in SSA and more volunteers map out their local areas. We will, therefore, update our dataset on a regular basis. Moving forward, it will be important to continuously monitor the validity of the data entered into the OSM and MFL dataset; a task that would ideally be accomplished by Ministries of Health in SSA.

This study has several limitations. First and foremost, while we (by combining MFL with OSM data) likely provide the most comprehensive source of data for the geolocation of healthcare facilities that is currently available, it is still likely to miss a substantial proportion of healthcare facilities. The degree to which this is the case probably varies between countries as both the participation in the OSM project and the degree to which documentation used for the MFL dataset were available and complete differ across countries.^32^ Second, we do not have any data on the readiness of healthcare facilities to provide care, nor the quality of care provided at healthcare facilities. Both are likely to vary across and within countries.^33^ Third, a limitation of our analysis for the COVID-19 response is that governments may decide that not all hospitals in a country should be providing care for COVID-19. Fourth, our analysis does not take into account that vulnerability to COVID-19 is likely affected by factors beyond age that vary across and within countries, including HIV, tuberculosis, and malnutrition. We decided against including these factors in our analysis because which conditions increase the risk of experiencing a severe disease course, and to what degree, is still largely unknown, especially in SSA. Lastly, our travel time numbers are approximations that, for example, do not take into account the frequency of transport services and assign an estimated (rather than measured) travel speed to different types of roads. Lastly, our analysis focusses on only one aspect of access to healthcare and thus does not, for instance, take into account financial barriers to accessing care.

Most countries in SSA contain areas in which older adults have little to no physical access to a hospital and (albeit to a lesser extent) healthcare facilities of any type. If COVID-19 becomes a generalized epidemic that infects large swaths of countries’ populations in the region, then it will be older adults living in these areas who are in a particularly high need for either improved transport options to existing hospitals or the provision of make-shift hospital care. Beyond their usefulness for the COVID-19 response, our maps could inform health system planning for conditions that commonly affect older adults, such as expansions of care for myocardial infarctions and strokes.

## Supplementary Material

Supplement 2020

Supplement 2020

## Figures and Tables

**Figure 4.**
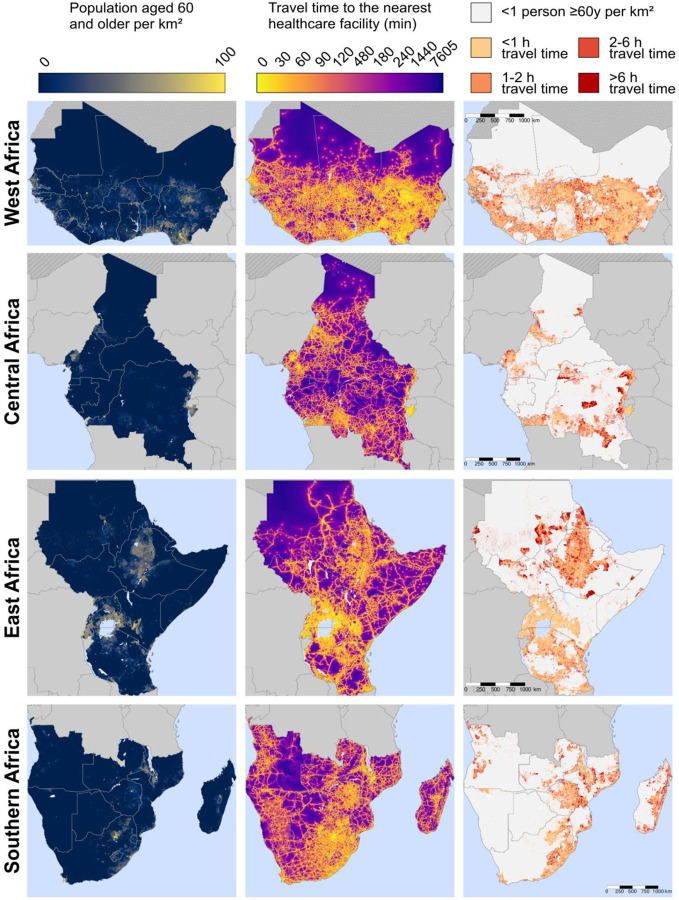
Maps of travel time to the nearest healthcare facility for adults ≥60 years, by region

**Table 1.** Population and number of healthcare facilities by country

Country	Population (millions)		Number of healthcare facilities						Number of healthcare facilities per 100,000 population					
	*Total*	*Age 60+*	MFL			OSM			MFL			OSM		
			*Primary care*	*Hospitals*	*Total*	*Primary care*	*Hospitals*	*Total*	*Primary care*	*Hospitals*	*Total*	*Primary care*	*Hospitals*	*Total*
*Central Africa*														
Burundi	13.097	0.534	619	49	668	22	1317	1339	4.726	0.374	5.101	0.168	10.056	10.224
Cameroon	26.265	1.673	2825	181	3006	479	541	1020	10.756	0.689	11.445	1.824	2.060	3.884
Central African Republic	5.360	0.143	526	20	546	18	590	608	9.814	0.373	10.187	0.336	11.008	11.344
Chad	16.435	0.747	1164	79	1243	91	140	231	7.082	0.481	7.563	0.554	0.852	1.406
DRC	89.636	3.908	13964	432	14396	1385	724	2109	15.579	0.482	16.061	1.545	0.808	2.353
Equatorial Guinea	0.925	0.044	28	14	42	2	5	7	3.027	1.514	4.541	0.216	0.541	0.757
Gabon	1.829	0.124	513	17	530	153	56	209	28.053	0.930	28.983	8.367	3.062	11.429
Republic of the Congo	5.244	0.176	305	27	332	81	87	168	5.816	0.515	6.331	1.545	1.659	3.204
*East Africa*														
Djibouti	0.671	0.023	51	13	64	6	27	33	7.598	1.937	9.535	0.894	4.022	4.916
Eritrea	5.955	0.259	252	20	272	2	19	21	4.232	0.336	4.568	0.034	0.319	0.353
Ethiopia	111.731	4.780	5838	164	6002	187	272	459	5.225	0.147	5.372	0.167	0.243	0.411
Kenya	51.513	2.202	1374	394	1768	283	811	1094	2.667	0.765	3.432	0.549	1.574	2.124
Rwanda	13.299	0.512	538	48	586	55	82	137	4.046	0.361	4.406	0.414	0.617	1.030
Somalia	12.459	0.574	548	61	609	4	28	32	4.398	0.490	4.888	0.032	0.225	0.257
South Sudan	14.112	0.545	1678	41	1719	36	68	104	11.891	0.291	12.181	0.255	0.482	0.737
Sudan	45.292	2.374	5	259	264	89	300	389	0.011	0.572	0.583	0.197	0.662	0.859
Tanzania	61.897	2.762	5937	222	6159	1019	977	1996	9.592	0.359	9.950	1.646	1.578	3.225
Uganda	45.982	2.043	3580	121	3701	1731	546	2277	7.786	0.263	8.049	3.765	1.187	4.952
*Southern Africa*														
Angola	29.150	1.043	1289	150	1439	76	162	238	4.422	0.515	4.936	0.261	0.556	0.816
Botswana	2.443	0.130	560	28	588	81	77	158	22.924	1.146	24.071	3.316	3.152	6.468
eSwatini	1.362	0.060	124	6	130	6	25	31	9.107	0.441	9.548	0.441	1.836	2.277
Lesotho	2.232	0.187	92	14	106	20	44	64	4.121	0.627	4.748	0.896	1.971	2.867
Madagascar	27.555	0.969	2497	117	2614	61	218	279	9.062	0.425	9.486	0.221	0.791	1.013
Malawi	20.052	0.843	574	83	657	36	194	230	2.863	0.414	3.276	0.180	0.967	1.147
Mozambique	31.732	1.452	1498	61	1559	743	148	891	4.721	0.192	4.913	2.342	0.466	2.808
Namibia	2.734	0.164	322	37	359	48	83	131	11.780	1.354	13.133	1.756	3.036	4.792
South Africa	56.423	4.614	3951	329	4280	255	644	899	7.002	0.583	7.586	0.452	1.141	1.593
Zambia	18.784	0.713	1162	89	1251	62	129	191	6.186	0.474	6.660	0.330	0.687	1.017
Zimbabwe	17.363	0.927	1031	170	1201	98	148	246	5.938	0.979	6.917	0.564	0.852	1.417
*West Africa*														
Benin	12.418	0.717	771	48	819	249	214	463	6.209	0.387	6.595	2.005	1.723	3.728
Burkina Faso	20.829	1.061	2368	14	2382	297	175	472	11.369	0.067	11.436	1.426	0.840	2.266
Ghana	30.256	1.582	1679	178	1857	275	333	608	5.549	0.588	6.138	0.909	1.101	2.010
Guinea	14.260	1.024	1482	36	1518	304	97	401	10.393	0.252	10.645	2.132	0.680	2.812
Guinea-Bissau	2.027	0.098	0	8	8	11	18	29	0.000	0.395	0.395	0.543	0.888	1.431
Ivory Coast	25.170	0.911	1638	95	1733	795	260	1055	6.508	0.377	6.885	3.159	1.033	4.191
Liberia	4.953	0.243	668	33	701	126	51	177	13.488	0.666	14.154	2.544	1.030	3.574
Mali	20.542	1.154	1446	18	1464	683	140	823	7.039	0.088	7.127	3.325	0.682	4.006
Mauritania	4.509	0.282	626	19	645	30	70	100	13.883	0.421	14.304	0.665	1.552	2.218
Niger	24.140	1.280	2790	41	2831	154	113	267	11.557	0.170	11.727	0.638	0.468	1.106
Nigeria	205.773	10.227	18714	887	19601	557	2888	3445	9.094	0.431	9.526	0.271	1.403	1.674
Senegal	17.384	0.943	1198	27	1225	310	184	494	6.891	0.155	7.047	1.783	1.058	2.842
Sierra Leone	6.951	0.433	1060	28	1088	150	145	295	15.249	0.403	15.652	2.158	2.086	4.244
The Gambia	2.186	0.107	91	5	96	11	58	69	4.162	0.229	4.391	0.503	2.653	3.156
Togo	8.296	0.534	149	37	186	95	172	267	1.796	0.446	2.242	1.145	2.073	3.218
*Sub-Saharan Africa*	*1131.227*	*55.123*	*87525*	*4720*	*92245*	*11176*	*13380*	*24556*	*7.737*	*0.417*	*8.154*	*0.988*	*1.183*	*2.171*

Abbreviations: MFL=Master Facility List data; OSM=OpenStreetMap dataset; DRC=Democratic Republic of the Congo

## Research in context

### Evidence before this study:

We searched MEDLINE from January 1966 until May 2020 for studies with variations of the words ‘physical access’, ‘distance’, ‘travel time’, ‘hospital’, and ‘healthcare facility’ in the title or abstract. To date, the only studies to systematically map physical access to healthcare facilities in sub-Saharan Africa at a high resolution examined access to emergency hospital care (with a focus on women of child-bearing age), access to care for children with fever, and travel time to the nearest healthcare facility for specific populations at risk of viral haemorrhagic fevers.

### Added value of this study:

The added value of this study is threefold. First, we assembled a new dataset of GPS-tagged healthcare facilities, which combines two unique data sources for the geolocation of healthcare facilities across sub-Saharan Africa: one-based on crowd-sourced data from OpenStreetMap and one based on information from ministries of health, health management information systems, government statistical agencies, and international organizations. Second, this is the first study to comprehensively map both hospitals and primary healthcare facilities, and including both public- and private-sector facilities, across sub-Saharan Africa. Third, because the COVID-19 epidemic causes a far higher need for hospital services among older than younger population groups, we focus on physical access to healthcare for the population aged 60 years and older, which is a population group that is rarely studied in investigations of healthcare demand and supply in the region. As such, our maps can inform not only the health system response to COVID-19, but more generally to conditions that are common among older adults in the region, particularly chronic non-communicable diseases and their sequelae.

### Implications of all the available evidence:

Low physical access to healthcare in sub-Saharan Africa will be a major barrier to receiving care for adults aged 60 years and older with COVID-19. However, there is a wide degree of variation in physical access to healthcare facilities for older adults in the region both between and within countries, which likely has an important bearing on the extent to which different population groups within countries are able to access care for COVID-19. Likewise, in those areas with a long travel time to the nearest healthcare facility of any type (of which there are a considerable number in most countries), symptomatic cases of COVID-19 are particularly unlikely to be reported to the healthcare system. Our high-resolution maps for each region and country in sub-Saharan Africa provide precise information about this geographic variation for local, national, and regional policymakers as well as non-governmental organizations.
