# Supplementary material for: Mapping physical access to healthcare for older adults in sub-Saharan Africa: A cross-sectional analysis with implications for the COVID-19 response
Source: medRxiv. 2020 Aug 26:2020.07.17.20152389. Preprint. [Version 3] doi: 10.1101/2020.07.17.20152389 (PMC7386521; doi:10.1101/2020.07.17.20152389)
Supplement: Supplement 2020 [file 89961-2020.07.17.20152389-1.pdf]

Supplementary appendix

Figure S1. Distribution of travel time to the nearest hospital, by country 7
Figure S2. Distribution of travel time to the nearest healthcare facility of any type, by country 8
Figure S3. Histogram of travel time to the nearest hospital in the MFL dataset for adults aged 60 years and older, by country 9
Figure S4. Histogram of travel time to the nearest hospital in the OSM dataset for adults aged 60 years and older, by country 10
Figure S5. Histogram of travel time to the nearest healthcare facility (of any type) in the MFL dataset for adults aged 60 years and older, by country 11
Figure S6. Histogram of travel time to the nearest healthcare facility (of any type) in the OSM dataset for adults aged 60 years and older, by country 12
Figure S7. Angola map of travel time to the nearest hospital for adults aged ≥ 60 years 13
Figure S8. Benin map of travel time to the nearest hospital for adults aged ≥ 60 years 14
Figure S9. Botswana map of travel time to the nearest hospital for adults aged ≥ 60 years 15
Figure S10. Burkina Faso map of travel time to the nearest hospital for adults aged ≥ 60 years 16
Figure S11. Burundi map of travel time to the nearest hospital for adults aged ≥ 60 years 17
Figure S12. Cameroon map of travel time to the nearest hospital for adults aged ≥ 60 years 18
Figure S13. Central African Republic map of travel time to the nearest hospital for adults aged ≥ 60 years 19
Figure S14. Chad map of travel time to the nearest hospital for adults aged ≥ 60 years 20
Figure S15. Djibouti map of travel time to the nearest hospital for adults aged ≥ 60 years 21
Figure S16. DRC map of travel time to the nearest hospital for adults aged ≥ 60 years 22
Figure S17. Equatorial Guinea map of travel time to the nearest hospital for adults aged ≥ 60 years 23
Figure S18. Eritrea map of travel time to the nearest hospital for adults aged ≥ 60 years 24

Figure S19. eSwatini map of travel time to the nearest hospital for adults aged ≥ 60 years 25
Figure S20. Ethiopia map of travel time to the nearest hospital for adults aged ≥ 60 years 26
Figure S21. Gabon map of travel time to the nearest hospital for adults aged ≥ 60 years 27
Figure S22. Ghana map of travel time to the nearest hospital for adults aged ≥ 60 years 28
Figure S23. Guinea map of travel time to the nearest hospital for adults aged ≥ 60 years 29
Figure S24. Guinea-Bissau map of travel time to the nearest hospital for adults aged ≥ 60 years 30
Figure S25. Ivory Coast map of travel time to the nearest hospital for adults aged ≥ 60 years 31
Figure S26. Kenya map of travel time to the nearest hospital for adults aged ≥ 60 years 32
Figure S27. Lesotho map of travel time to the nearest hospital for adults aged ≥ 60 years 33
Figure S28. Liberia map of travel time to the nearest hospital for adults aged ≥ 60 years 34
Figure S29. Madagascar map of travel time to the nearest hospital for adults aged ≥ 60 years 35
Figure S30. Malawi map of travel time to the nearest hospital for adults aged ≥ 60 years 36
Figure S31. Mali map of travel time to the nearest hospital for adults aged ≥ 60 years 37
Figure S32. Mauritania map of travel time to the nearest hospital for adults aged ≥ 60 years 38
Figure S33. Mozambique map of travel time to the nearest hospital for adults aged ≥ 60 years 39
Figure S34. Namibia map of travel time to the nearest hospital for adults aged ≥ 60 years 40
Figure S35. Niger map of travel time to the nearest hospital for adults aged ≥ 60 years 41
Figure S36. Nigeria map of travel time to the nearest hospital for adults aged ≥ 60 years 42
Figure S37. Republic of the Congo map of travel time to the nearest hospital for adults aged ≥ 60 years 43

Figure S38. Rwanda map of travel time to the nearest hospital for adults aged ≥ 60 years 44
Figure S39. Senegal map of travel time to the nearest hospital for adults aged ≥ 60 years 45
Figure S40. Sierra Leone map of travel time to the nearest hospital for adults aged ≥ 60 years 46
Figure S41. Somalia map of travel time to the nearest hospital for adults aged ≥ 60 years 47
Figure S42. South Africa map of travel time to the nearest hospital for adults aged ≥ 60 years 48
Figure S43. South Sudan map of travel time to the nearest hospital for adults aged ≥ 60 years 49
Figure S44. Sudan map of travel time to the nearest hospital for adults aged ≥ 60 years 50
Figure S45. Tanzania map of travel time to the nearest hospital for adults aged ≥ 60 years 51
Figure S46. The Gambia map of travel time to the nearest hospital for adults aged ≥ 60 years 52
Figure S47. Togo map of travel time to the nearest hospital for adults aged ≥ 60 years 53
Figure S48. Uganda map of travel time to the nearest hospital for adults aged ≥ 60 years 54
Figure S49. Zambia map of travel time to the nearest hospital for adults aged ≥ 60 years 55
Figure S50. Zimbabwe map of travel time to the nearest hospital for adults aged ≥ 60 years 56
Figure S51. Maps of travel time to the nearest hospital for adults ≥ 60 years, by region based on the MFL dataset 57
Figure S52. Maps of travel time to the nearest hospital for adults ≥ 60 years, by region based on the OSM dataset 58
Figure S53. Angola map of travel time to the nearest healthcare facility for adults aged ≥ 60 years 59
Figure S54. Benin map of travel time to the nearest healthcare facility for adults aged ≥ 60 years 60
Figure S55. Botswana map of travel time to the nearest healthcare facility for adults aged ≥ 60 years 61
Figure S56. Burkina Faso map of travel time to the nearest healthcare facility for adults aged ≥ 60 years 62

Figure S57. Burundi map of travel time to the nearest healthcare facility for adults aged ≥ 60 years 63

Figure S58. Cameroon map of travel time to the nearest healthcare facility for adults aged ≥ 60 years 64

Figure S59. Central African Republic map of travel time to the nearest healthcare facility for adults aged ≥ 60 years 65

Figure S60. Chad map of travel time to the nearest healthcare facility for adults aged ≥ 60 years 66

Figure S61. Djibouti map of travel time to the nearest healthcare facility for adults aged ≥ 60 years 67

Figure S62. DRC map of travel time to the nearest healthcare facility for adults aged ≥ 60 years 68

Figure S63. Equatorial Guinea map of travel time to the nearest healthcare facility for adults aged ≥ 60 years 69

Figure S64. Eritrea map of travel time to the nearest healthcare facility for adults aged ≥ 60 years 70

Figure S65. eSwatini map of travel time to the nearest healthcare facility for adults aged ≥ 60 years 71

Figure S66. Ethiopia map of travel time to the nearest healthcare facility for adults aged ≥ 60 years 72

Figure S67. Gabon map of travel time to the nearest healthcare facility for adults aged ≥ 60 years 73

Figure S68. Ghana map of travel time to the nearest healthcare facility for adults aged ≥ 60 years 74

Figure S69. Guinea map of travel time to the nearest healthcare facility for adults aged ≥ 60 years 75

Figure S70. Guinea-Bissau map of travel time to the nearest healthcare facility for adults aged ≥ 60 years 76

Figure S71. Ivory Coast map of travel time to the nearest healthcare facility for adults aged ≥ 60 years 77

Figure S72. Kenya map of travel time to the nearest healthcare facility for adults aged ≥ 60 years 78

Figure S73. Lesotho map of travel time to the nearest healthcare facility for adults aged ≥ 60 years 79

Figure S74. Liberia map of travel time to the nearest healthcare facility for adults aged ≥ 60 years 80

Figure S75. Madagascar map of travel time to the nearest healthcare facility for adults aged ≥ 60 years 81

Figure S76. Malawi map of travel time to the nearest healthcare facility for adults aged ≥ 60 years 82
Figure S77. Mali map of travel time to the nearest healthcare facility for adults aged ≥ 60 years 83
Figure S78. Mauritania map of travel time to the nearest healthcare facility for adults aged ≥ 60 years 84
Figure S79. Mozambique map of travel time to the nearest healthcare facility for adults aged ≥ 60 years 85
Figure S80. Namibia map of travel time to the nearest healthcare facility for adults aged ≥ 60 years 86
Figure S81. Niger map of travel time to the nearest healthcare facility for adults aged ≥ 60 years 87
Figure S82. Nigeria map of travel time to the nearest healthcare facility for adults aged ≥ 60 years 88
Figure S83. Republic of the Congo map of travel time to the nearest healthcare facility for adults aged ≥ 60 years 89
Figure S84. Rwanda map of travel time to the nearest healthcare facility for adults aged ≥ 60 years 90
Figure S85. Senegal map of travel time to the nearest healthcare facility for adults aged ≥ 60 years 91
Figure S86. Sierra Leone map of travel time to the nearest healthcare facility for adults aged ≥ 60 years 92
Figure S87. Somalia map of travel time to the nearest healthcare facility for adults aged ≥ 60 years 93
Figure S88. South Africa map of travel time to the nearest healthcare facility for adults aged ≥ 60 years 94
Figure S89. South Sudan map of travel time to the nearest healthcare facility for adults aged ≥ 60 years 95
Figure S90. Sudan map of travel time to the nearest healthcare facility for adults aged ≥ 60 years 96
Figure S91. Tanzania map of travel time to the nearest healthcare facility for adults aged ≥ 60 years 97
Figure S92. The Gambia map of travel time to the nearest healthcare facility for adults aged ≥ 60 years 98
Figure S93. Togo map of travel time to the nearest healthcare facility for adults aged ≥ 60 years 99
Figure S94. Uganda map of travel time to the nearest healthcare facility for adults aged ≥ 60 years 100

Figure S95. Zambia map of travel time to the nearest healthcare facility for adults aged ≥ 60 years 101

Figure S96. Zimbabwe map of travel time to the nearest healthcare facility for adults aged ≥ 60 years 102

Figure S97. Maps of travel time to the nearest healthcare facility for adults ≥ 60 years, by region based on the MFL dataset 103

Figure S98. Maps of travel time to the nearest healthcare facility for adults ≥ 60 years, by region based on the OSM dataset 104

Figure S1. Distribution of travel time to the nearest hospital, by country

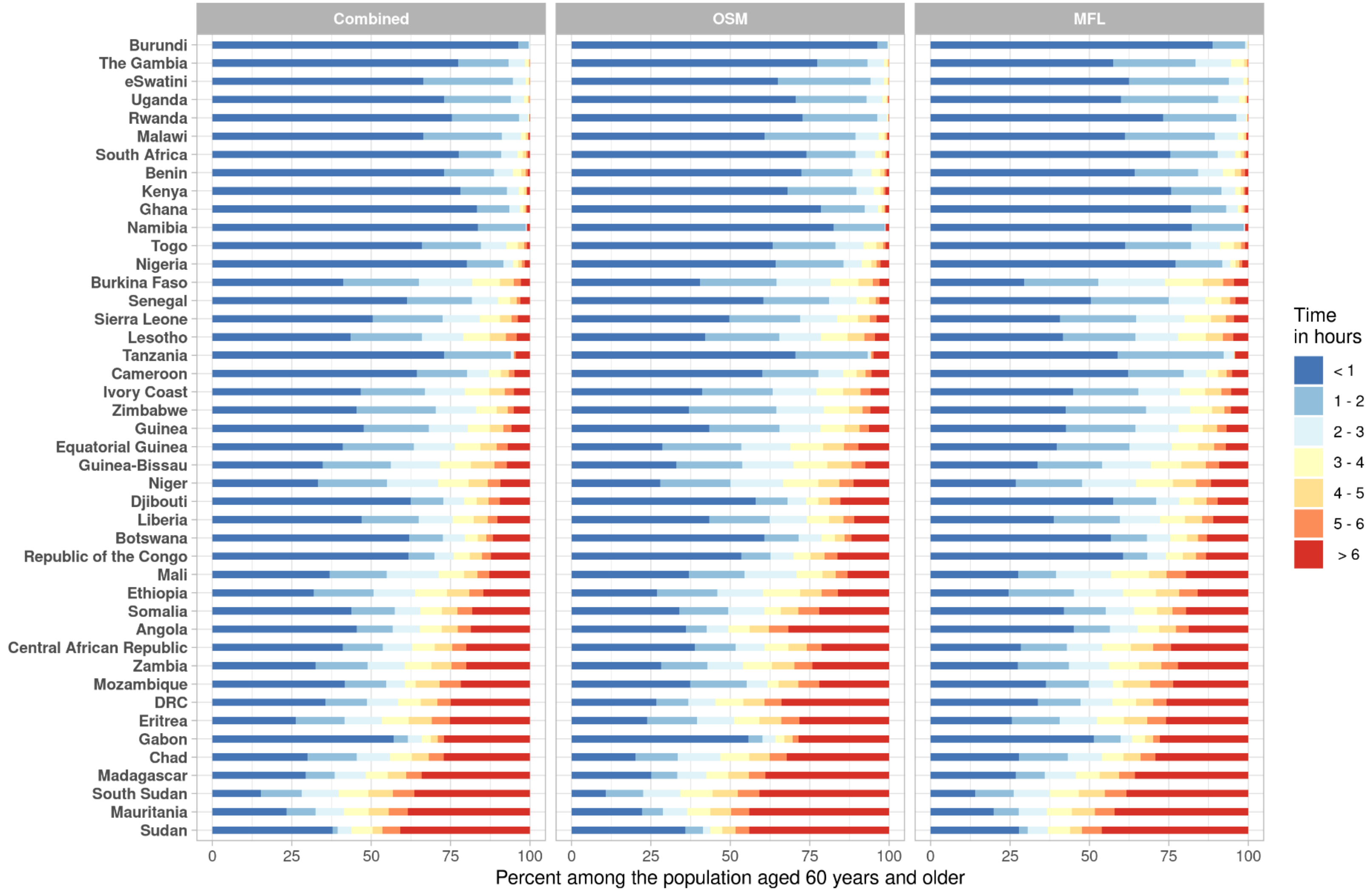

Abbreviations: DRC=Democratic Republic of the Congo
"Combined" refers to the travel time to the nearest hospital regardless of whether the hospital was recorded in the MFL or OSM data.

Figure S2. Distribution of travel time to the nearest healthcare facility of any type, by country

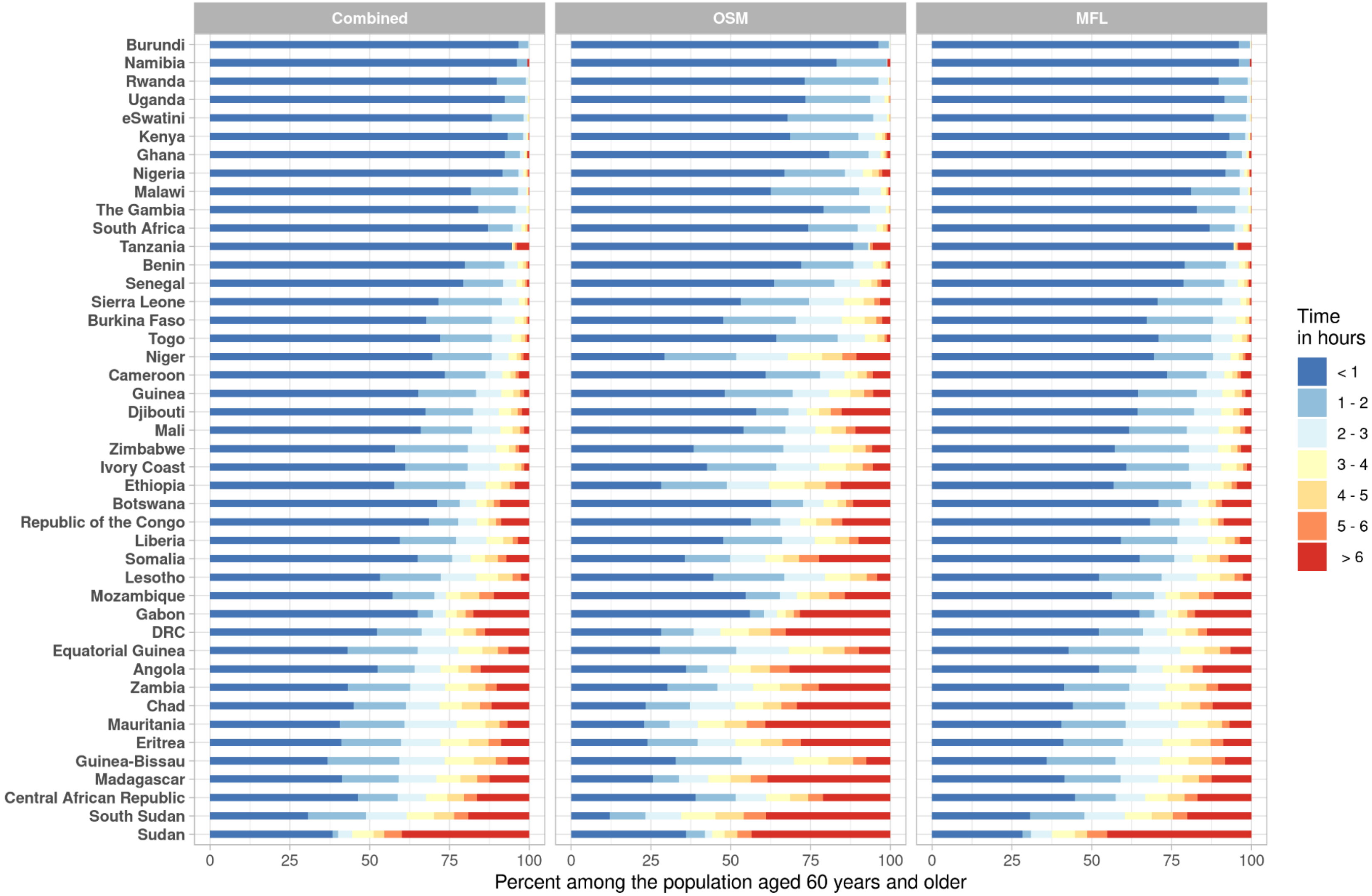

Abbreviations: DRC=Democratic Republic of the Congo

"Combined" refers to the travel time to the nearest healthcare facility regardless of whether the facility was recorded in the MFL or OSM data.

Figure S3. Histogram of travel time to the nearest hospital in the MFL dataset for adults aged 60 years and older, by country[1]

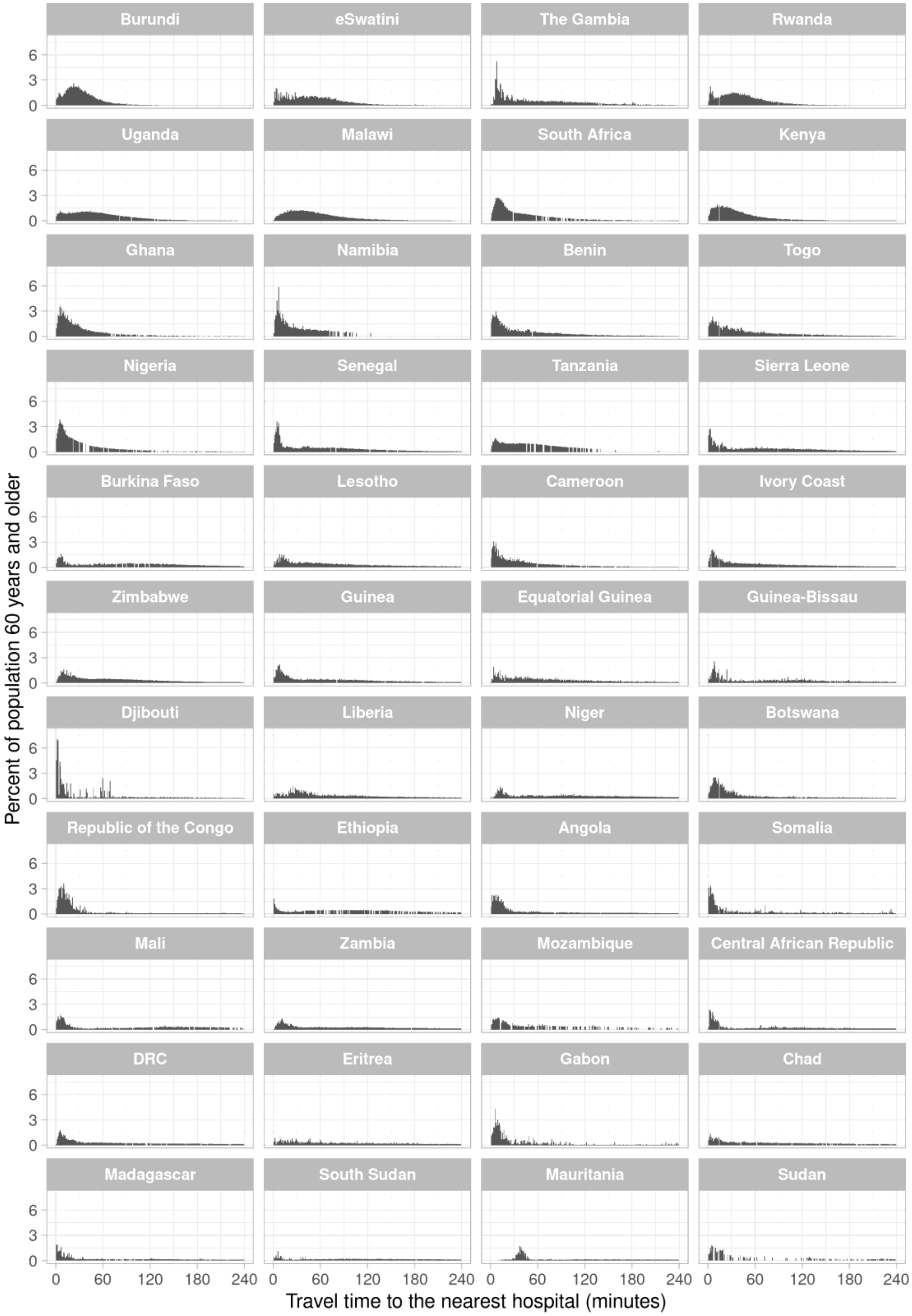

Abbreviations: DRC=Democratic Republic of the Congo

[1] Countries were ordered in ascending order by the proportion of adults aged 60 years and older in their population who reside in a 1km x 1km area that has an estimated travel time >2 hours to the nearest hospital.

Figure S4. Histogram of travel time to the nearest hospital in the OSM dataset for adults aged 60 years and older, by country[1]

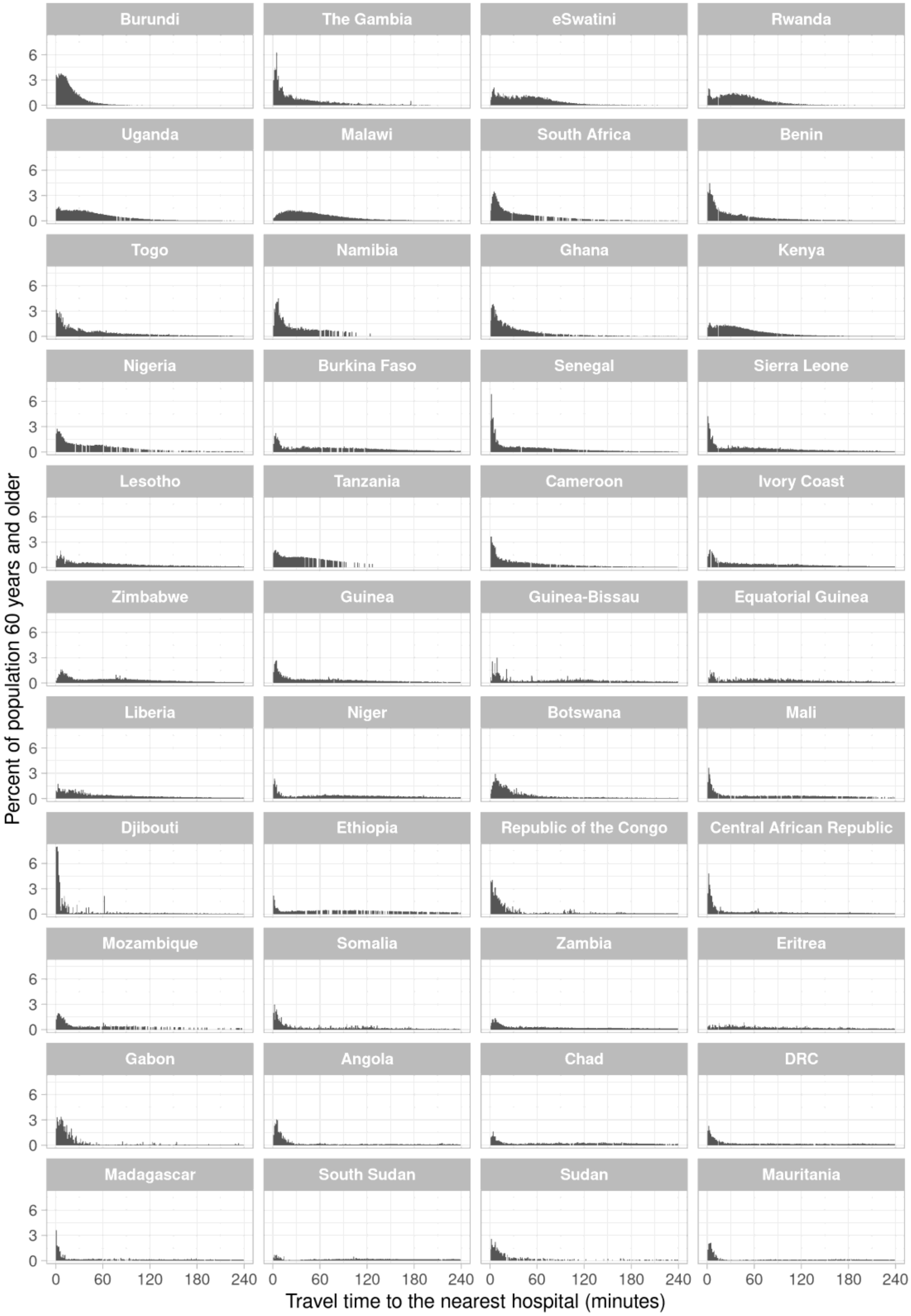

Abbreviations: DRC=Democratic Republic of the Congo

[1] Countries were ordered in ascending order by the proportion of adults aged 60 years and older in their population who reside in a 1km x 1km area that has an estimated travel time >2 hours to the nearest hospital.

Figure S5. Histogram of travel time to the nearest healthcare facility (of any type) in the MFL dataset for adults aged 60 years and older, by country[1]

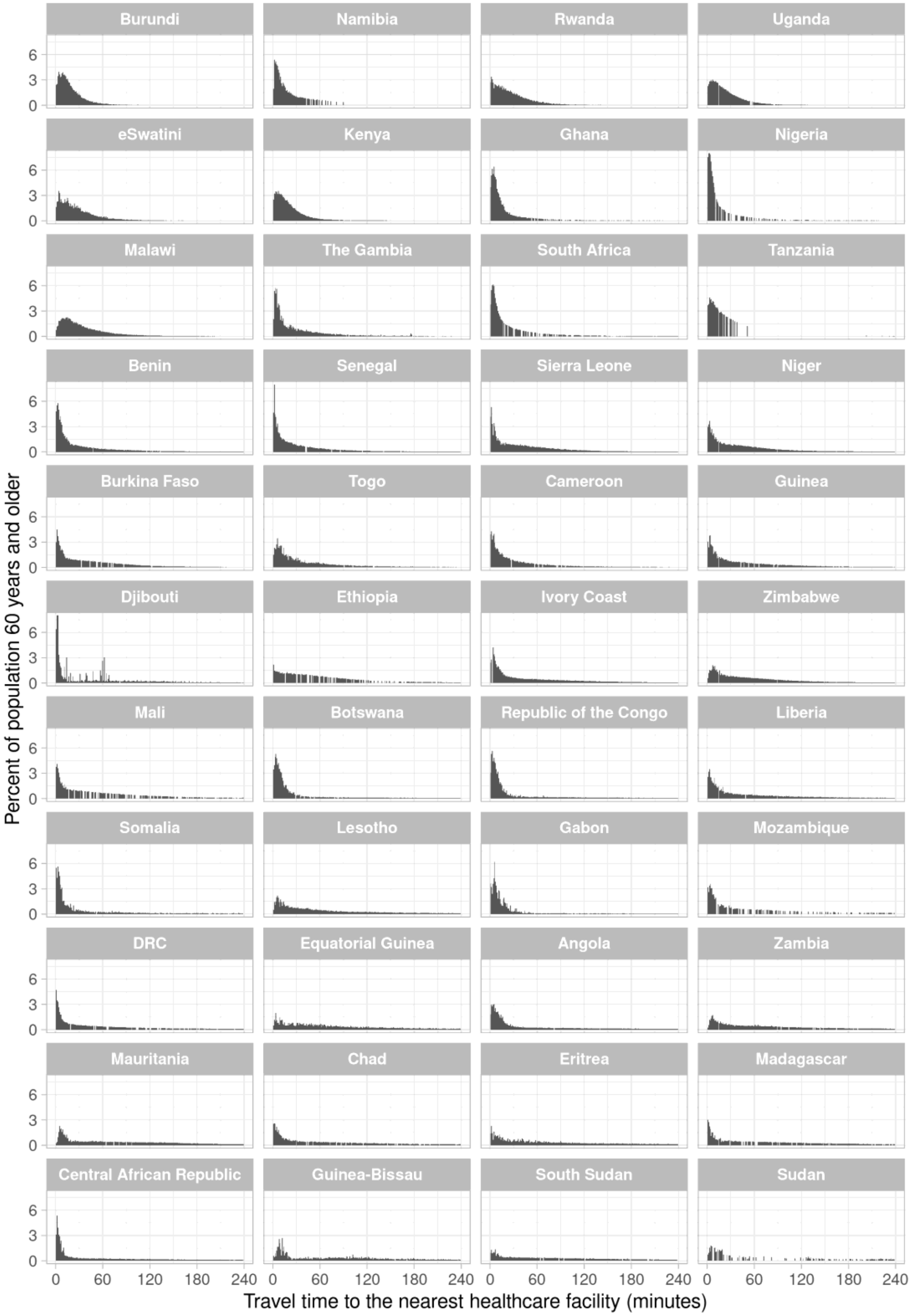

Abbreviations: DRC=Democratic Republic of the Congo

[1] Countries were ordered in ascending order by the proportion of adults aged 60 years and older in their population who reside in a 1km x 1km area that has an estimated travel time >2 hours to the nearest healthcare facility.

Figure S6. Histogram of travel time to the nearest healthcare facility (of any type) in the OSM dataset for adults aged 60 years and older, by country[1]

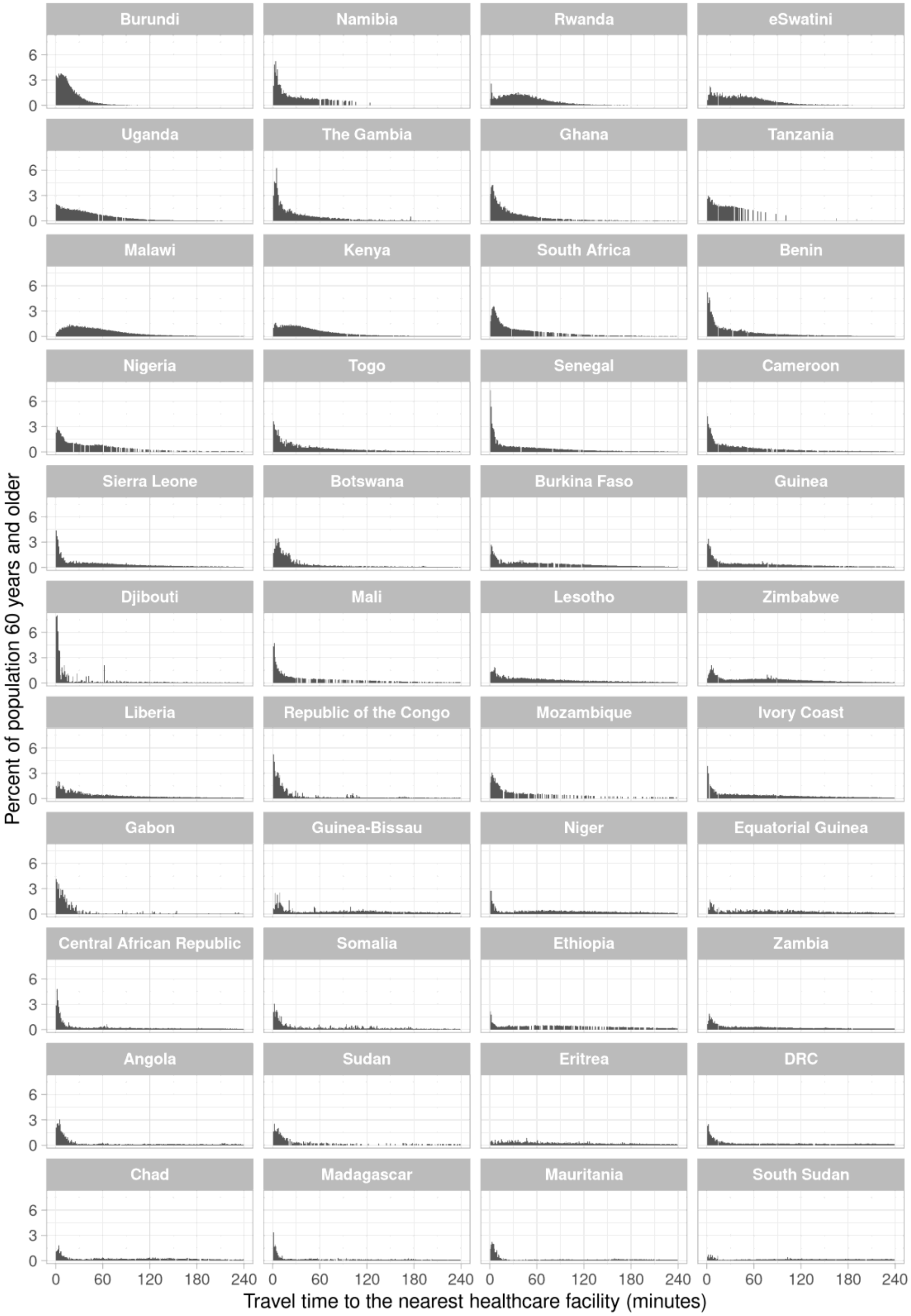

Abbreviations: DRC=Democratic Republic of the Congo

[1] Countries were ordered in ascending order by the proportion of adults aged 60 years and older in their population who reside in a 1km x 1km area that has an estimated travel time >2 hours to the nearest healthcare facility.

Figure S7. Angola map of travel time to the nearest hospital for adults aged ≥ 60 years

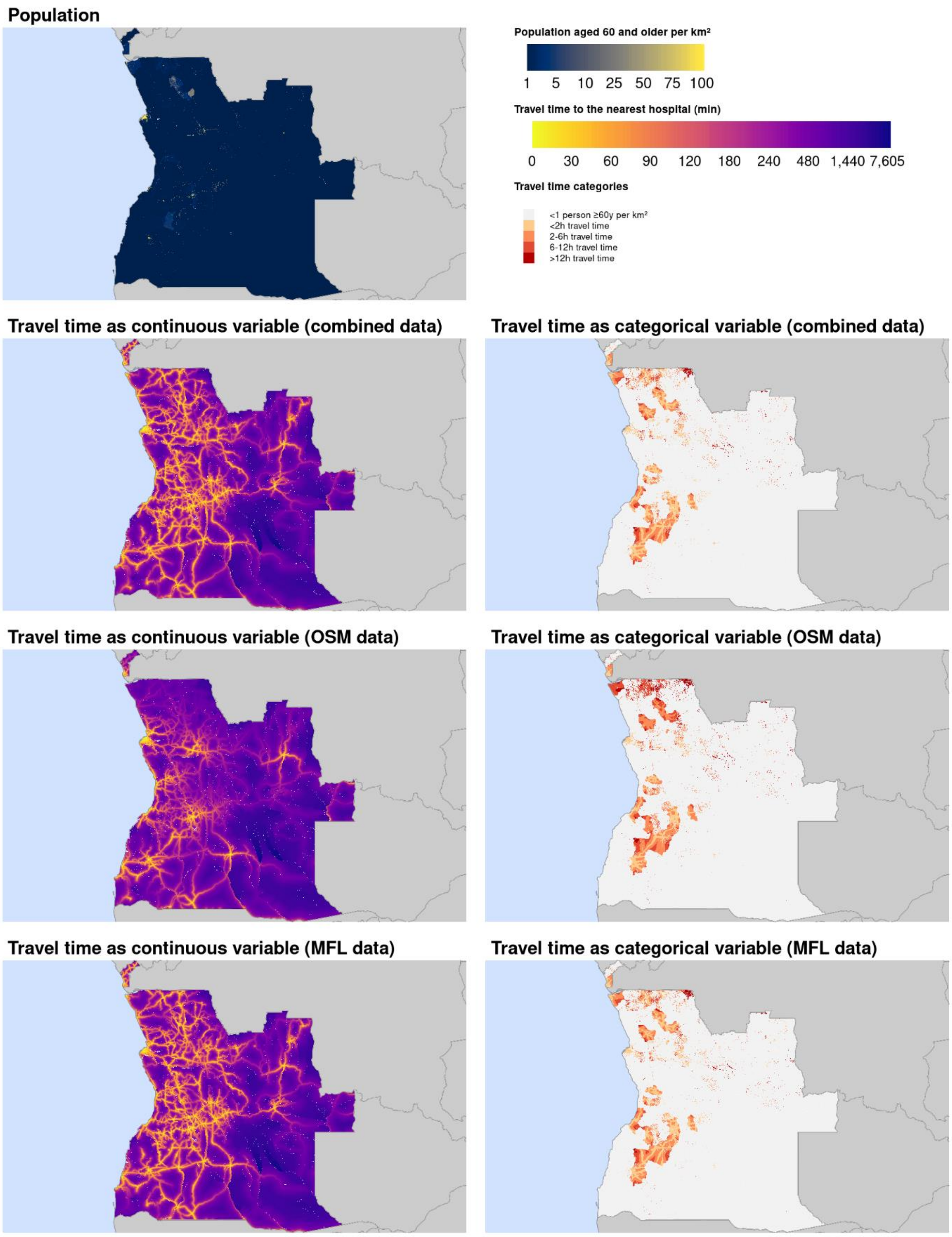

Figure S8. Benin map of travel time to the nearest hospital for adults aged ≥ 60 years

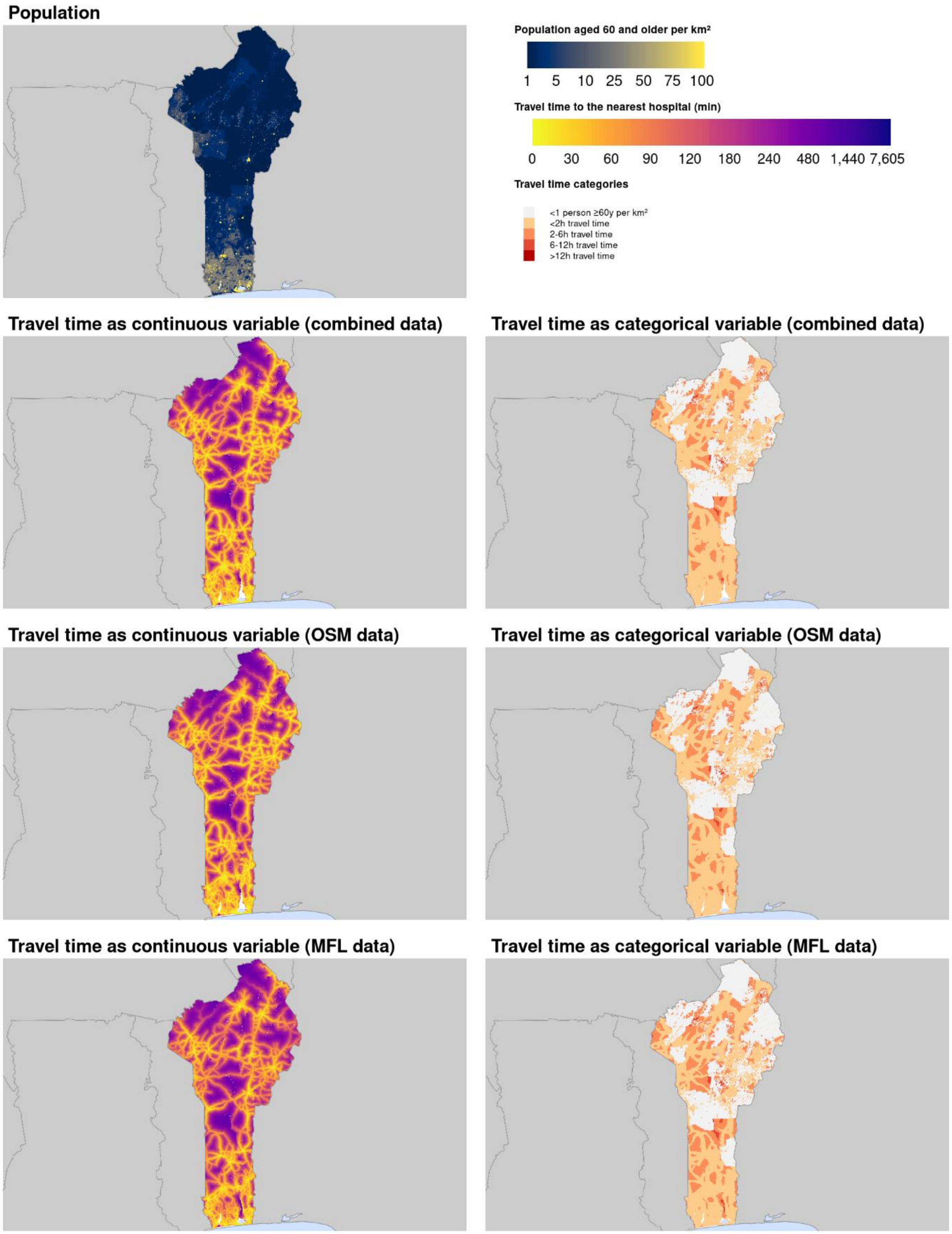

Figure S9. Botswana map of travel time to the nearest hospital for adults aged ≥ 60 years

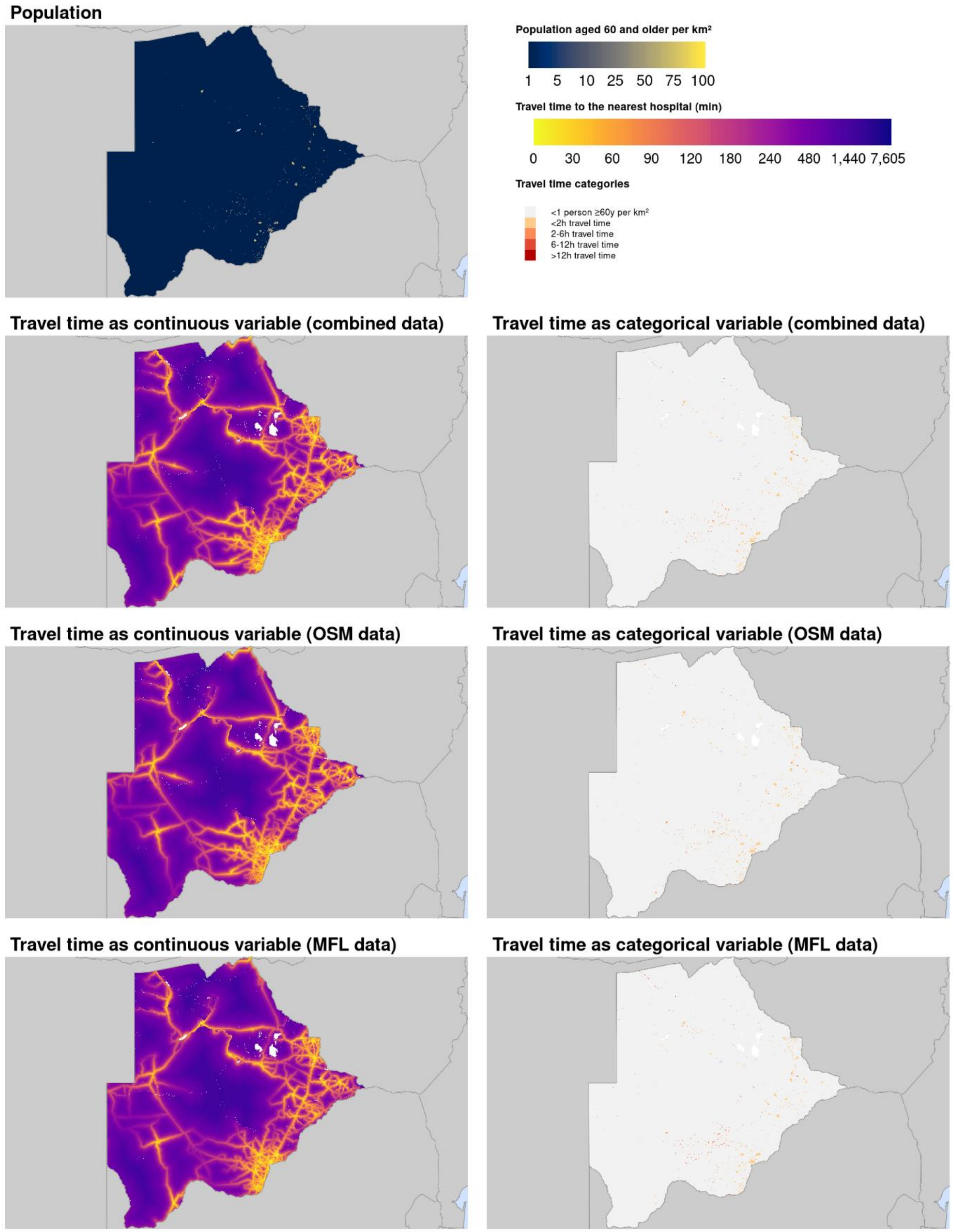

Figure S10. Burkina Faso map of travel time to the nearest hospital for adults aged ≥ 60 years

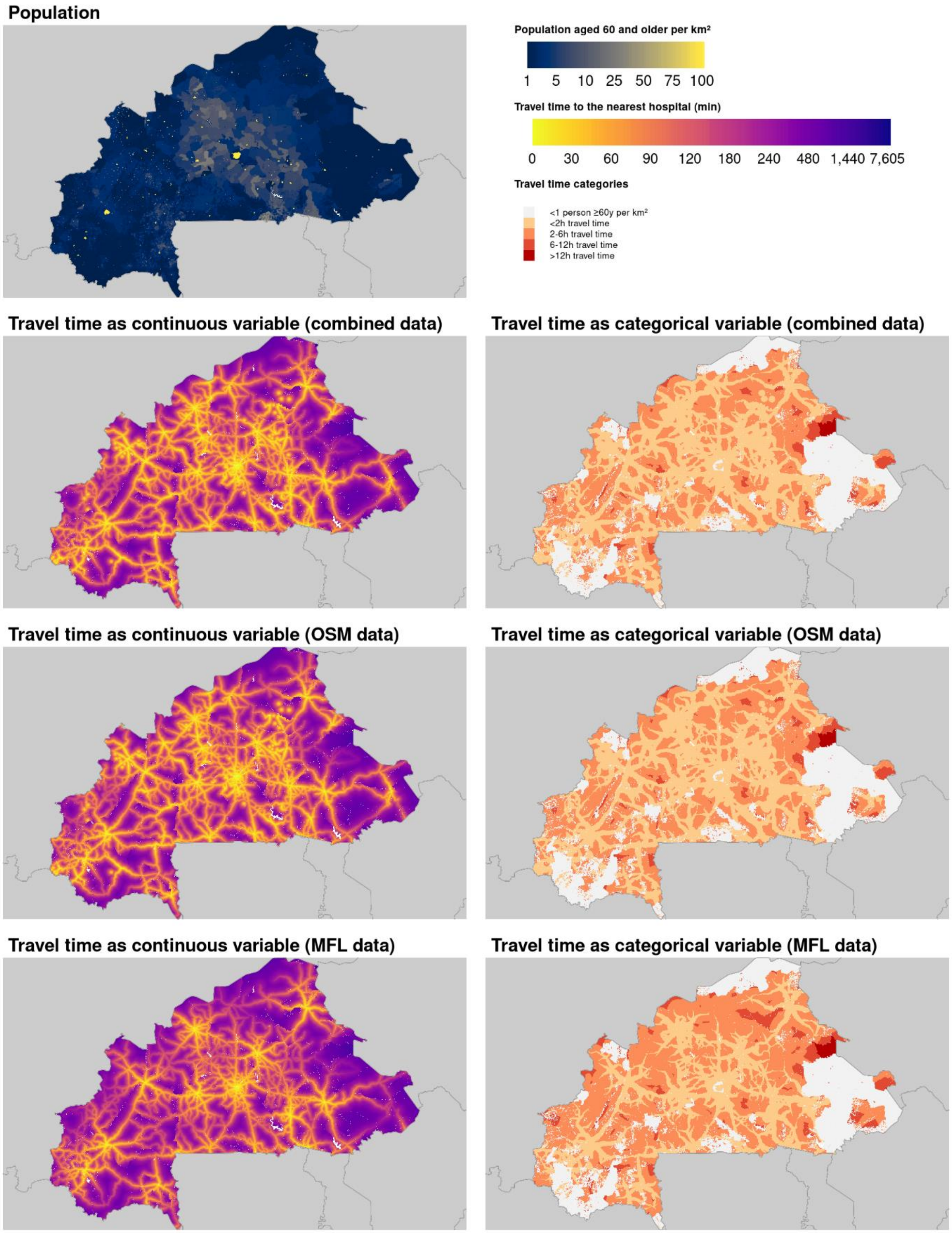

Figure S11. Burundi map of travel time to the nearest hospital for adults aged ≥ 60 years

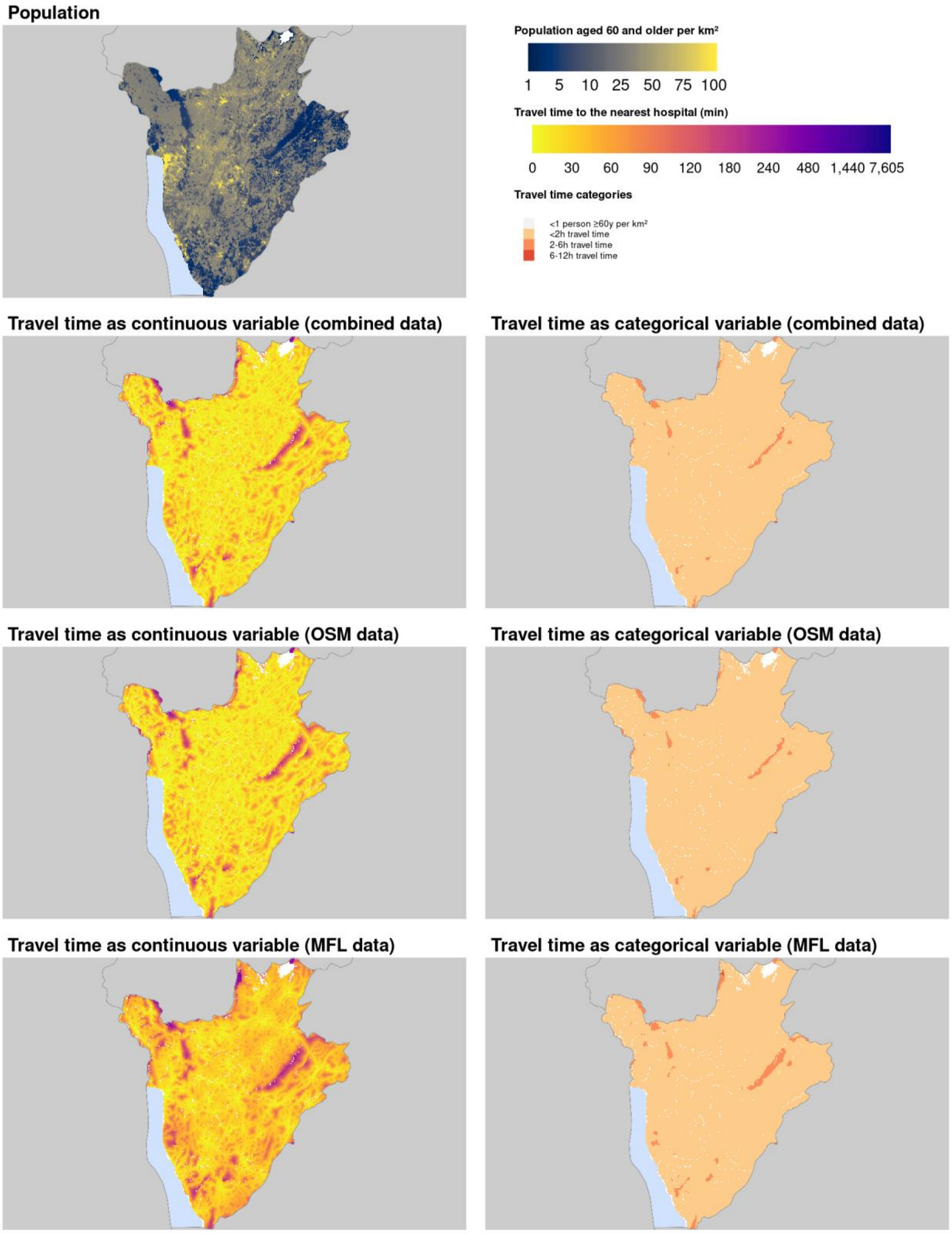

Figure S12. Cameroon map of travel time to the nearest hospital for adults aged ≥ 60 years

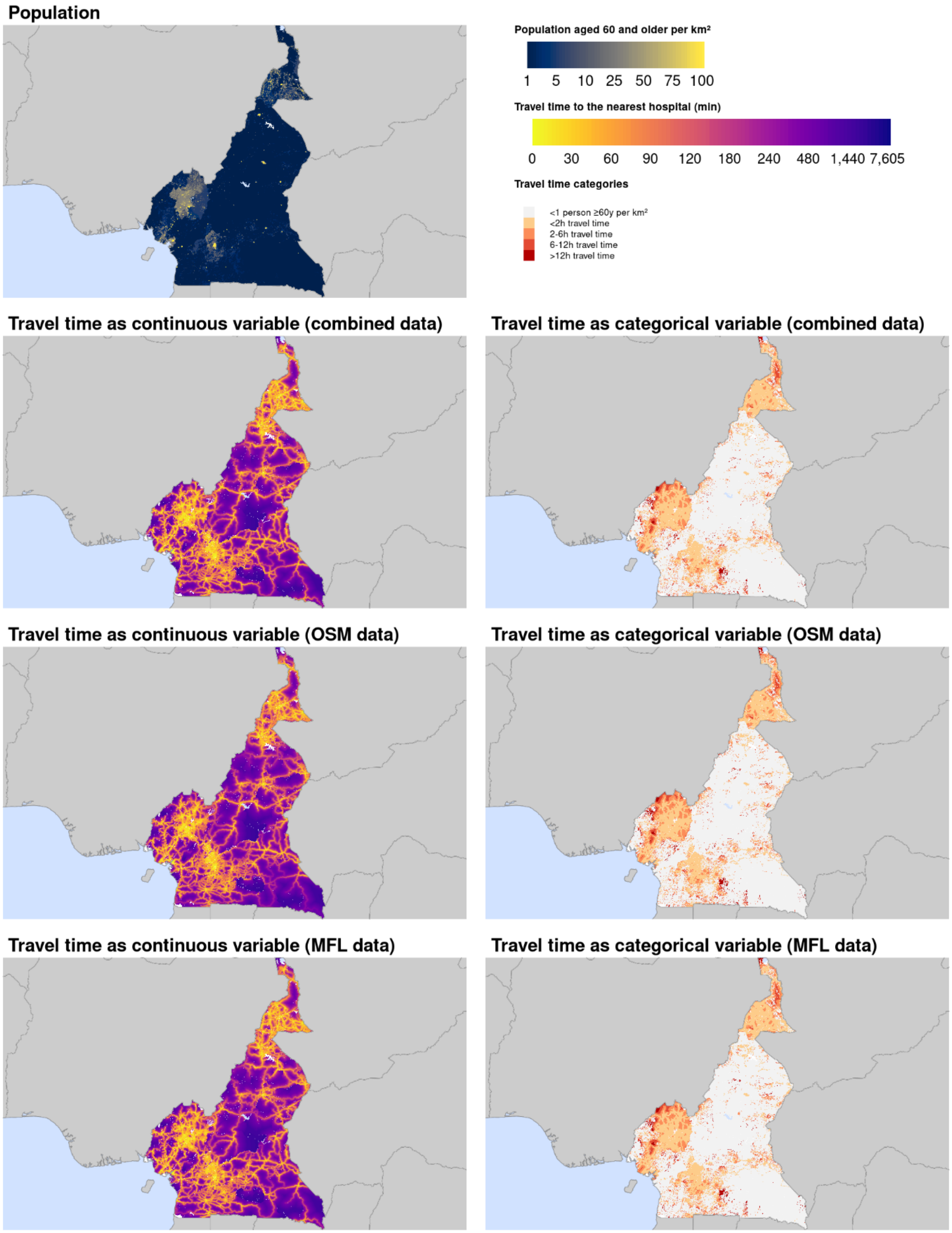

Figure S13. Central African Republic map of travel time to the nearest hospital for adults aged ≥ 60 years

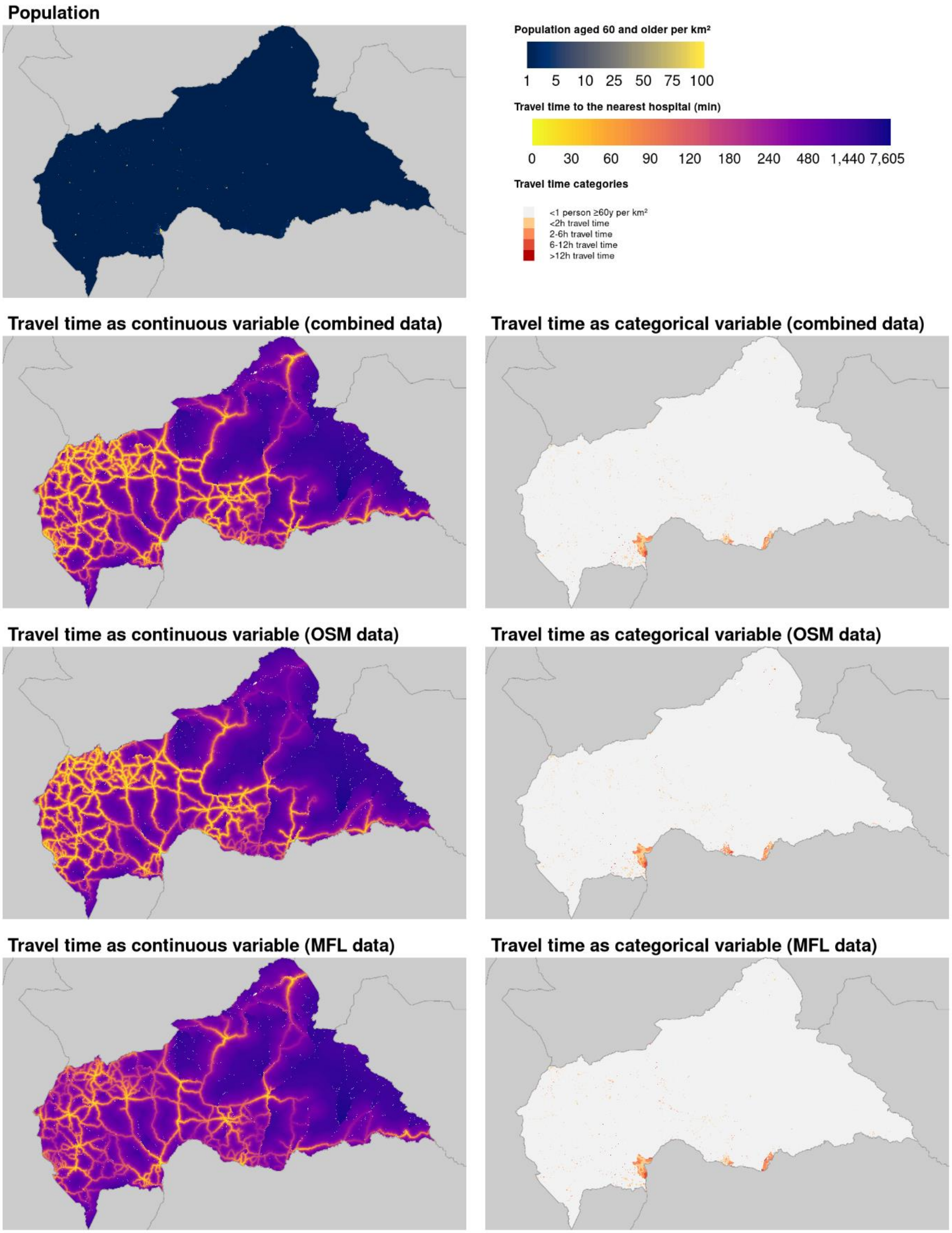

Figure S14. Chad map of travel time to the nearest hospital for adults aged ≥ 60 years

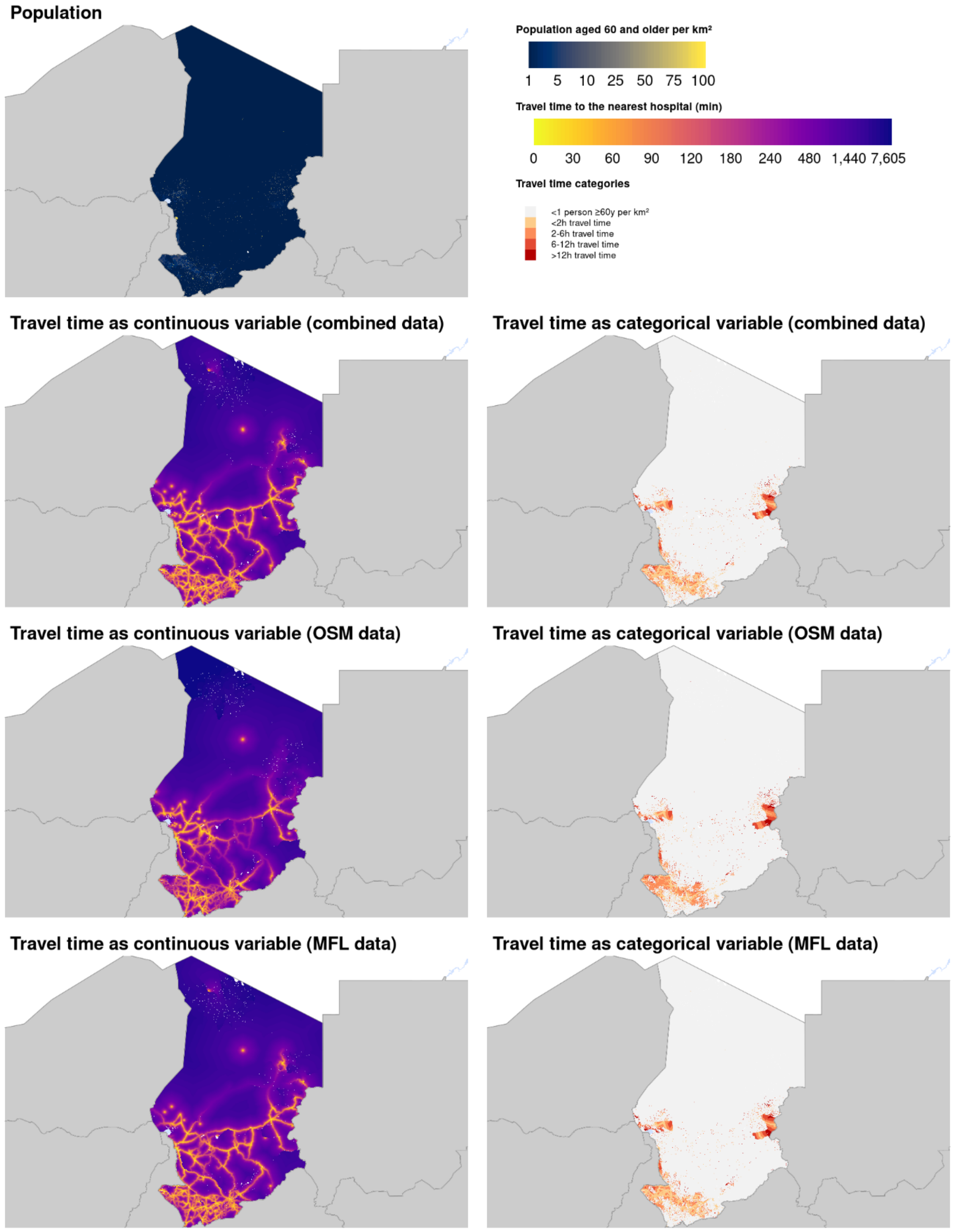

Figure S15. Djibouti map of travel time to the nearest hospital for adults aged ≥ 60 years

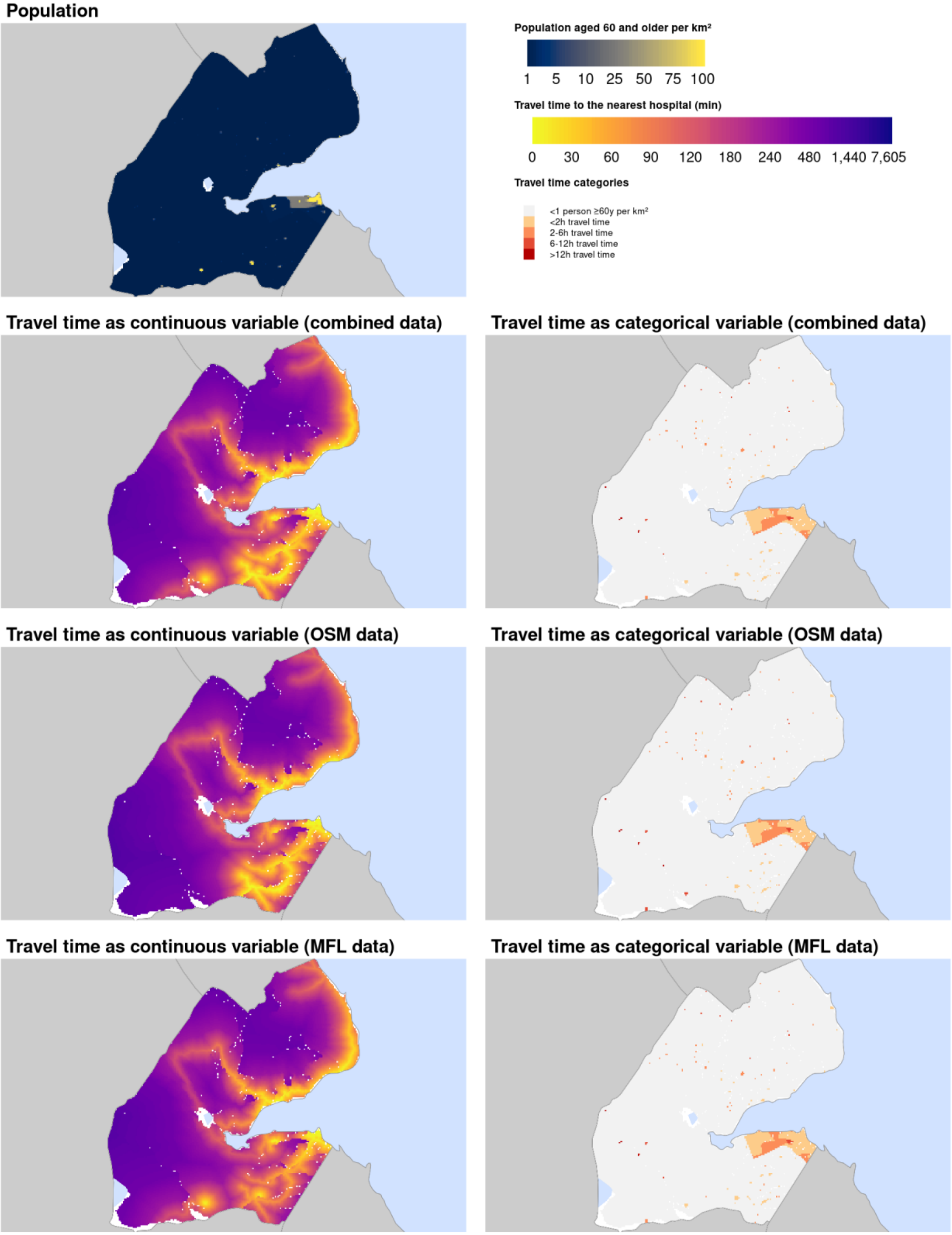

Figure S16. DRC map of travel time to the nearest hospital for adults aged ≥ 60 years

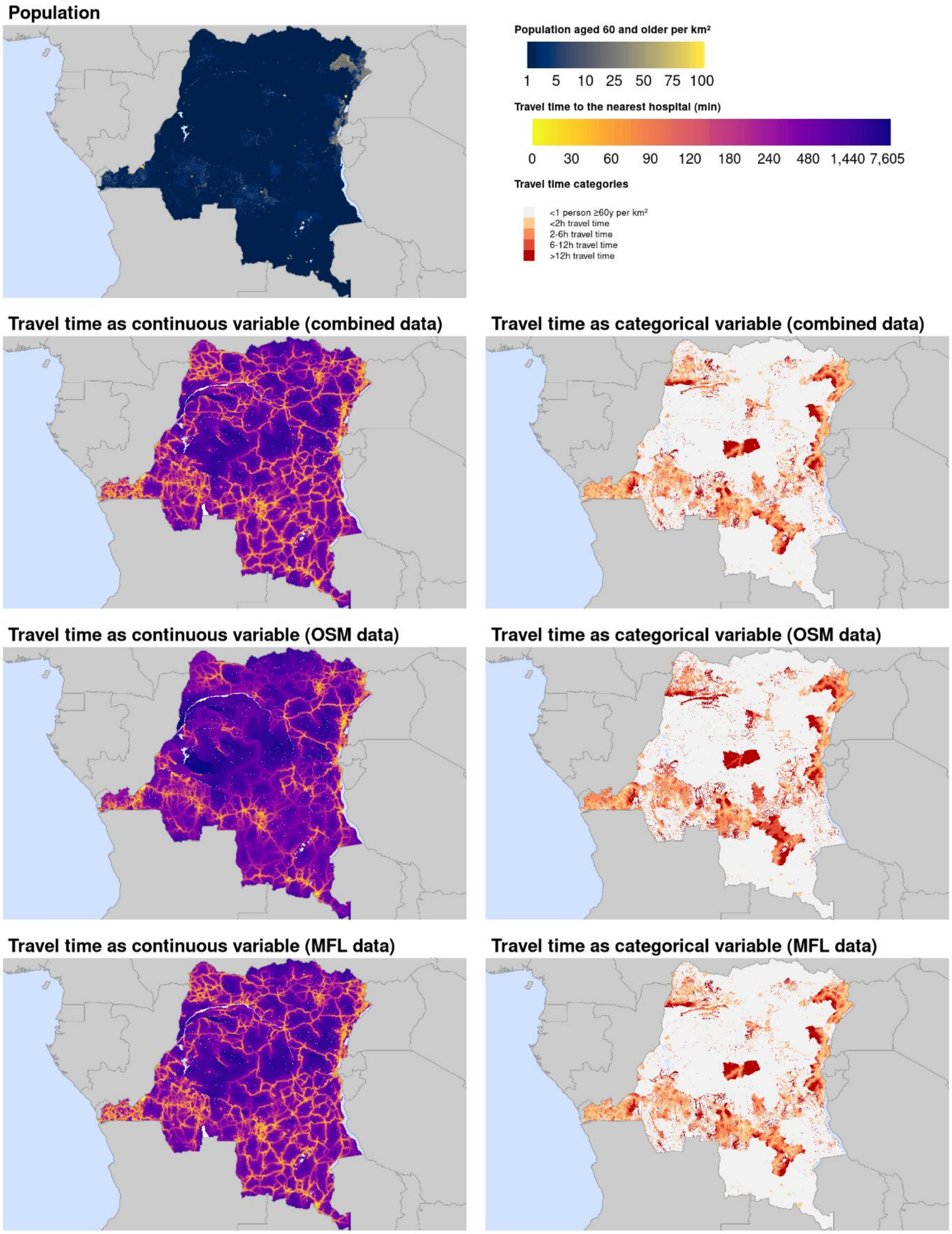

Figure S17. Equatorial Guinea map of travel time to the nearest hospital for adults aged ≥ 60 years

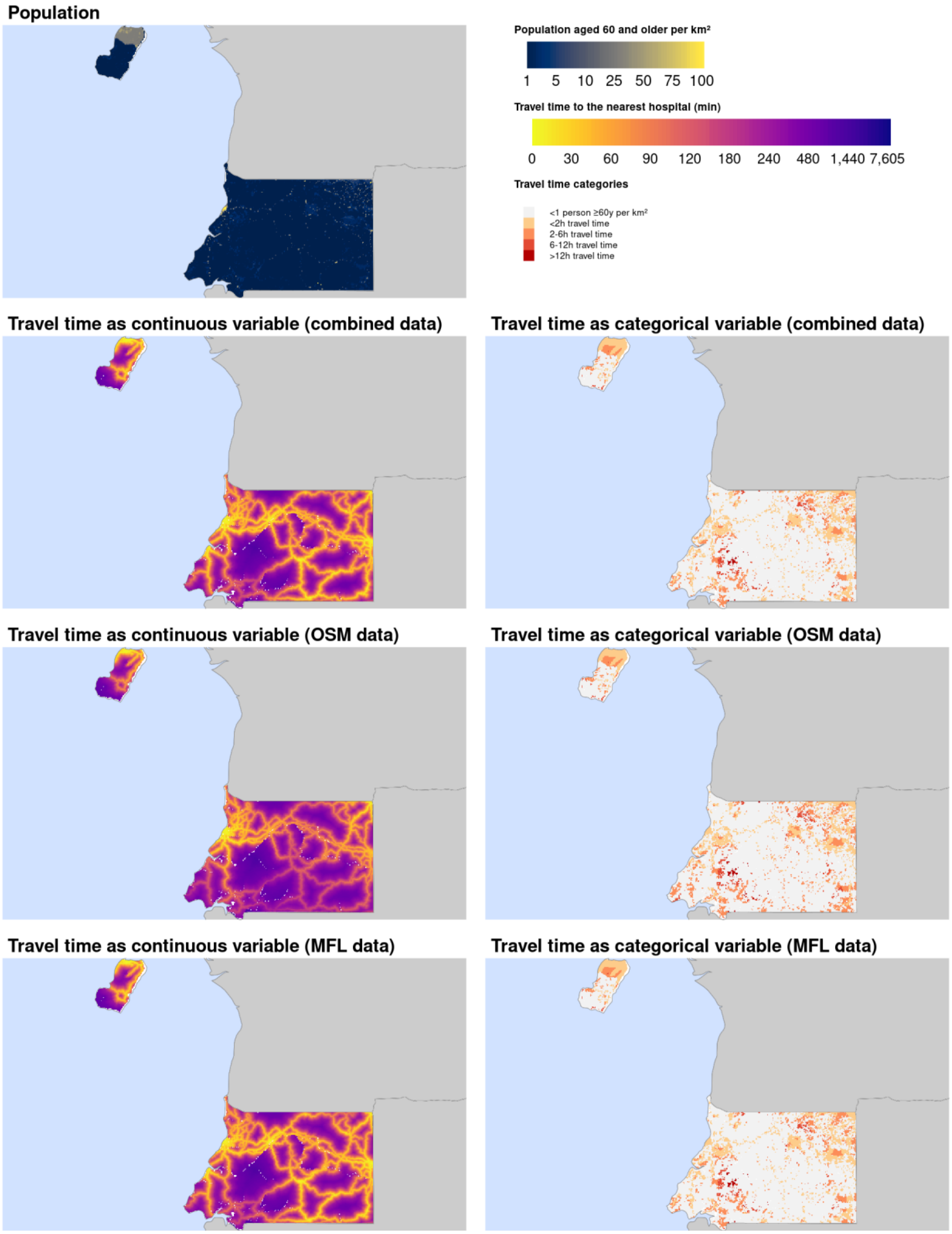

Figure S18. Eritrea map of travel time to the nearest hospital for adults aged ≥ 60 years

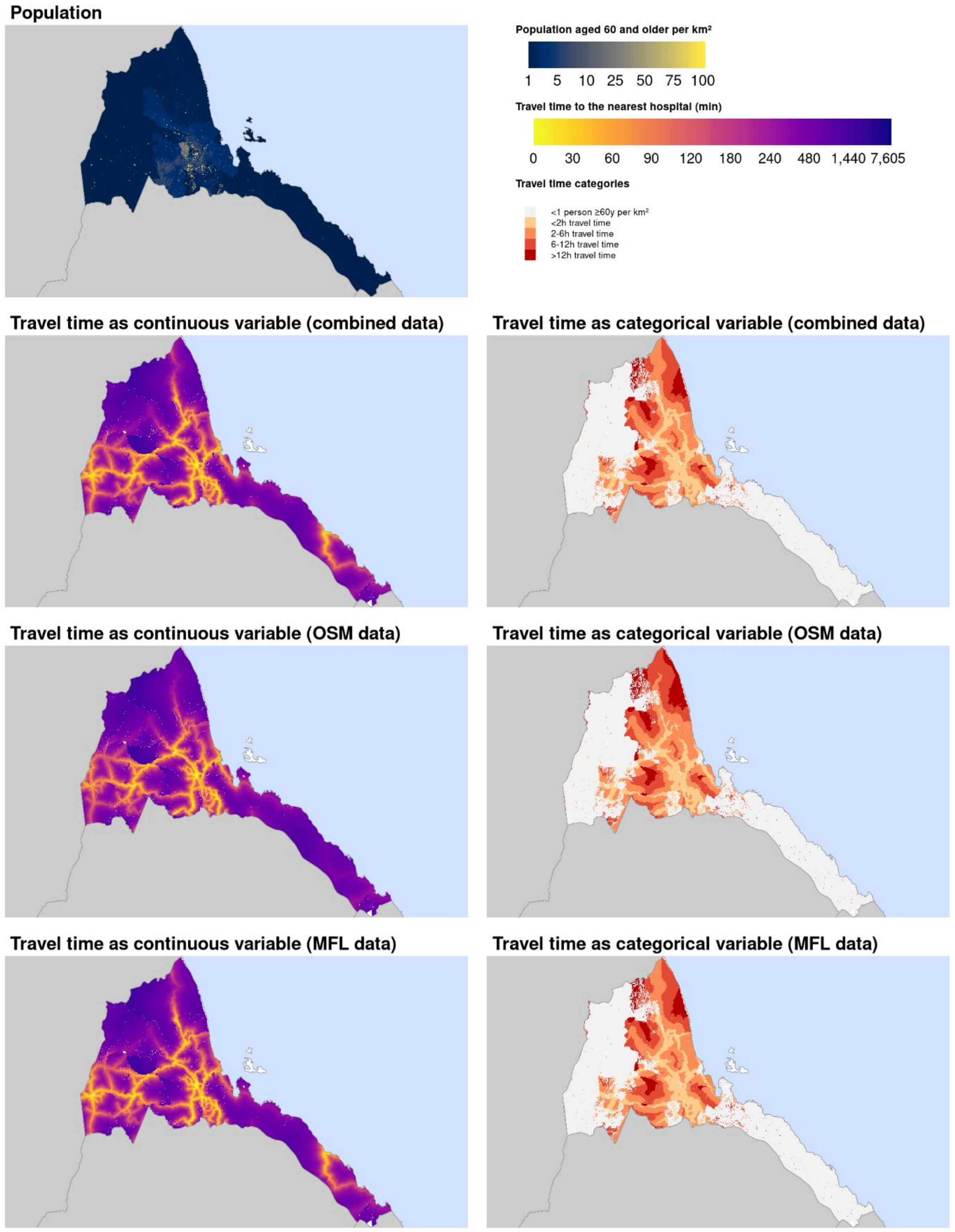

Figure S19. eSwatini map of travel time to the nearest hospital for adults aged ≥ 60 years

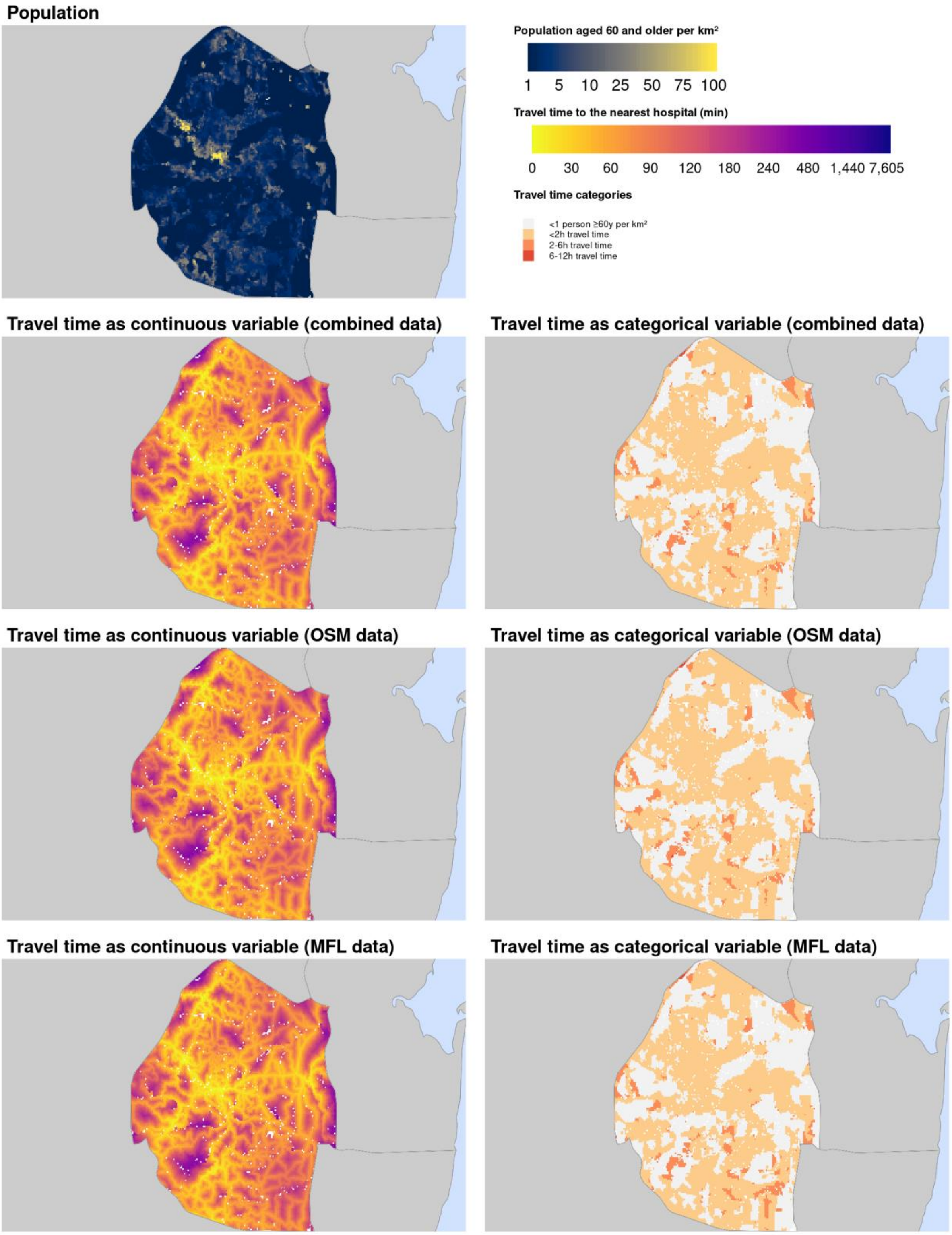

Figure S20. Ethiopia map of travel time to the nearest hospital for adults aged ≥ 60 years

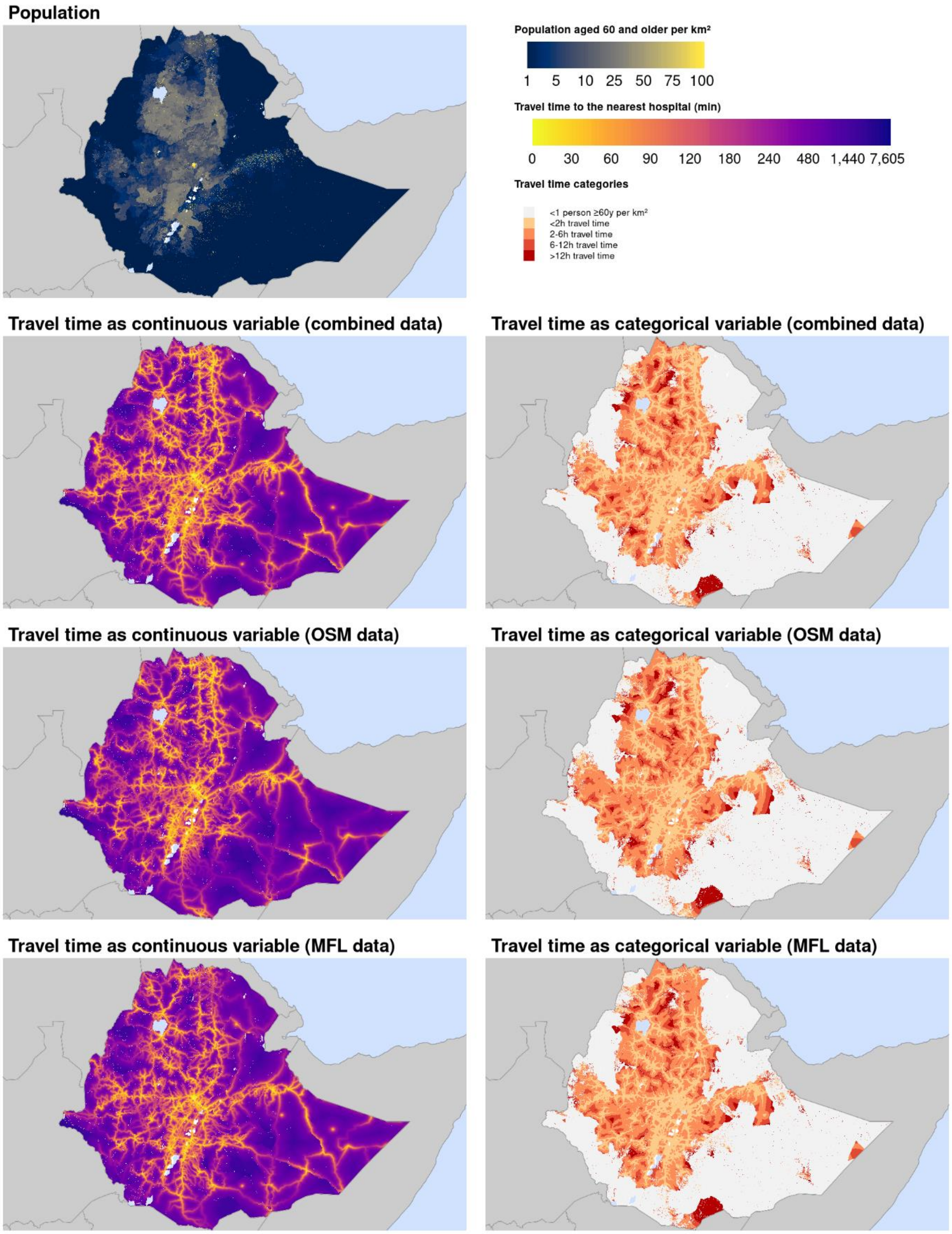

Figure S21. Gabon map of travel time to the nearest hospital for adults aged ≥ 60 years

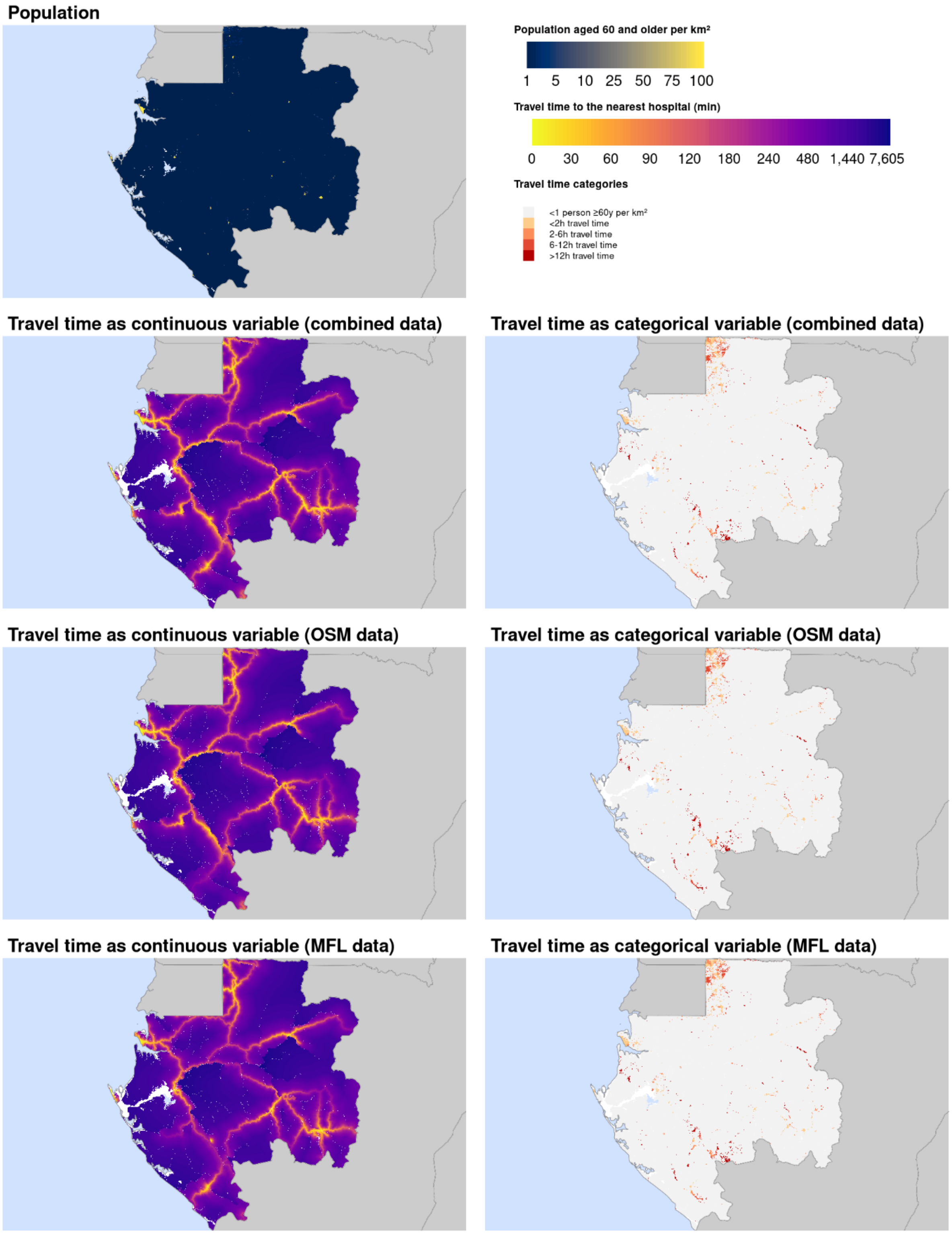

Figure S22. Ghana map of travel time to the nearest hospital for adults aged ≥ 60 years

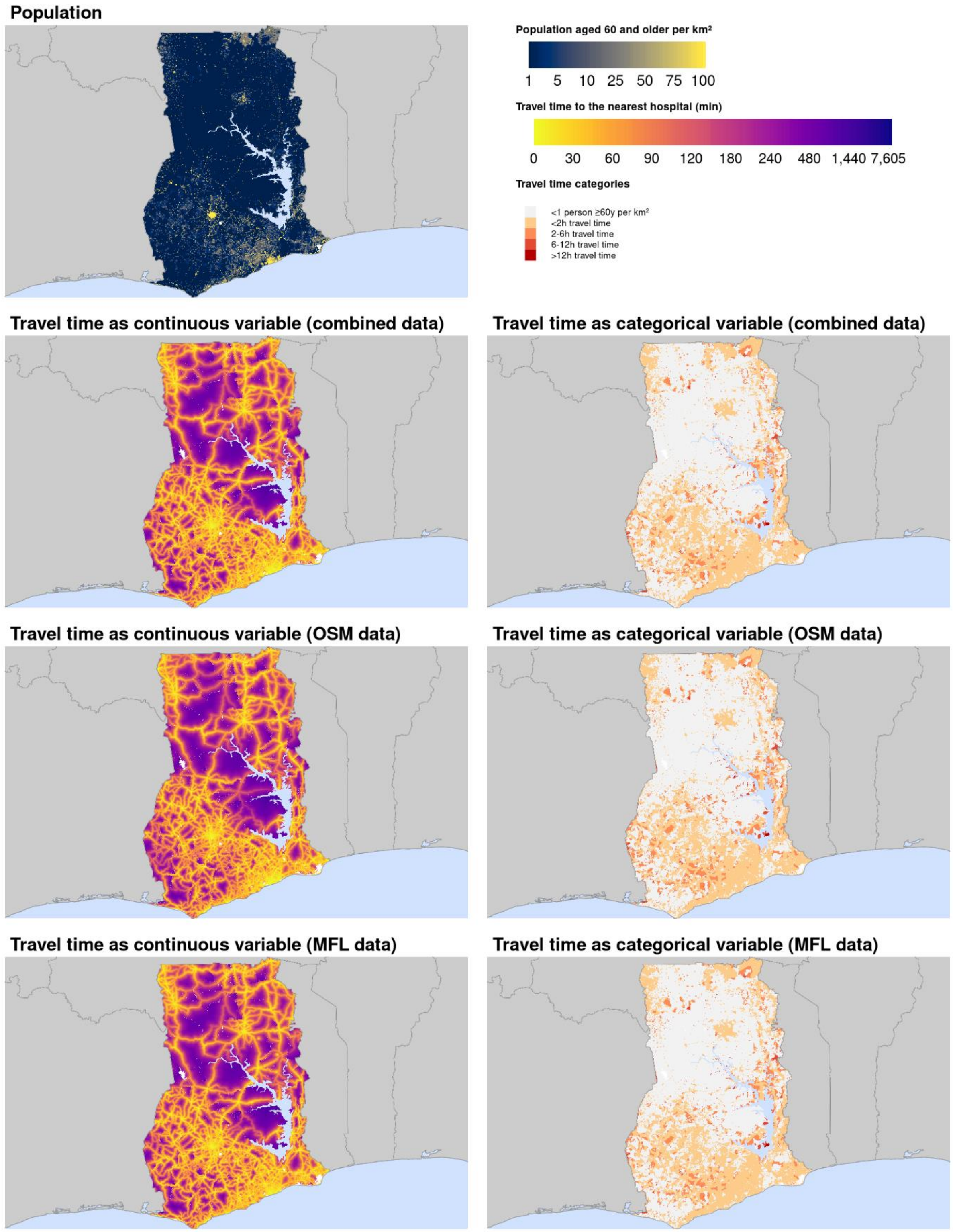

Figure S23. Guinea map of travel time to the nearest hospital for adults aged ≥ 60 years

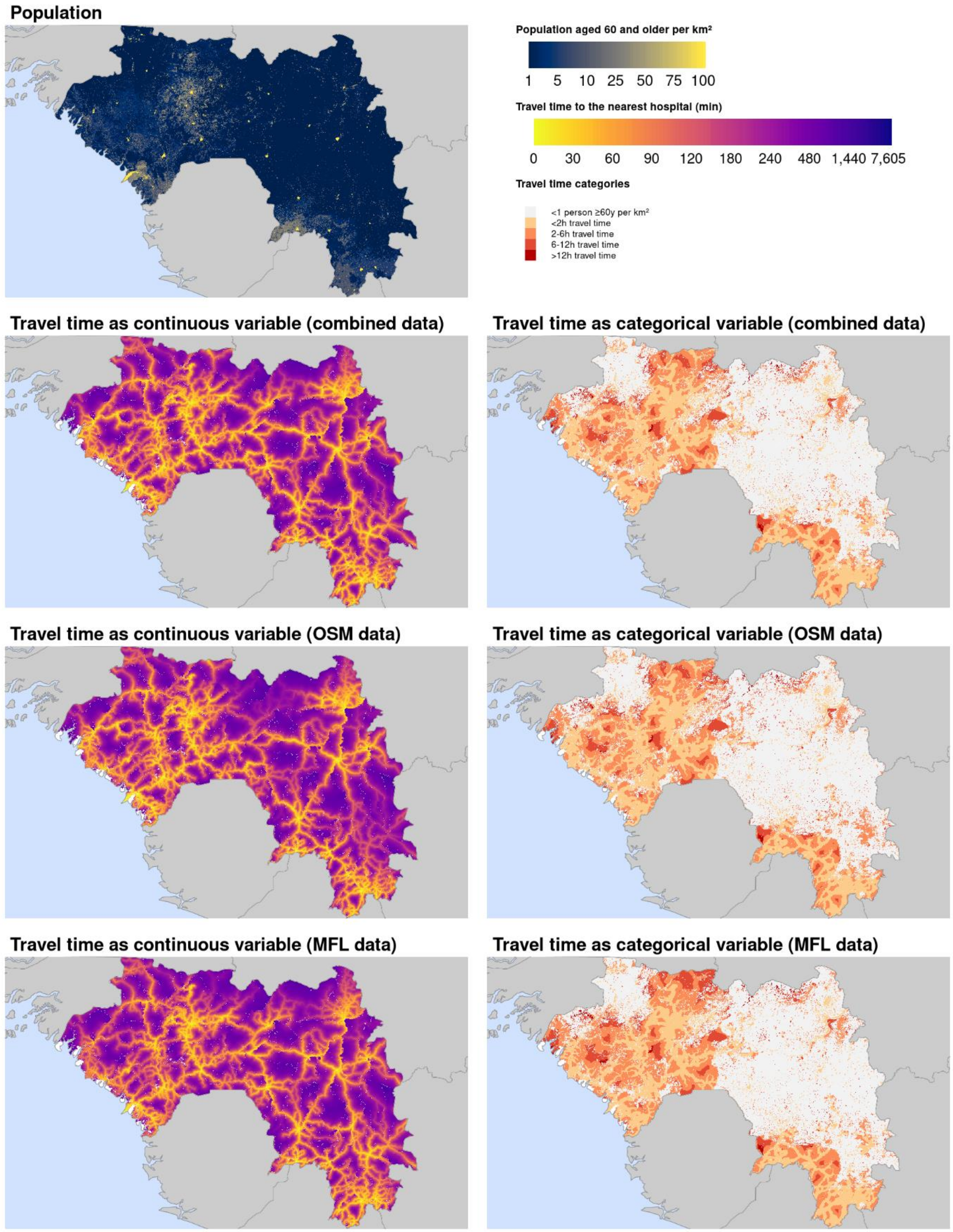

Figure S24. Guinea-Bissau map of travel time to the nearest hospital for adults aged ≥ 60 years

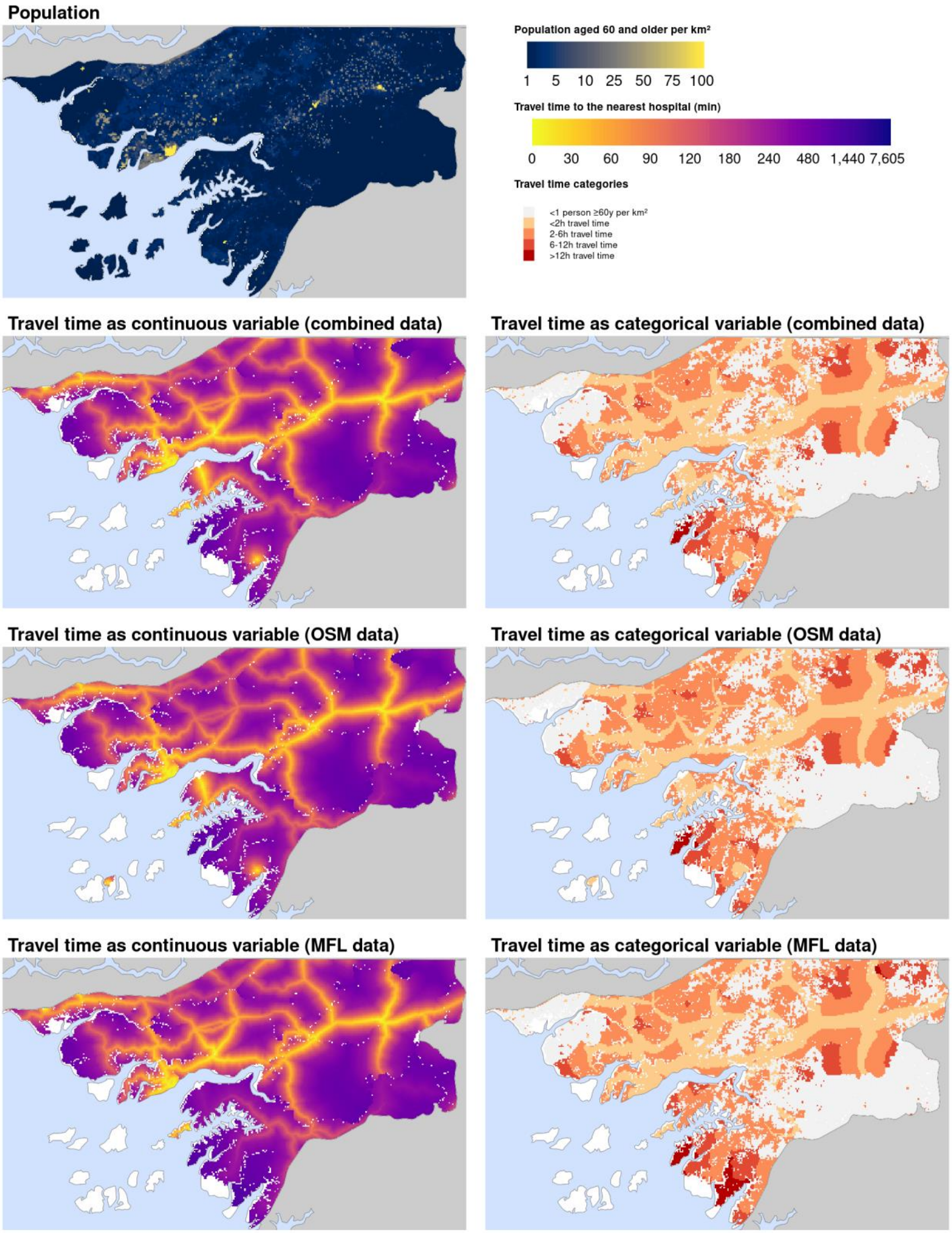

Figure S25. Ivory Coast map of travel time to the nearest hospital for adults aged ≥ 60 years

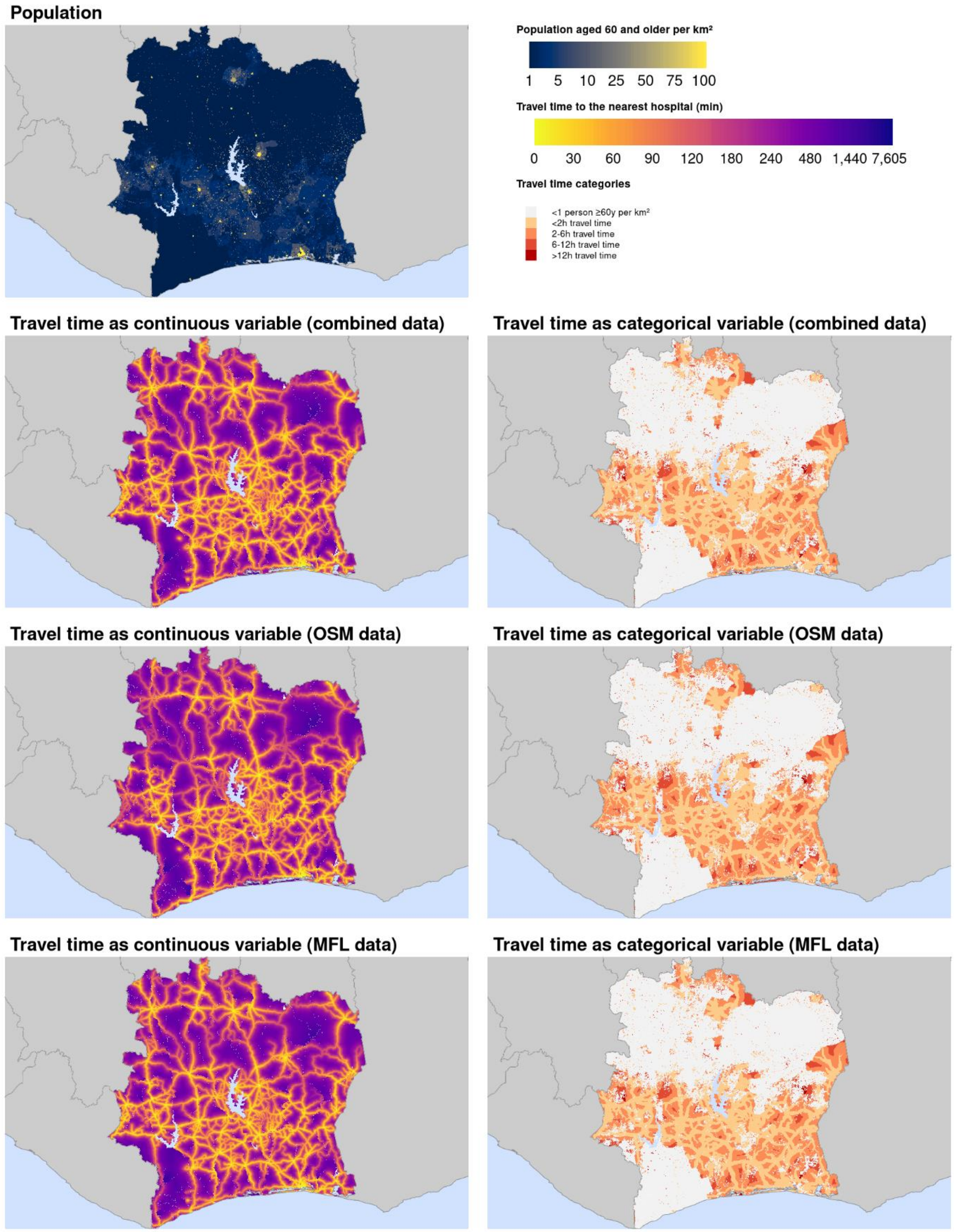

Figure S26. Kenya map of travel time to the nearest hospital for adults aged ≥ 60 years

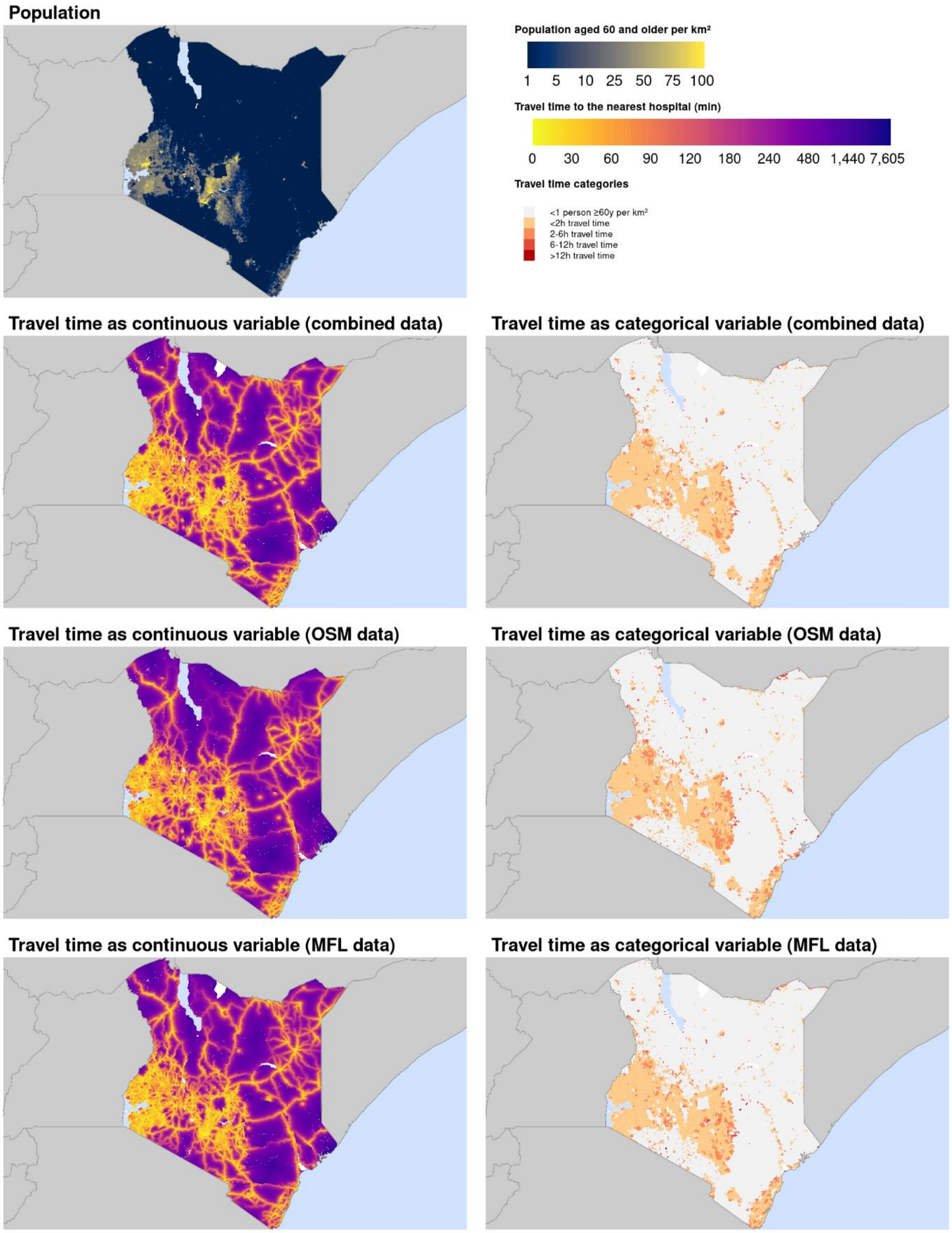

Figure S27. Lesotho map of travel time to the nearest hospital for adults aged ≥ 60 years

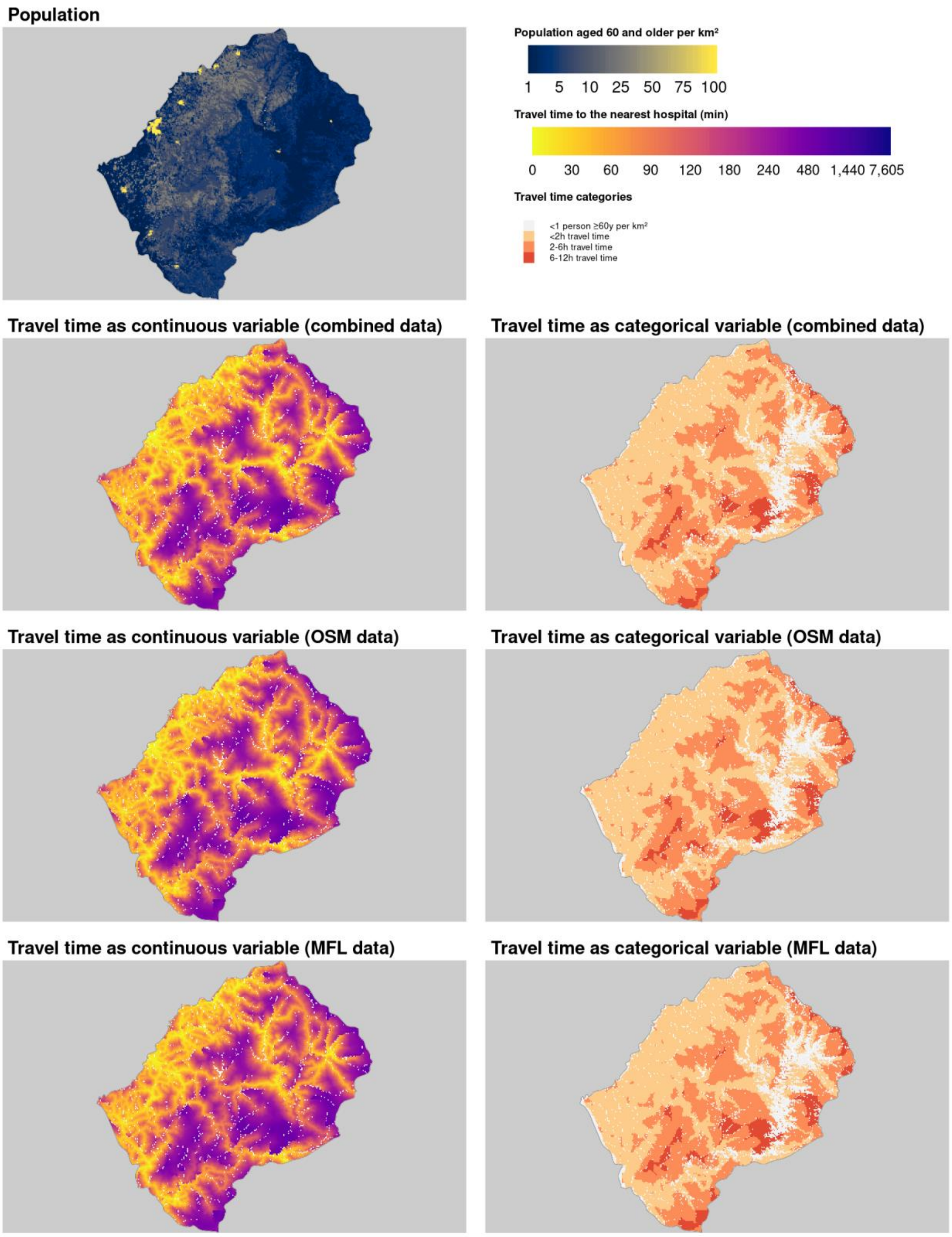

Figure S28. Liberia map of travel time to the nearest hospital for adults aged ≥ 60 years

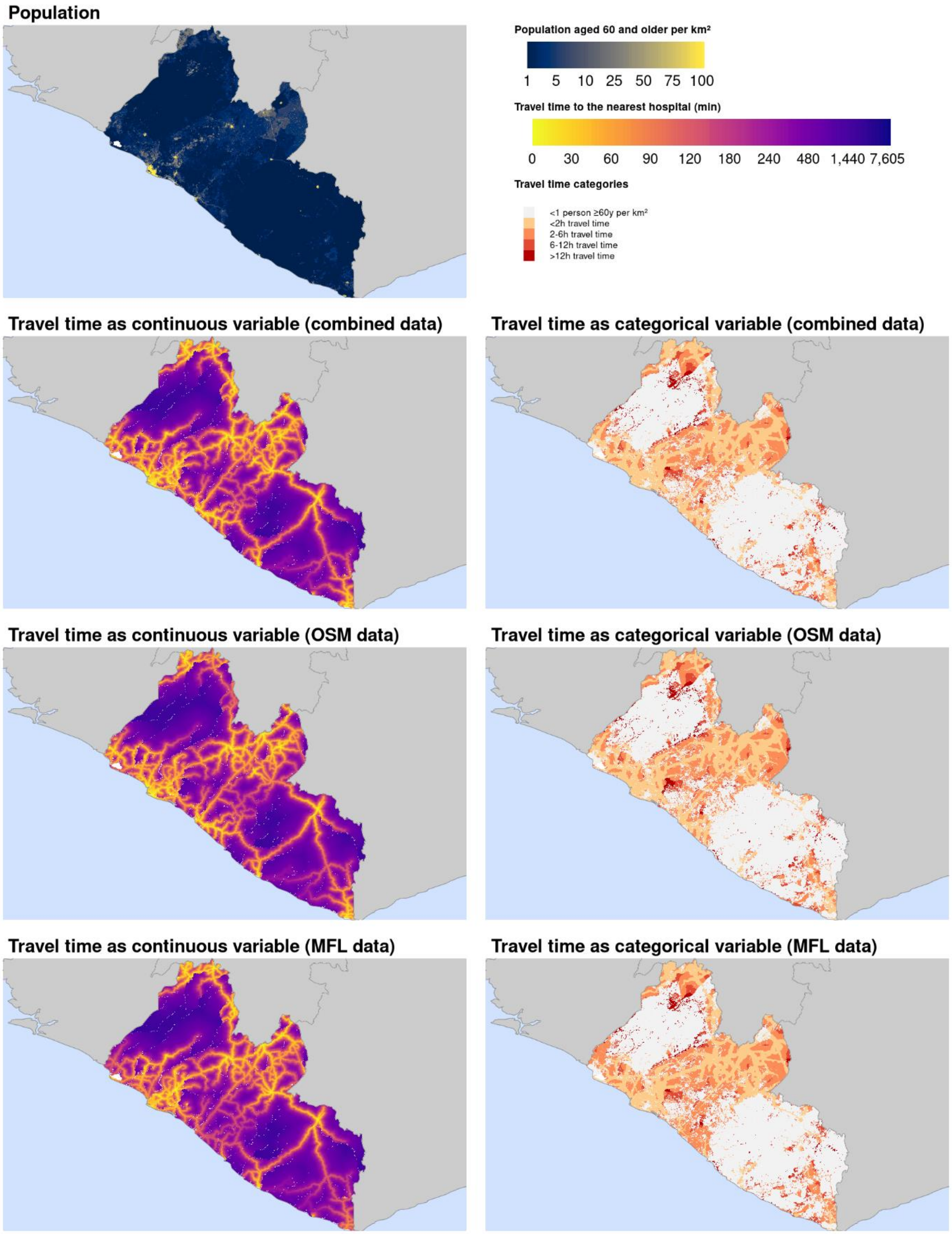

Figure S29. Madagascar map of travel time to the nearest hospital for adults aged ≥ 60 years

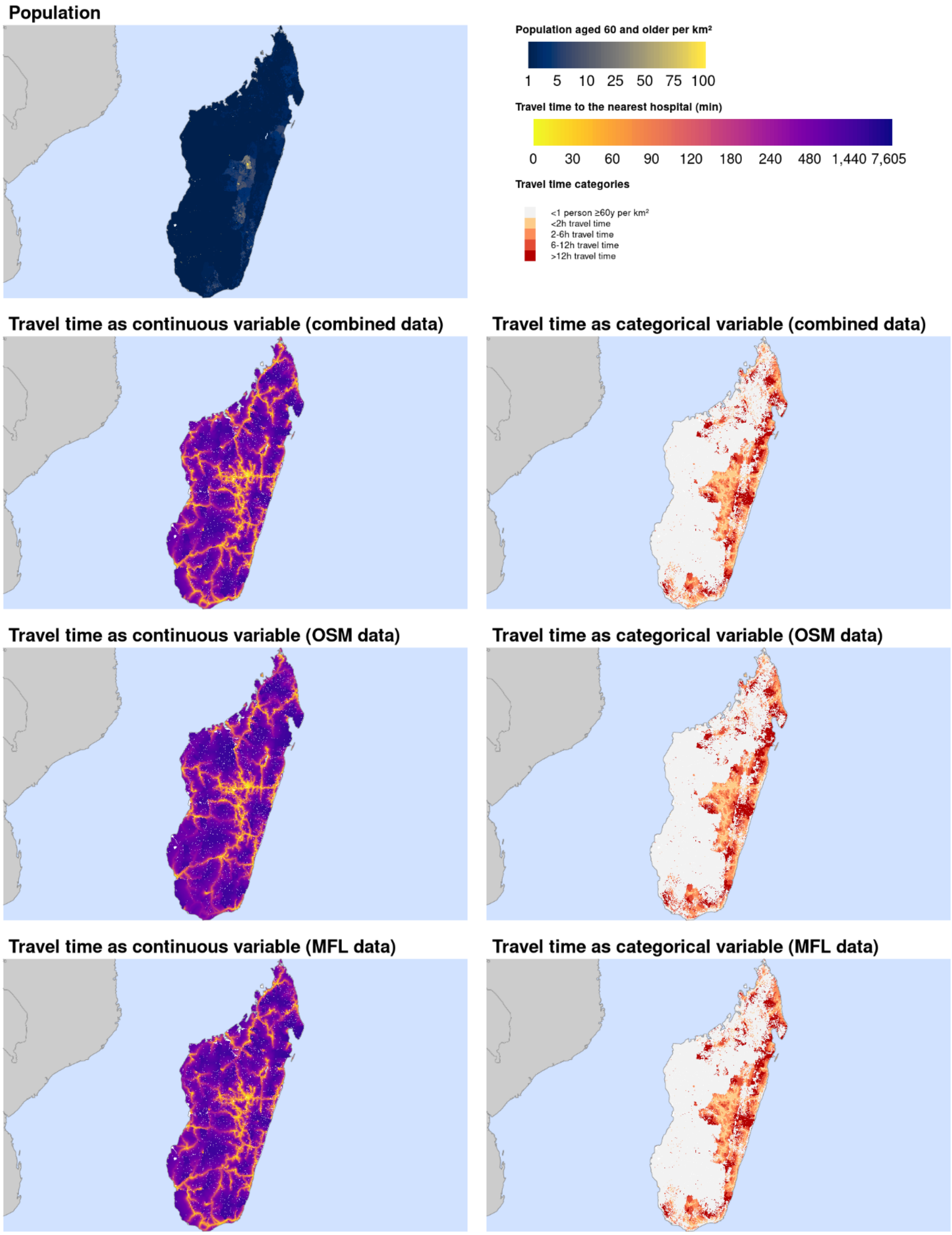

Figure S30. Malawi map of travel time to the nearest hospital for adults aged ≥ 60 years

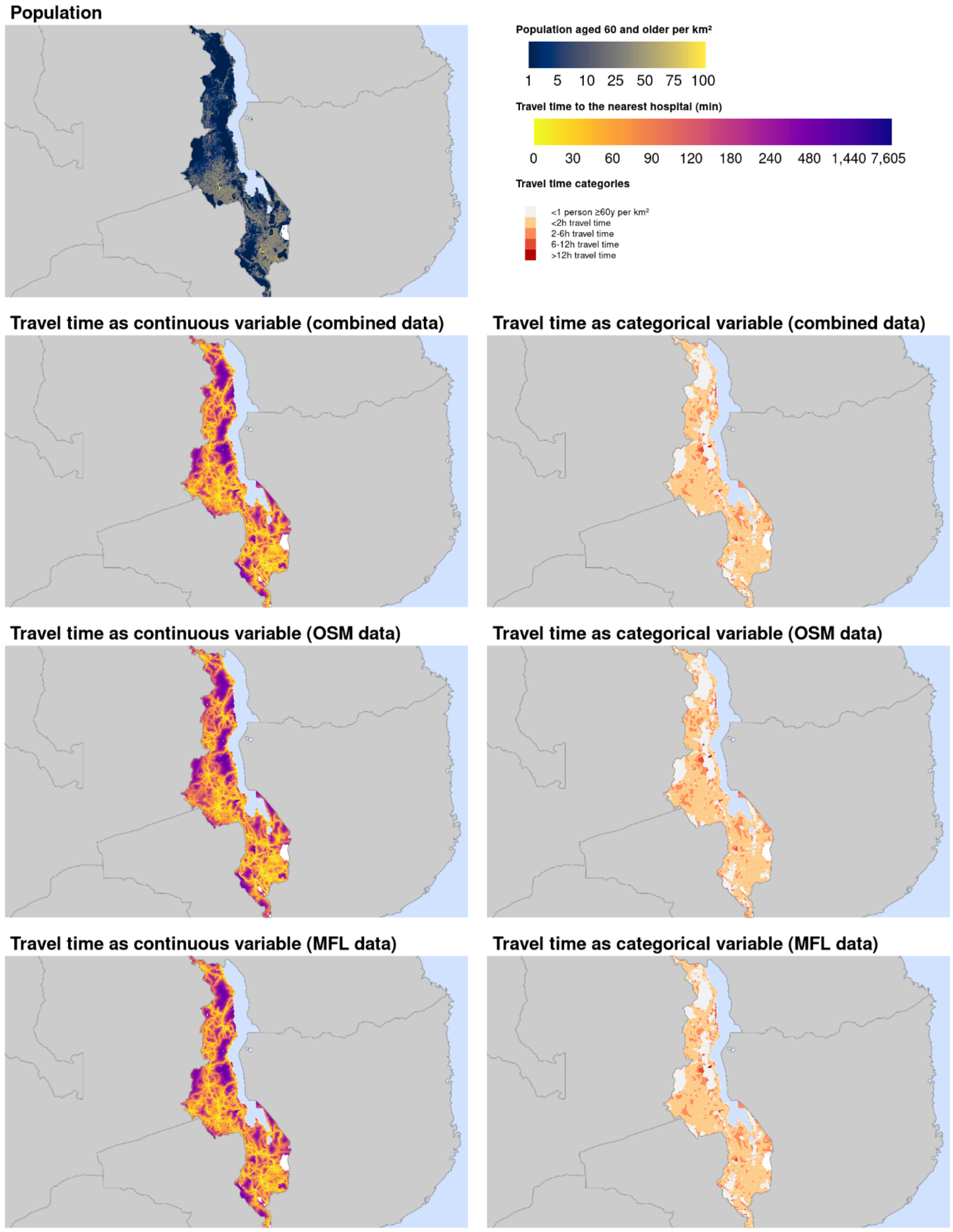

Figure S31. Mali map of travel time to the nearest hospital for adults aged ≥ 60 years

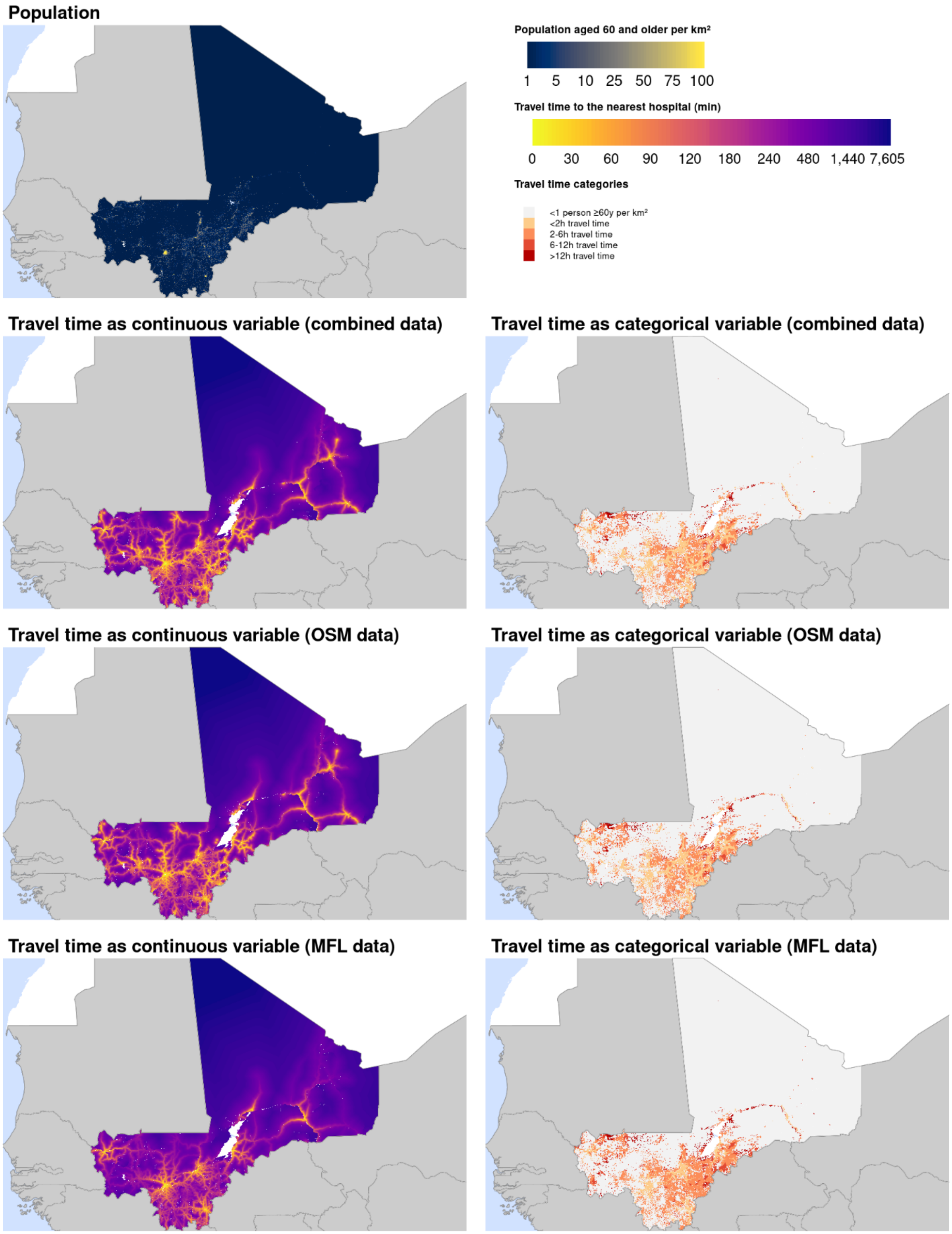

Figure S32. Mauritania map of travel time to the nearest hospital for adults aged ≥ 60 years

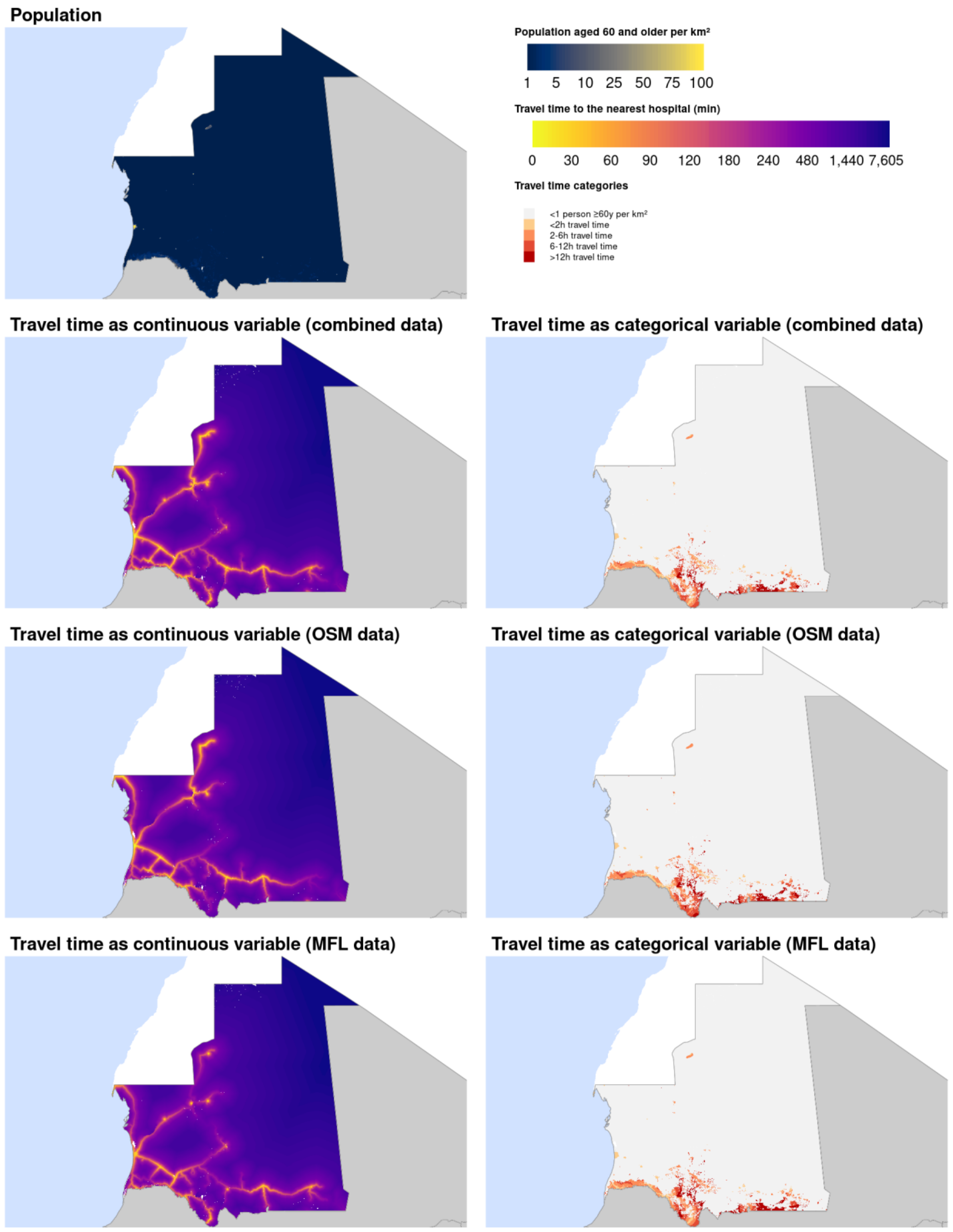

Figure S33. Mozambique map of travel time to the nearest hospital for adults aged ≥ 60 years

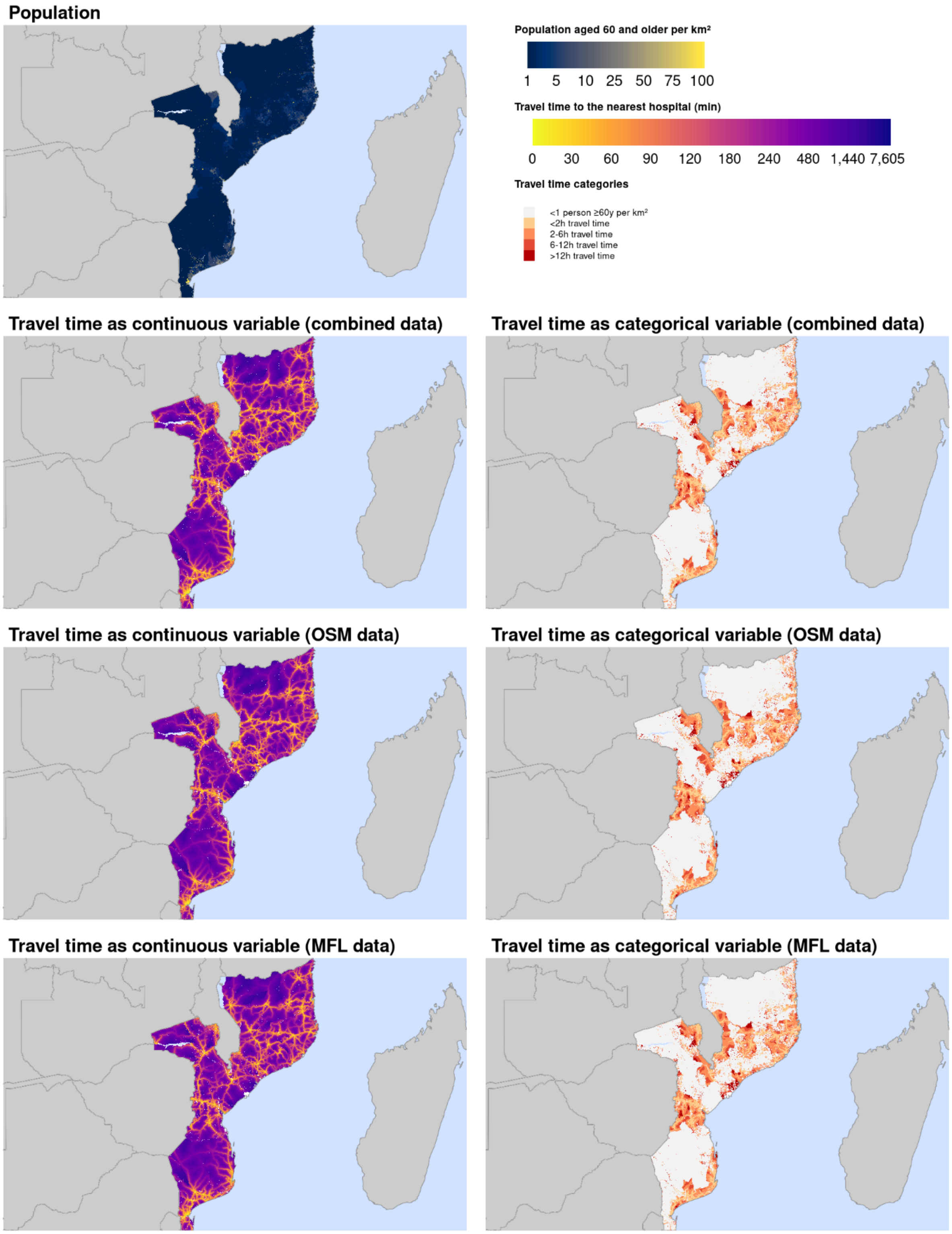

Figure S34. Namibia map of travel time to the nearest hospital for adults aged ≥ 60 years

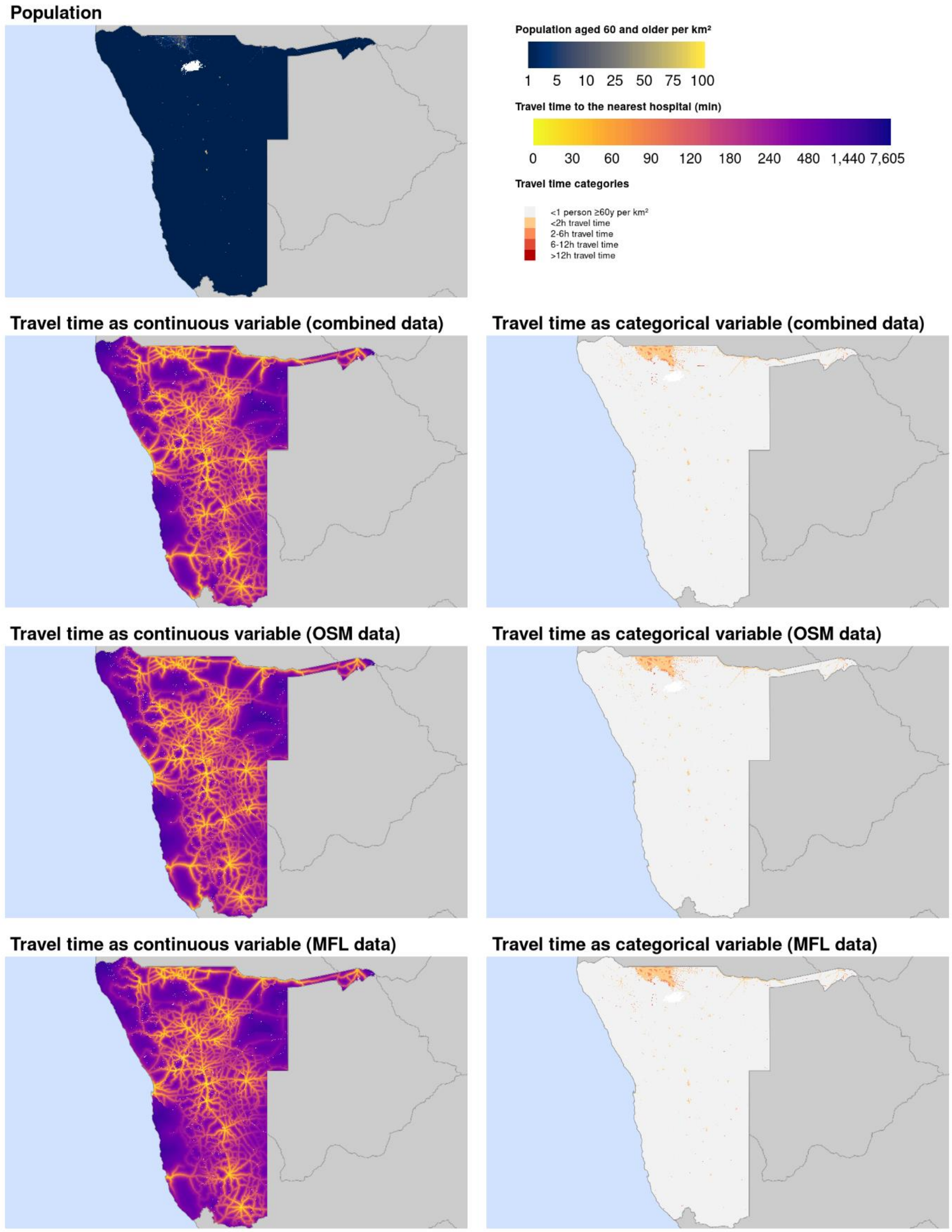

Figure S35. Niger map of travel time to the nearest hospital for adults aged ≥ 60 years

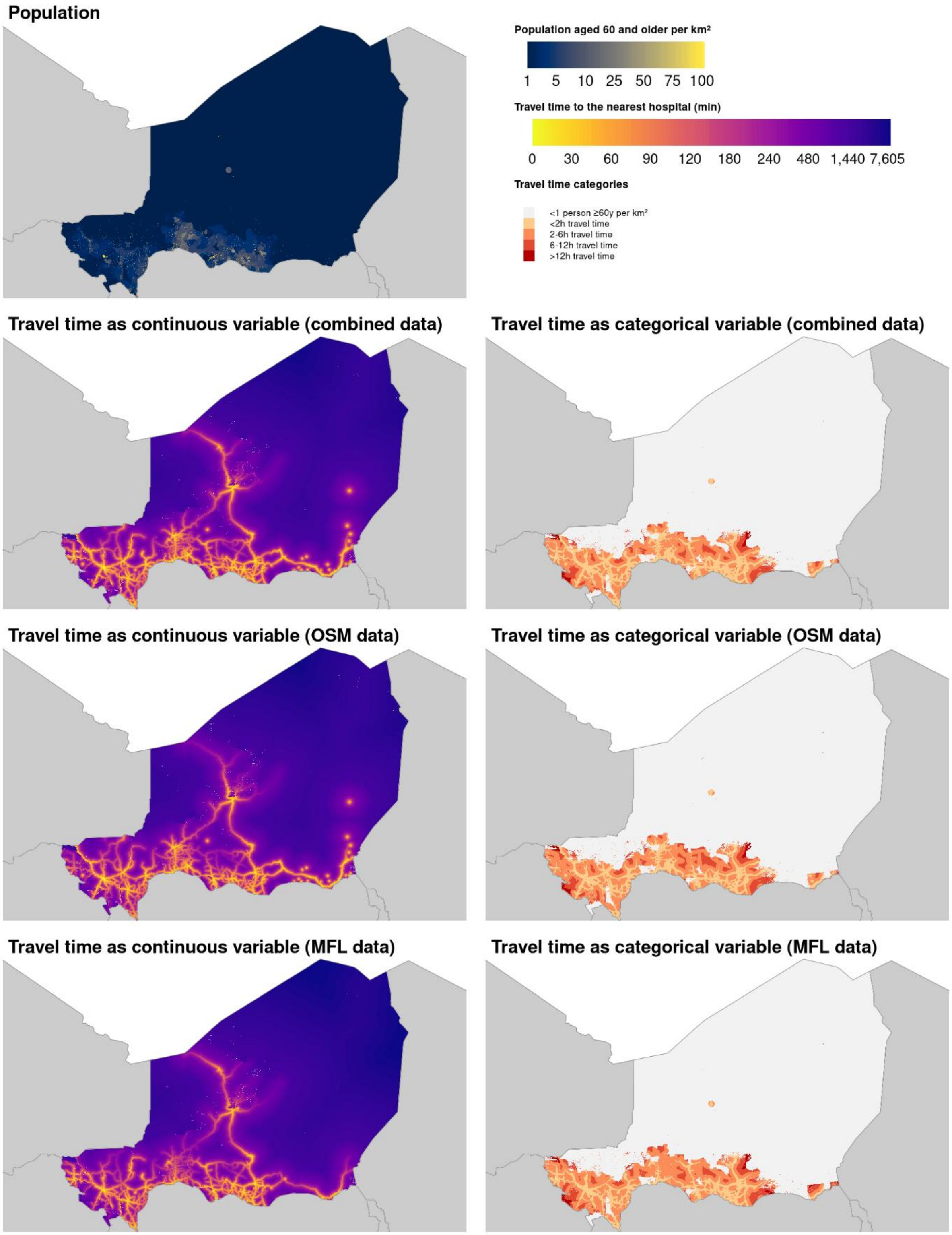

Figure S36. Nigeria map of travel time to the nearest hospital for adults aged ≥ 60 years

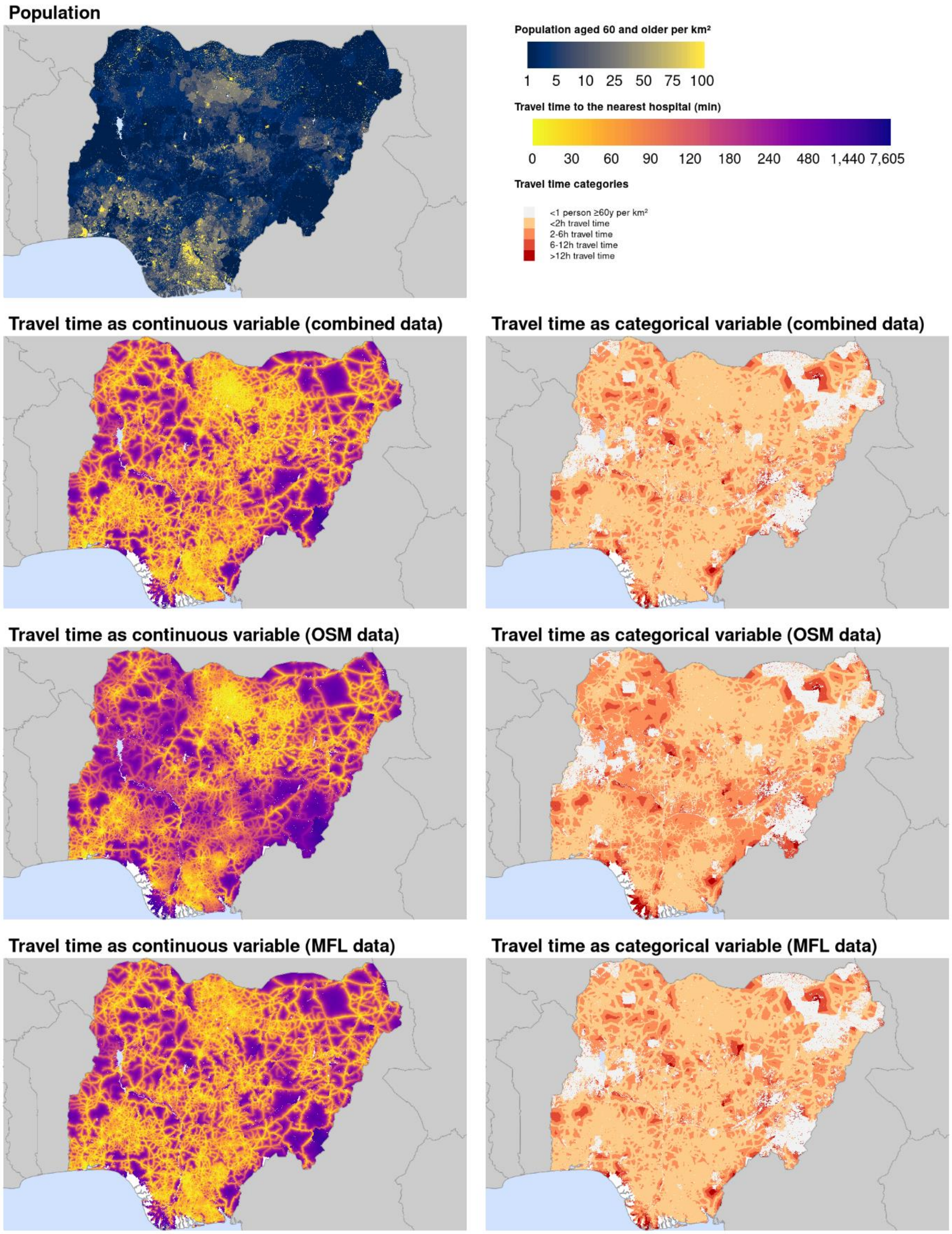

Figure S37. Republic of the Congo map of travel time to the nearest hospital for adults aged ≥ 60 years

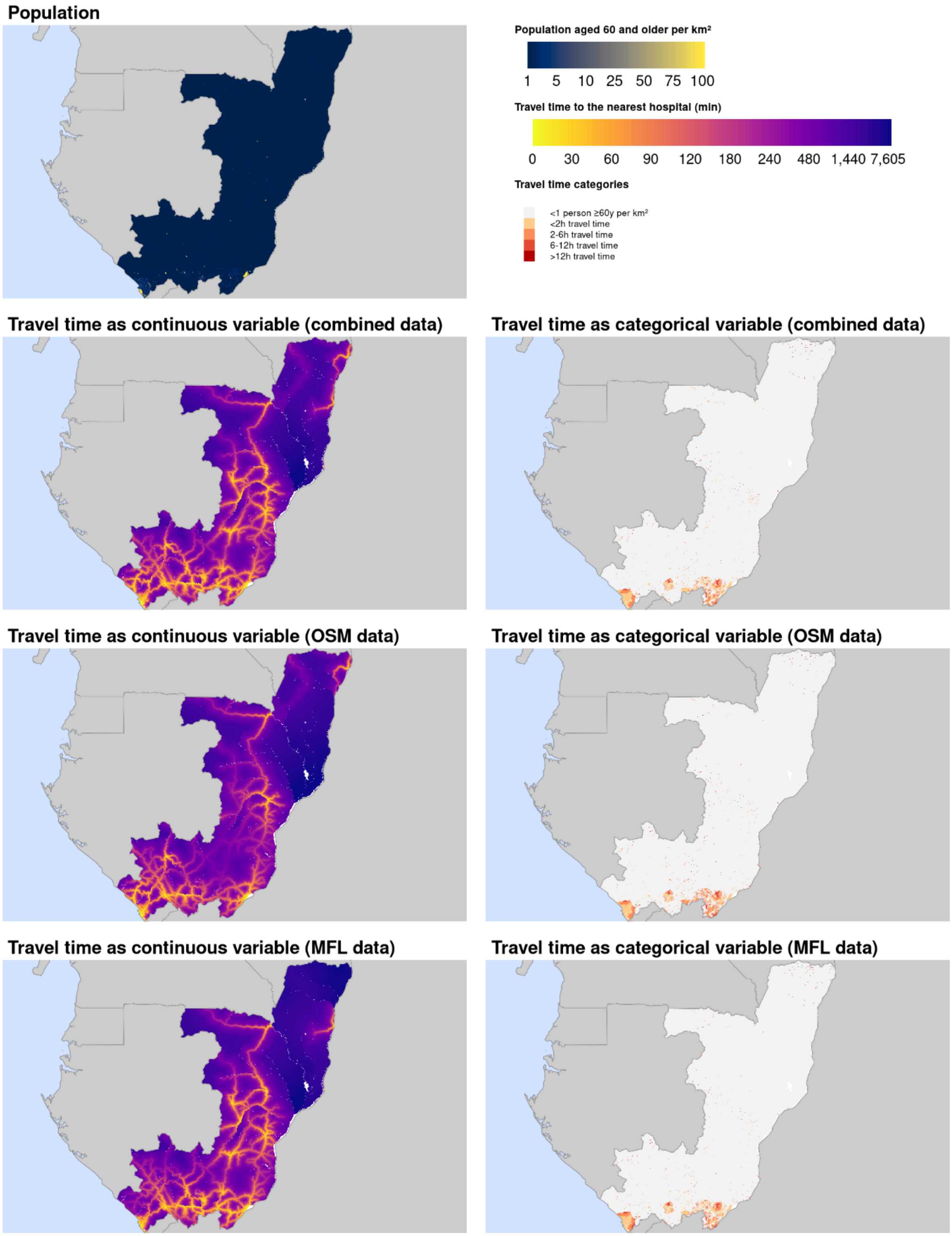

Figure S38. Rwanda map of travel time to the nearest hospital for adults aged ≥ 60 years

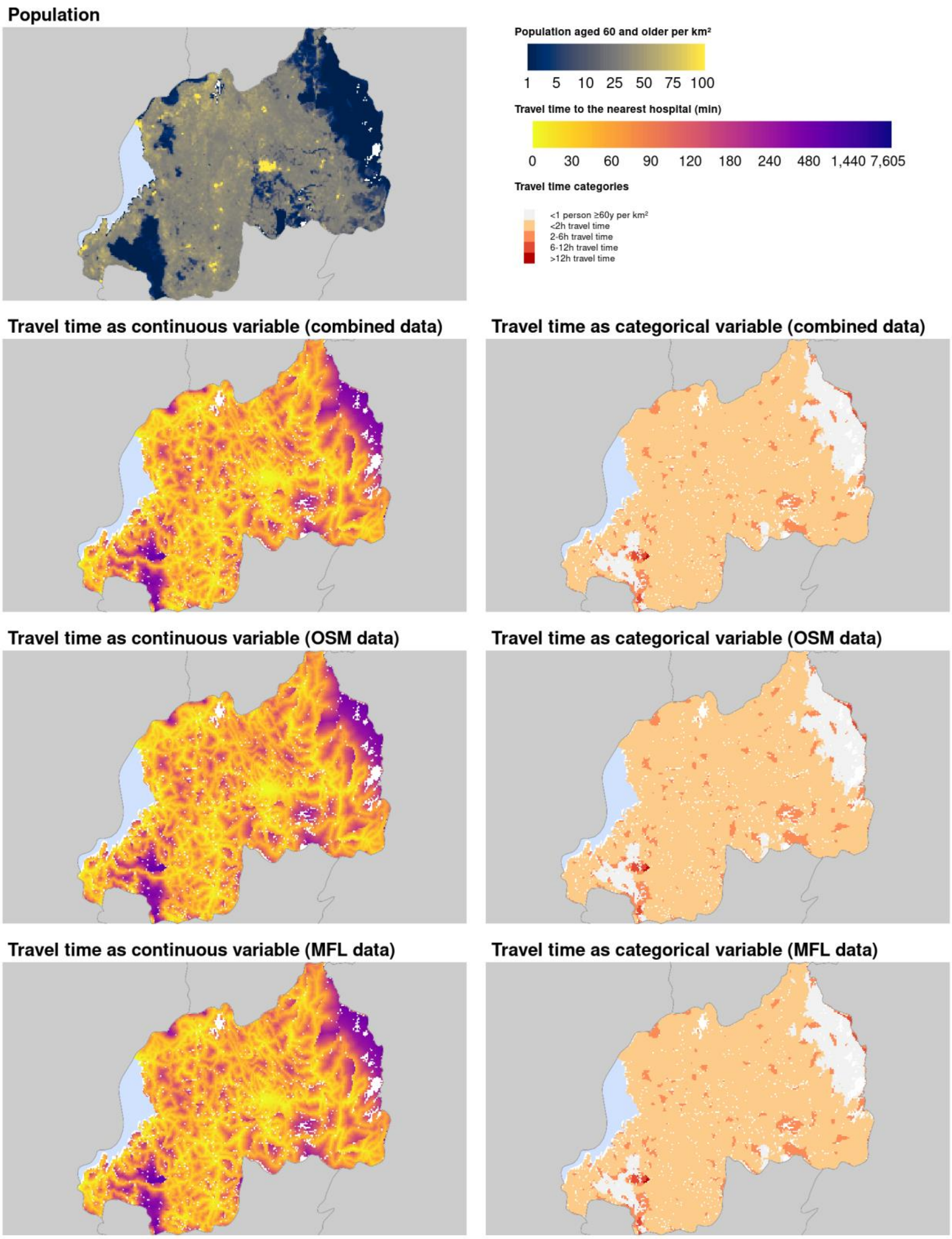

Figure S39. Senegal map of travel time to the nearest hospital for adults aged ≥ 60 years

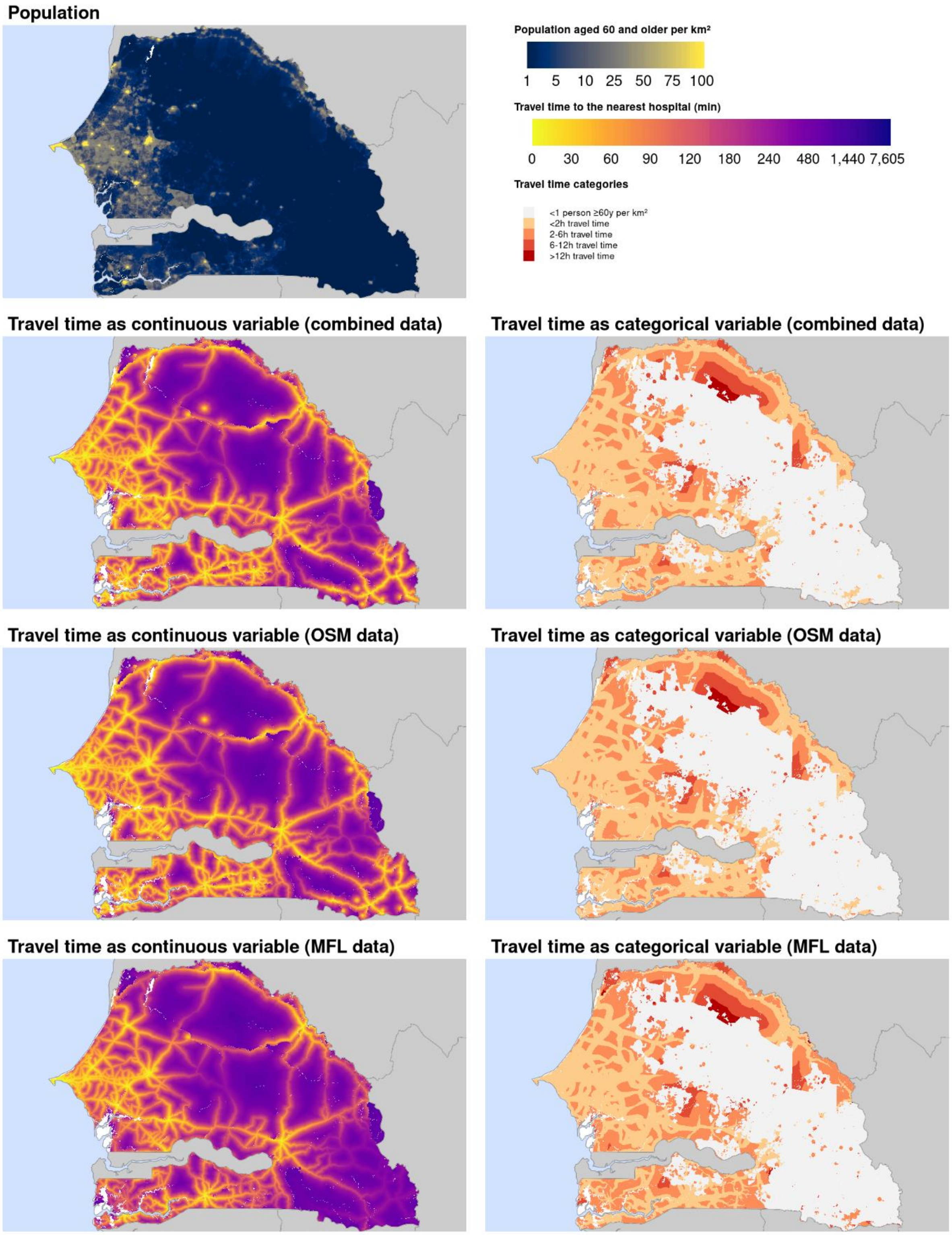

Figure S40. Sierra Leone map of travel time to the nearest hospital for adults aged ≥ 60 years

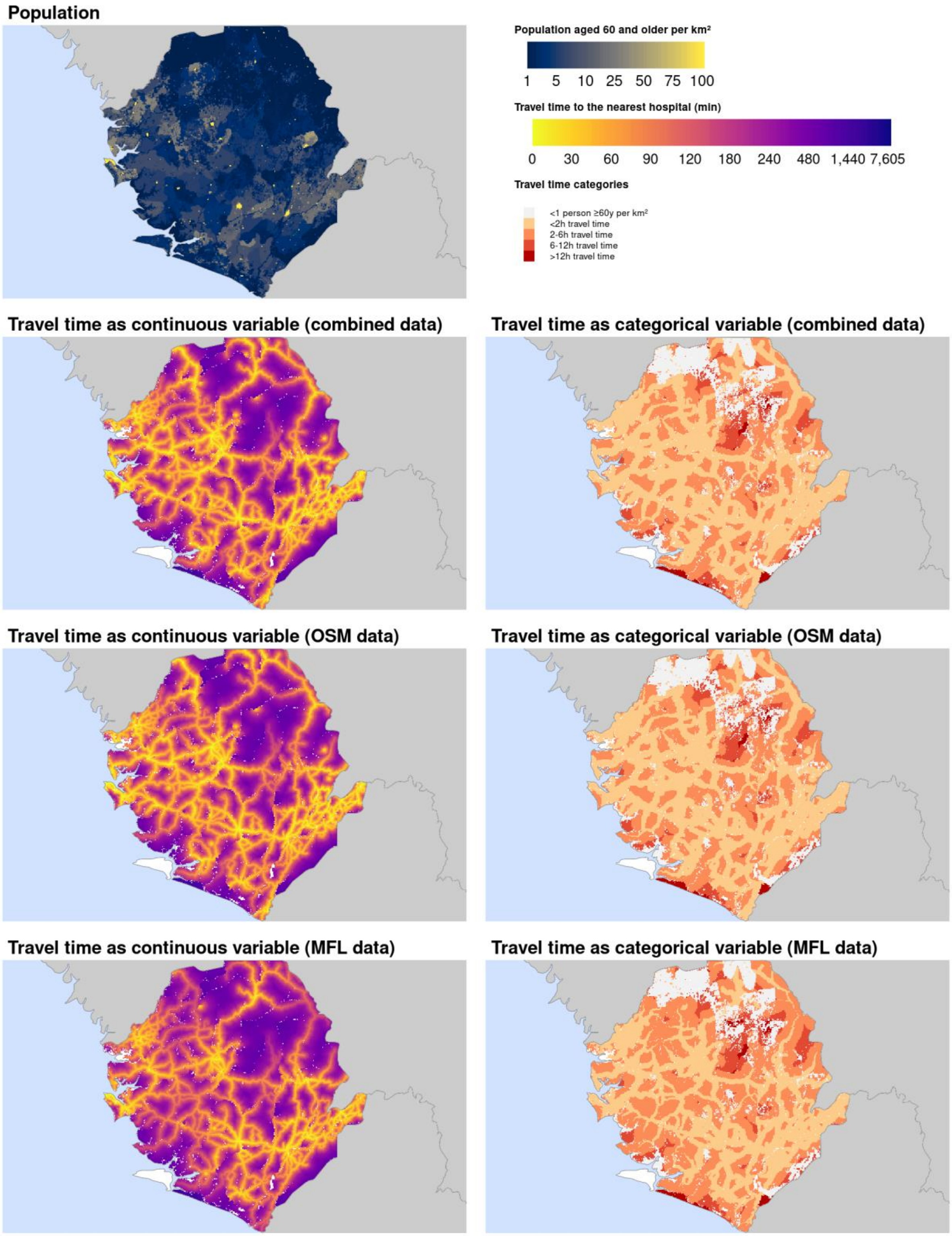

Figure S41. Somalia map of travel time to the nearest hospital for adults aged ≥ 60 years

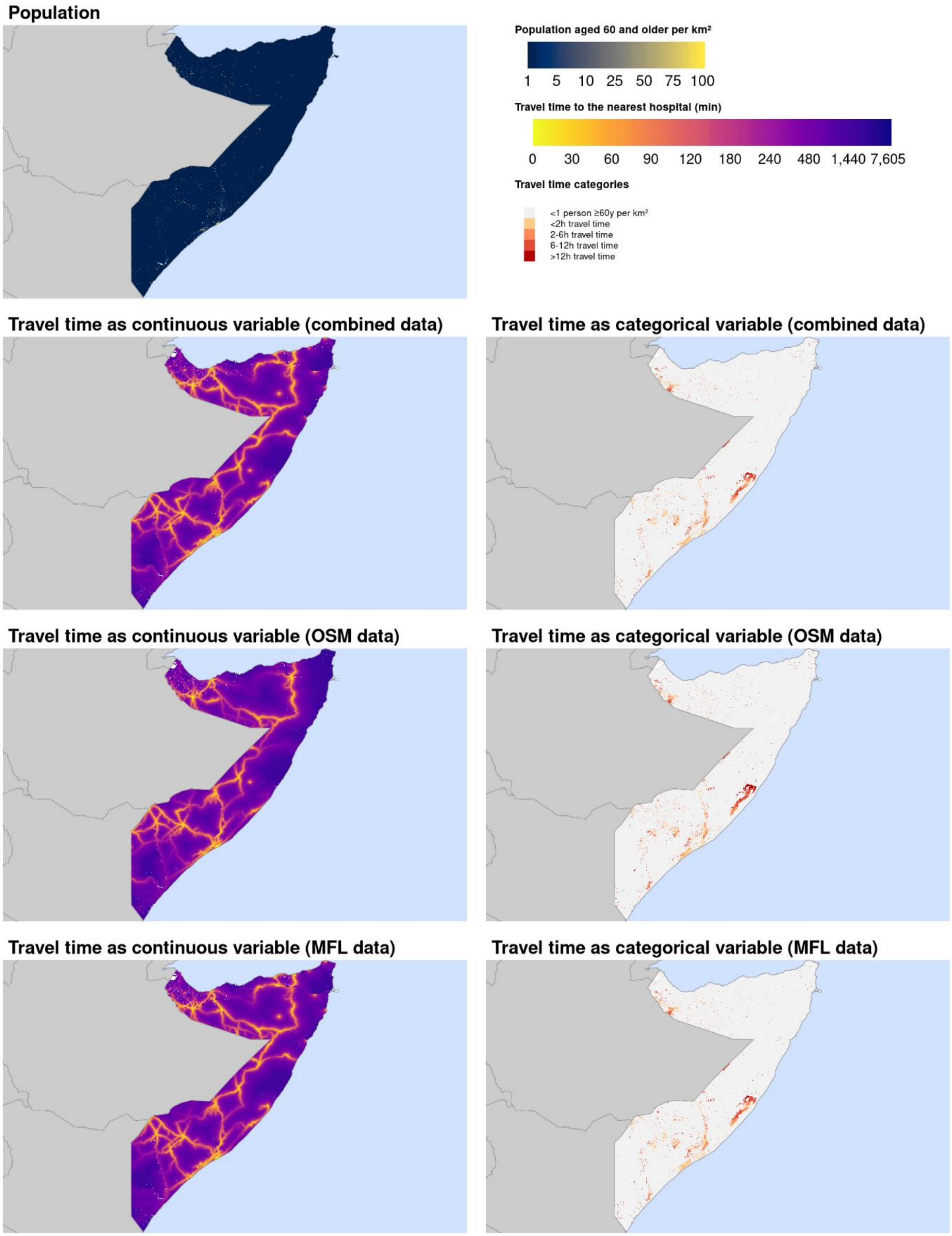

Figure S42. South Africa map of travel time to the nearest hospital for adults aged ≥ 60 years

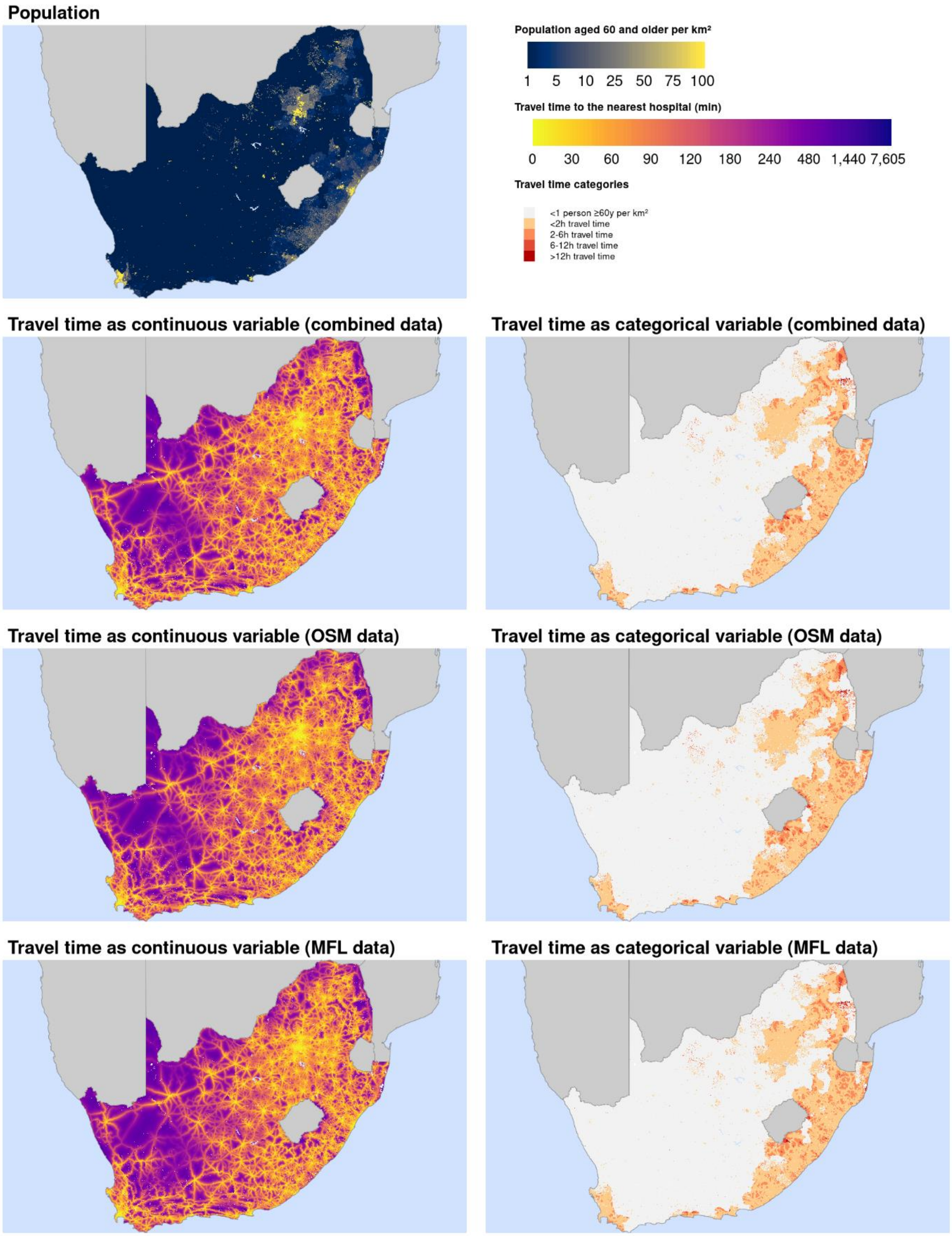

Figure S43. South Sudan map of travel time to the nearest hospital for adults aged ≥ 60 years

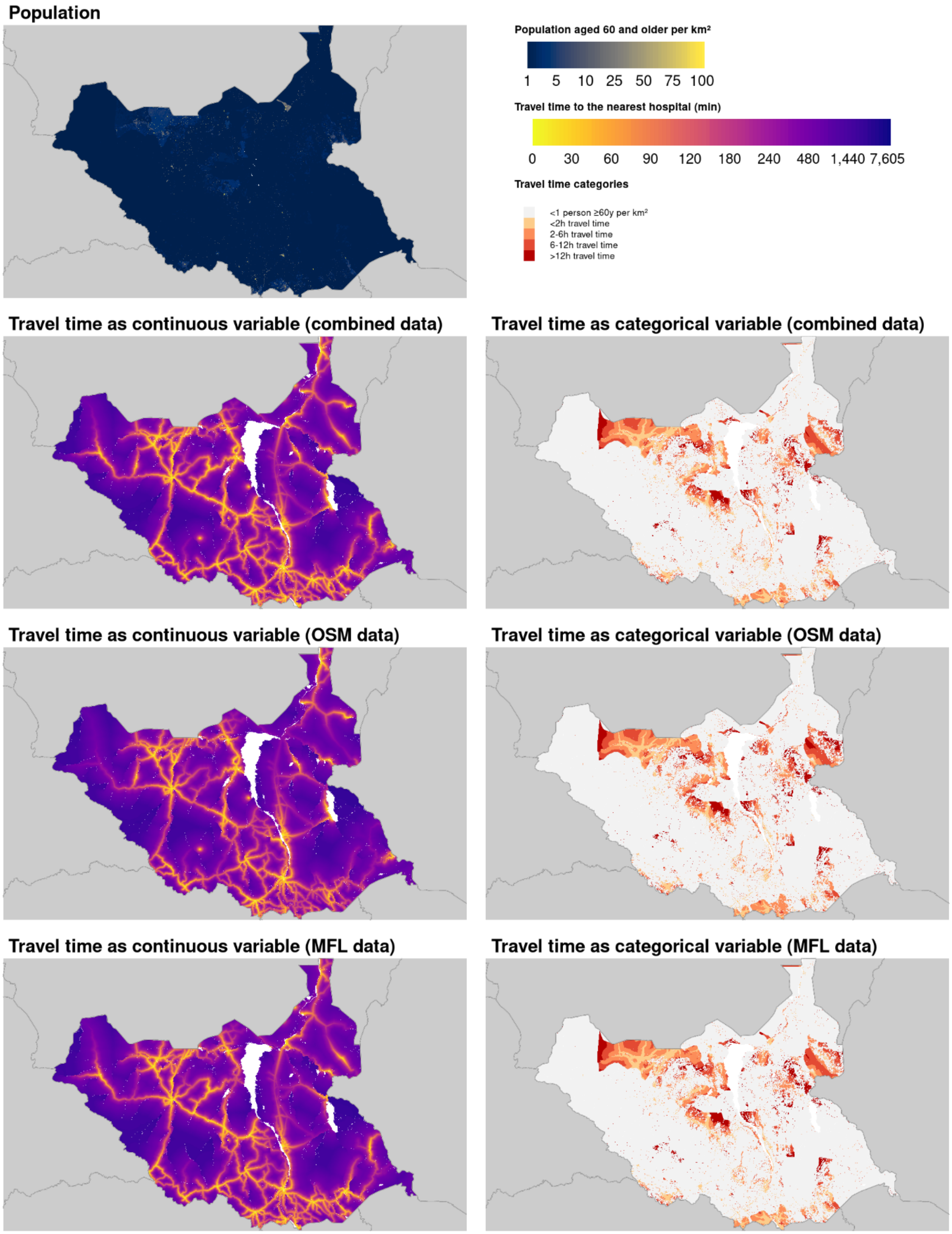

Figure S44. Sudan map of travel time to the nearest hospital for adults aged ≥ 60 years

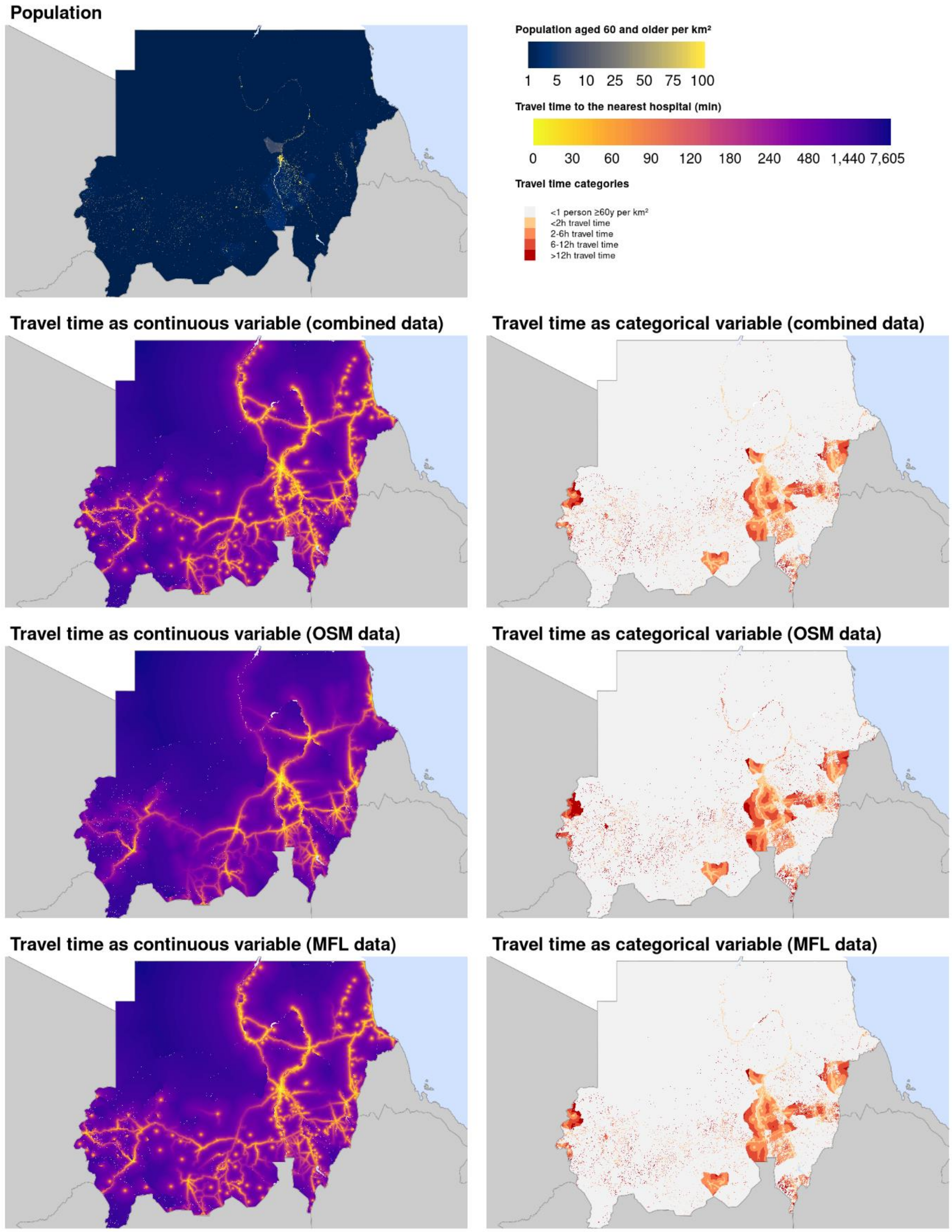

Figure S45. Tanzania map of travel time to the nearest hospital for adults aged ≥ 60 years

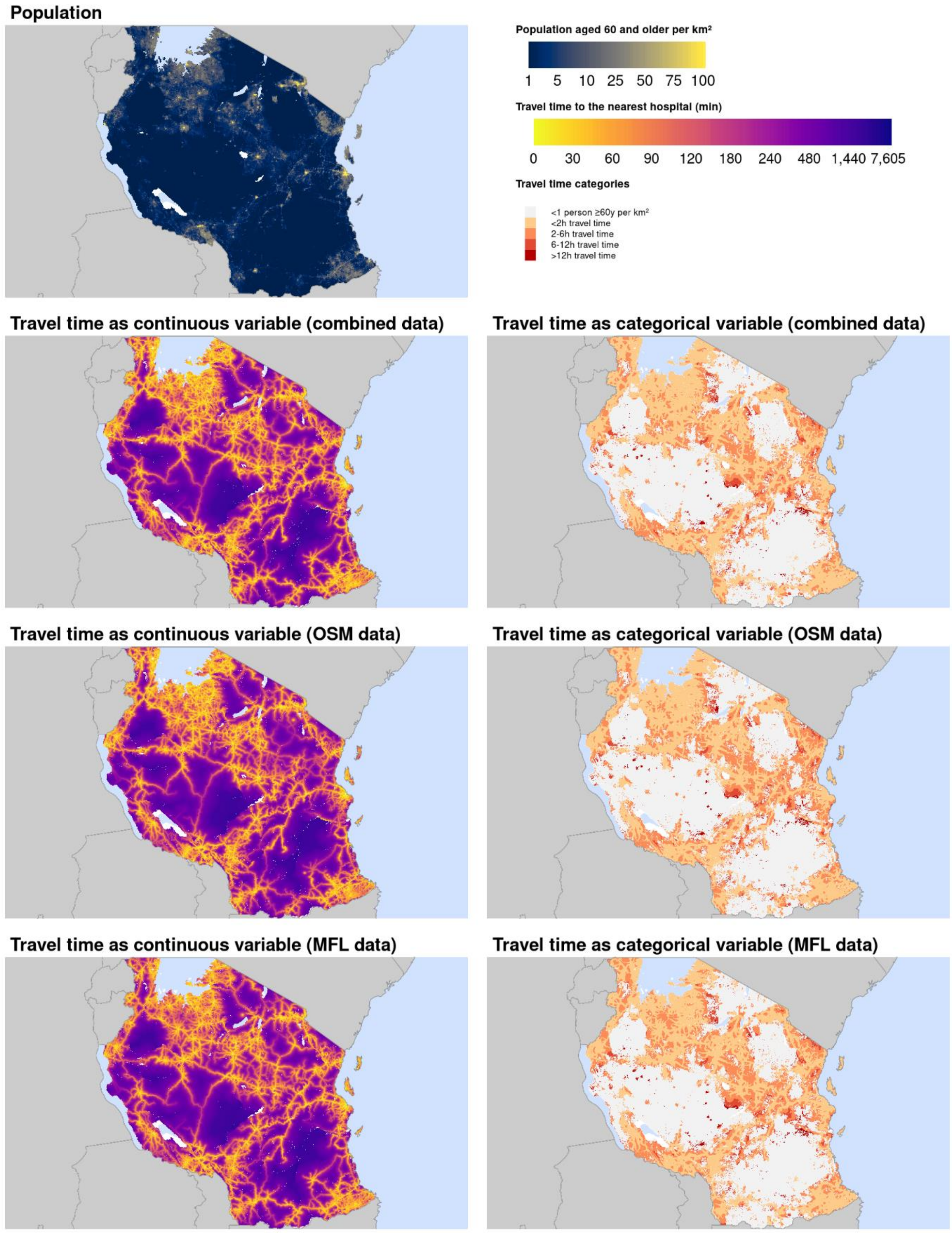

Figure S46. The Gambia map of travel time to the nearest hospital for adults aged ≥ 60 years

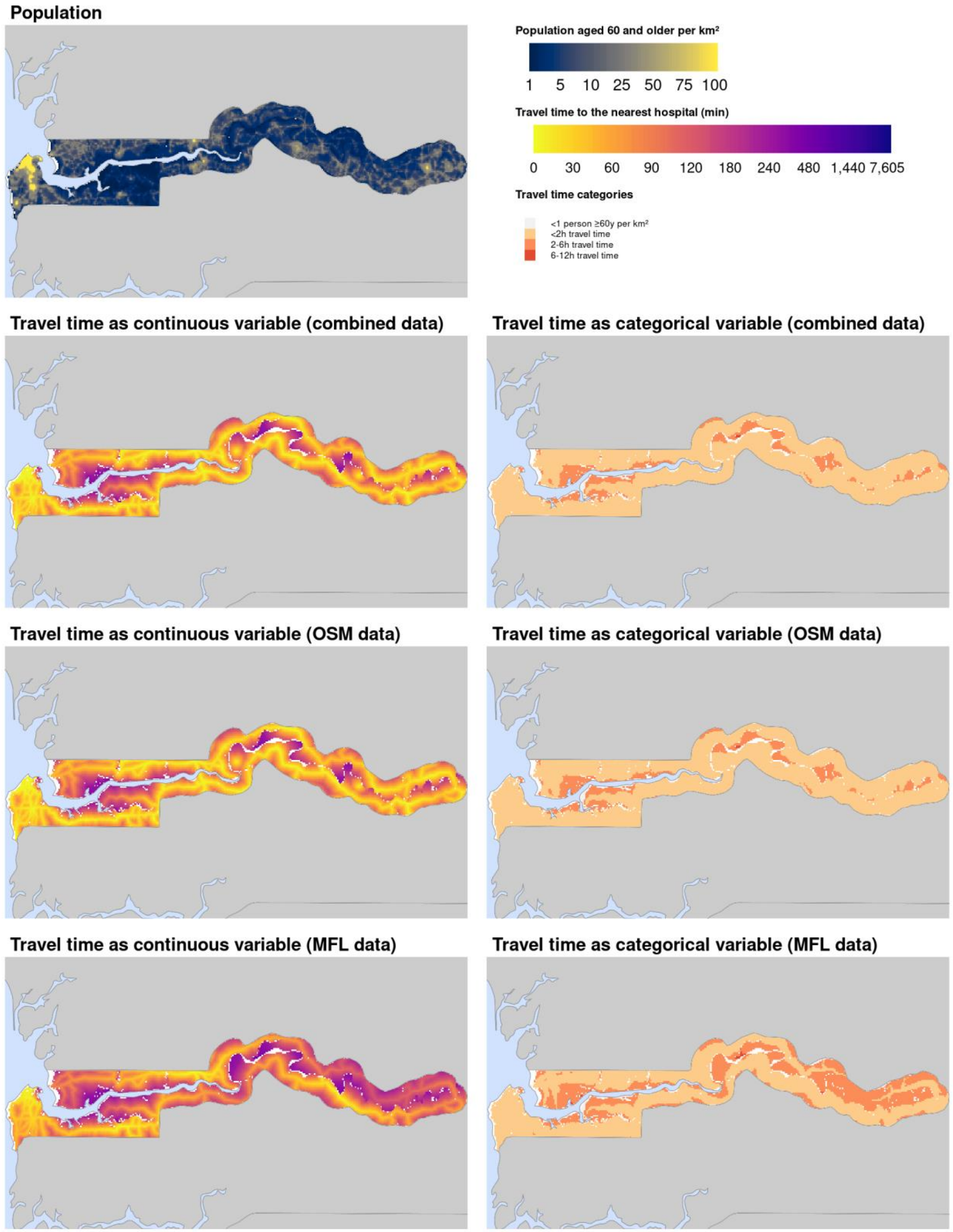

Figure S47. Togo map of travel time to the nearest hospital for adults aged ≥ 60 years

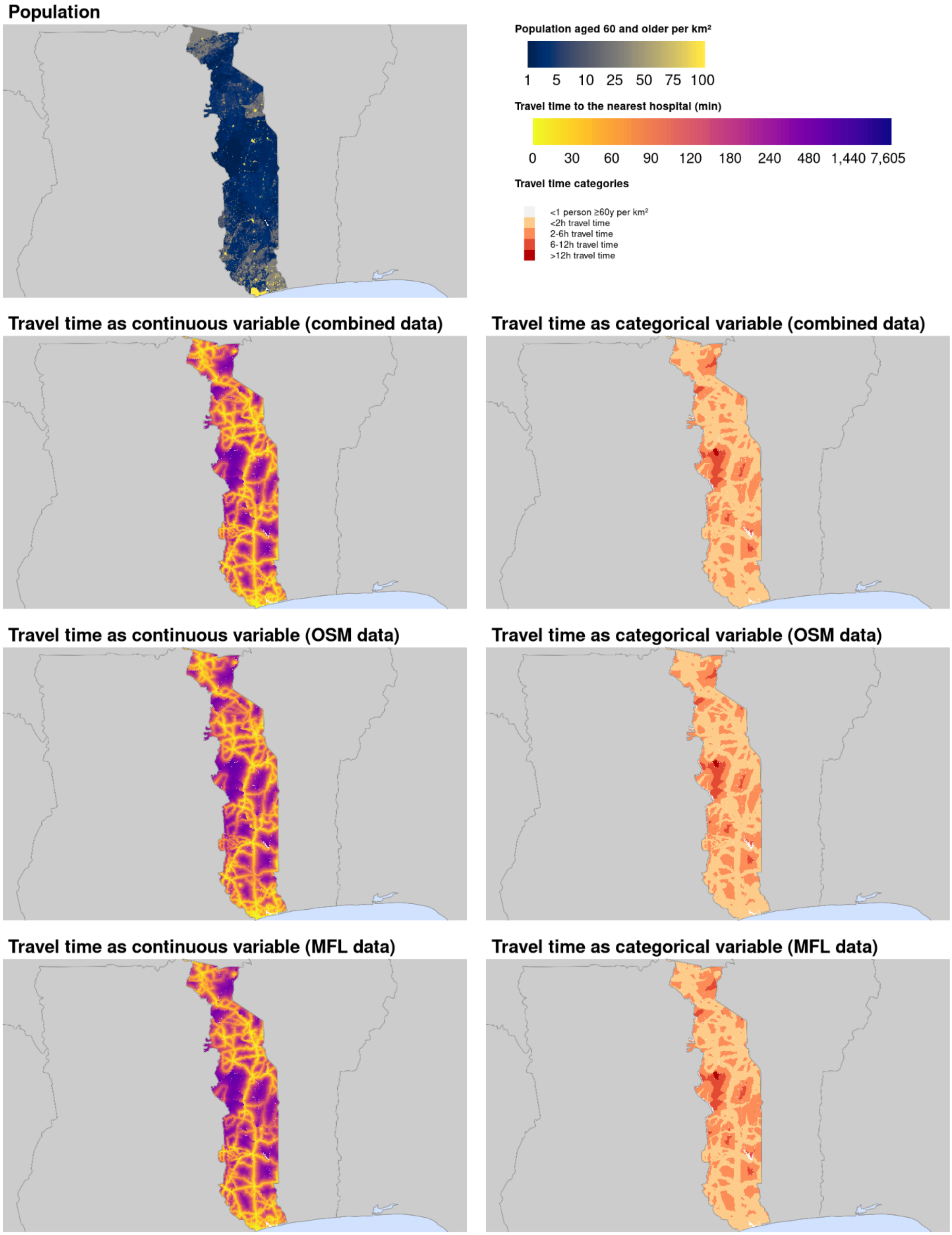

Figure S48. Uganda map of travel time to the nearest hospital for adults aged ≥ 60 years

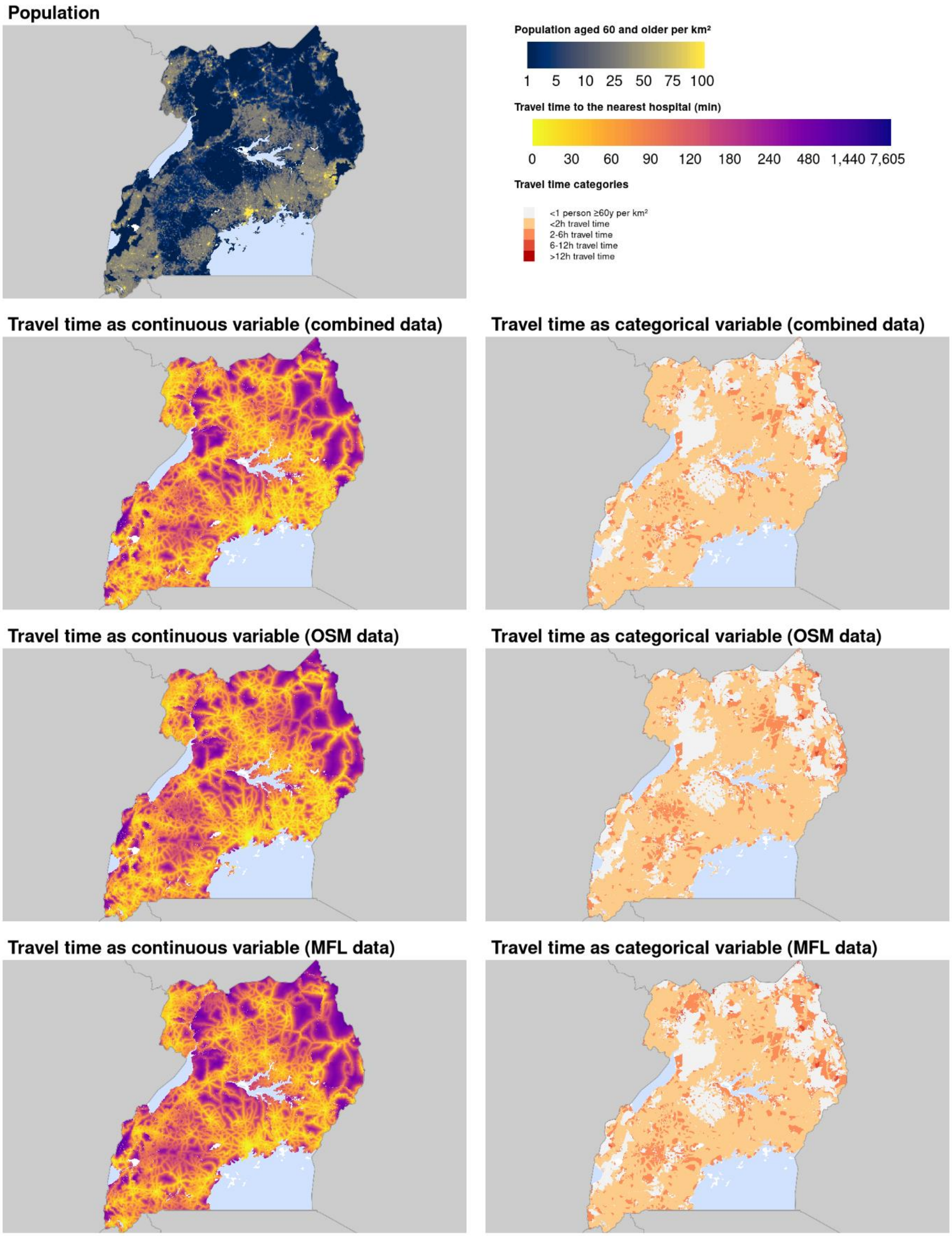

Figure S49. Zambia map of travel time to the nearest hospital for adults aged ≥ 60 years

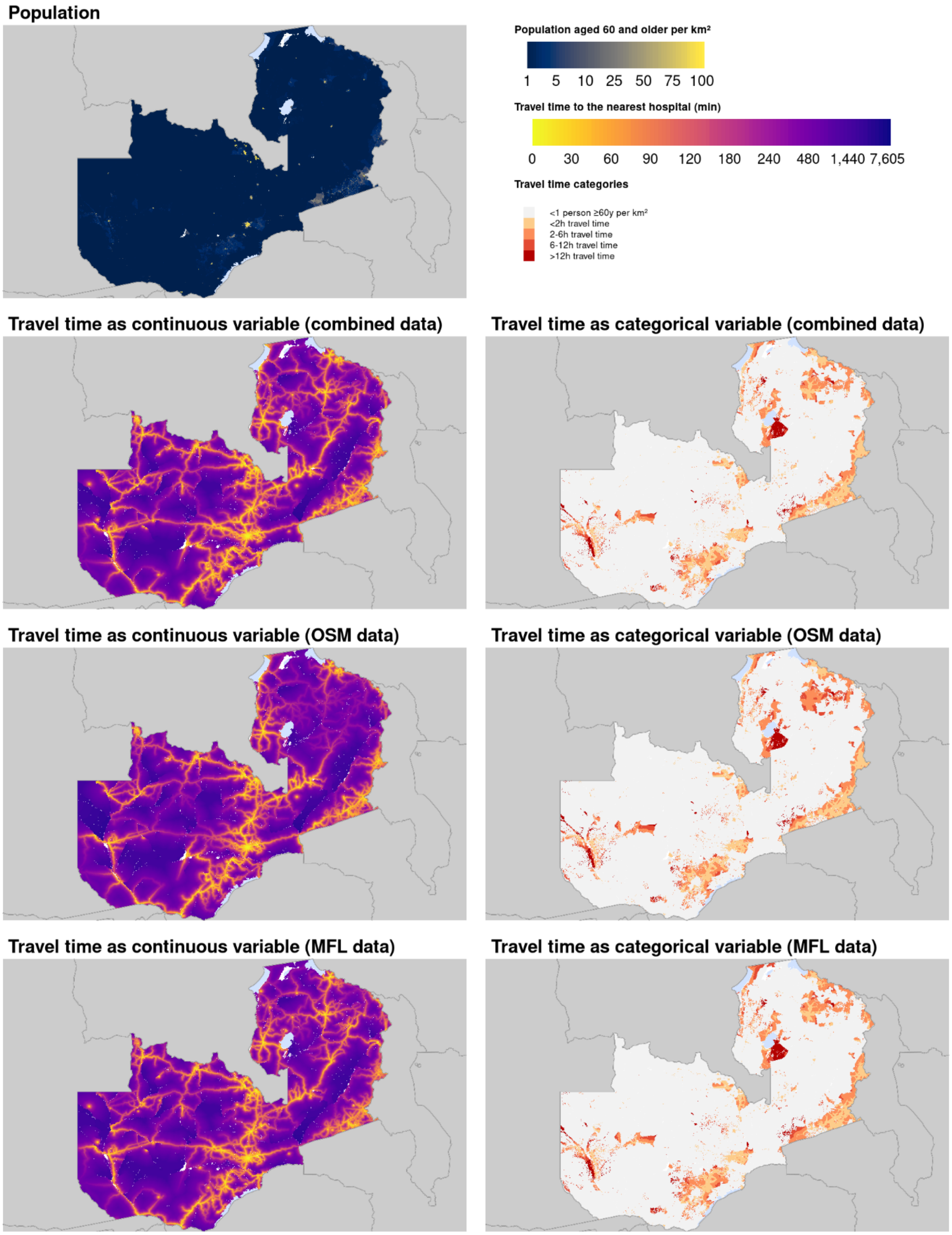

Figure S50. Zimbabwe map of travel time to the nearest hospital for adults aged ≥ 60 years

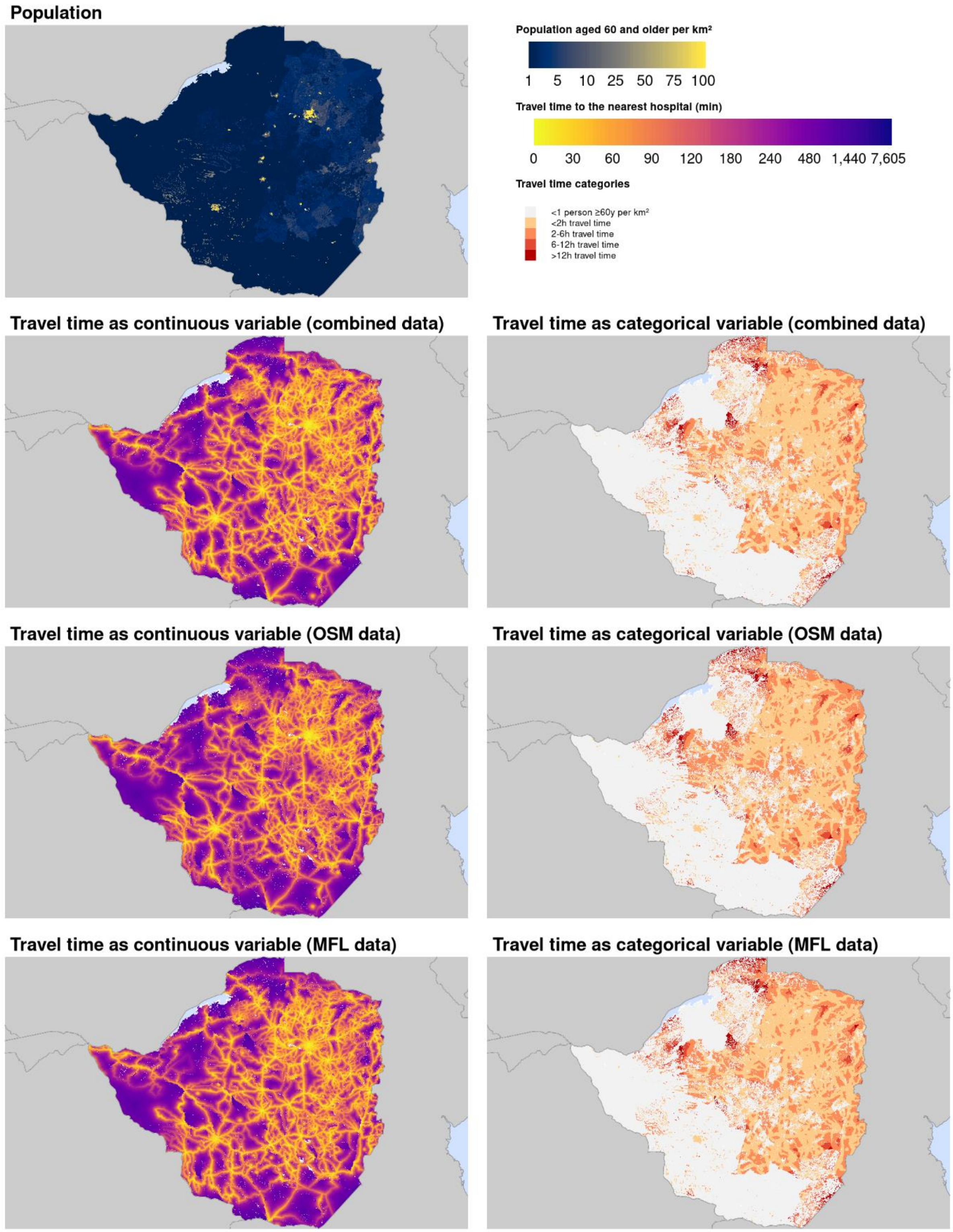

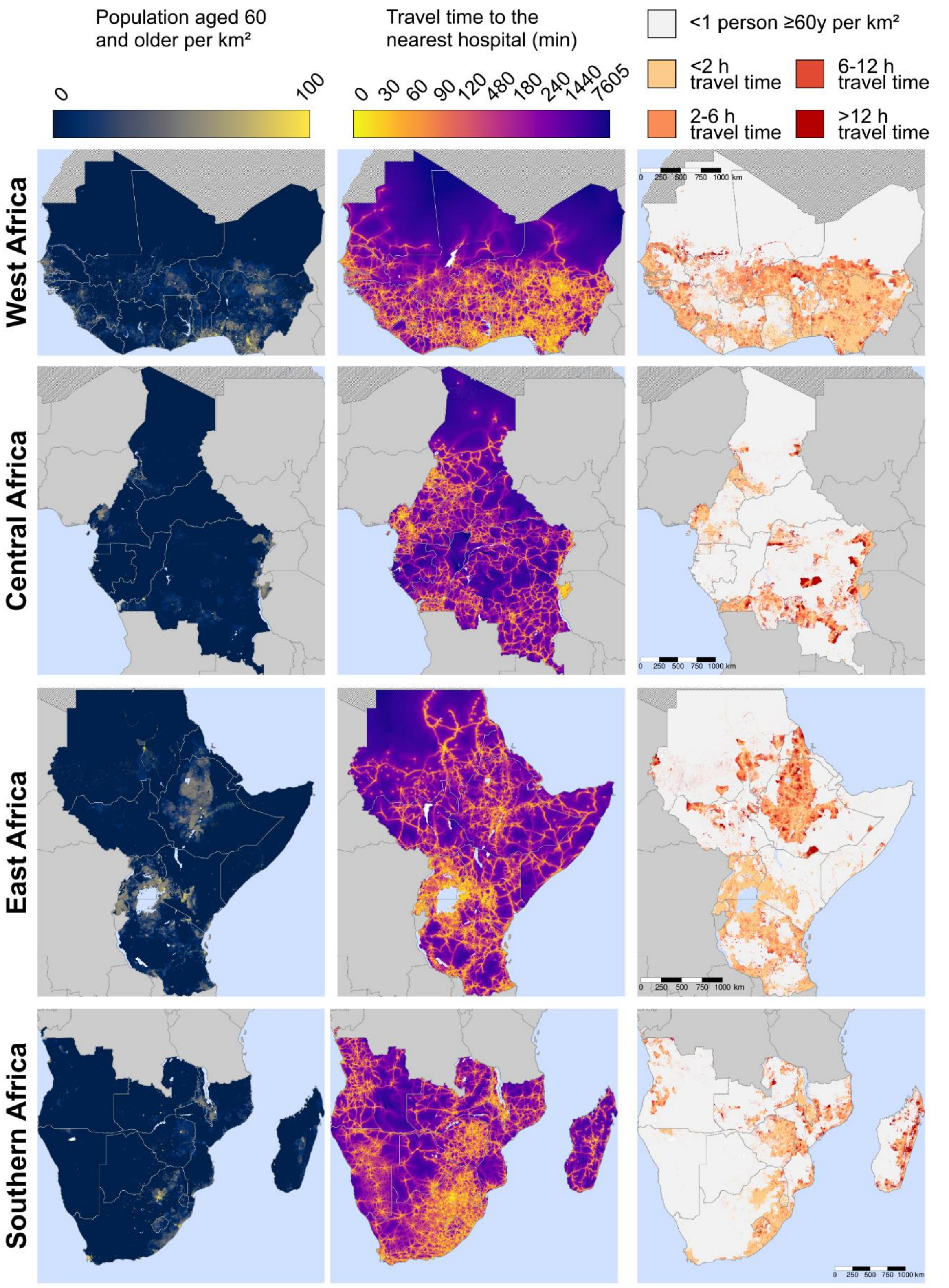

Figure S51. Maps of travel time to the nearest hospital for adults ≥ 60 years, by region based on the MFL dataset

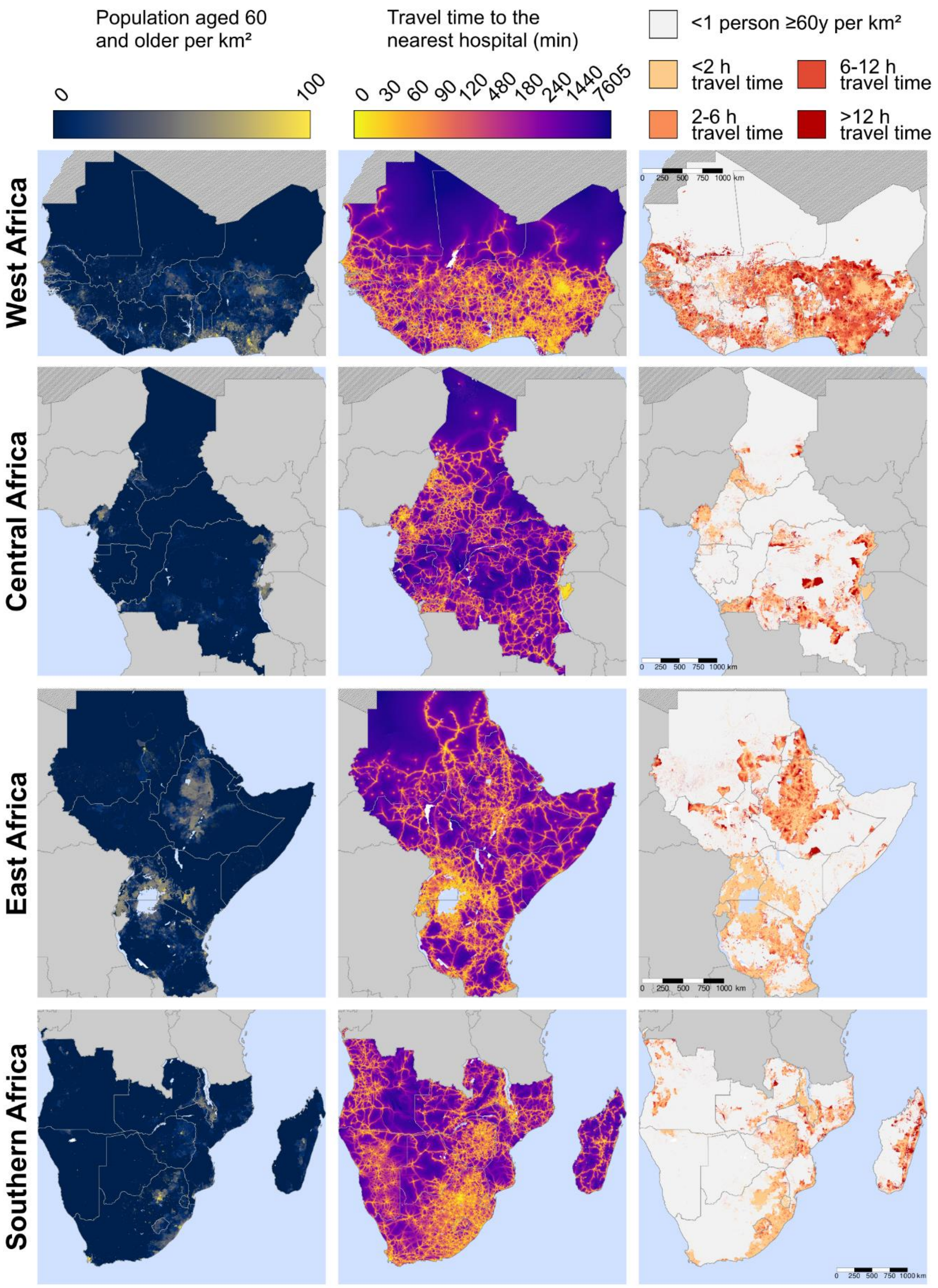

Figure S52. Maps of travel time to the nearest hospital for adults ≥ 60 years, by region based on the OSM dataset

Figure S53. Angola map of travel time to the nearest healthcare facility for adults aged ≥ 60 years

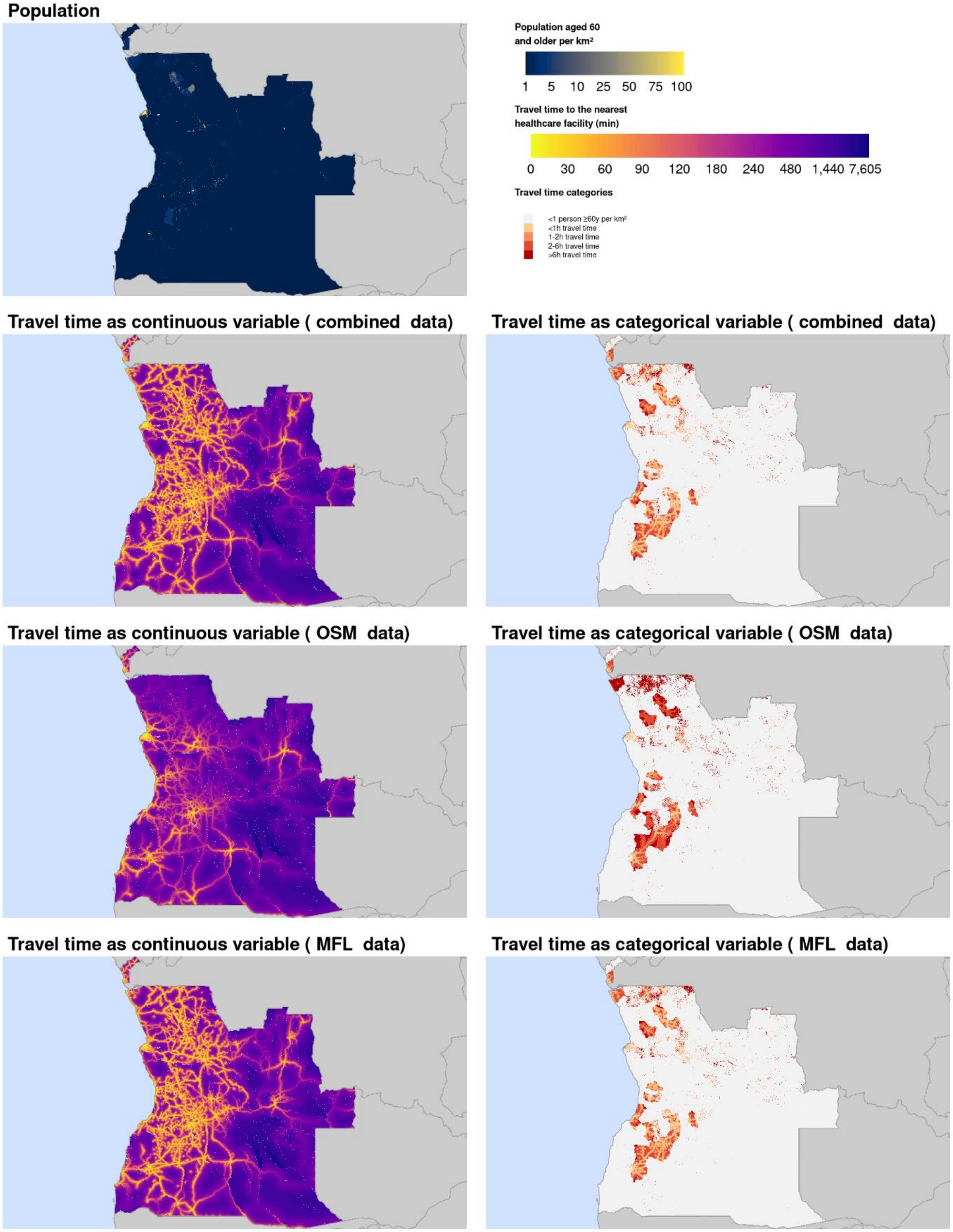

Figure S54. Benin map of travel time to the nearest healthcare facility for adults aged ≥ 60 years

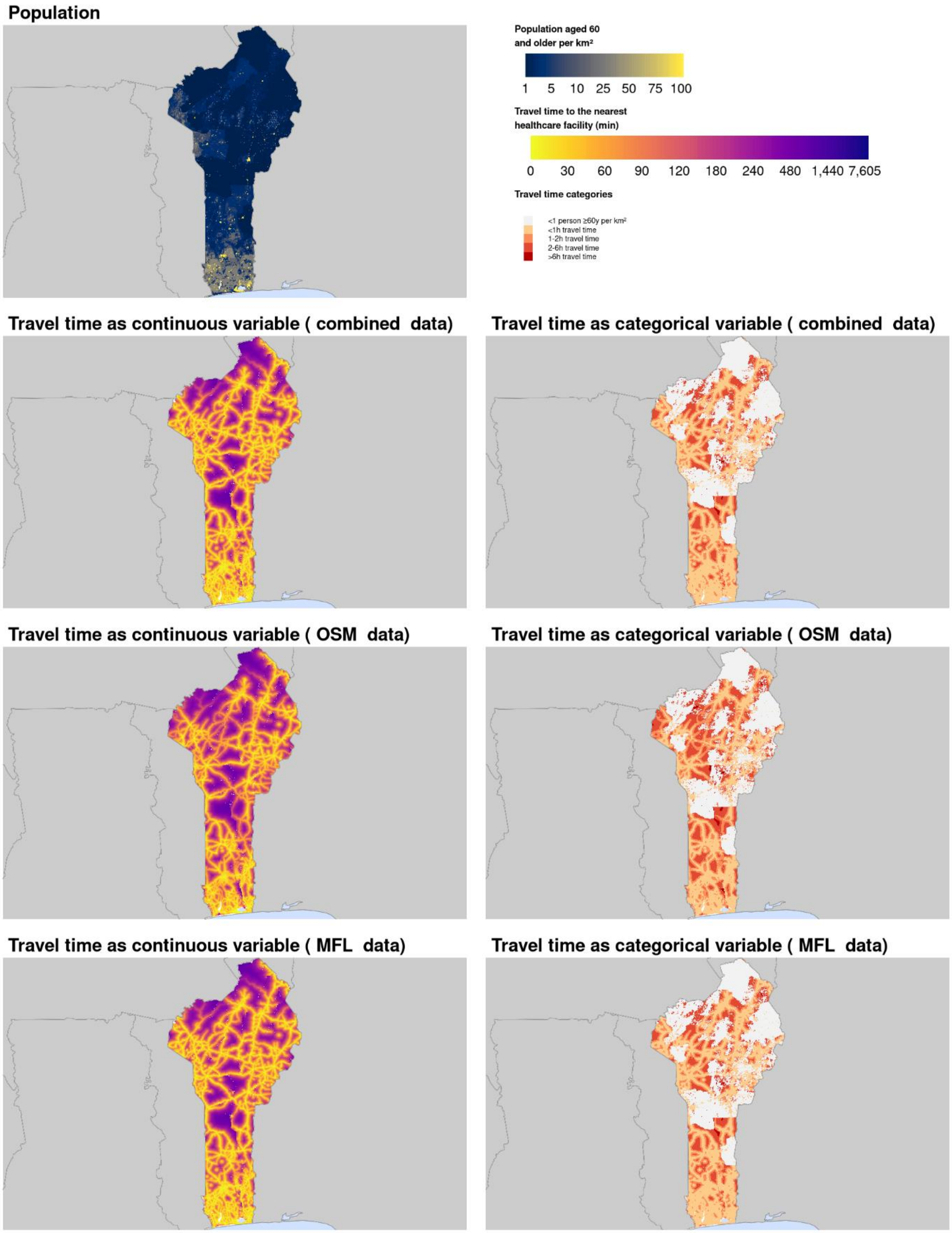

Figure S55. Botswana map of travel time to the nearest healthcare facility for adults aged ≥ 60 years

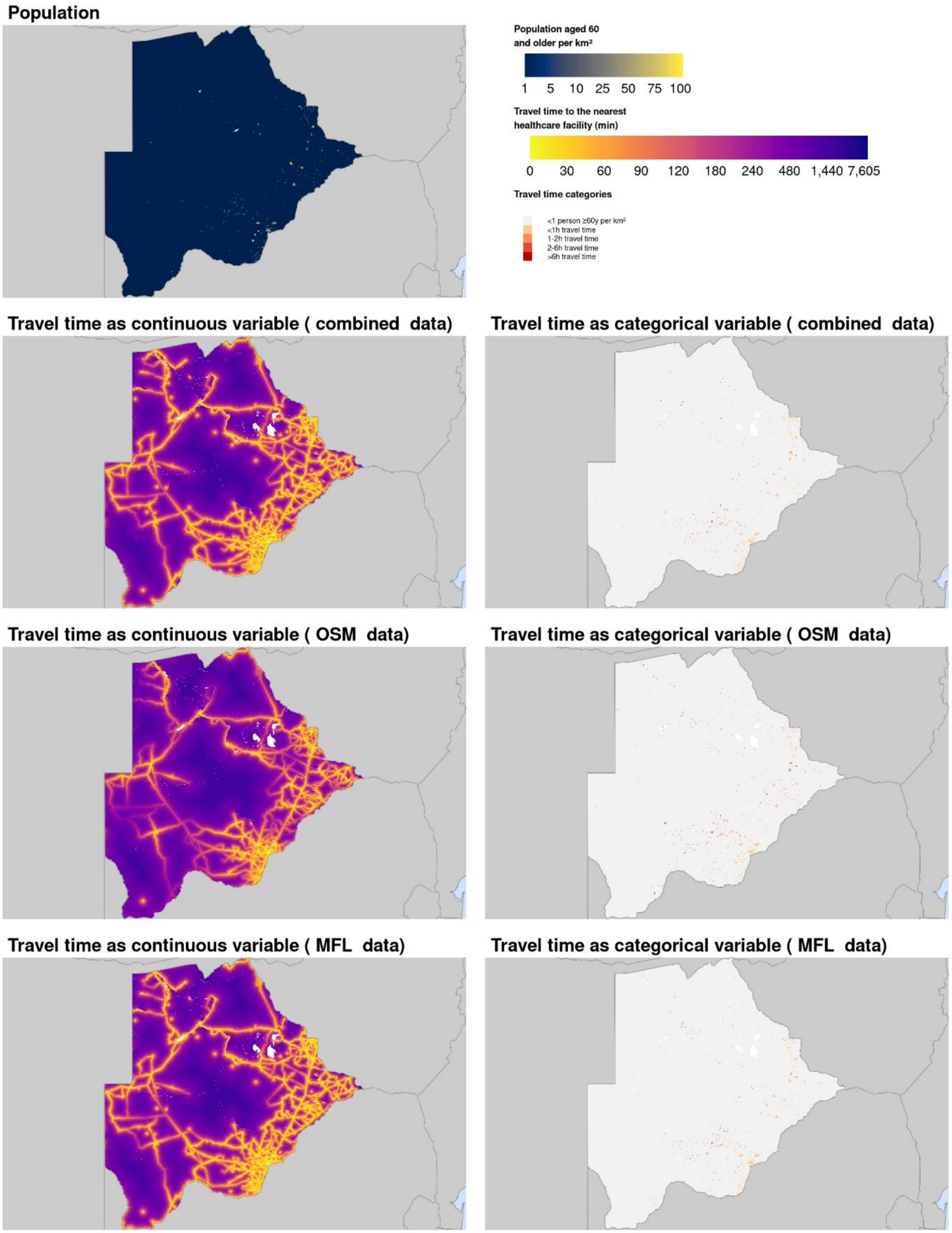

Figure S56. Burkina Faso map of travel time to the nearest healthcare facility for adults aged ≥ 60 years

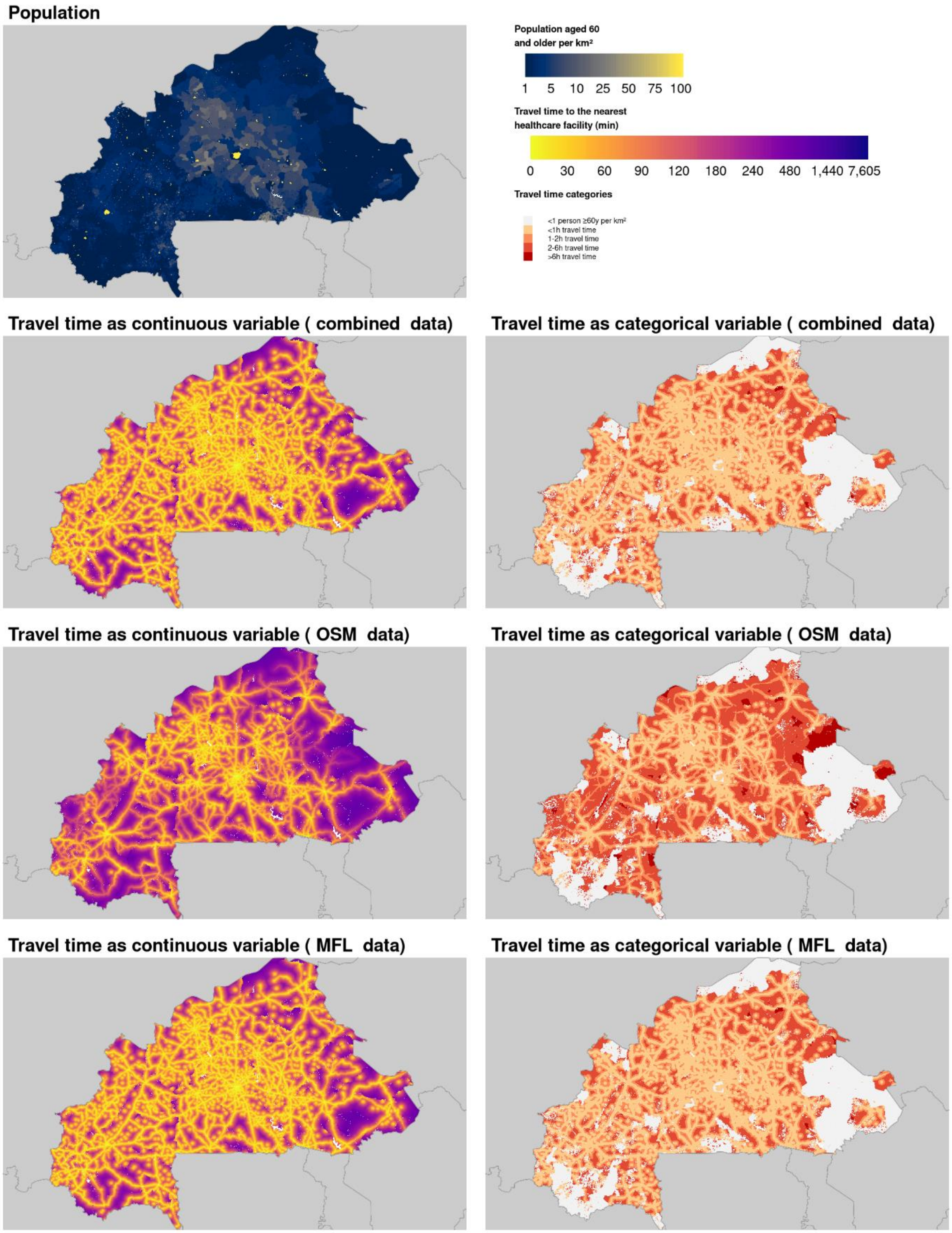

Figure S57. Burundi map of travel time to the nearest healthcare facility for adults aged ≥ 60 years

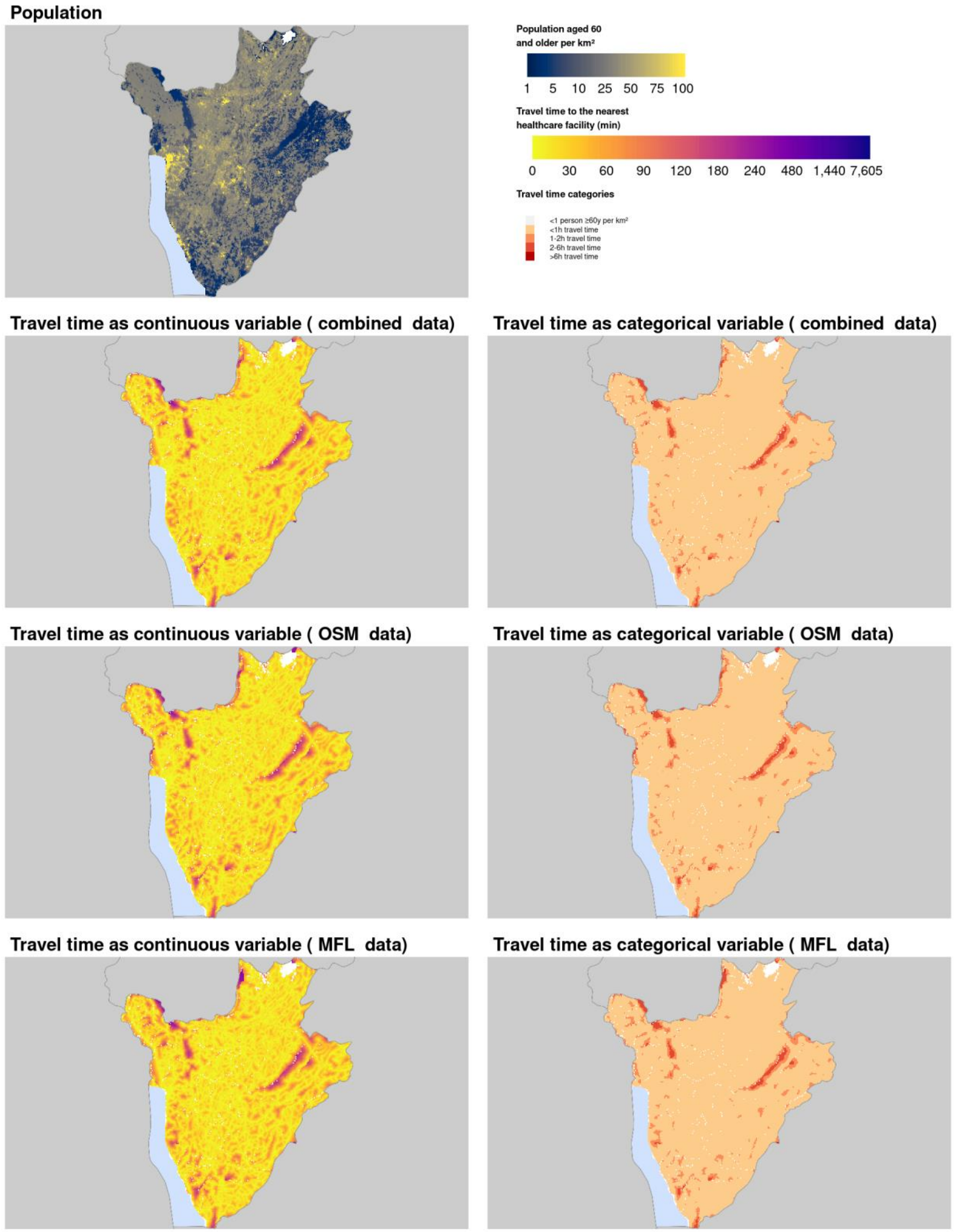

Figure S58. Cameroon map of travel time to the nearest healthcare facility for adults aged ≥ 60 years

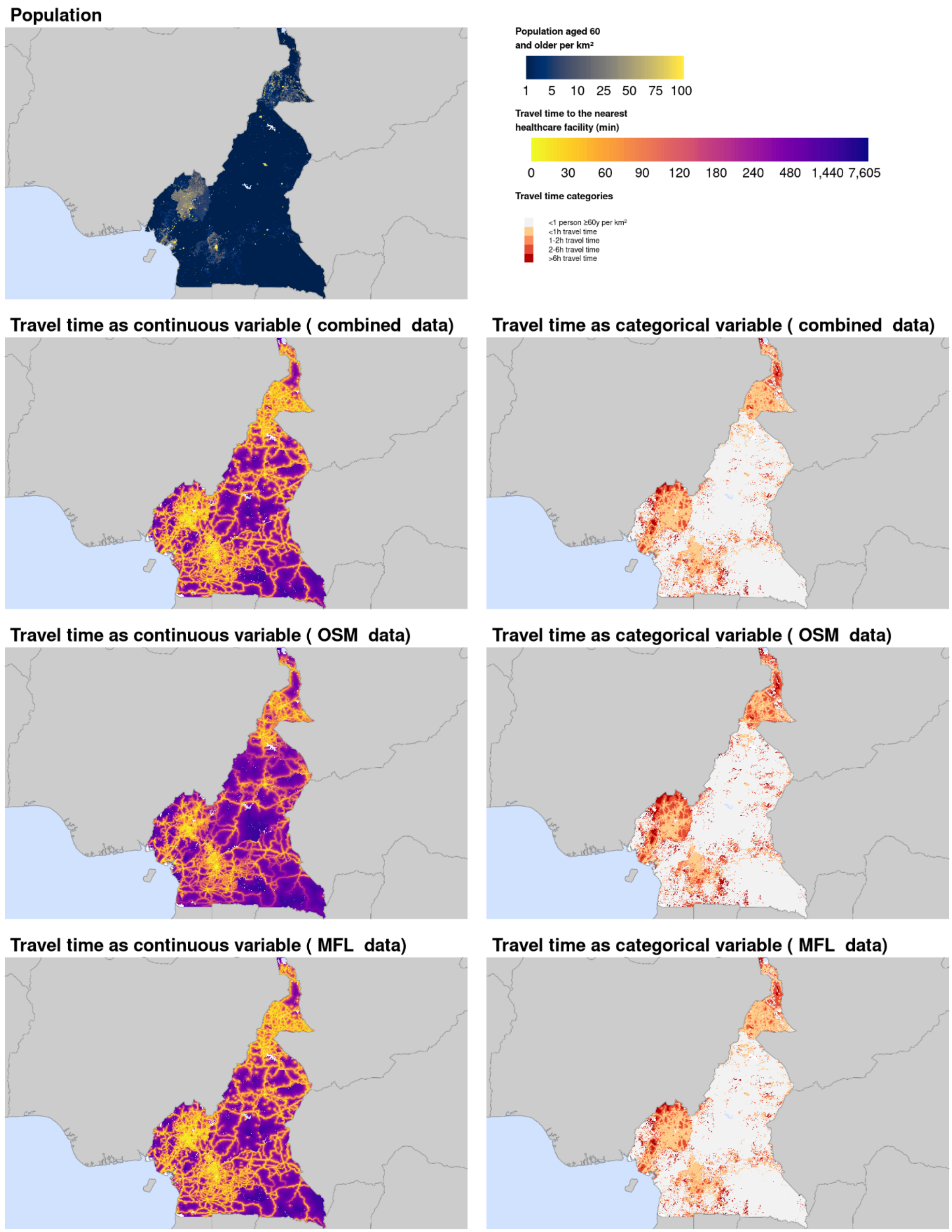

Figure S59. Central African Republic map of travel time to the nearest healthcare facility for adults aged ≥ 60 years

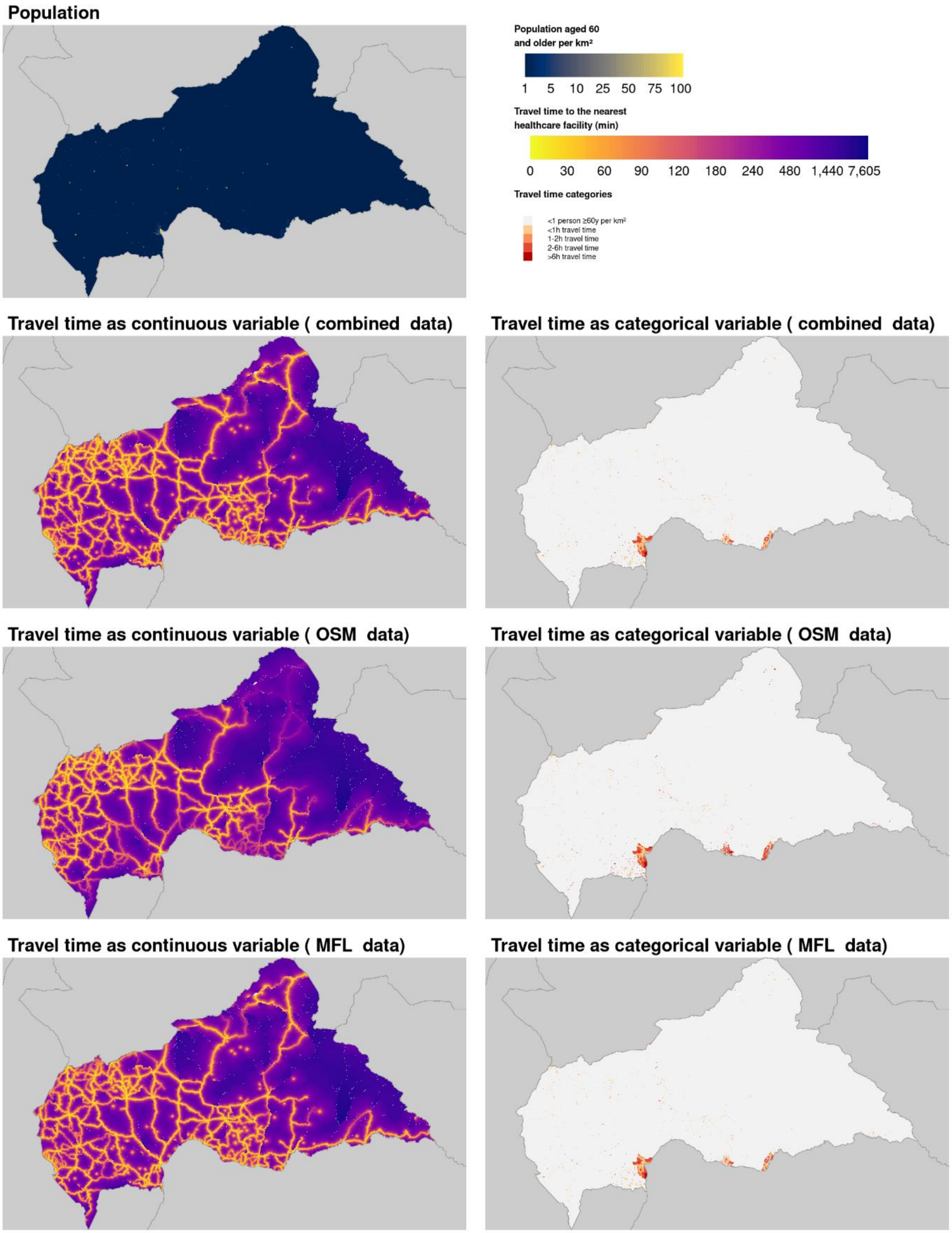

Figure S60. Chad map of travel time to the nearest healthcare facility for adults aged ≥ 60 years

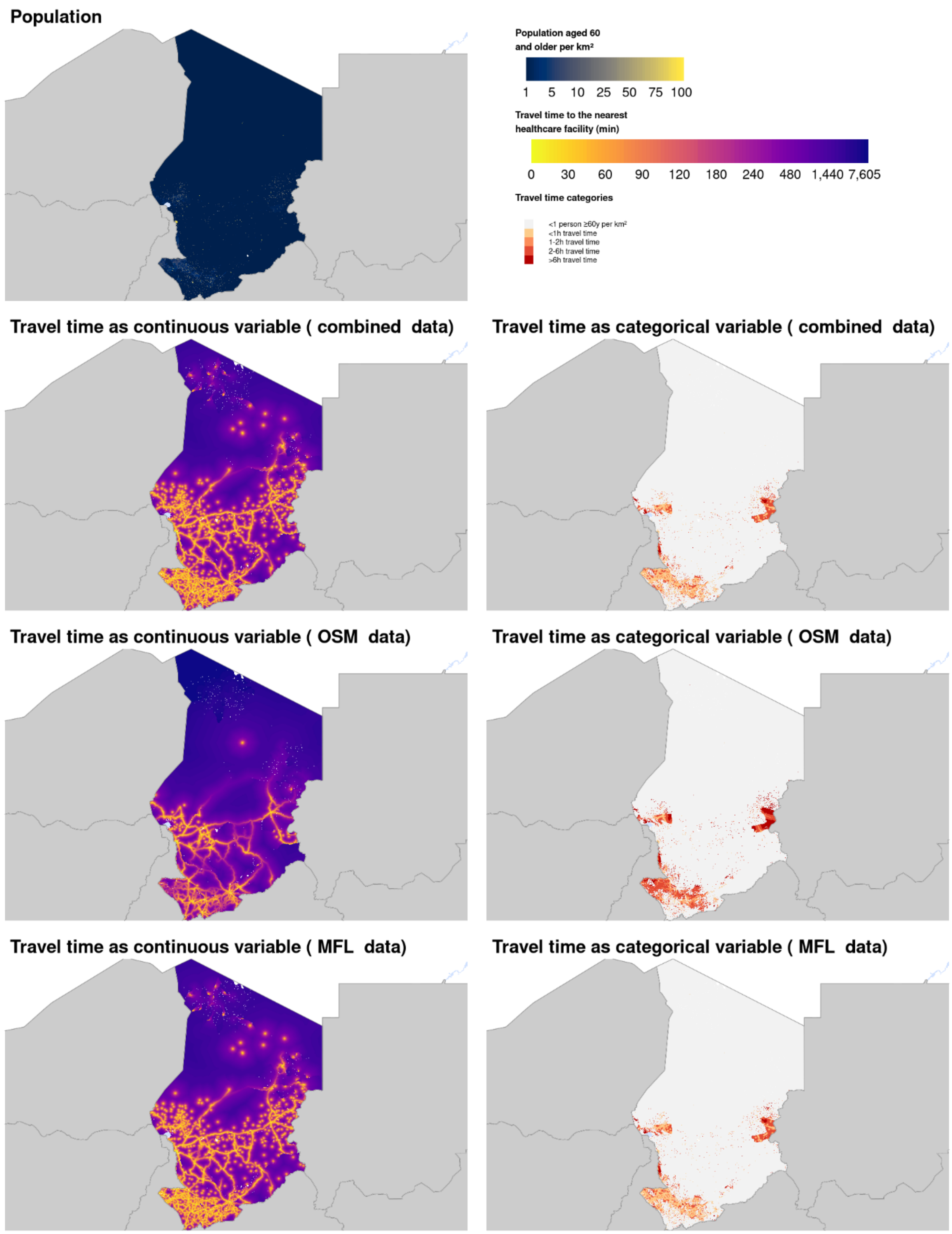

Figure S61. Djibouti map of travel time to the nearest healthcare facility for adults aged ≥ 60 years

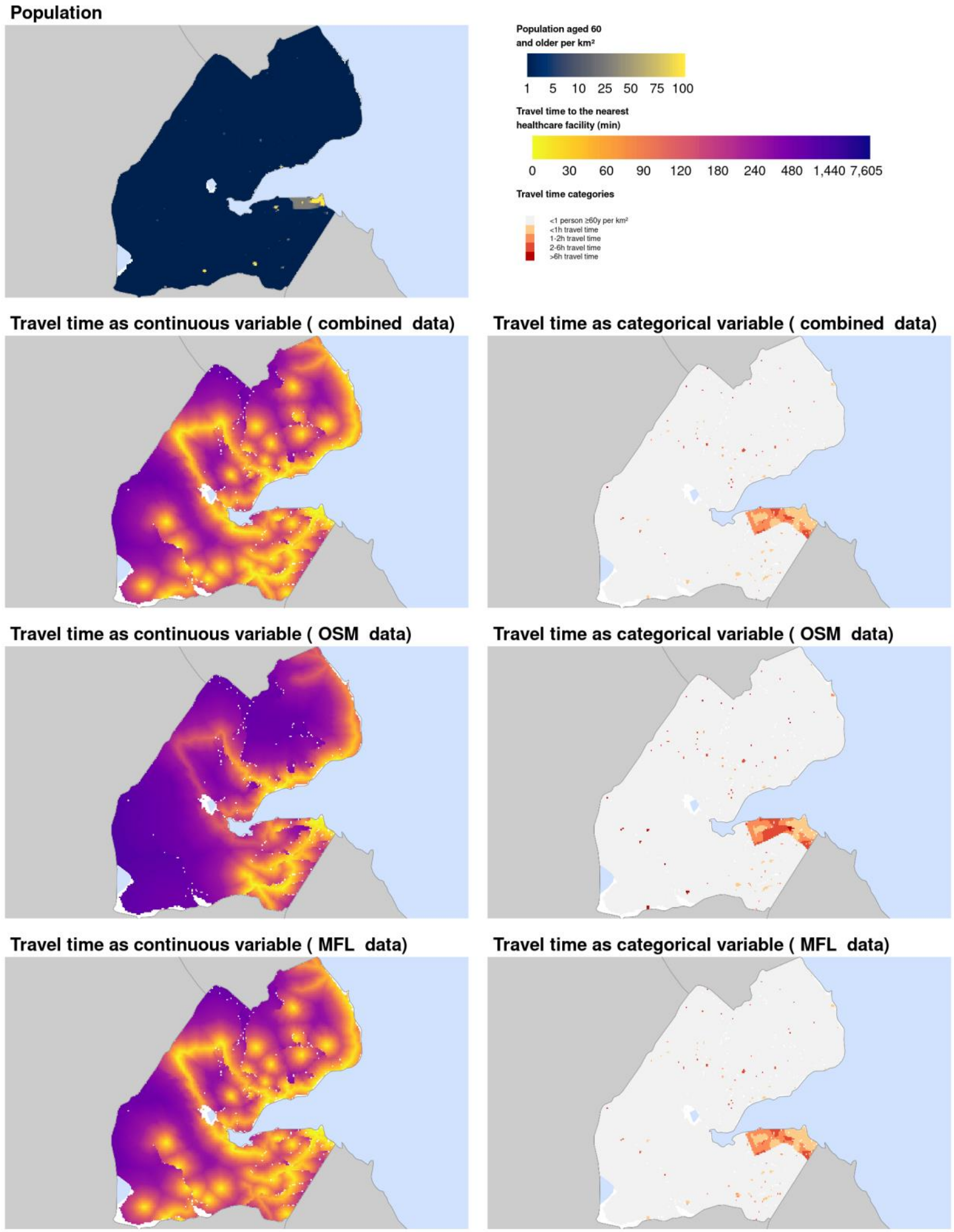

Figure S62. DRC map of travel time to the nearest healthcare facility for adults aged ≥ 60 years

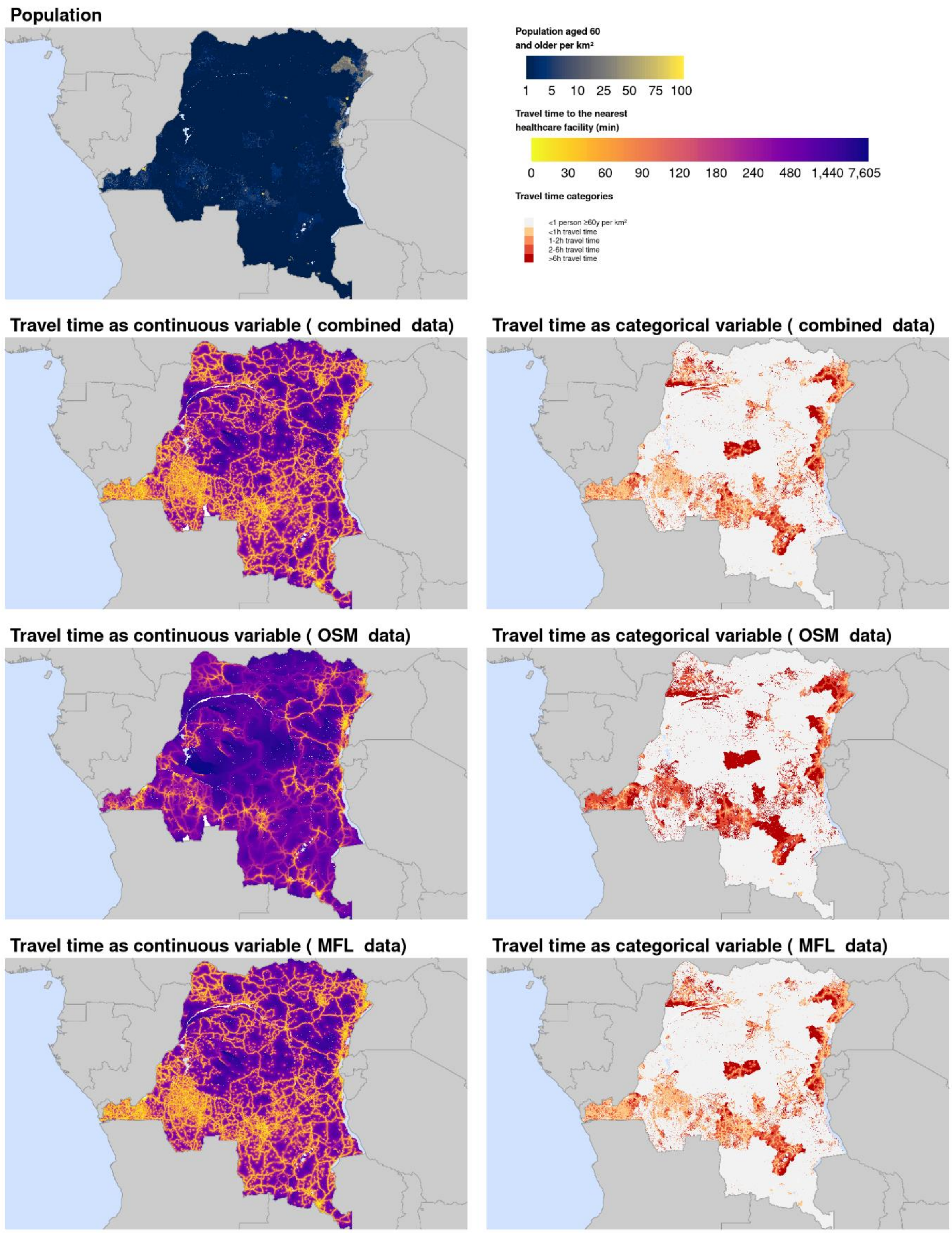

Figure S63. Equatorial Guinea map of travel time to the nearest healthcare facility for adults aged ≥ 60 years

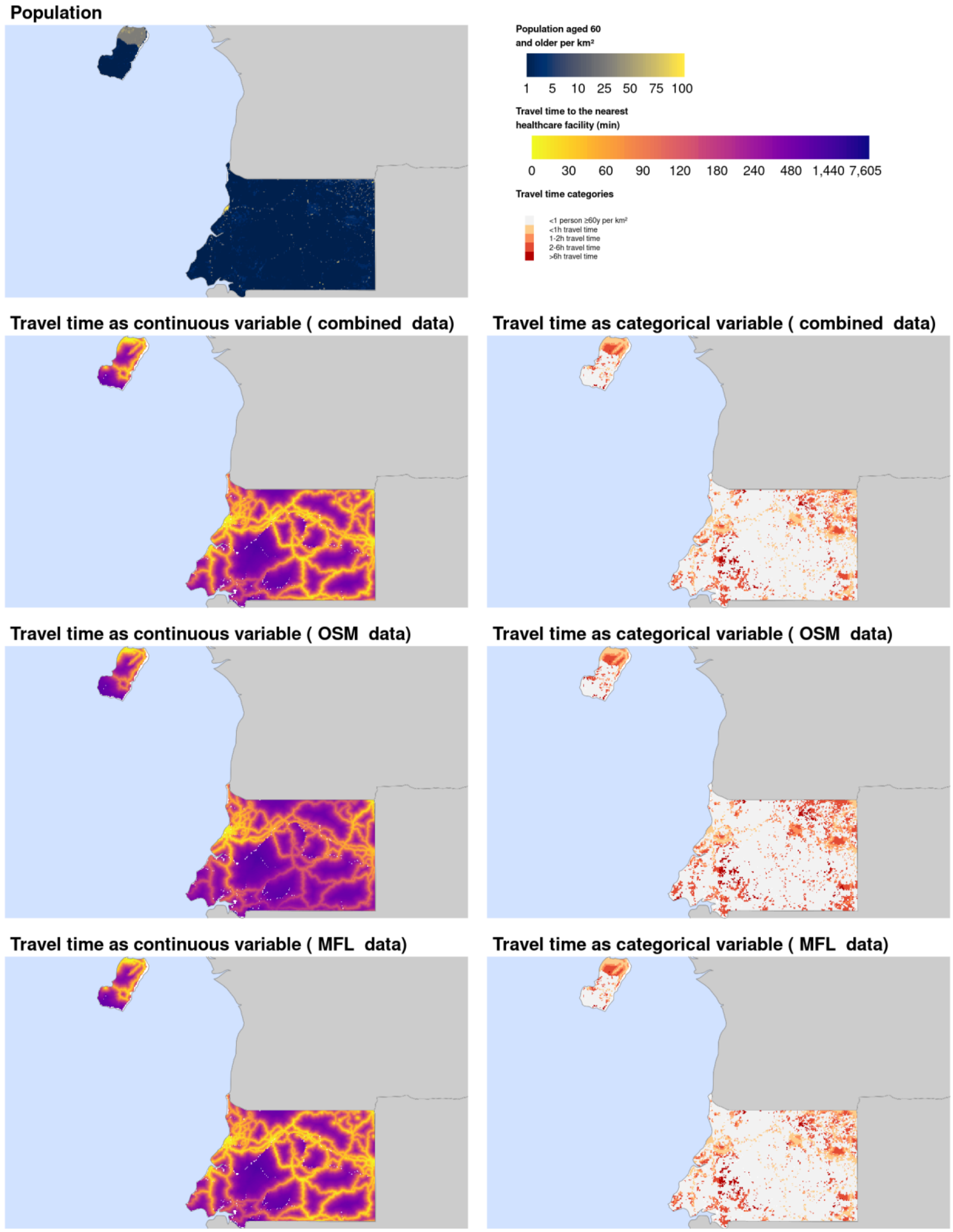

Figure S64. Eritrea map of travel time to the nearest healthcare facility for adults aged ≥ 60 years

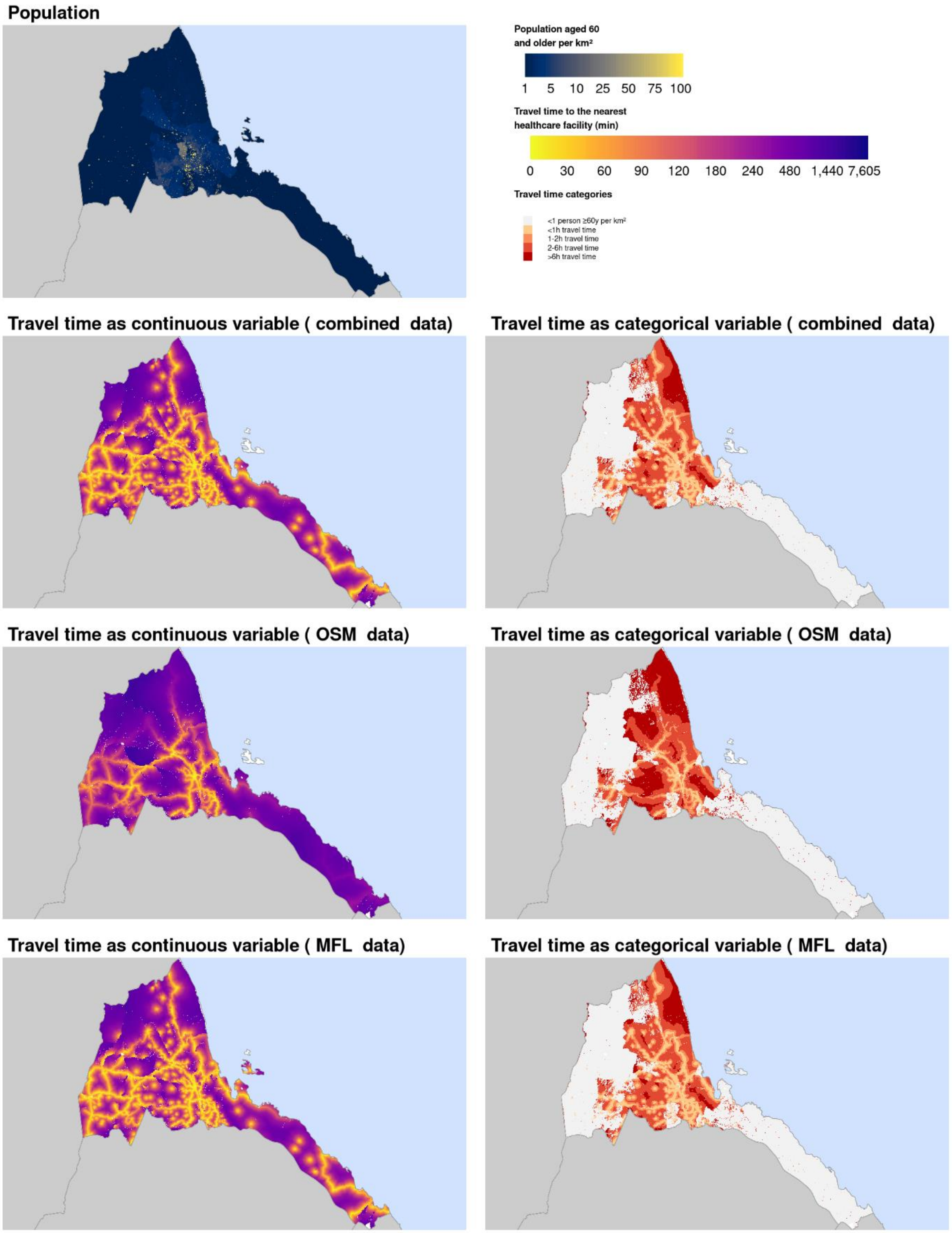

Figure S65. eSwatini map of travel time to the nearest healthcare facility for adults aged ≥ 60 years

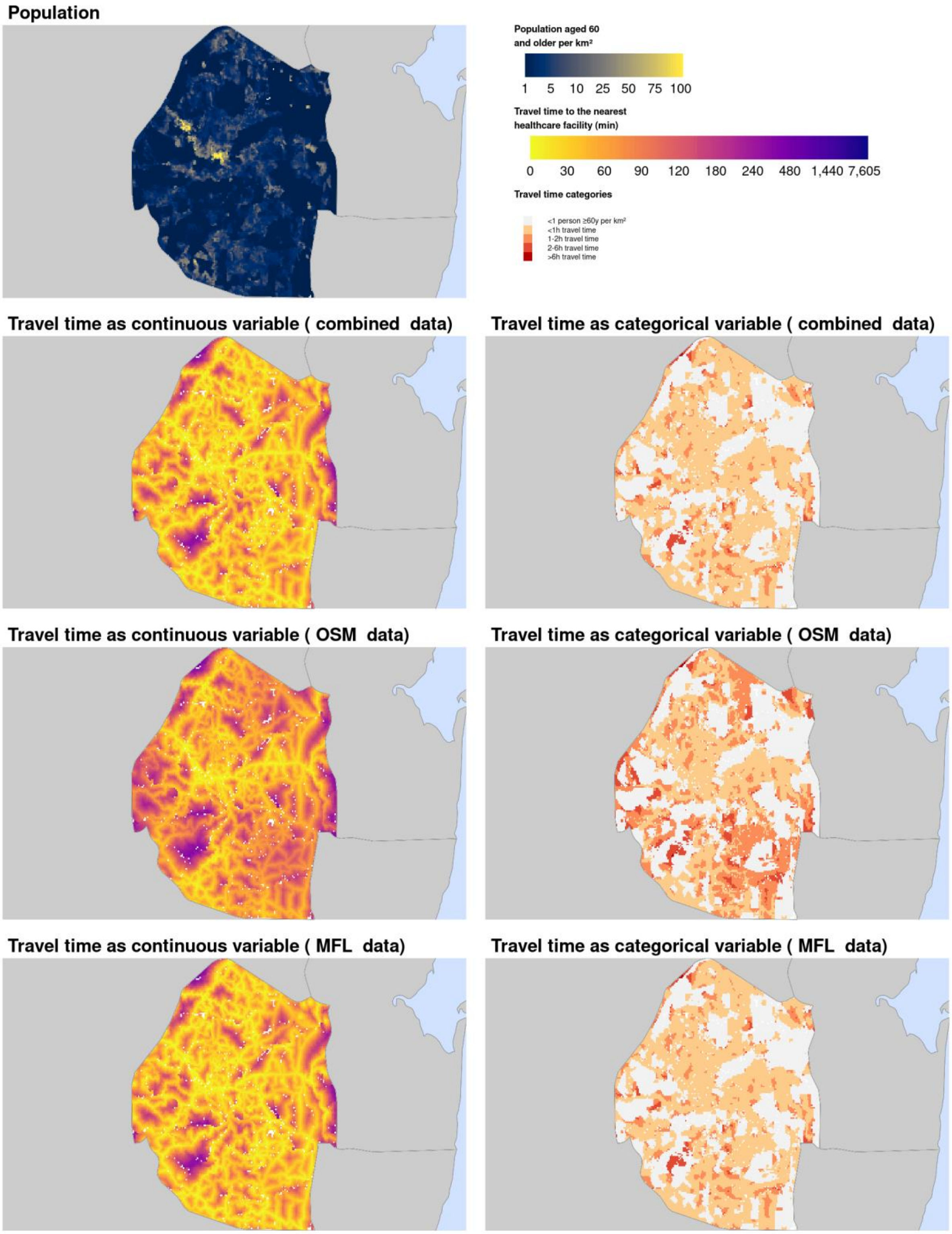

Figure S66. Ethiopia map of travel time to the nearest healthcare facility for adults aged ≥ 60 years

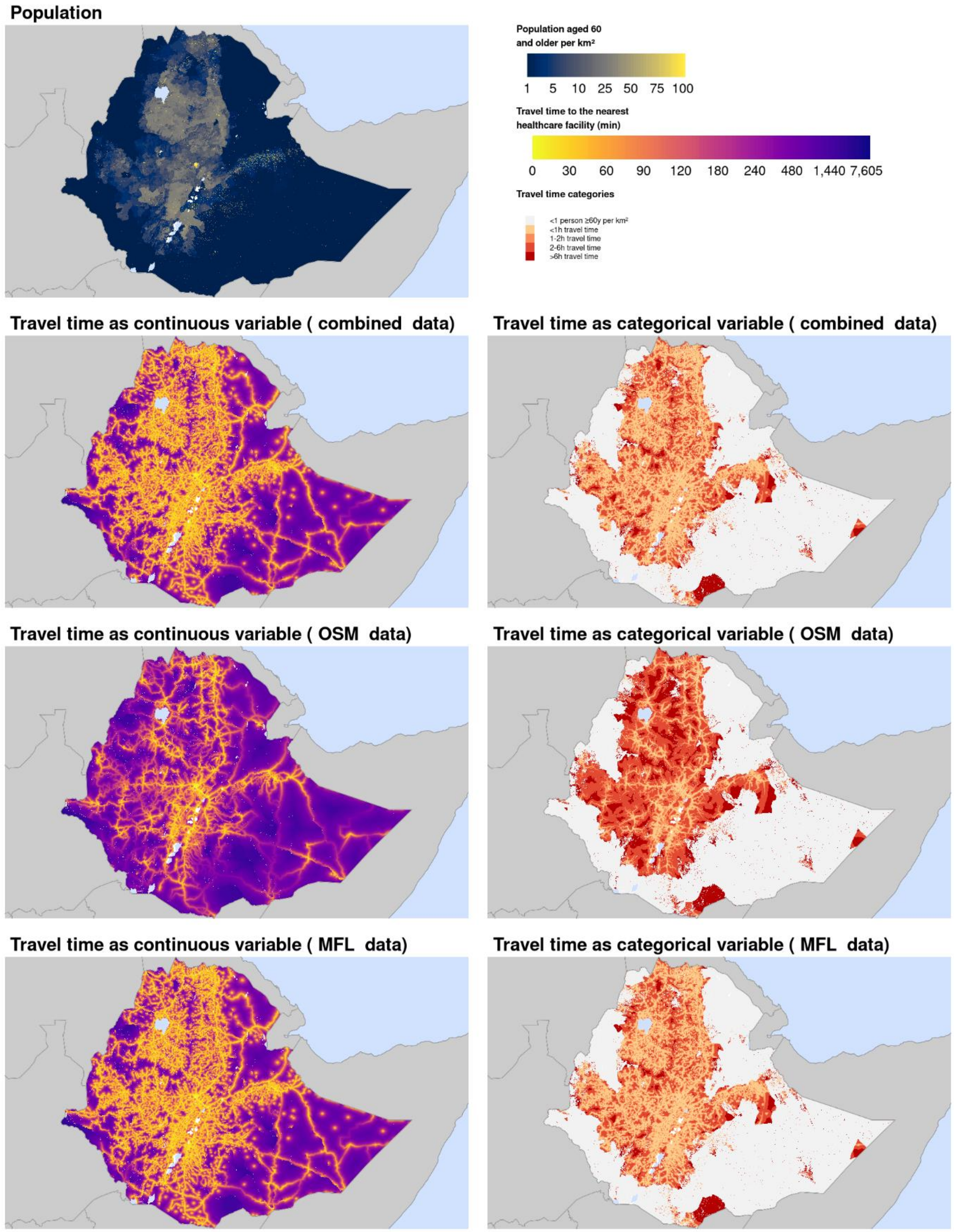

Figure S67. Gabon map of travel time to the nearest healthcare facility for adults aged ≥ 60 years

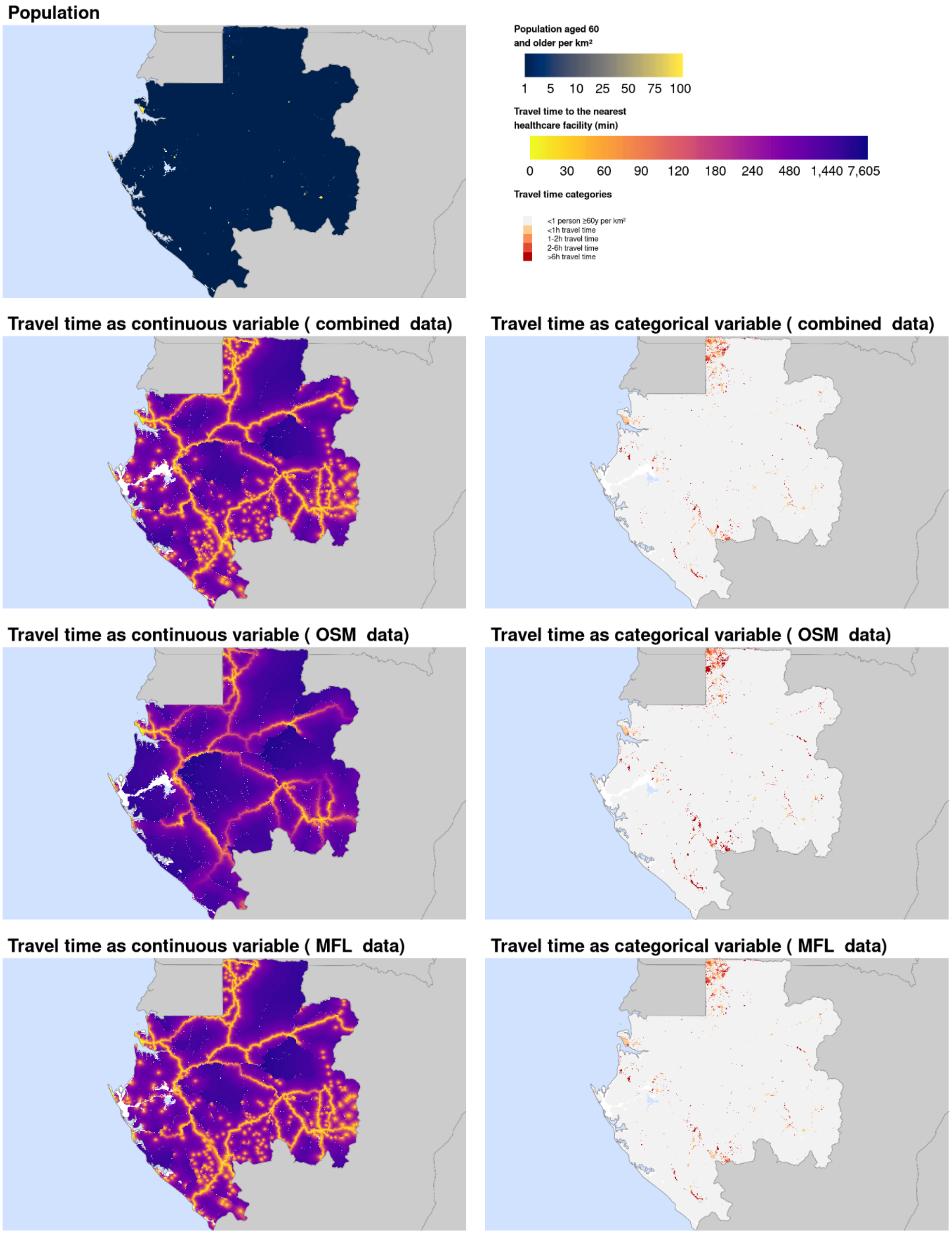

Figure S68. Ghana map of travel time to the nearest healthcare facility for adults aged ≥ 60 years

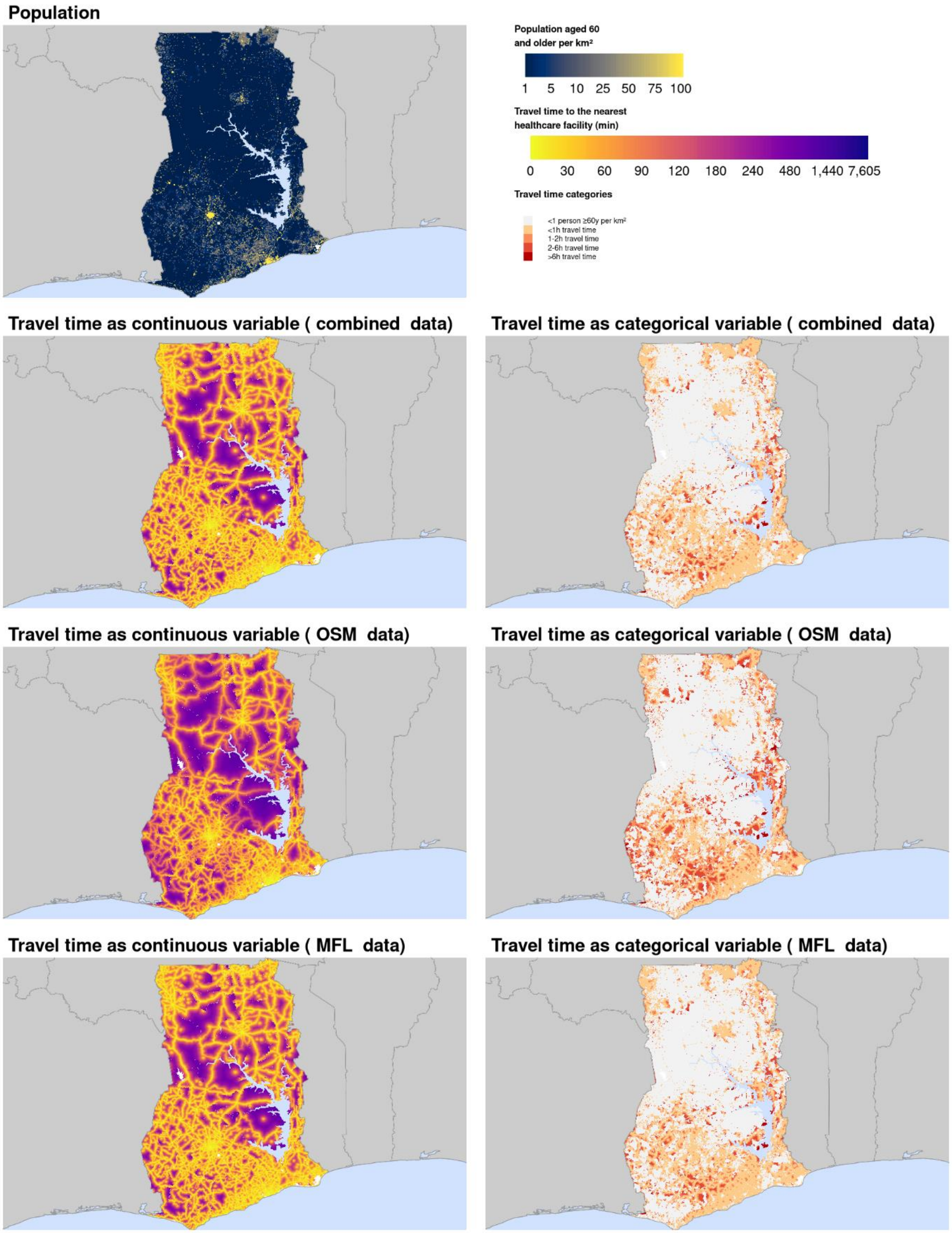

Figure S69. Guinea map of travel time to the nearest healthcare facility for adults aged ≥ 60 years

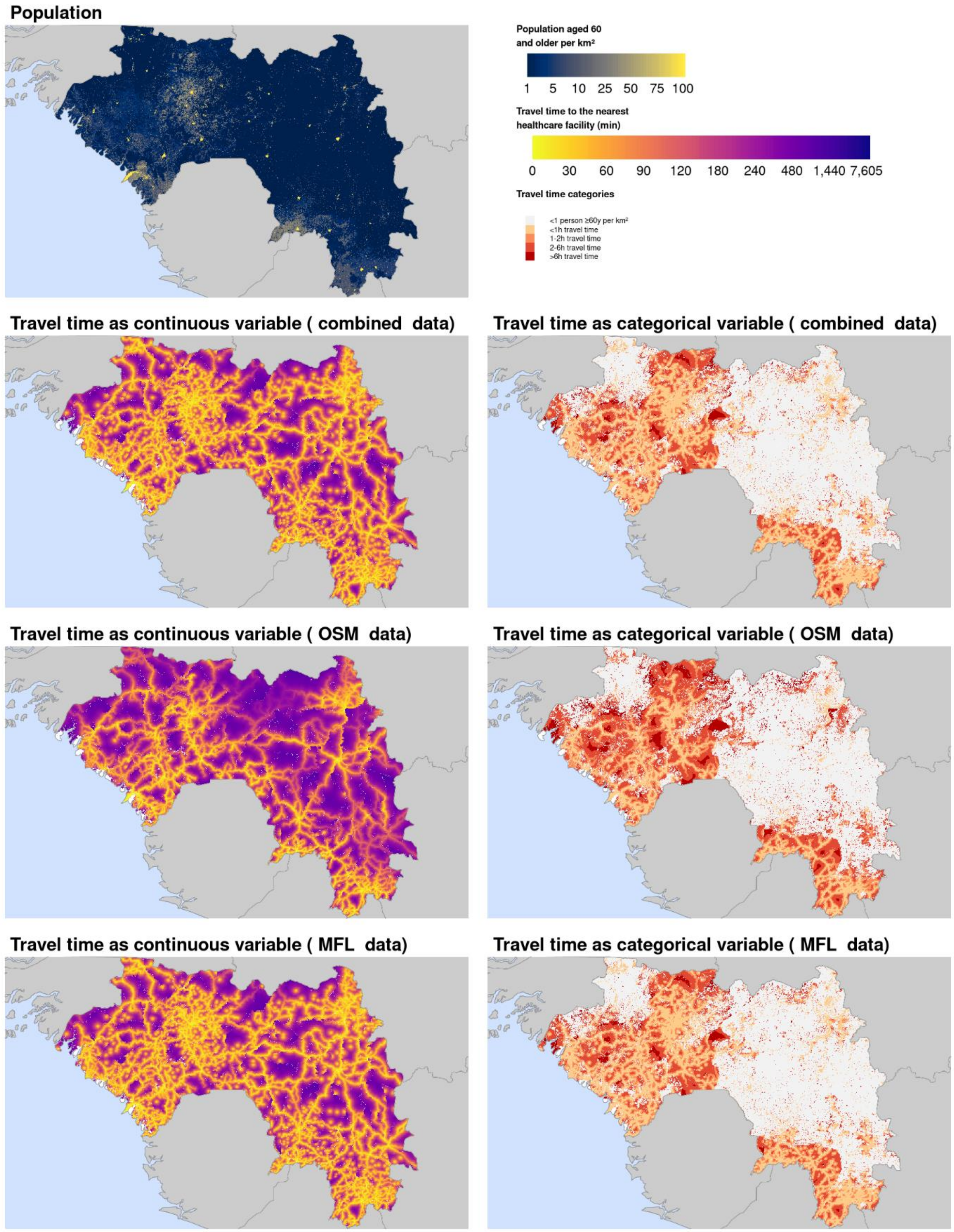

Figure S70. Guinea-Bissau map of travel time to the nearest healthcare facility for adults aged ≥ 60 years

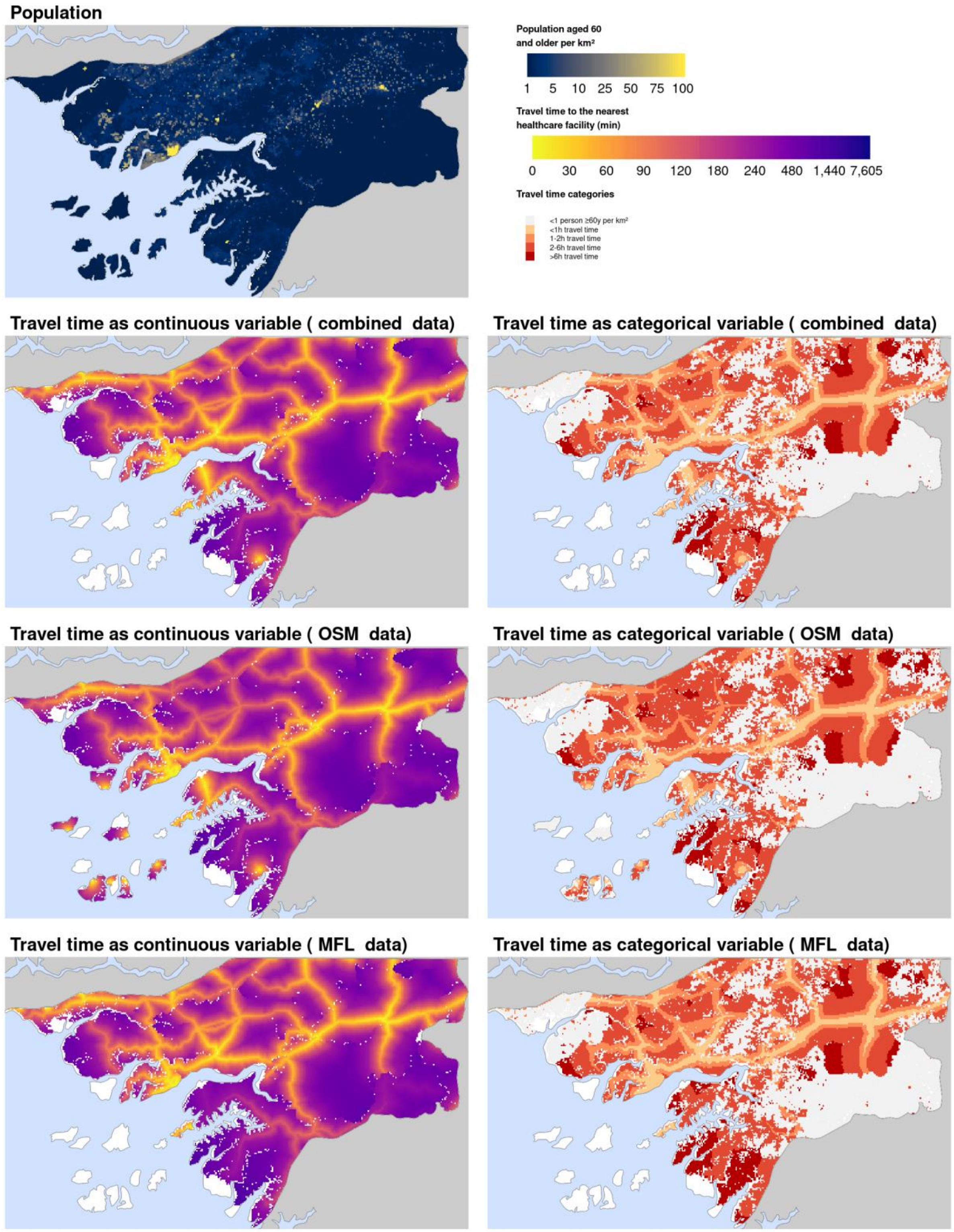

Figure S71. Ivory Coast map of travel time to the nearest healthcare facility for adults aged ≥ 60 years

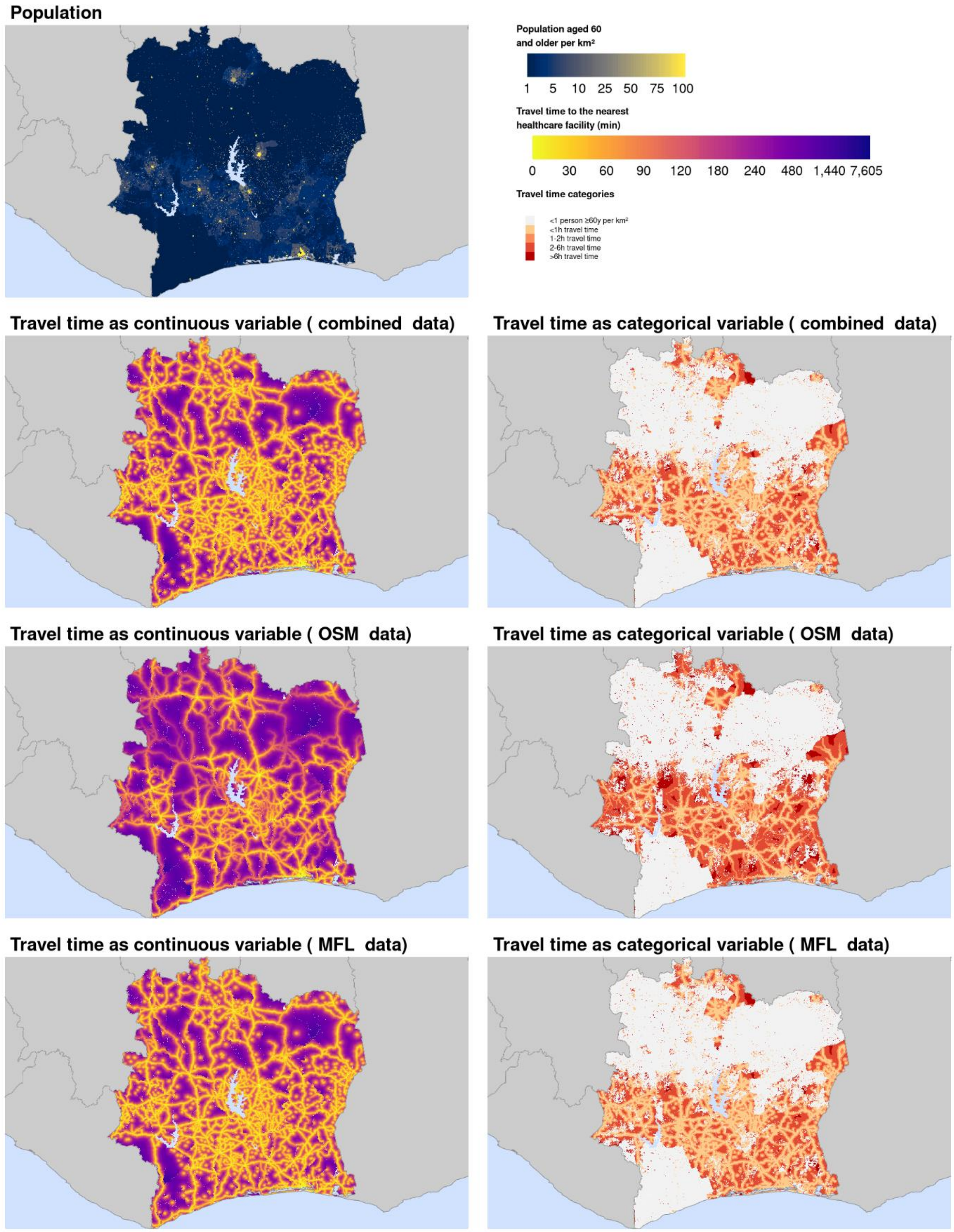

Figure S72. Kenya map of travel time to the nearest healthcare facility for adults aged ≥ 60 years

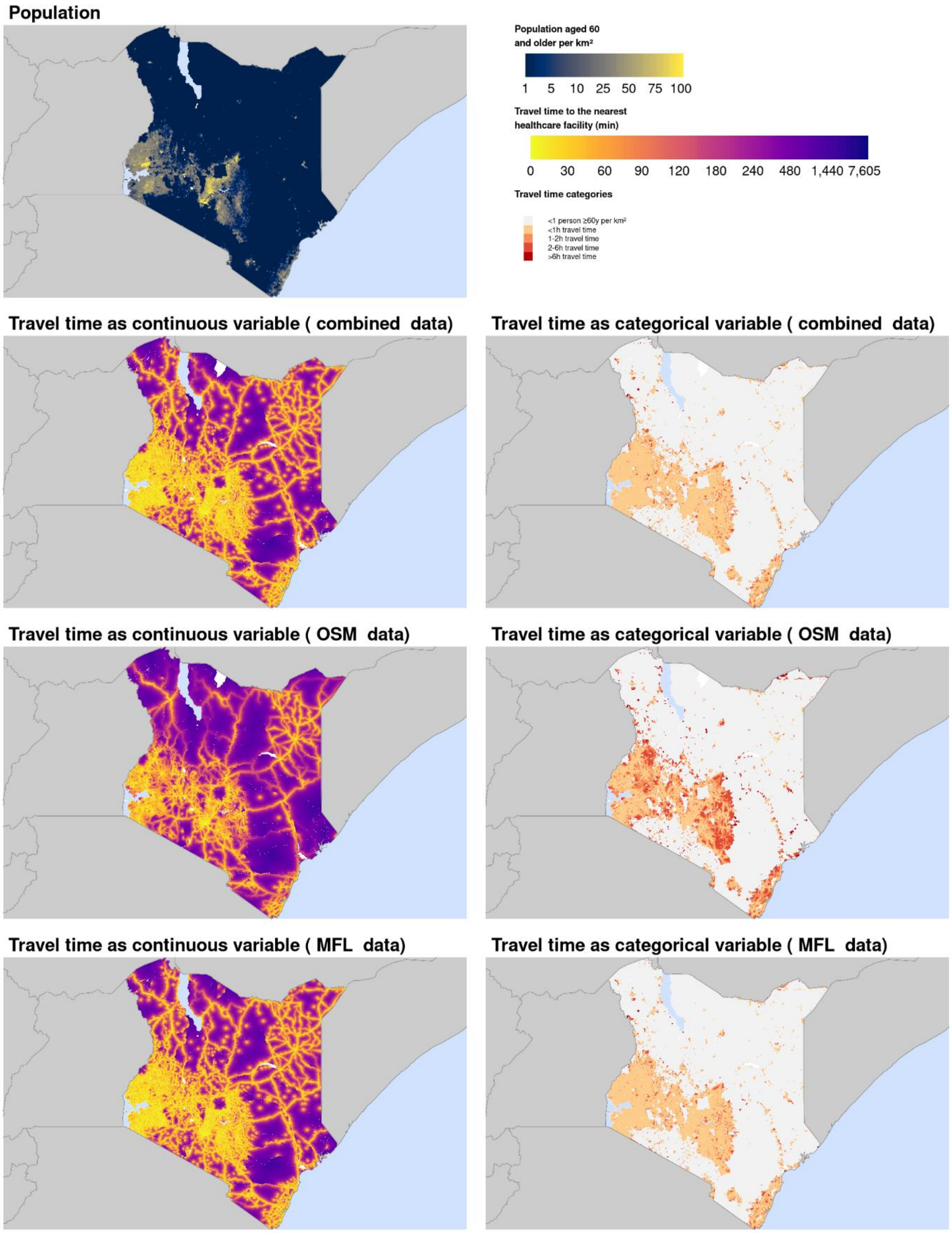

Figure S73. Lesotho map of travel time to the nearest healthcare facility for adults aged ≥ 60 years

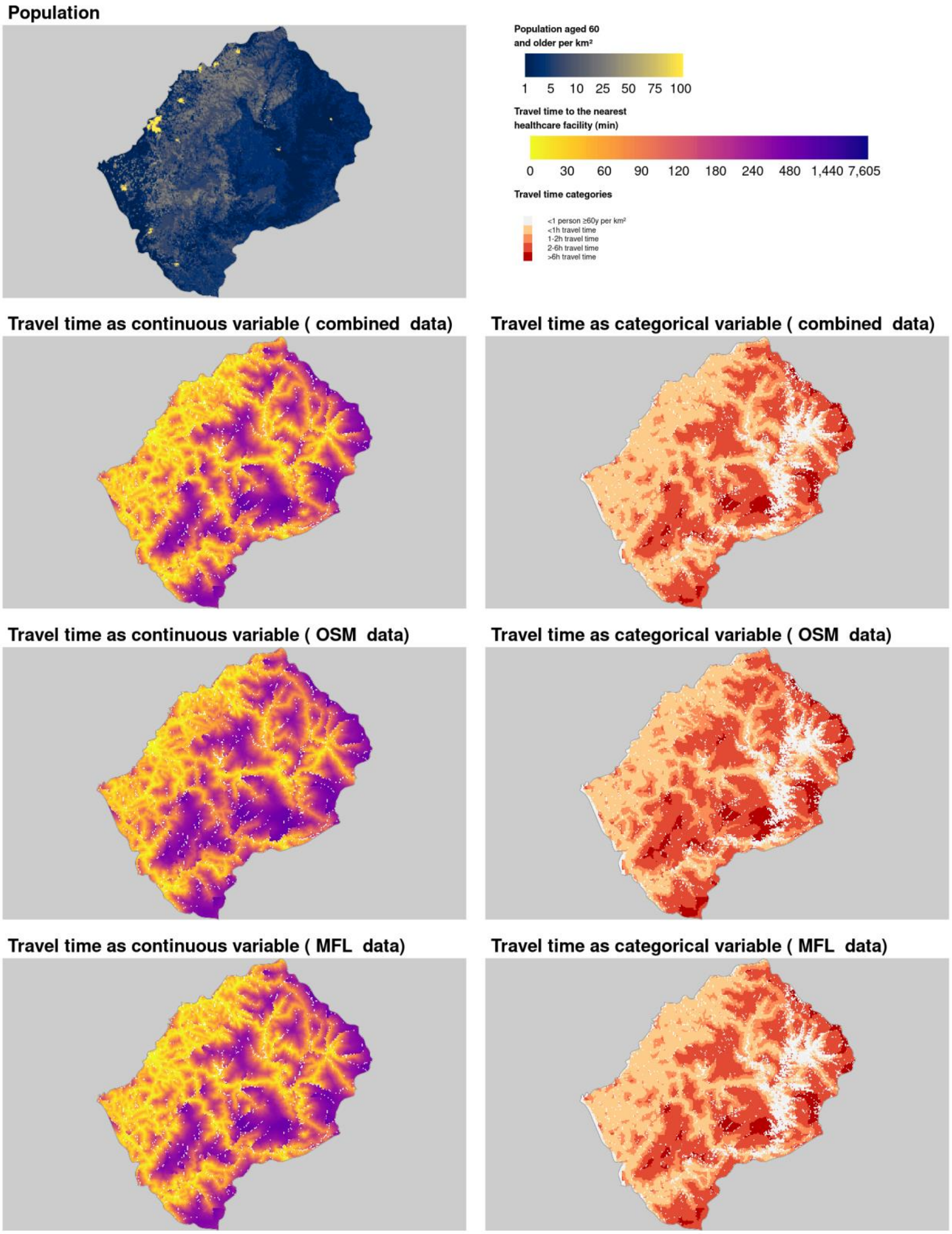

Figure S74. Liberia map of travel time to the nearest healthcare facility for adults aged ≥ 60 years

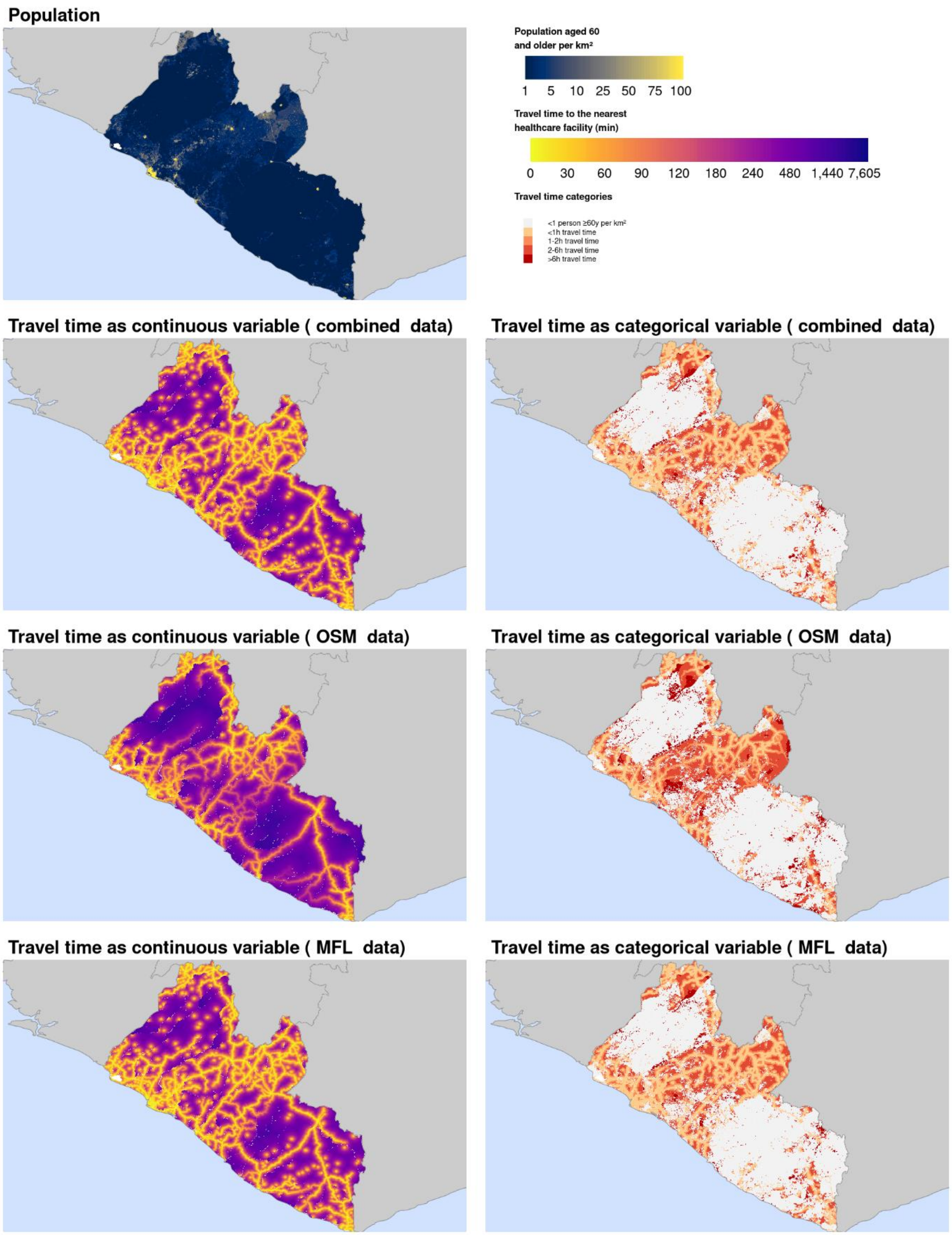

Figure S75. Madagascar map of travel time to the nearest healthcare facility for adults aged ≥ 60 years

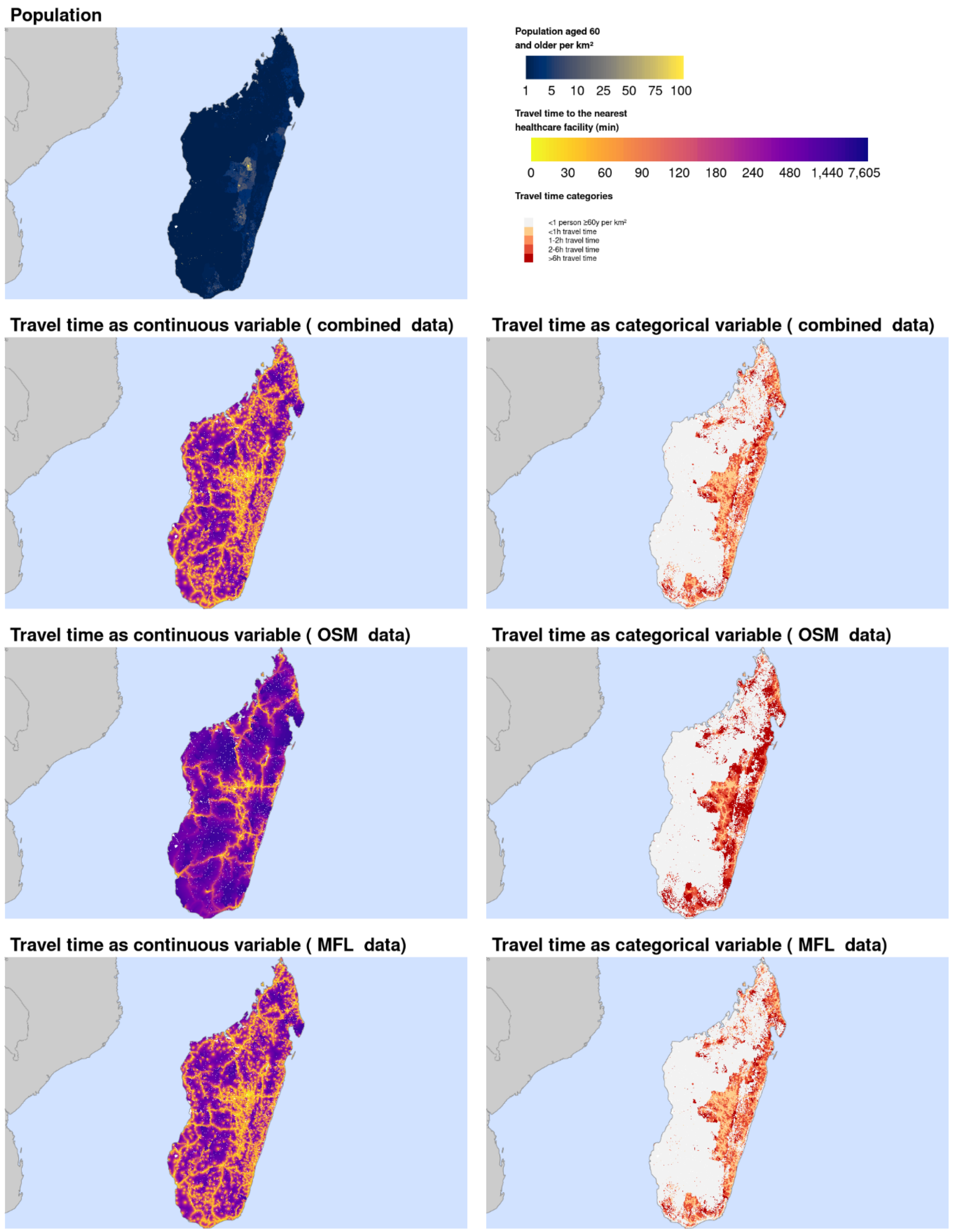

Figure S76. Malawi map of travel time to the nearest healthcare facility for adults aged ≥ 60 years

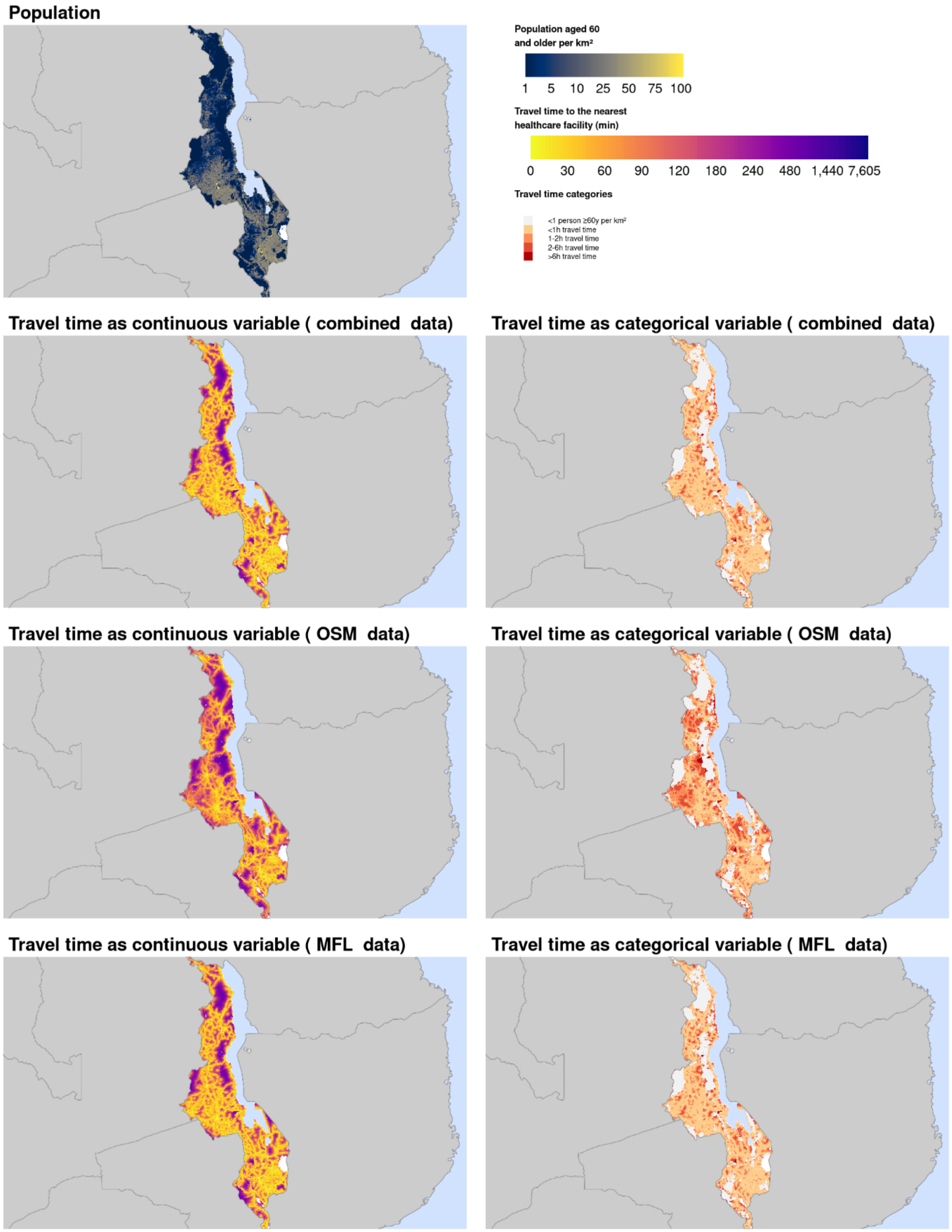

Figure S77. Mali map of travel time to the nearest healthcare facility for adults aged ≥ 60 years

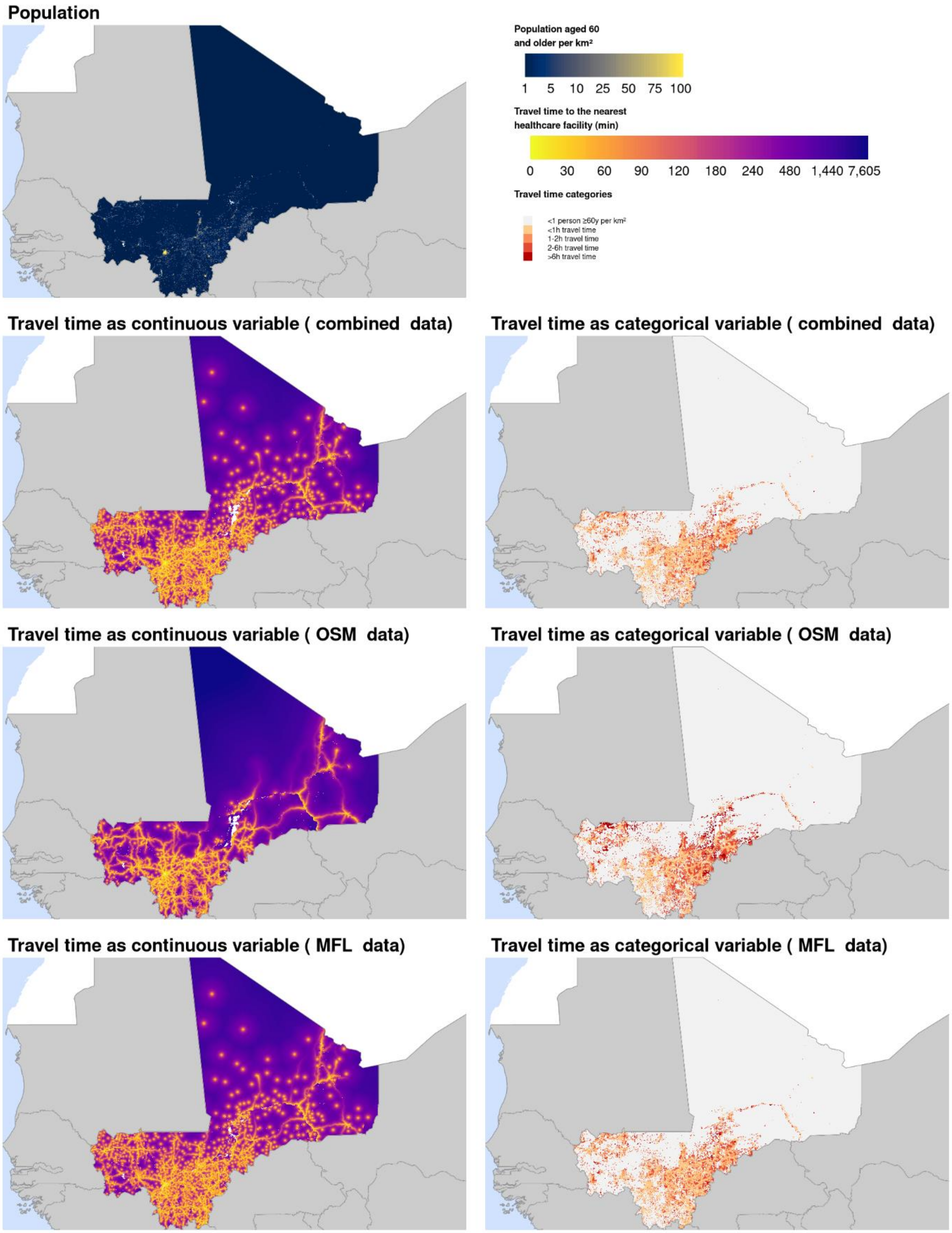

Figure S78. Mauritania map of travel time to the nearest healthcare facility for adults aged ≥ 60 years

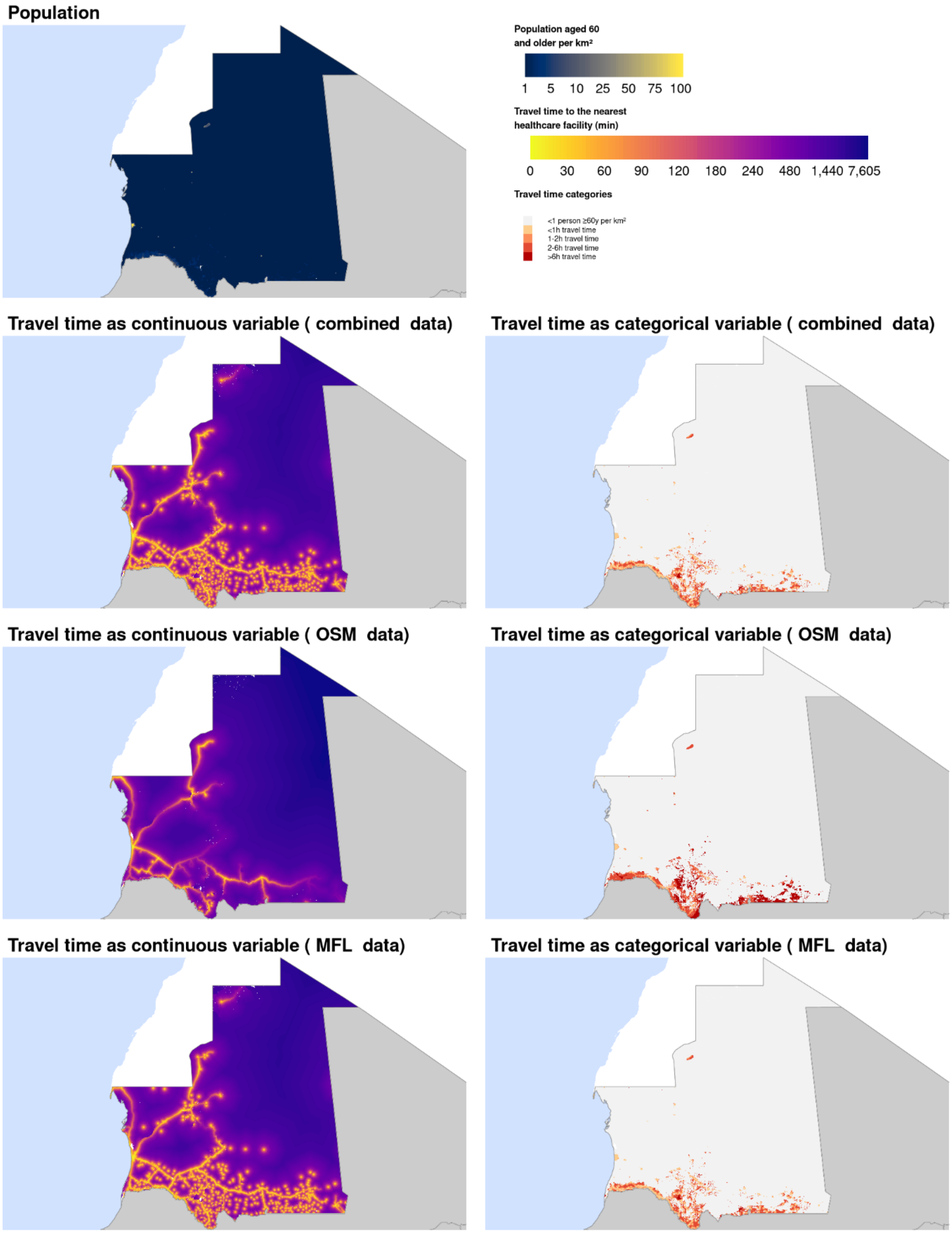

Figure S79. Mozambique map of travel time to the nearest healthcare facility for adults aged ≥ 60 years

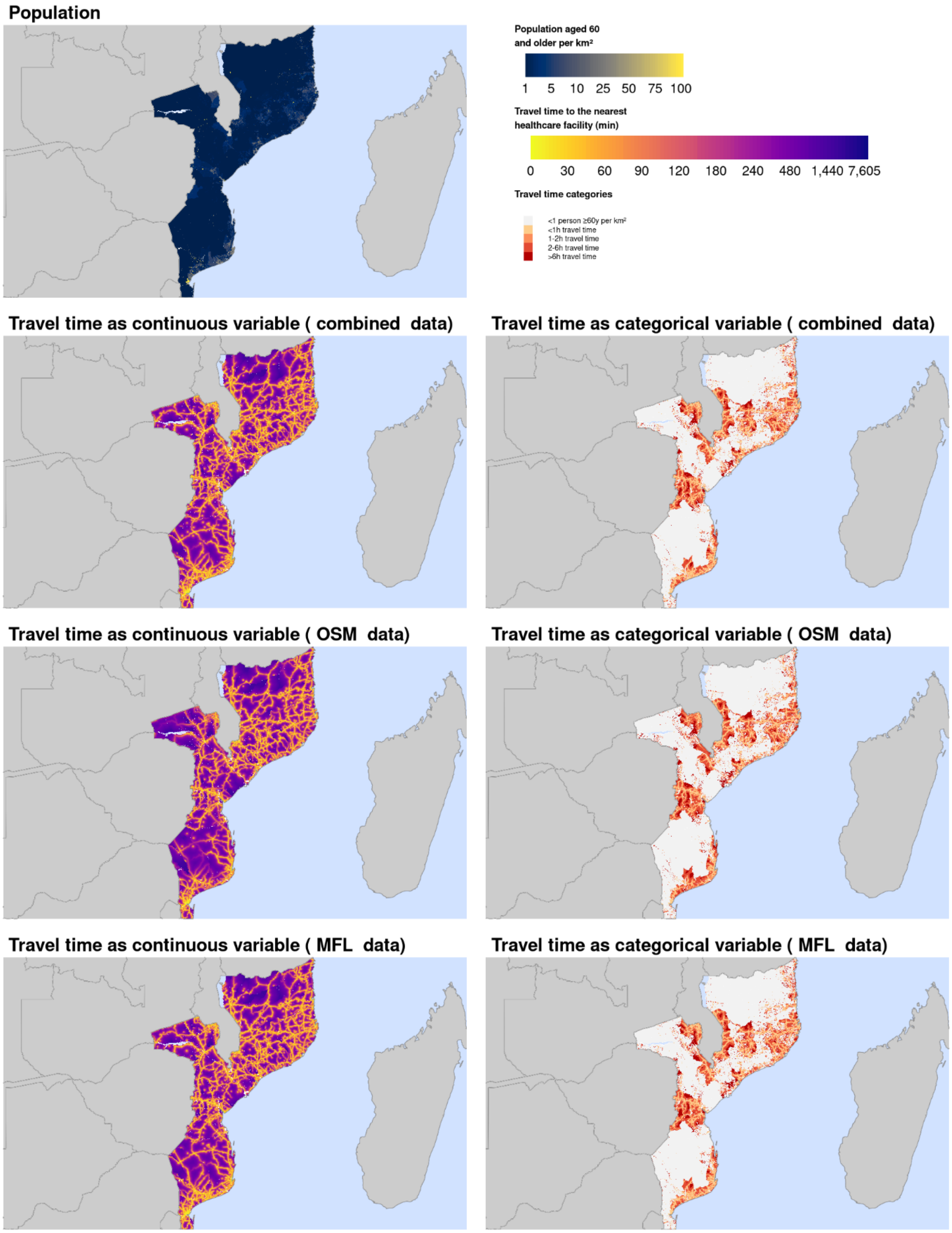

Figure S80. Namibia map of travel time to the nearest healthcare facility for adults aged ≥ 60 years

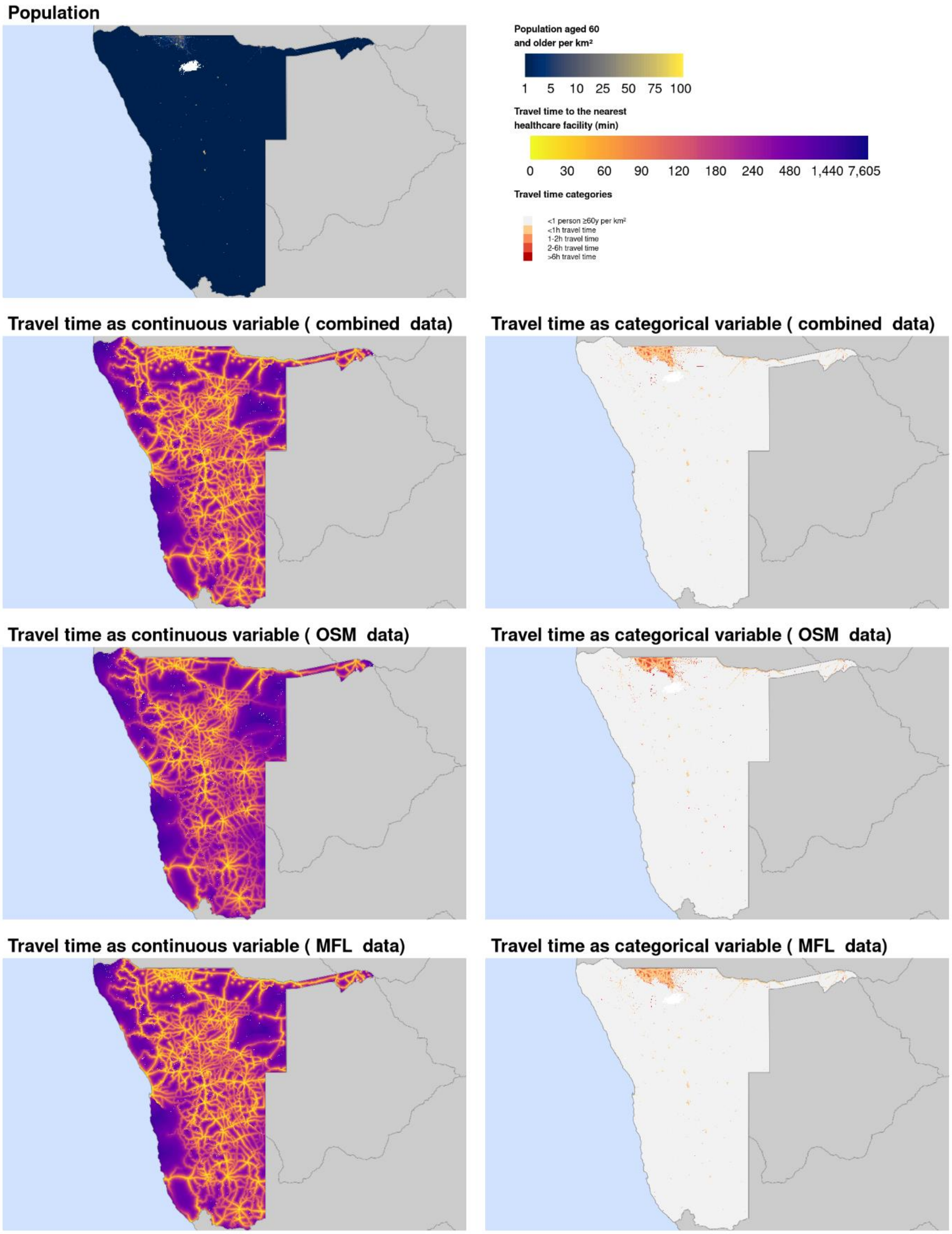

Figure S81. Niger map of travel time to the nearest healthcare facility for adults aged ≥ 60 years

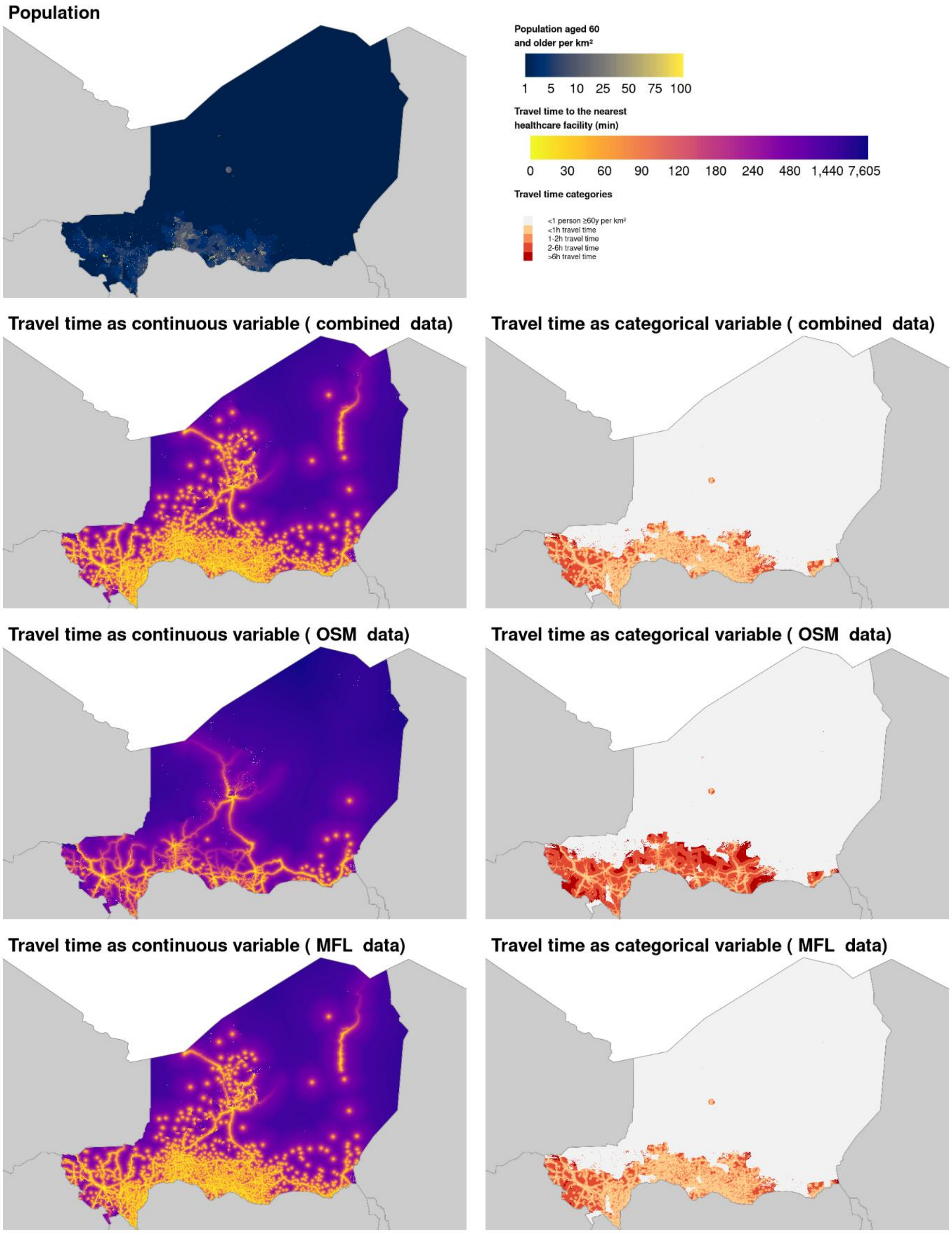

Figure S82. Nigeria map of travel time to the nearest healthcare facility for adults aged ≥ 60 years

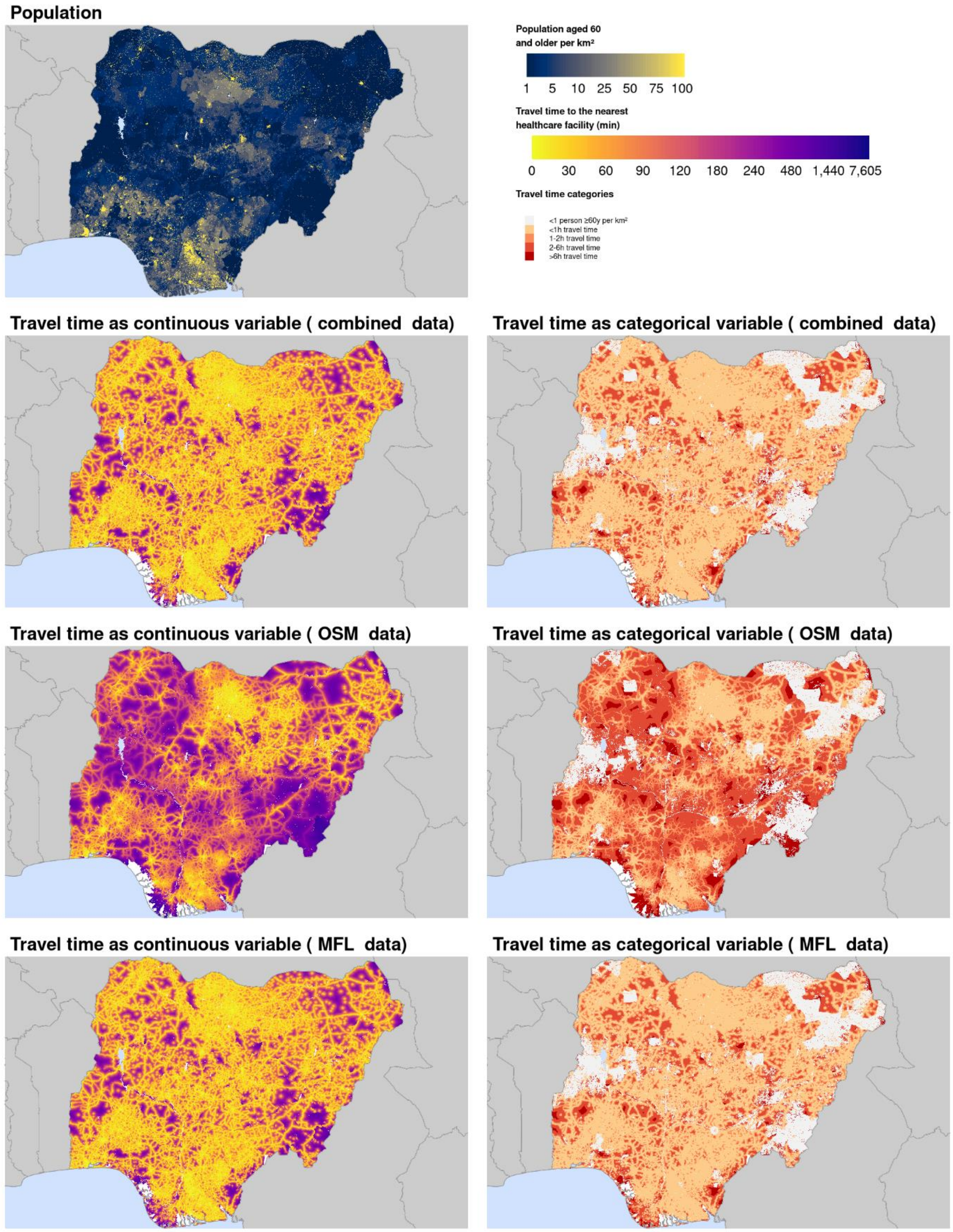

Figure S83. Republic of the Congo map of travel time to the nearest healthcare facility for adults aged ≥ 60 years

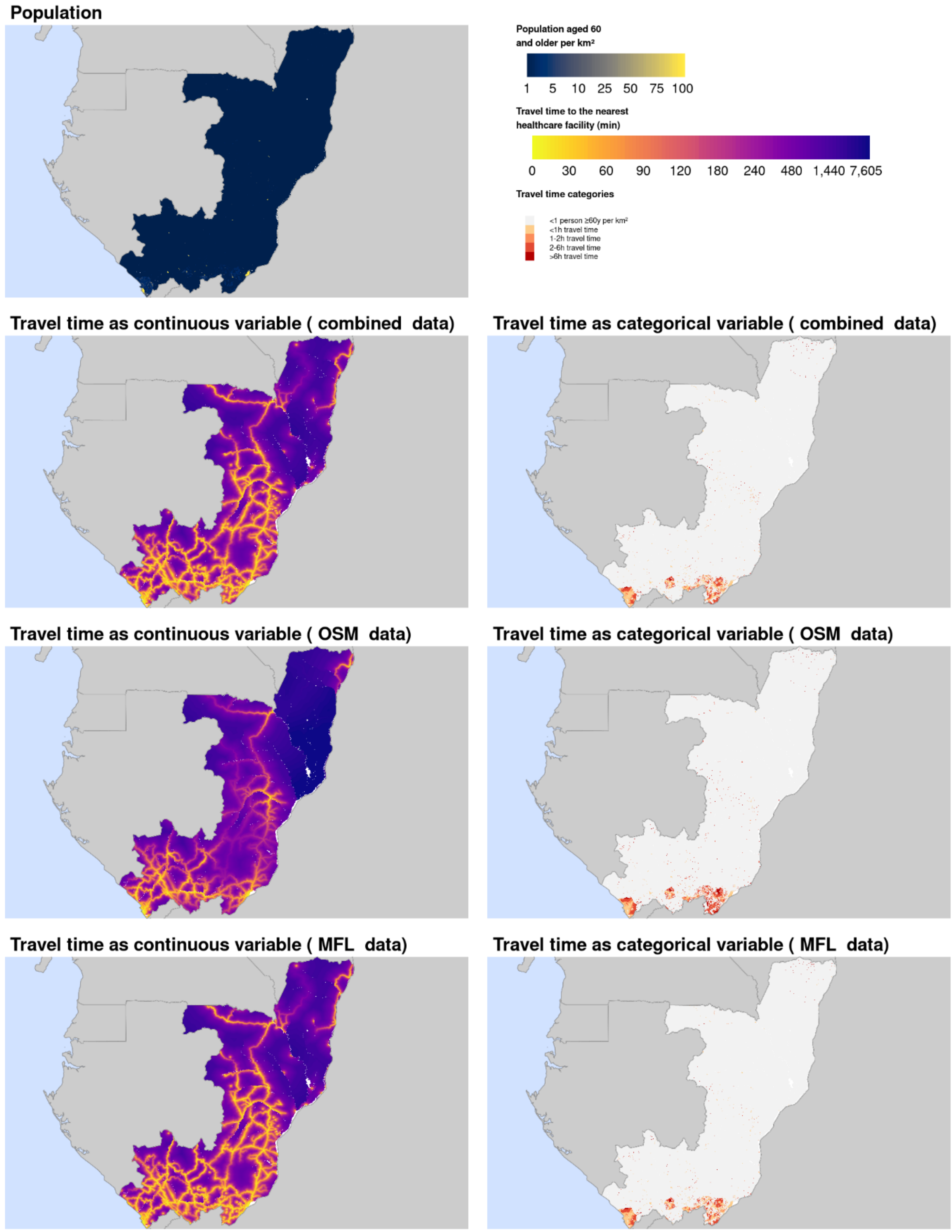

Figure S84. Rwanda map of travel time to the nearest healthcare facility for adults aged ≥ 60 years

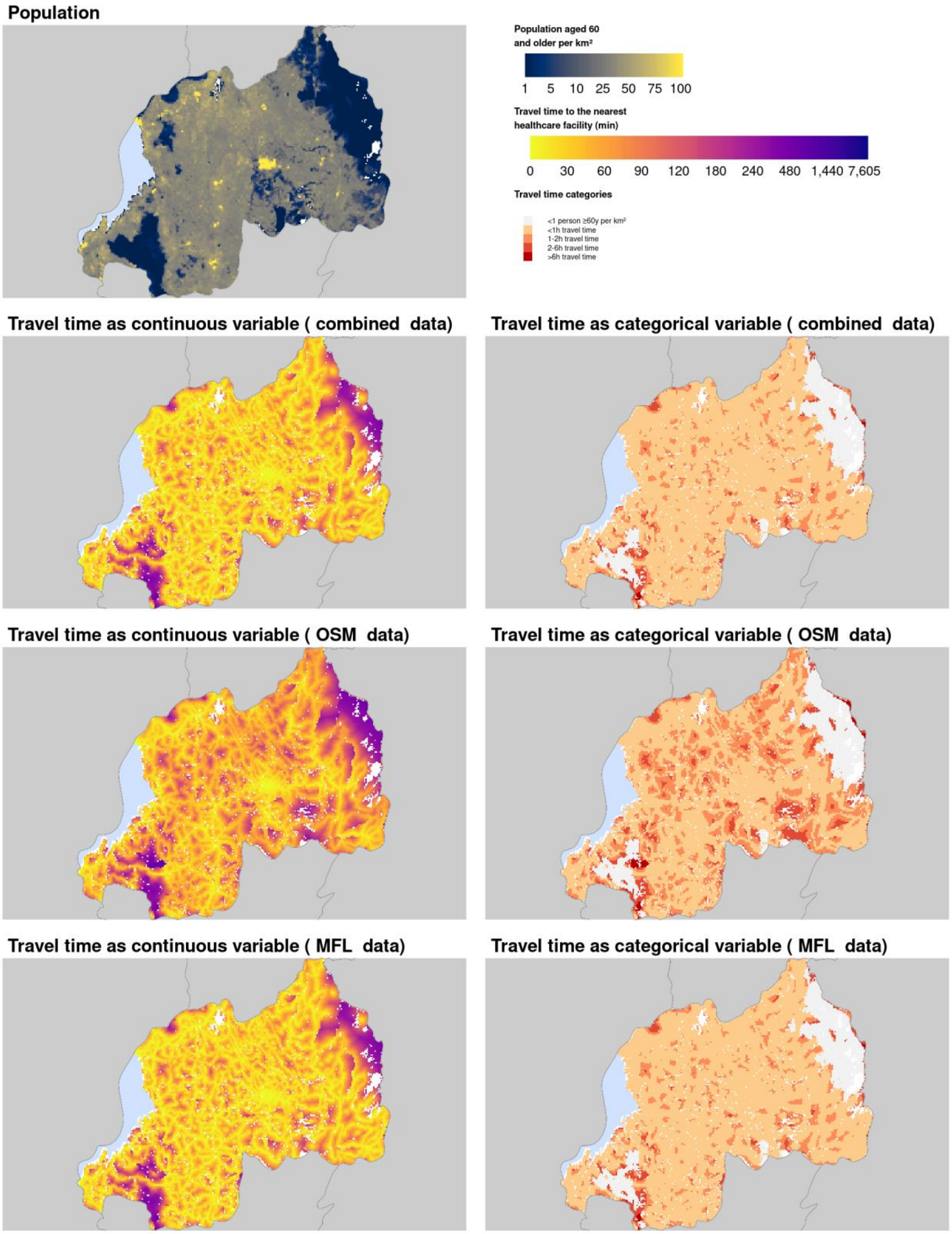

Figure S85. Senegal map of travel time to the nearest healthcare facility for adults aged ≥ 60 years

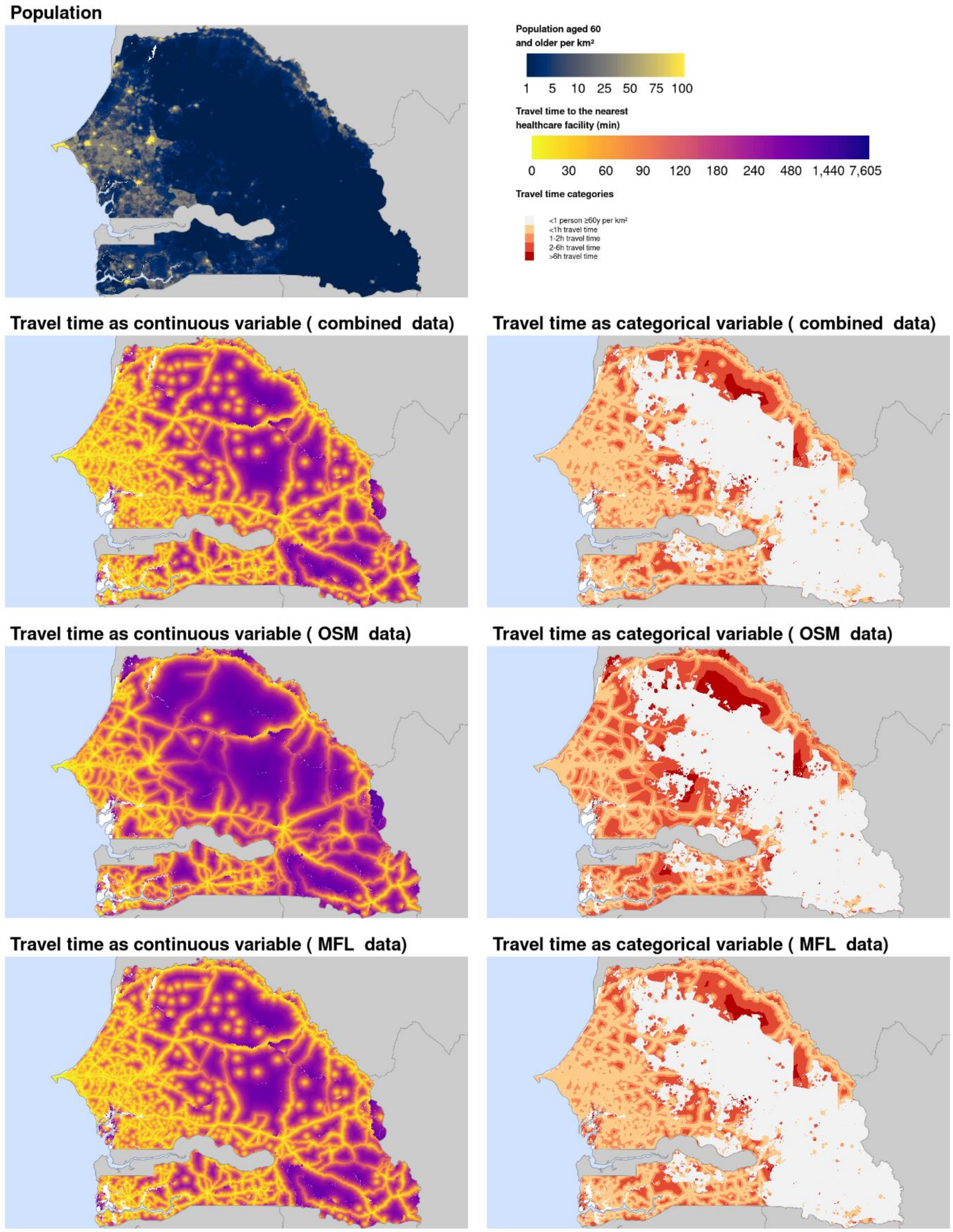

Figure S86. Sierra Leone map of travel time to the nearest healthcare facility for adults aged ≥ 60 years

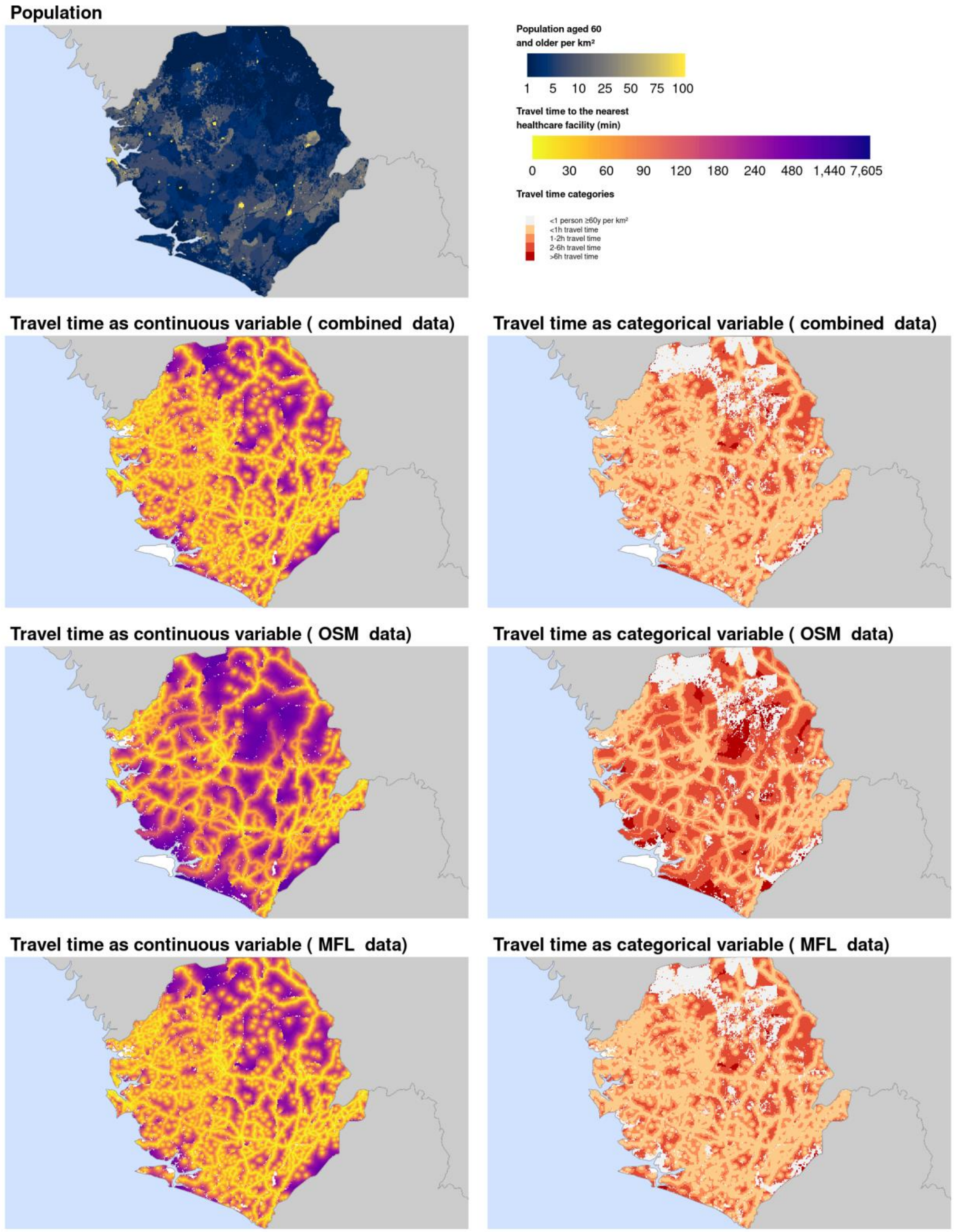

Figure S87. Somalia map of travel time to the nearest healthcare facility for adults aged ≥ 60 years

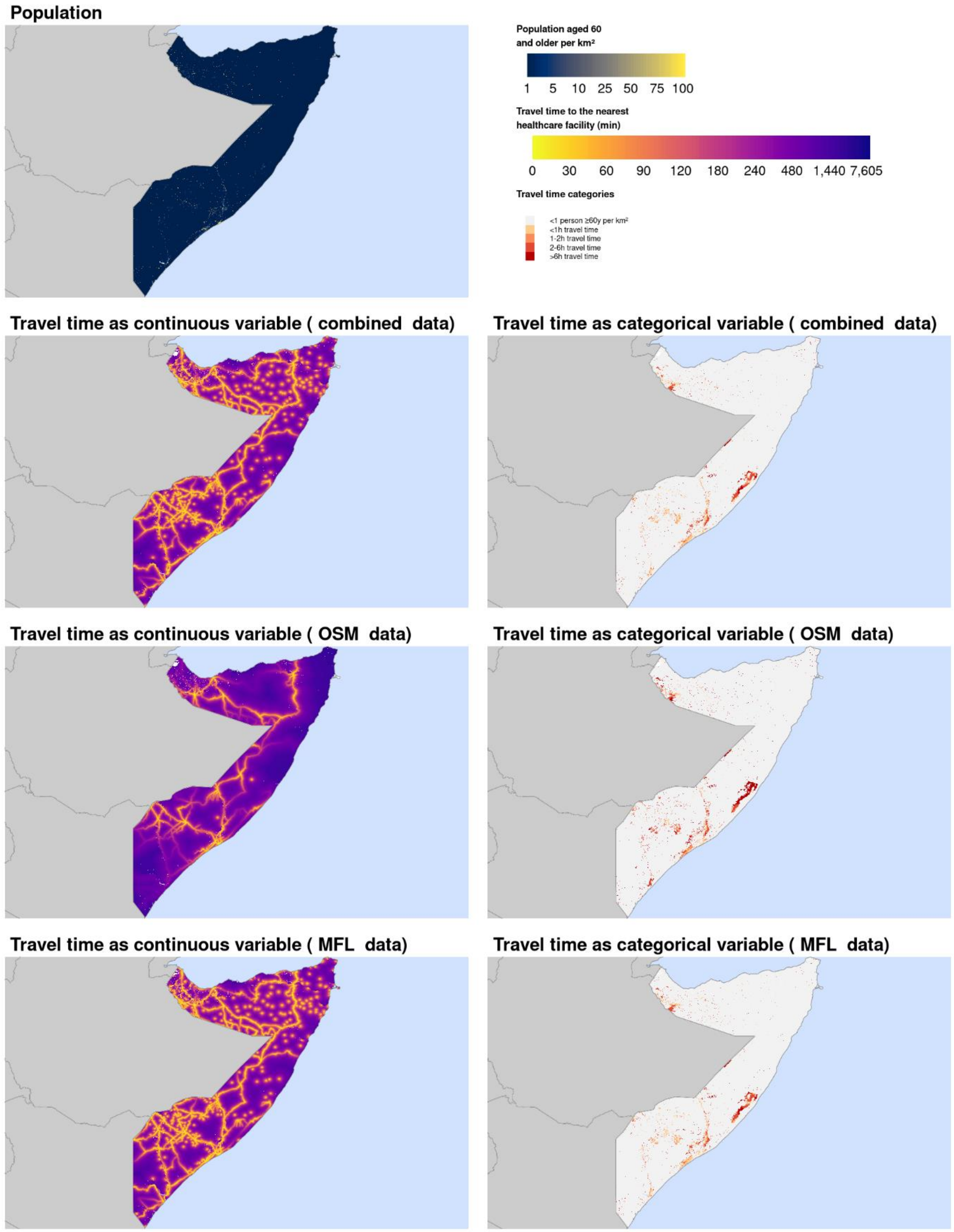

Figure S88. South Africa map of travel time to the nearest healthcare facility for adults aged ≥ 60 years

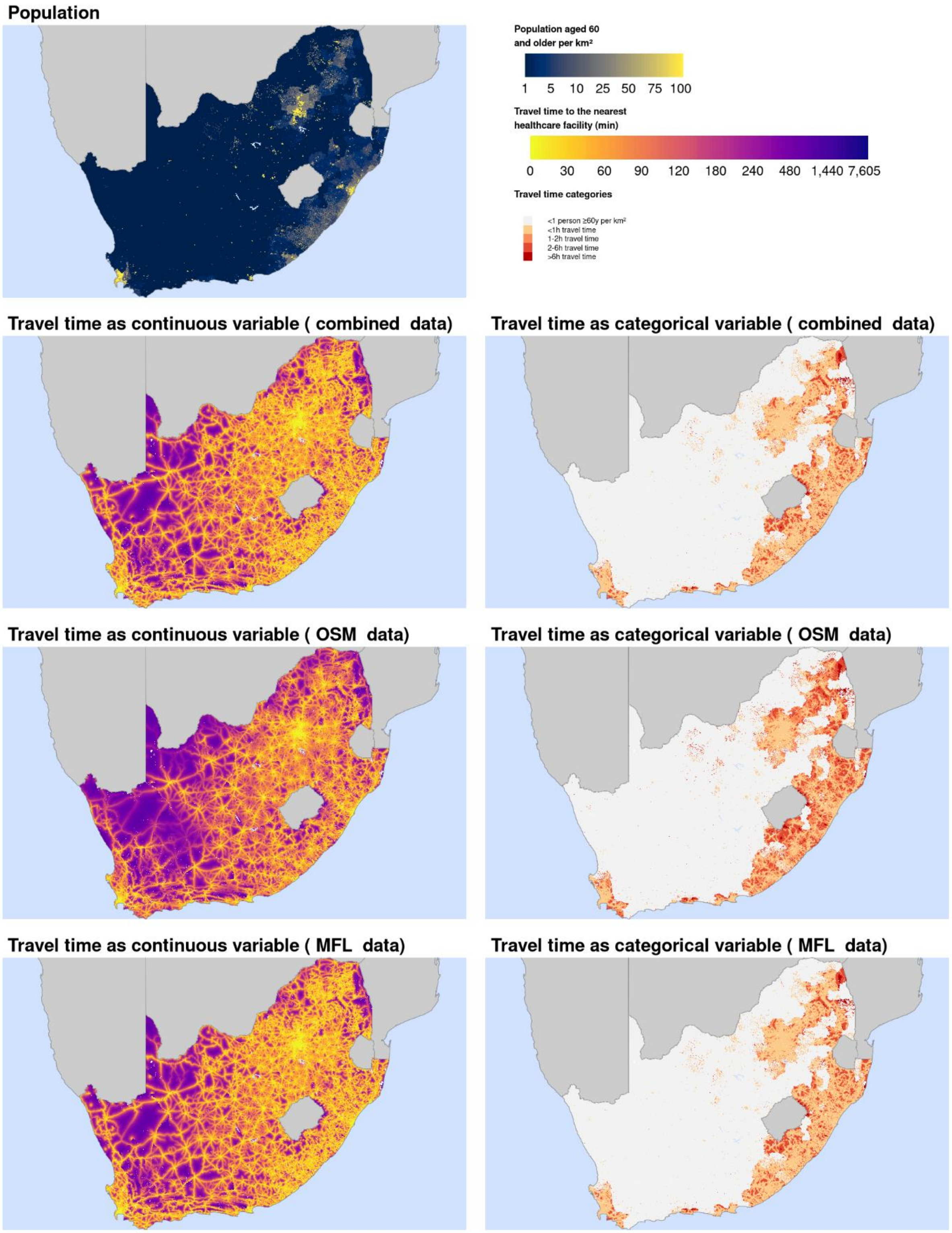

Figure S89. South Sudan map of travel time to the nearest healthcare facility for adults aged ≥ 60 years

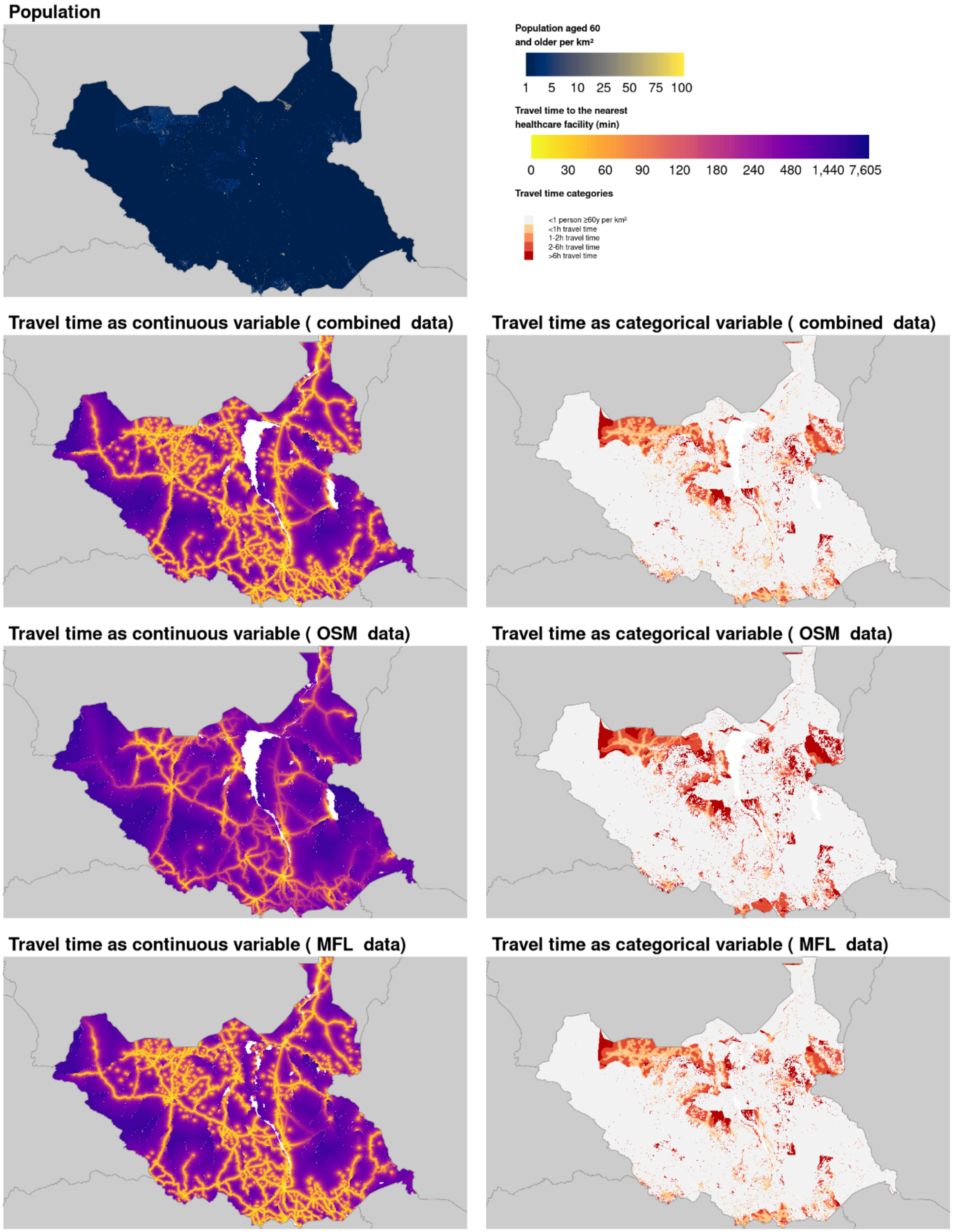

Figure S90. Sudan map of travel time to the nearest healthcare facility for adults aged ≥ 60 years

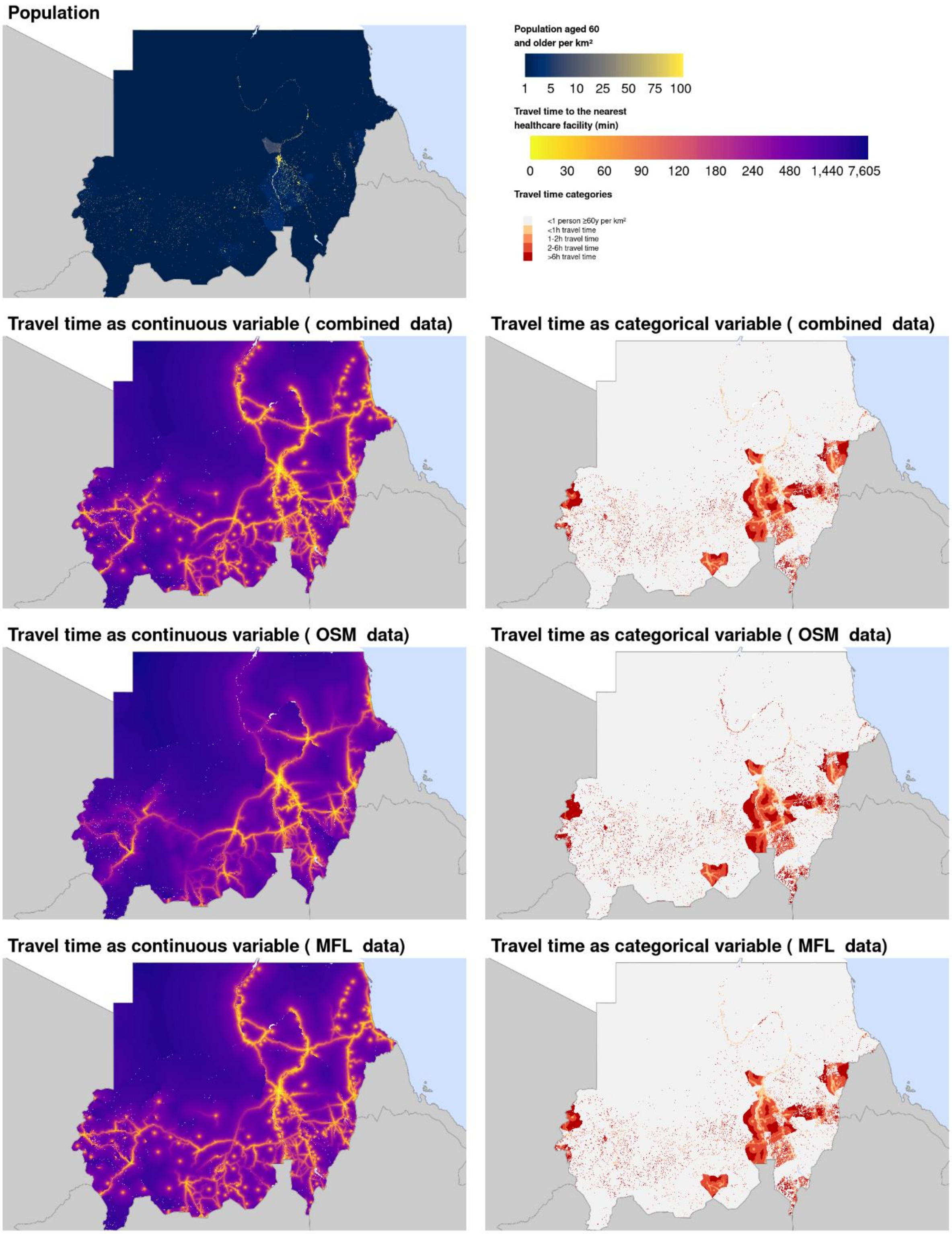

Figure S91. Tanzania map of travel time to the nearest healthcare facility for adults aged ≥ 60 years

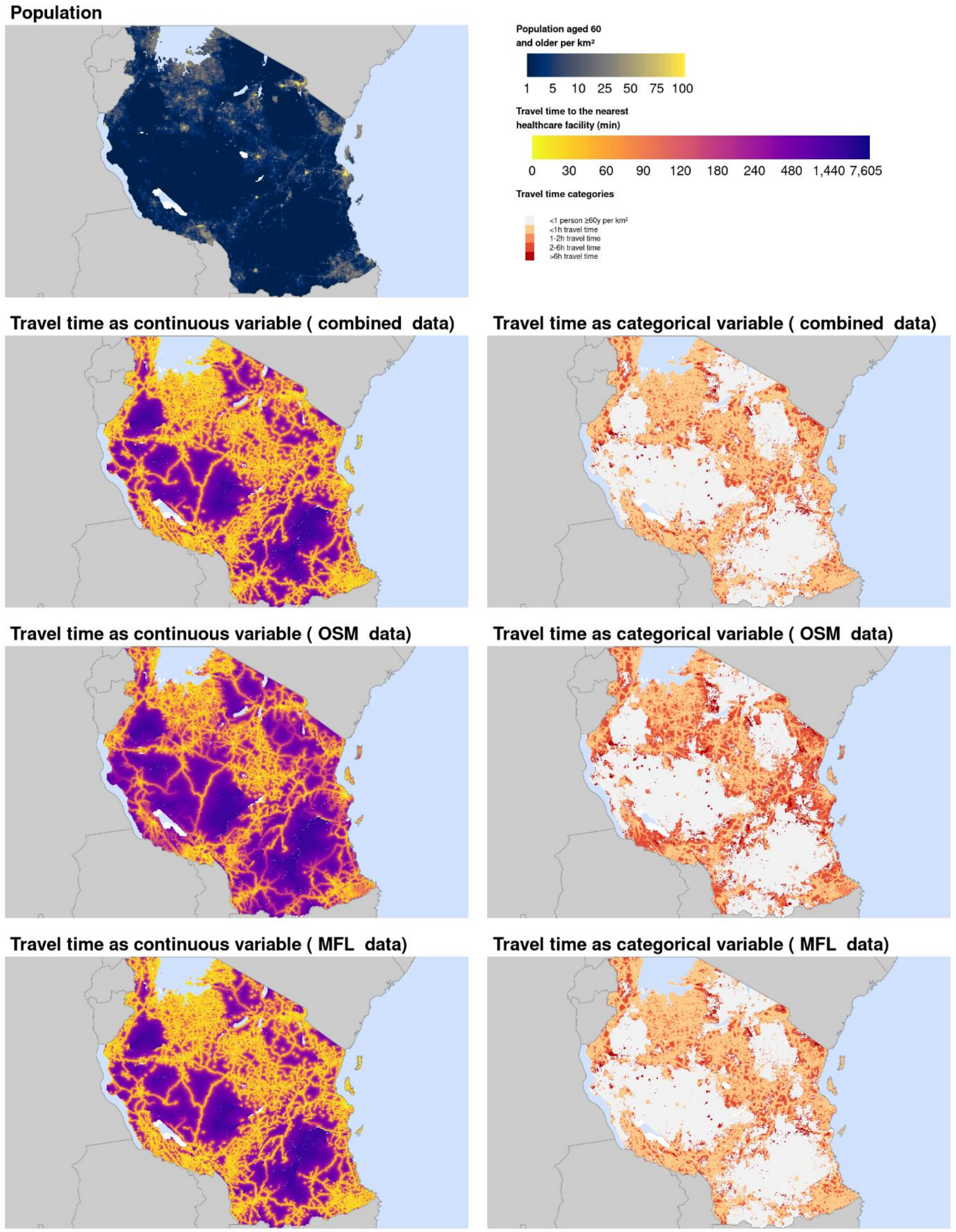

Figure S92. The Gambia map of travel time to the nearest healthcare facility for adults aged ≥ 60 years

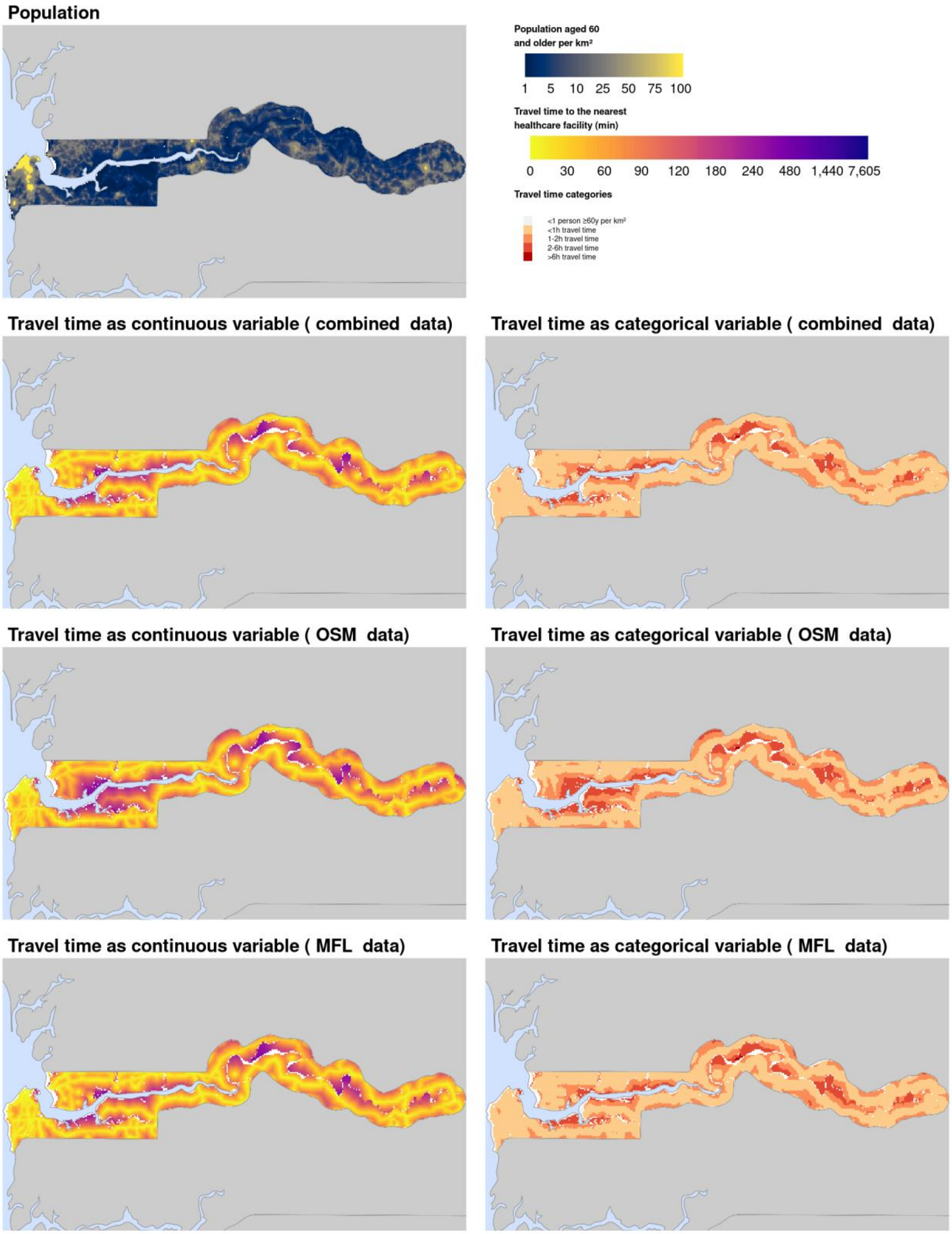

Figure S93. Togo map of travel time to the nearest healthcare facility for adults aged ≥ 60 years

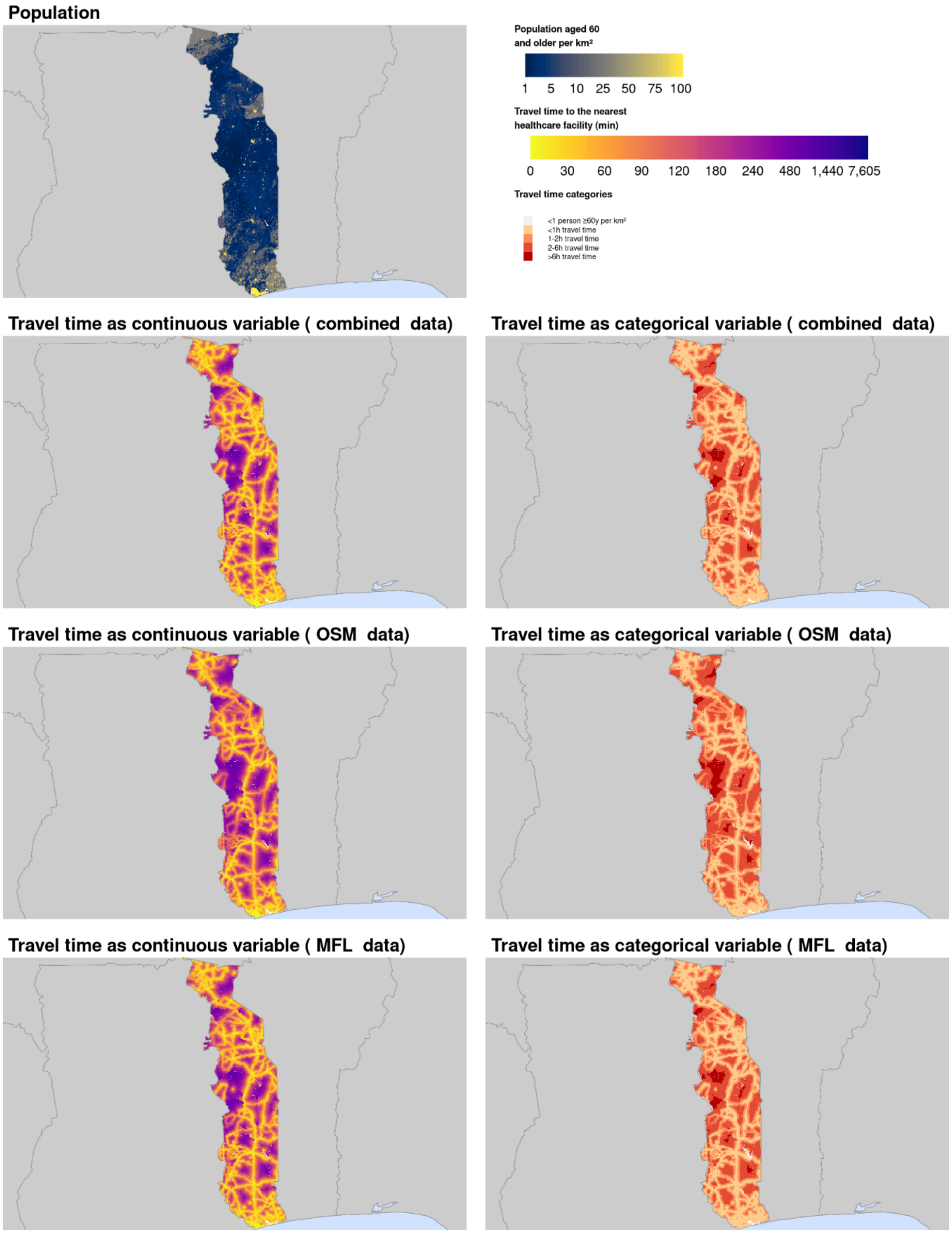

Figure S94. Uganda map of travel time to the nearest healthcare facility for adults aged ≥ 60 years

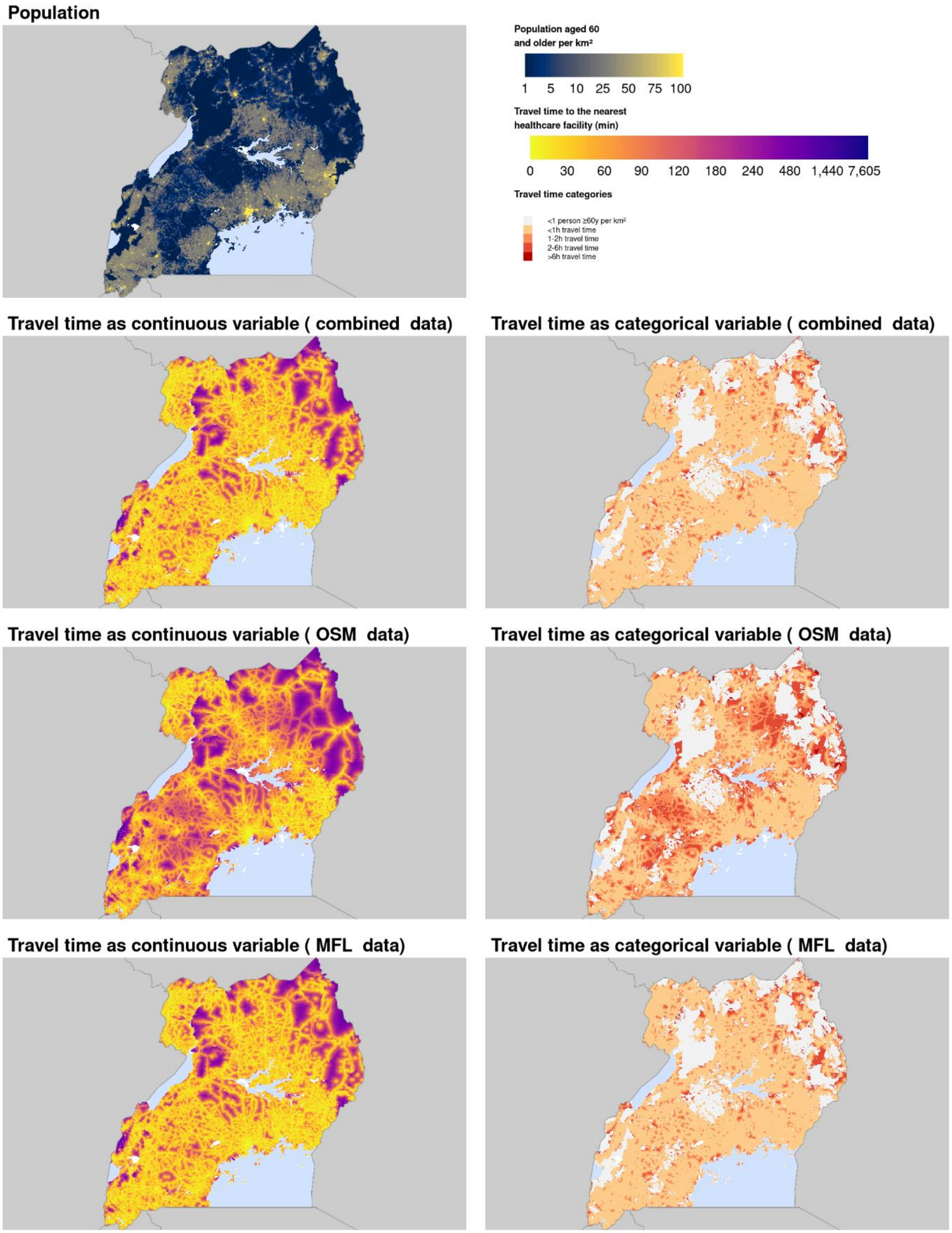

Figure S95. Zambia map of travel time to the nearest healthcare facility for adults aged ≥ 60 years

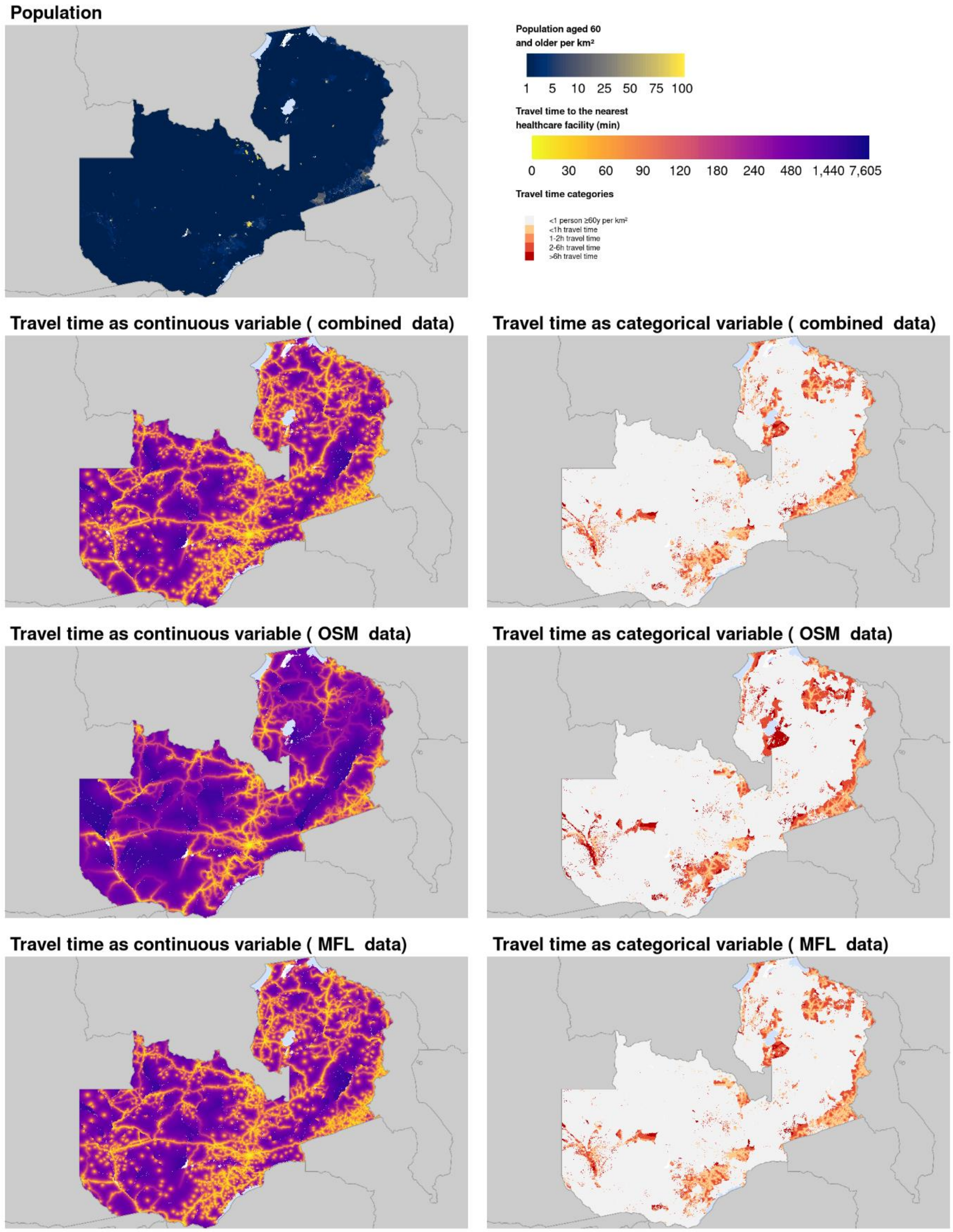

Figure S96. Zimbabwe map of travel time to the nearest healthcare facility for adults aged ≥ 60 years

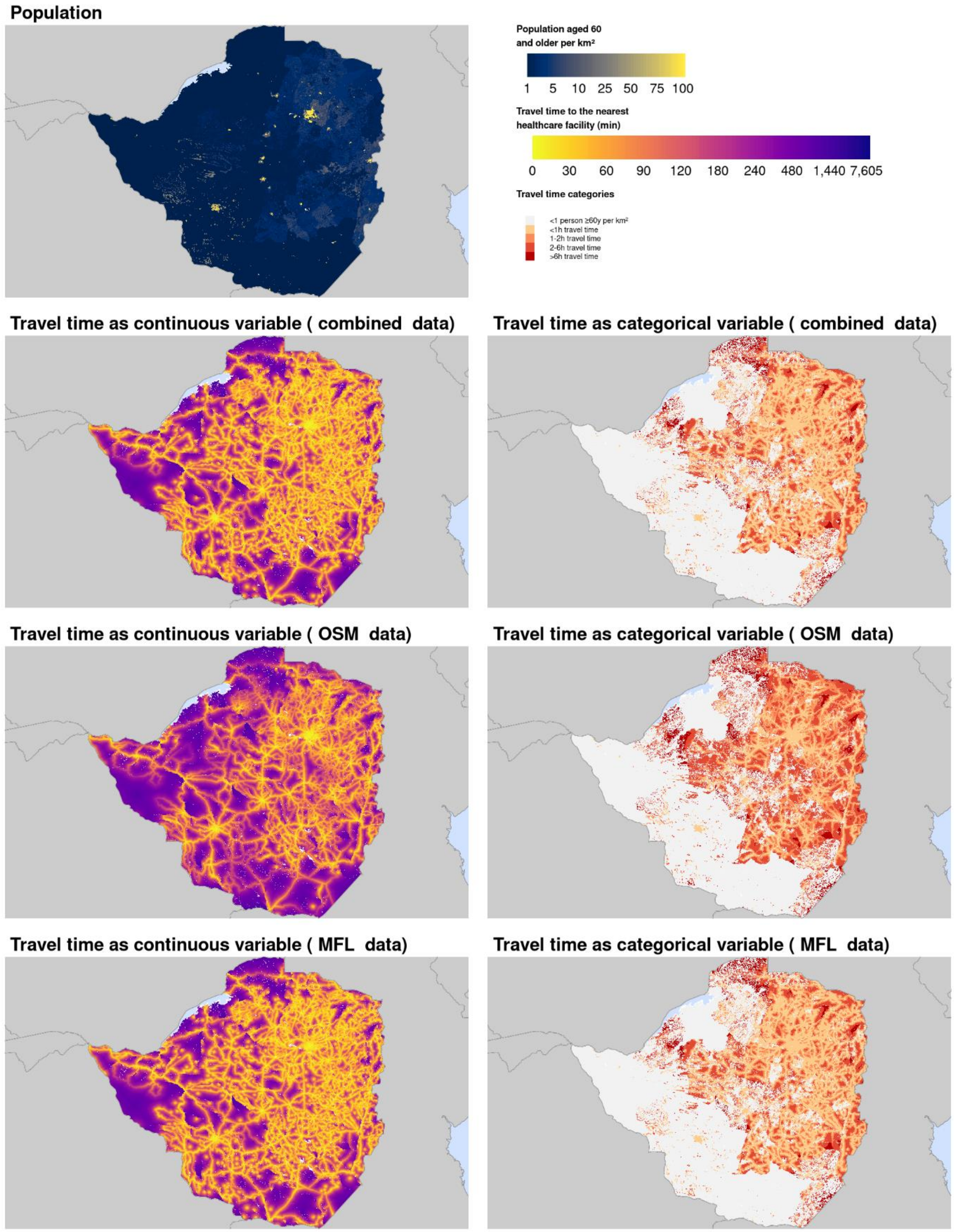

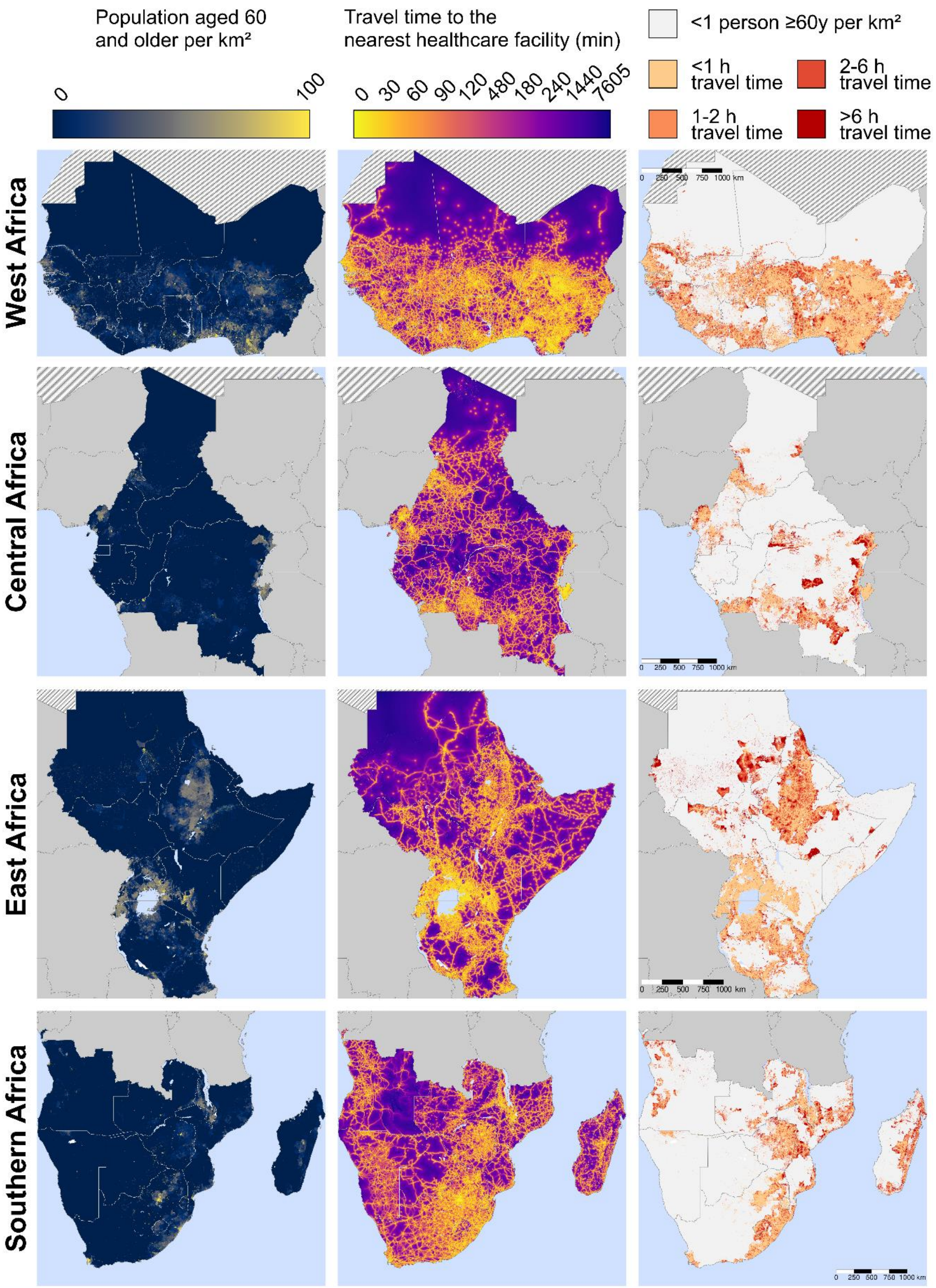

Figure S97. Maps of travel time to the nearest healthcare facility for adults ≥ 60 years, by region based on the MFL dataset

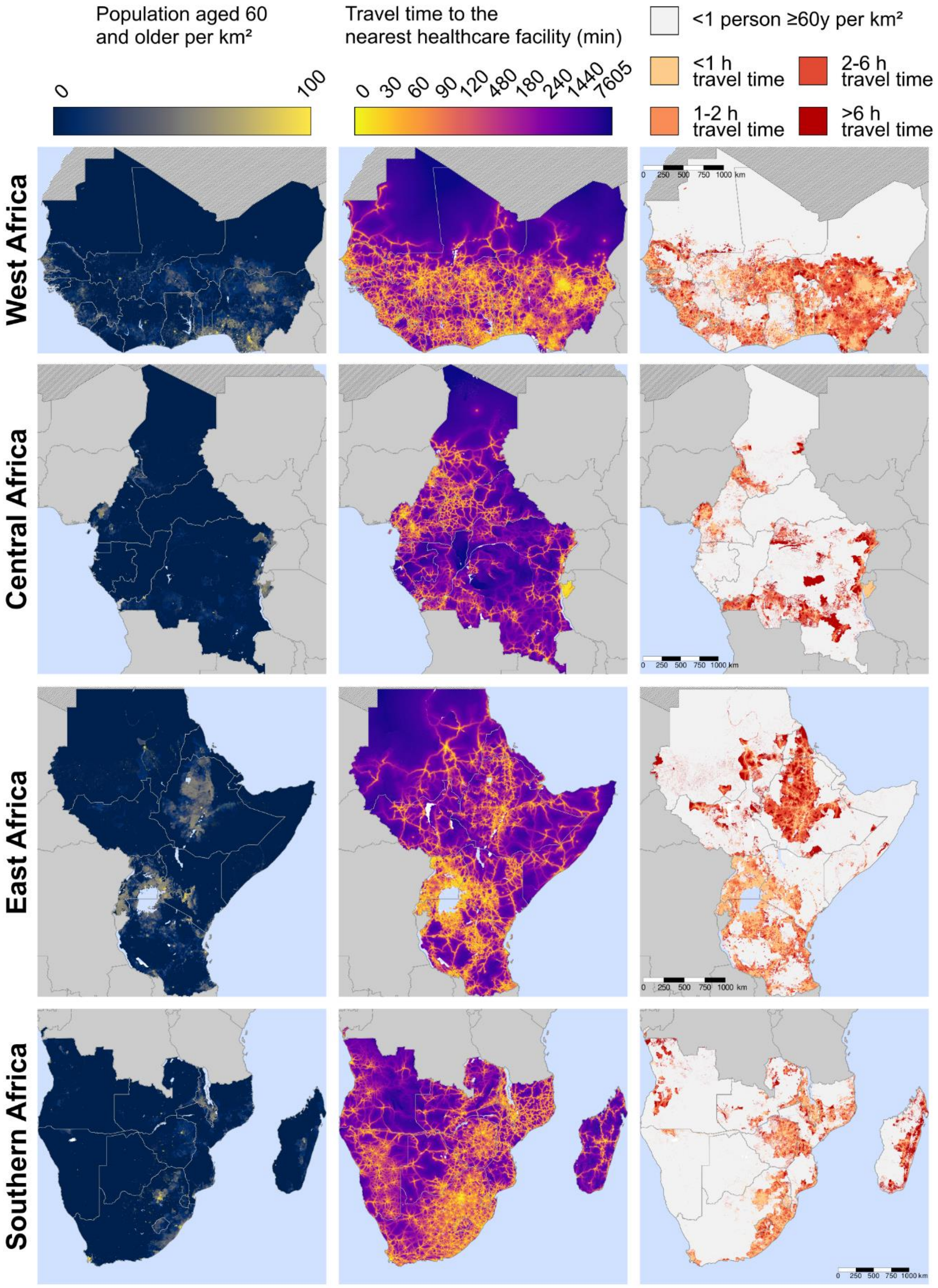

Figure S98. Maps of travel time to the nearest healthcare facility for adults ≥ 60 years, by region based on the OSM dataset
