# Supplementary material for: Mapping physical access to healthcare for older adults in sub-Saharan Africa: A cross-sectional analysis with implications for the COVID-19 response
Source: medRxiv. 2020 Aug 26:2020.07.17.20152389. Preprint. [Version 3] doi: 10.1101/2020.07.17.20152389 (PMC7386521; doi:10.1101/2020.07.17.20152389)
Supplement: Supplement 2020 [file 94402-2020.07.17.20152389-1.docx]

**Supplementary appendix**

[Figure S1. Distribution of travel time to the nearest hospital, by country 9](#_Toc49180618)

[Figure S2. Distribution of travel time to the nearest healthcare facility of any type, by country 10](#_Toc49180619)

[Figure S3. Histogram of travel time to the nearest hospital in the MFL dataset for adults aged 60 years and older, by country 11](#_Toc49180620)

[Figure S4. Histogram of travel time to the nearest hospital in the OSM dataset for adults aged 60 years and older, by country 12](#_Toc49180621)

[Figure S5. Histogram of travel time to the nearest healthcare facility (of any type) in the MFL dataset for adults aged 60 years and older, by country 13](#_Toc49180622)

[Figure S6. Histogram of travel time to the nearest healthcare facility (of any type) in the OSM dataset for adults aged 60 years and older, by country 14](#_Toc49180623)

[Figure S7. Angola map of travel time to the nearest hospital for adults aged ≥ 60 years 15](#_Toc49180624)

[Figure S8. Benin map of travel time to the nearest hospital for adults aged ≥ 60 years 16](#_Toc49180625)

[Figure S9. Botswana map of travel time to the nearest hospital for adults aged ≥ 60 years 17](#_Toc49180626)

[Figure S10. Burkina Faso map of travel time to the nearest hospital for adults aged ≥ 60 years 18](#_Toc49180627)

[Figure S11. Burundi map of travel time to the nearest hospital for adults aged ≥ 60 years 19](#_Toc49180628)

[Figure S12. Cameroon map of travel time to the nearest hospital for adults aged ≥ 60 years 20](#_Toc49180629)

[Figure S13. Central African Republic map of travel time to the nearest hospital for adults aged ≥ 60 years 21](#_Toc49180630)

[Figure S14. Chad map of travel time to the nearest hospital for adults aged ≥ 60 years 22](#_Toc49180631)

[Figure S15. Djibouti map of travel time to the nearest hospital for adults aged ≥ 60 years 23](#_Toc49180632)

[Figure S16. DRC map of travel time to the nearest hospital for adults aged ≥ 60 years 24](#_Toc49180633)

[Figure S17. Equatorial Guinea map of travel time to the nearest hospital for adults aged ≥ 60 years 25](#_Toc49180634)

[Figure S18. Eritrea map of travel time to the nearest hospital for adults aged ≥ 60 years 26](#_Toc49180635)

[Figure S19. eSwatini map of travel time to the nearest hospital for adults aged ≥ 60 years 27](#_Toc49180636)

[Figure S20. Ethiopia map of travel time to the nearest hospital for adults aged ≥ 60 years 28](#_Toc49180637)

[Figure S21. Gabon map of travel time to the nearest hospital for adults aged ≥ 60 years 29](#_Toc49180638)

[Figure S22. Ghana map of travel time to the nearest hospital for adults aged ≥ 60 years 30](#_Toc49180639)

[Figure S23. Guinea map of travel time to the nearest hospital for adults aged ≥ 60 years 31](#_Toc49180640)

[Figure S24. Guinea-Bissau map of travel time to the nearest hospital for adults aged ≥ 60 years 32](#_Toc49180641)

[Figure S25. Ivory Coast map of travel time to the nearest hospital for adults aged ≥ 60 years 33](#_Toc49180642)

[Figure S26. Kenya map of travel time to the nearest hospital for adults aged ≥ 60 years 34](#_Toc49180643)

[Figure S27. Lesotho map of travel time to the nearest hospital for adults aged ≥ 60 years 35](#_Toc49180644)

[Figure S28. Liberia map of travel time to the nearest hospital for adults aged ≥ 60 years 36](#_Toc49180645)

[Figure S29. Madagascar map of travel time to the nearest hospital for adults aged ≥ 60 years 37](#_Toc49180646)

[Figure S30. Malawi map of travel time to the nearest hospital for adults aged ≥ 60 years 38](#_Toc49180647)

[Figure S31. Mali map of travel time to the nearest hospital for adults aged ≥ 60 years 39](#_Toc49180648)

[Figure S32. Mauritania map of travel time to the nearest hospital for adults aged ≥ 60 years 40](#_Toc49180649)

[Figure S33. Mozambique map of travel time to the nearest hospital for adults aged ≥ 60 years 41](#_Toc49180650)

[Figure S34. Namibia map of travel time to the nearest hospital for adults aged ≥ 60 years 42](#_Toc49180651)

[Figure S35. Niger map of travel time to the nearest hospital for adults aged ≥ 60 years 43](#_Toc49180652)

[Figure S36. Nigeria map of travel time to the nearest hospital for adults aged ≥ 60 years 44](#_Toc49180653)

[Figure S37. Republic of the Congo map of travel time to the nearest hospital for adults aged ≥ 60 years 45](#_Toc49180654)

[Figure S38. Rwanda map of travel time to the nearest hospital for adults aged ≥ 60 years 46](#_Toc49180655)

[Figure S39. Senegal map of travel time to the nearest hospital for adults aged ≥ 60 years 47](#_Toc49180656)

[Figure S40. Sierra Leone map of travel time to the nearest hospital for adults aged ≥ 60 years 48](#_Toc49180657)

[Figure S41. Somalia map of travel time to the nearest hospital for adults aged ≥ 60 years 49](#_Toc49180658)

[Figure S42. South Africa map of travel time to the nearest hospital for adults aged ≥ 60 years 50](#_Toc49180659)

[Figure S43. South Sudan map of travel time to the nearest hospital for adults aged ≥ 60 years 51](#_Toc49180660)

[Figure S44. Sudan map of travel time to the nearest hospital for adults aged ≥ 60 years 52](#_Toc49180661)

[Figure S45. Tanzania map of travel time to the nearest hospital for adults aged ≥ 60 years 53](#_Toc49180662)

[Figure S46. The Gambia map of travel time to the nearest hospital for adults aged ≥ 60 years 54](#_Toc49180663)

[Figure S47. Togo map of travel time to the nearest hospital for adults aged ≥ 60 years 55](#_Toc49180664)

[Figure S48. Uganda map of travel time to the nearest hospital for adults aged ≥ 60 years 56](#_Toc49180665)

[Figure S49. Zambia map of travel time to the nearest hospital for adults aged ≥ 60 years 57](#_Toc49180666)

[Figure S50. Zimbabwe map of travel time to the nearest hospital for adults aged ≥ 60 years 58](#_Toc49180667)

[Figure S51. Maps of travel time to the nearest hospital for adults ≥ 60 years, by region based on the MFL dataset 59](#_Toc49180668)

[Figure S52. Maps of travel time to the nearest hospital for adults ≥ 60 years, by region based on the OSM dataset 60](#_Toc49180669)

[Figure S53. Angola map of travel time to the nearest healthcare facility for adults aged ≥ 60 years 61](#_Toc49180670)

[Figure S54. Benin map of travel time to the nearest healthcare facility for adults aged ≥ 60 years 62](#_Toc49180671)

[Figure S55. Botswana map of travel time to the nearest healthcare facility for adults aged ≥ 60 years 63](#_Toc49180672)

[Figure S56. Burkina Faso map of travel time to the nearest healthcare facility for adults aged ≥ 60 years 64](#_Toc49180673)

[Figure S57. Burundi map of travel time to the nearest healthcare facility for adults aged ≥ 60 years 65](#_Toc49180674)

[Figure S58. Cameroon map of travel time to the nearest healthcare facility for adults aged ≥ 60 years 66](#_Toc49180675)

[Figure S59. Central African Republic map of travel time to the nearest healthcare facility for adults aged ≥ 60 years 67](#_Toc49180676)

[Figure S60. Chad map of travel time to the nearest healthcare facility for adults aged ≥ 60 years 68](#_Toc49180677)

[Figure S61. Djibouti map of travel time to the nearest healthcare facility for adults aged ≥ 60 years 69](#_Toc49180678)

[Figure S62. DRC map of travel time to the nearest healthcare facility for adults aged ≥ 60 years 70](#_Toc49180679)

[Figure S63. Equatorial Guinea map of travel time to the nearest healthcare facility for adults aged ≥ 60 years 71](#_Toc49180680)

[Figure S64. Eritrea map of travel time to the nearest healthcare facility for adults aged ≥ 60 years 72](#_Toc49180681)

[Figure S65. eSwatini map of travel time to the nearest healthcare facility for adults aged ≥ 60 years 73](#_Toc49180682)

[Figure S66. Ethiopia map of travel time to the nearest healthcare facility for adults aged ≥ 60 years 74](#_Toc49180683)

[Figure S67. Gabon map of travel time to the nearest healthcare facility for adults aged ≥ 60 years 75](#_Toc49180684)

[Figure S68. Ghana map of travel time to the nearest healthcare facility for adults aged ≥ 60 years 76](#_Toc49180685)

[Figure S69. Guinea map of travel time to the nearest healthcare facility for adults aged ≥ 60 years 77](#_Toc49180686)

[Figure S70. Guinea-Bissau map of travel time to the nearest healthcare facility for adults aged ≥ 60 years 78](#_Toc49180687)

[Figure S71. Ivory Coast map of travel time to the nearest healthcare facility for adults aged ≥ 60 years 79](#_Toc49180688)

[Figure S72. Kenya map of travel time to the nearest healthcare facility for adults aged ≥ 60 years 80](#_Toc49180689)

[Figure S73. Lesotho map of travel time to the nearest healthcare facility for adults aged ≥ 60 years 81](#_Toc49180690)

[Figure S74. Liberia map of travel time to the nearest healthcare facility for adults aged ≥ 60 years 82](#_Toc49180691)

[Figure S75. Madagascar map of travel time to the nearest healthcare facility for adults aged ≥ 60 years 83](#_Toc49180692)

[Figure S76. Malawi map of travel time to the nearest healthcare facility for adults aged ≥ 60 years 84](#_Toc49180693)

[Figure S77. Mali map of travel time to the nearest healthcare facility for adults aged ≥ 60 years 85](#_Toc49180694)

[Figure S78. Mauritania map of travel time to the nearest healthcare facility for adults aged ≥ 60 years 86](#_Toc49180695)

[Figure S79. Mozambique map of travel time to the nearest healthcare facility for adults aged ≥ 60 years 87](#_Toc49180696)

[Figure S80. Namibia map of travel time to the nearest healthcare facility for adults aged ≥ 60 years 88](#_Toc49180697)

[Figure S81. Niger map of travel time to the nearest healthcare facility for adults aged ≥ 60 years 89](#_Toc49180698)

[Figure S82. Nigeria map of travel time to the nearest healthcare facility for adults aged ≥ 60 years 90](#_Toc49180699)

[Figure S83. Republic of the Congo map of travel time to the nearest healthcare facility for adults aged ≥ 60 years 91](#_Toc49180700)

[Figure S84. Rwanda map of travel time to the nearest healthcare facility for adults aged ≥ 60 years 92](#_Toc49180701)

[Figure S85. Senegal map of travel time to the nearest healthcare facility for adults aged ≥ 60 years 93](#_Toc49180702)

[Figure S86. Sierra Leone map of travel time to the nearest healthcare facility for adults aged ≥ 60 years 94](#_Toc49180703)

[Figure S87. Somalia map of travel time to the nearest healthcare facility for adults aged ≥ 60 years 95](#_Toc49180704)

[Figure S88. South Africa map of travel time to the nearest healthcare facility for adults aged ≥ 60 years 96](#_Toc49180705)

[Figure S89. South Sudan map of travel time to the nearest healthcare facility for adults aged ≥ 60 years 97](#_Toc49180706)

[Figure S90. Sudan map of travel time to the nearest healthcare facility for adults aged ≥ 60 years 98](#_Toc49180707)

[Figure S91. Tanzania map of travel time to the nearest healthcare facility for adults aged ≥ 60 years 99](#_Toc49180708)

[Figure S92. The Gambia map of travel time to the nearest healthcare facility for adults aged ≥ 60 years 100](#_Toc49180709)

[Figure S93. Togo map of travel time to the nearest healthcare facility for adults aged ≥ 60 years 101](#_Toc49180710)

[Figure S94. Uganda map of travel time to the nearest healthcare facility for adults aged ≥ 60 years 102](#_Toc49180711)

[Figure S95. Zambia map of travel time to the nearest healthcare facility for adults aged ≥ 60 years 103](#_Toc49180712)

[Figure S96. Zimbabwe map of travel time to the nearest healthcare facility for adults aged ≥ 60 years 104](#_Toc49180713)

[Figure S97. Maps of travel time to the nearest healthcare facility for adults ≥ 60 years, by region based on the MFL dataset 105](#_Toc49180714)

[Figure S98. Maps of travel time to the nearest healthcare facility for adults ≥ 60 years, by region based on the OSM dataset 106](#_Toc49180715)

[Figure S99. Location of healthcare facilities in OSM and MFL data for Angola 107](#_Toc49180716)

[Figure S100. Location of healthcare facilities in OSM and MFL data for Benin 107](#_Toc49180717)

[Figure S101. Location of healthcare facilities in OSM and MFL data for Botswana 108](#_Toc49180718)

[Figure S102. Location of healthcare facilities in OSM and MFL data for Burkina Faso 108](#_Toc49180719)

[Figure S103. Location of healthcare facilities in OSM and MFL data for Burundi 109](#_Toc49180720)

[Figure S104. Location of healthcare facilities in OSM and MFL data for Cameroon 109](#_Toc49180721)

[Figure S105. Location of healthcare facilities in OSM and MFL data for Central African Republic 110](#_Toc49180722)

[Figure S106. Location of healthcare facilities in OSM and MFL data for Chad 110](#_Toc49180723)

[Figure S107. Location of healthcare facilities in OSM and MFL data for Djibouti 111](#_Toc49180724)

[Figure S108. Location of healthcare facilities in OSM and MFL data for DRC 111](#_Toc49180725)

[Figure S109. Location of healthcare facilities in OSM and MFL data for Equatorial Guinea 112](#_Toc49180726)

[Figure S110. Location of healthcare facilities in OSM and MFL data for Eritrea 112](#_Toc49180727)

[Figure S111. Location of healthcare facilities in OSM and MFL data for eSwatini 113](#_Toc49180728)

[Figure S112. Location of healthcare facilities in OSM and MFL data for Ethiopia 113](#_Toc49180729)

[Figure S113. Location of healthcare facilities in OSM and MFL data for Gabon 114](#_Toc49180730)

[Figure S114. Location of healthcare facilities in OSM and MFL data for Ghana 114](#_Toc49180731)

[Figure S115. Location of healthcare facilities in OSM and MFL data for Guinea 115](#_Toc49180732)

[Figure S116. Location of healthcare facilities in OSM and MFL data for Guinea-Bissau 115](#_Toc49180733)

[Figure S117. Location of healthcare facilities in OSM and MFL data for Ivory Coast 116](#_Toc49180734)

[Figure S118. Location of healthcare facilities in OSM and MFL data for Kenya 116](#_Toc49180735)

[Figure S119. Location of healthcare facilities in OSM and MFL data for Lesotho 117](#_Toc49180736)

[Figure S120. Location of healthcare facilities in OSM and MFL data for Liberia 117](#_Toc49180737)

[Figure S121. Location of healthcare facilities in OSM and MFL data for Madagascar 118](#_Toc49180738)

[Figure S122. Location of healthcare facilities in OSM and MFL data for Malawi 118](#_Toc49180739)

[Figure S123. Location of healthcare facilities in OSM and MFL data for Mali 119](#_Toc49180740)

[Figure S124. Location of healthcare facilities in OSM and MFL data for Mauritania 119](#_Toc49180741)

[Figure S125. Location of healthcare facilities in OSM and MFL data for Mozambique 120](#_Toc49180742)

[Figure S126. Location of healthcare facilities in OSM and MFL data for Namibia 120](#_Toc49180743)

[Figure S127. Location of healthcare facilities in OSM and MFL data for Niger 121](#_Toc49180744)

[Figure S128. Location of healthcare facilities in OSM and MFL data for Nigeria 121](#_Toc49180745)

[Figure S129. Location of healthcare facilities in OSM and MFL data for Republic of the Congo 122](#_Toc49180746)

[Figure S130. Location of healthcare facilities in OSM and MFL data for Rwanda 122](#_Toc49180747)

[Figure S131. Location of healthcare facilities in OSM and MFL data for Senegal 123](#_Toc49180748)

[Figure S132. Location of healthcare facilities in OSM and MFL data for Sierra Leone 123](#_Toc49180749)

[Figure S133. Location of healthcare facilities in OSM and MFL data for Somalia 124](#_Toc49180750)

[Figure S134. Location of healthcare facilities in OSM and MFL data for South Africa 124](#_Toc49180751)

[Figure S135. Location of healthcare facilities in OSM and MFL data for South Sudan 125](#_Toc49180752)

[Figure S136. Location of healthcare facilities in OSM and MFL data for Sudan 125](#_Toc49180753)

[Figure S137. Location of healthcare facilities in OSM and MFL data for Tanzania 126](#_Toc49180754)

[Figure S138. Location of healthcare facilities in OSM and MFL data for The Gambia 126](#_Toc49180755)

[Figure S139. Location of healthcare facilities in OSM and MFL data for Togo 127](#_Toc49180756)

[Figure S140. Location of healthcare facilities in OSM and MFL data for Uganda 127](#_Toc49180757)

[Figure S141. Location of healthcare facilities in OSM and MFL data for Zambia 128](#_Toc49180758)

[Figure S142. Location of healthcare facilities in OSM and MFL data for Zimbabwe
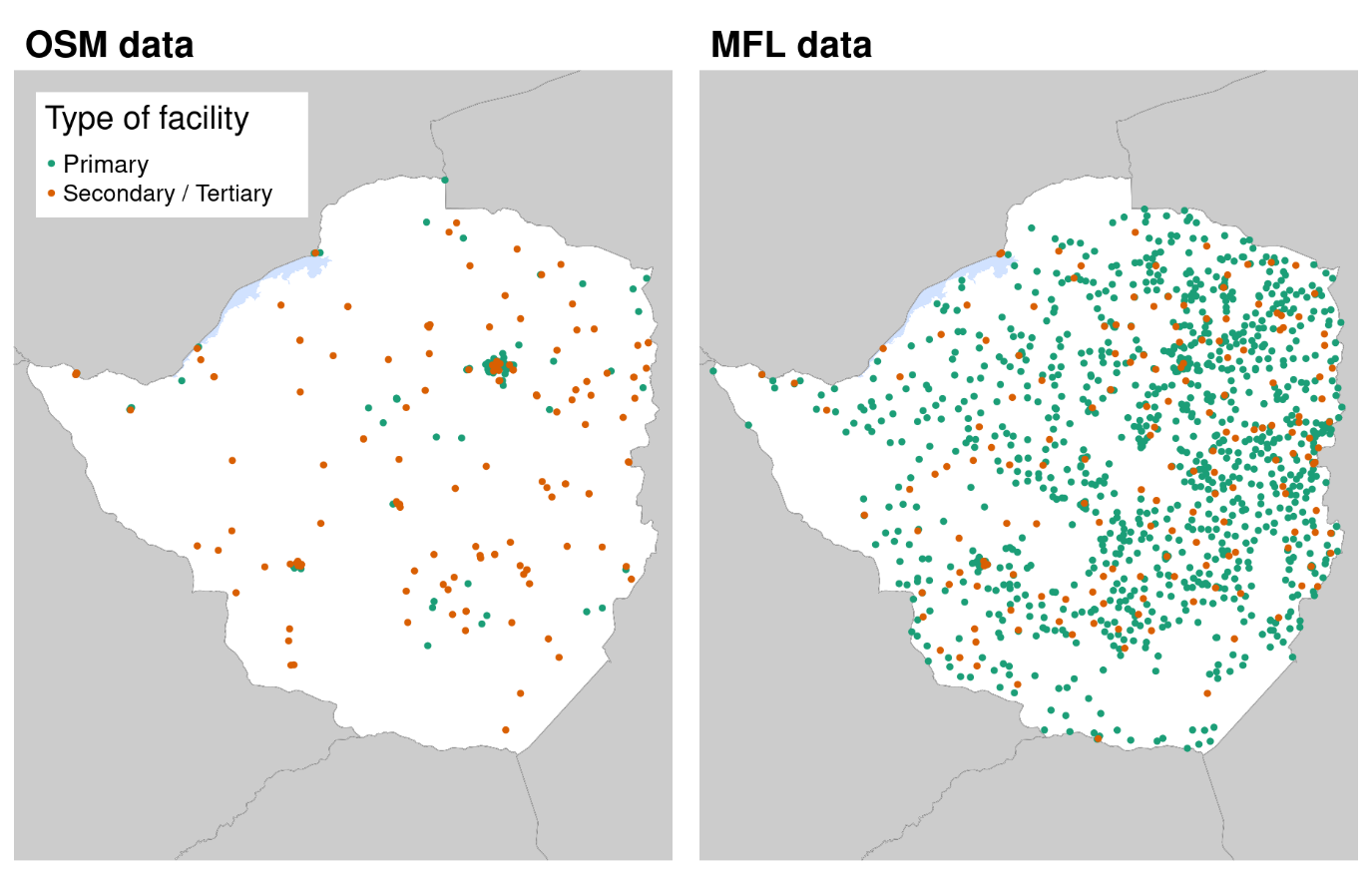
 129](#_Toc49180759)

[Table S1. Overlap of the GPS location of healthcare facilities with a building footprint and settlement locations in Bing satellite imagery 129](#_Toc49180760)

# **
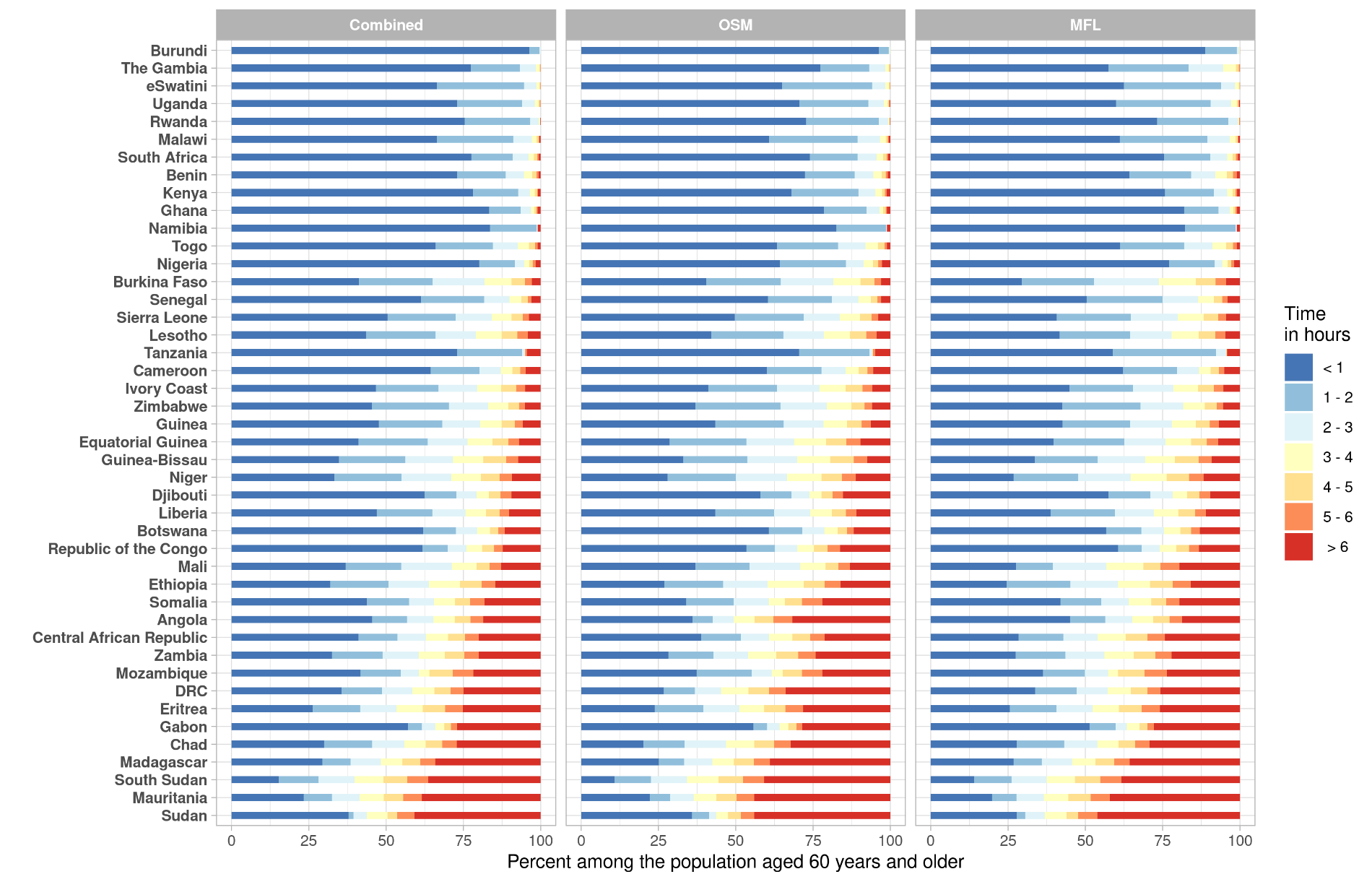
Figure S1. Distribution of travel time to the nearest hospital, by count****ry**

Abbreviations: DRC=Democratic Republic of the Congo

“Combined” refers to the travel time to the nearest hospital regardless of whether the hospital was recorded in the MFL or OSM data.

#
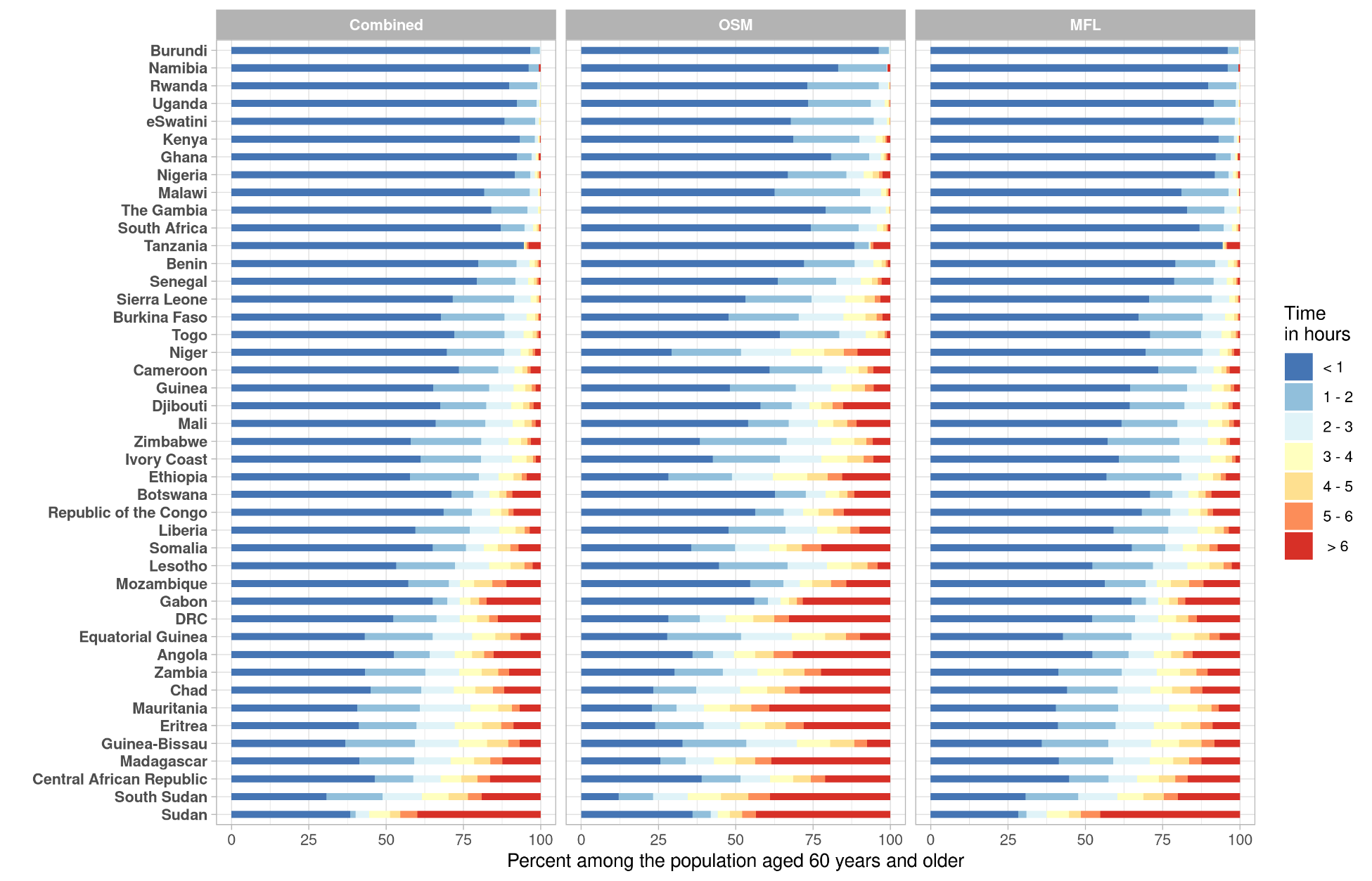
**Figure S2. Distribution of travel time to the nearest healthcare facility of any type, by count****ry**

Abbreviations: DRC=Democratic Republic of the Congo

“Combined” refers to the travel time to the nearest healthcare facility regardless of whether the facility was recorded in the MFL or OSM data.

**
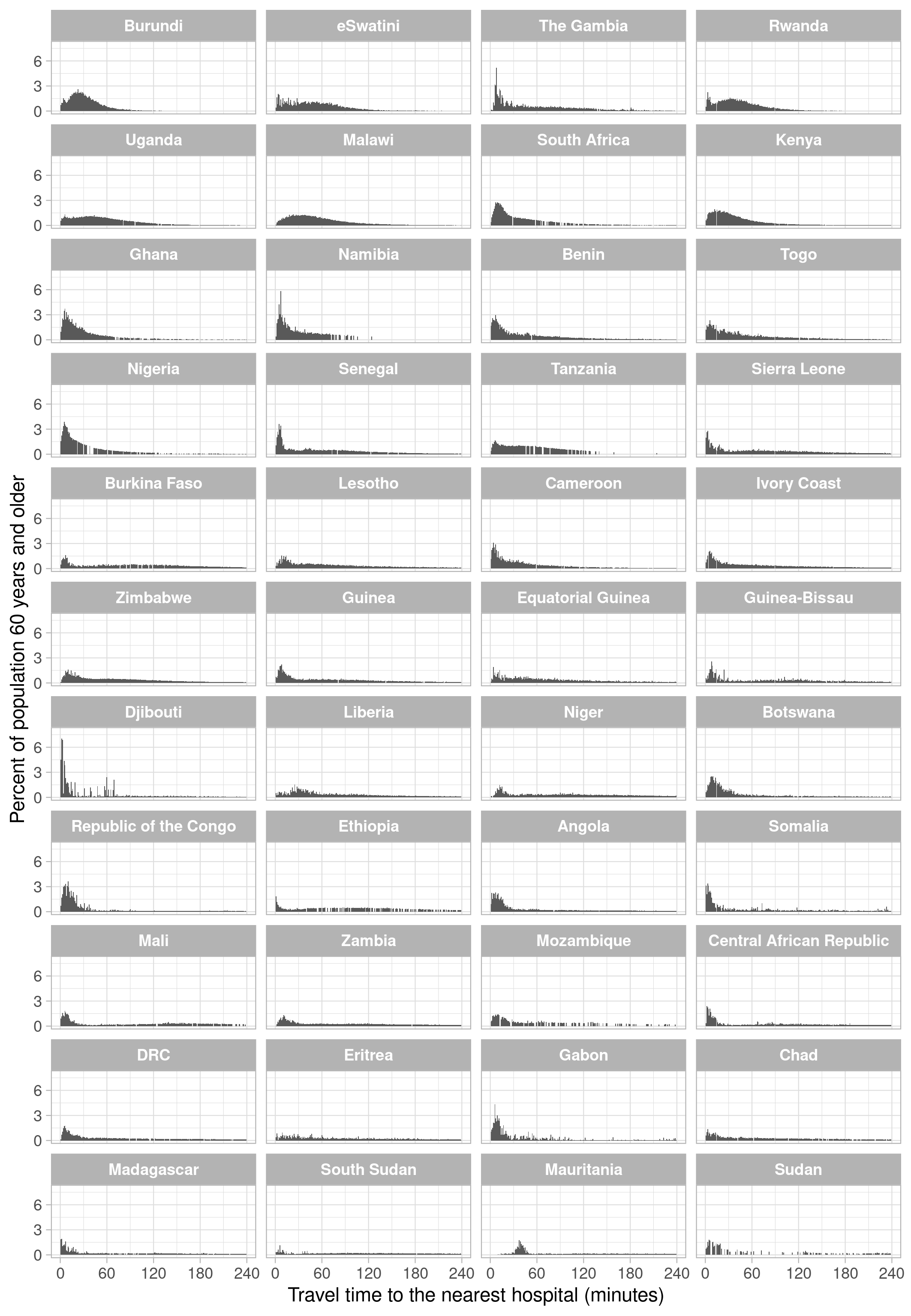
****Figure S3. Histogram of travel time to the nearest hospital in the MFL dataset for adults aged 60 years and older, by country^1^**

Abbreviations: DRC=Democratic Republic of the Congo
^1^ Countries were ordered in ascending order by the proportion of adults aged 60 years and older in their population who reside in a 1km x 1km area that has an estimated travel time >2 hours to the nearest hospital.

**Figure S4. Histogram of travel time to the nearest hospital in the OSM dataset for adults aged 60 years and older, by country^1^**


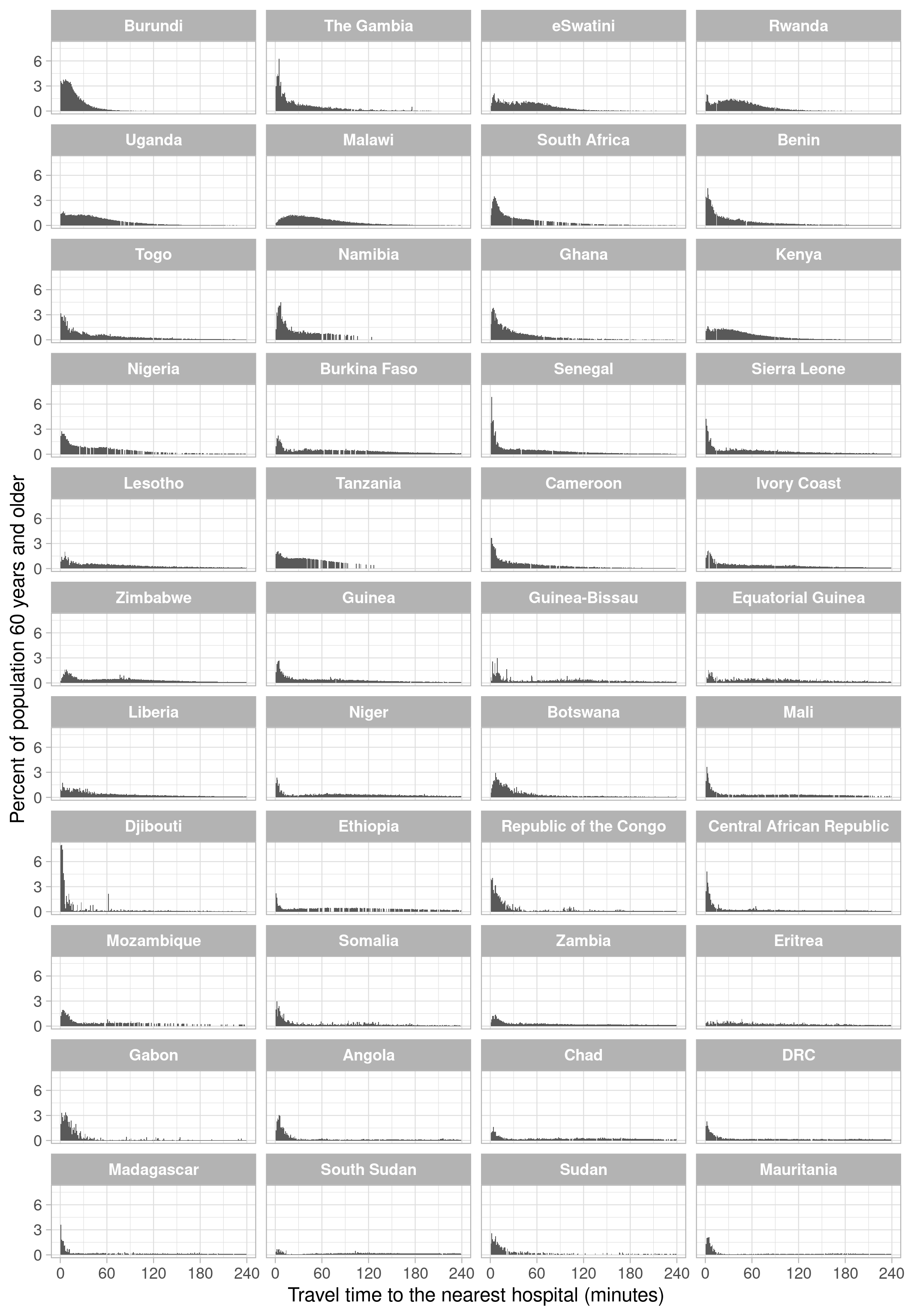
Abbreviations: DRC=Democratic Republic of the Congo
^1^ Countries were ordered in ascending order by the proportion of adults aged 60 years and older in their population who reside in a 1km x 1km area that has an estimated travel time >2 hours to the nearest hospital.

**
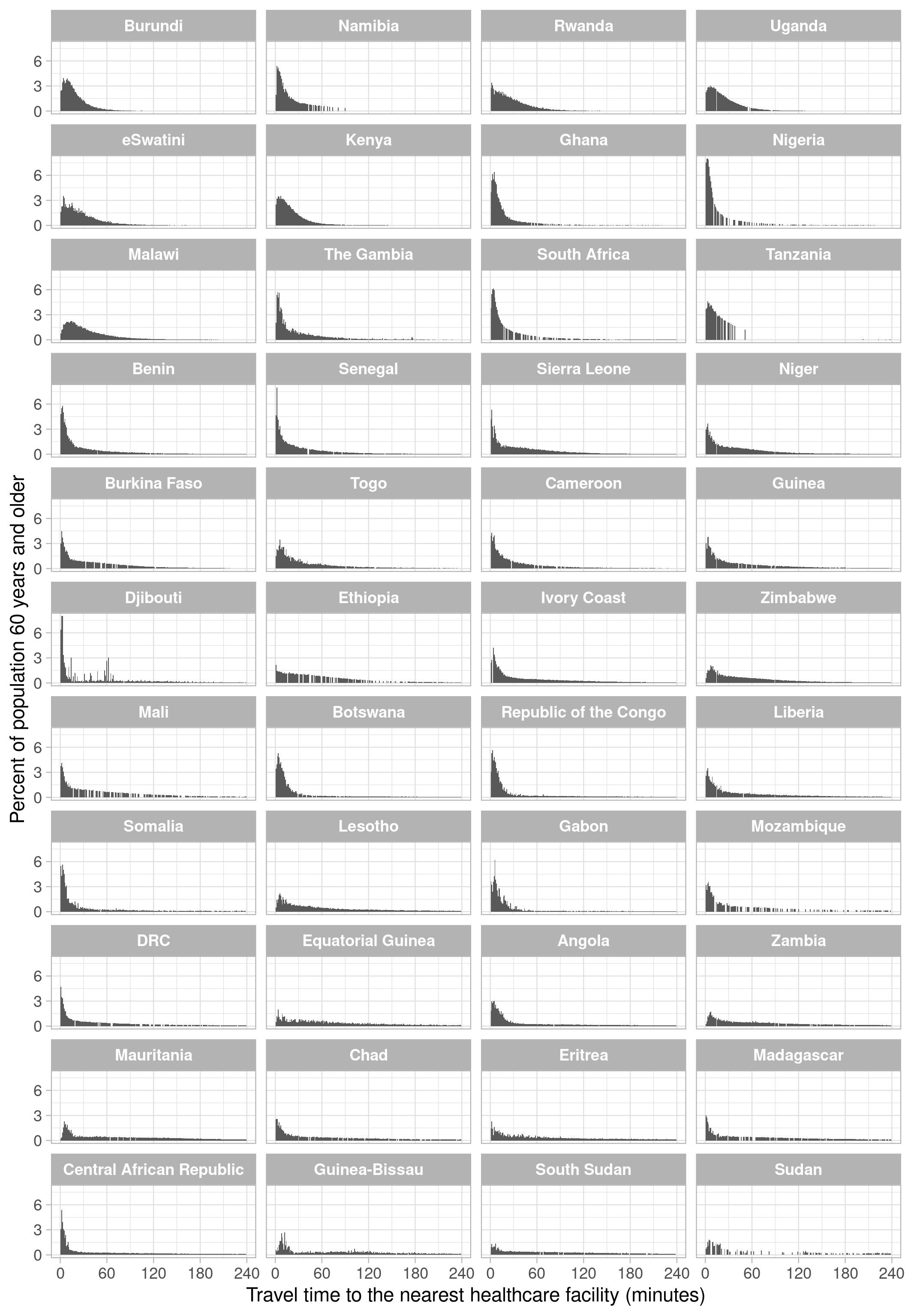
****Figure S5. Histogram of travel time to the nearest healthcare facility (of any type) in the MFL dataset for adults aged 60 years and older, by country^1^**

Abbreviations: DRC=Democratic Republic of the Congo
^1^ Countries were ordered in ascending order by the proportion of adults aged 60 years and older in their population who reside in a 1km x 1km area that has an estimated travel time >2 hours to the nearest healthcare facility.

**Figure S6. Histogram of travel time to the nearest healthcare facility (of any type) in the OSM dataset for adults aged 60 years and older, by countr****y^1^**


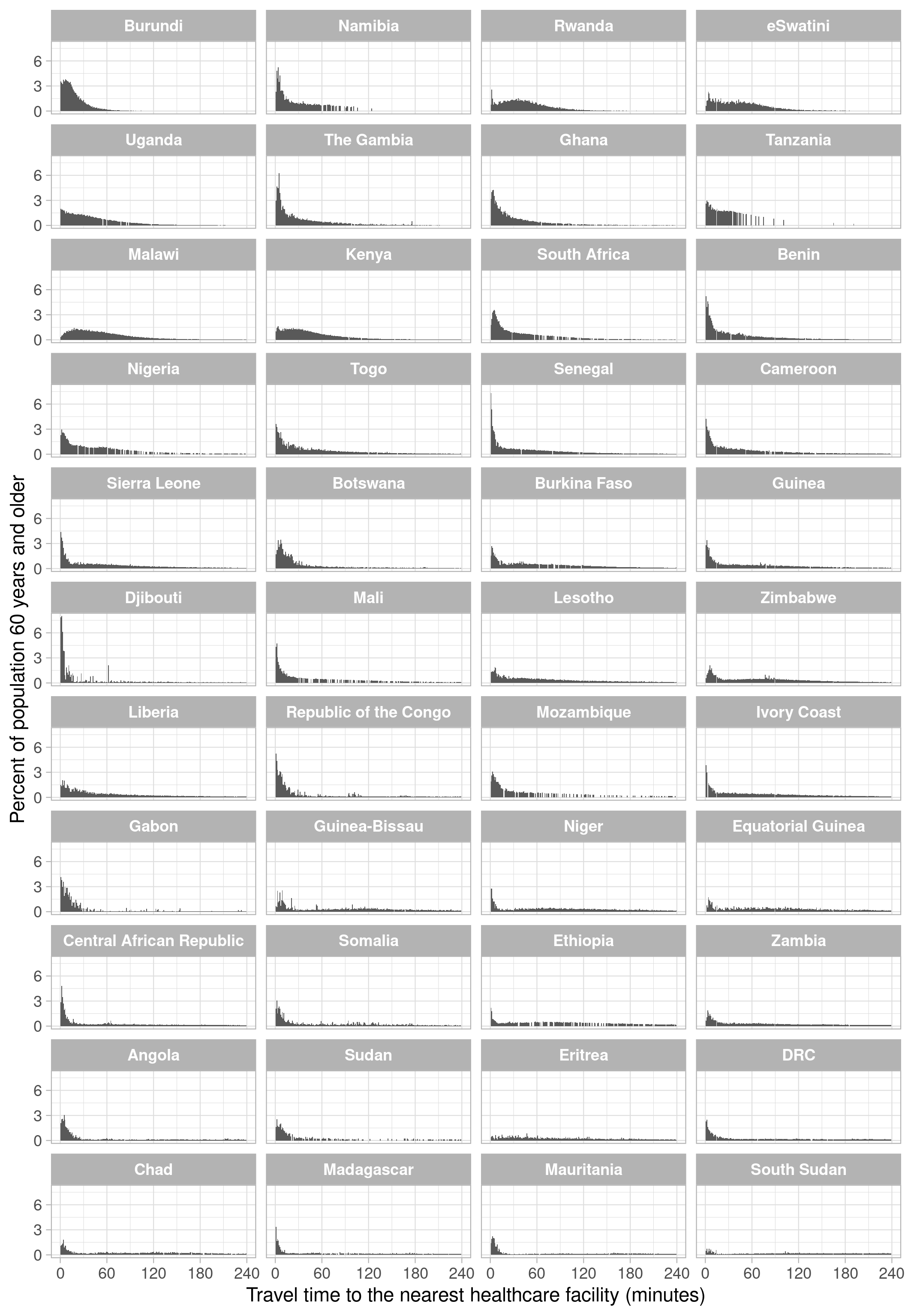
Abbreviations: DRC=Democratic Republic of the Congo
^1^ Countries were ordered in ascending order by the proportion of adults aged 60 years and older in their population who reside in a 1km x 1km area that has an estimated travel time >2 hours to the nearest healthcare facility.

# **Figure S7. Angola map of travel time to the nearest hospital for adults aged ≥ 60 years**


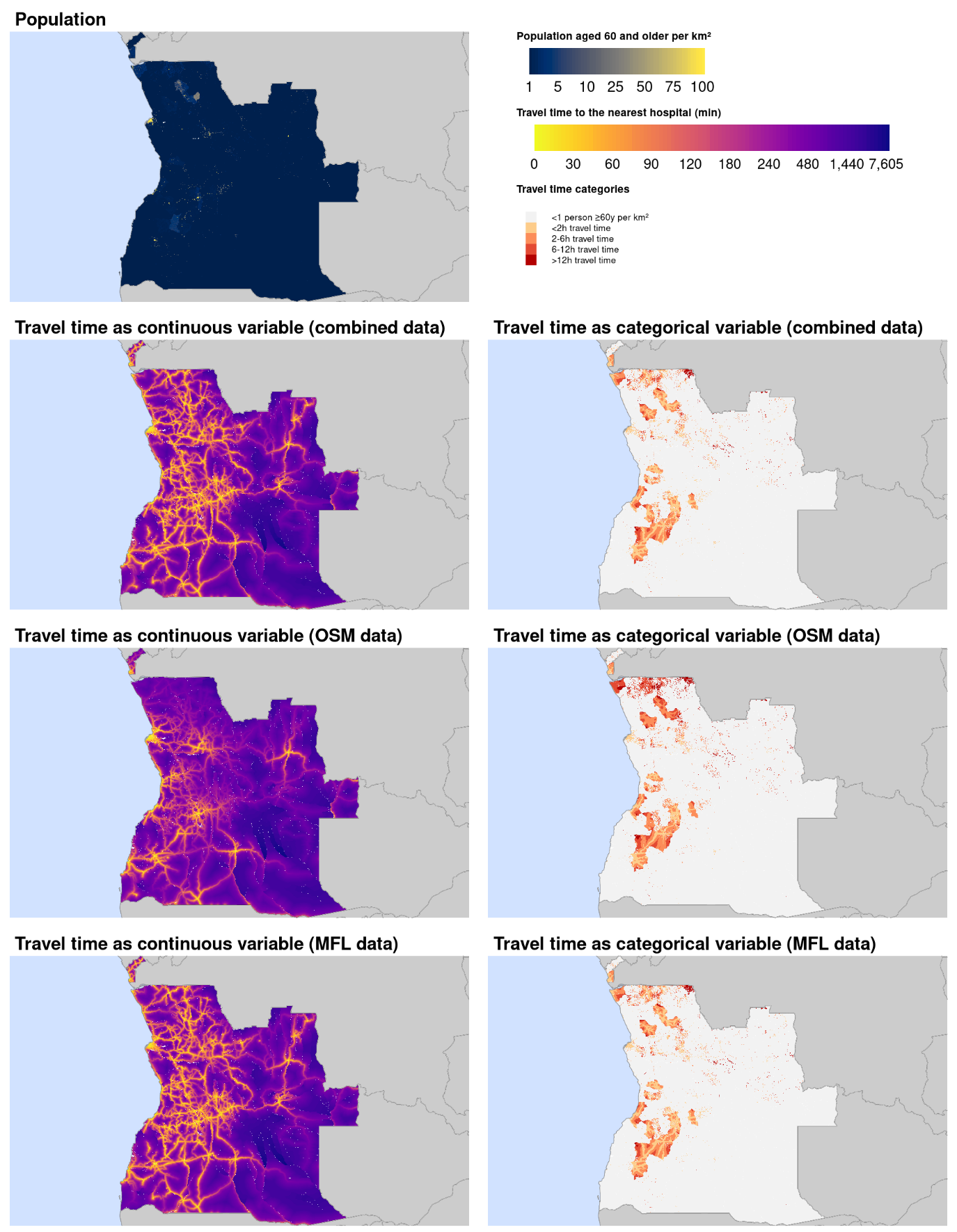


# **Figure S8. Benin map of travel time to the nearest hospital for adults aged ≥ 60 years**


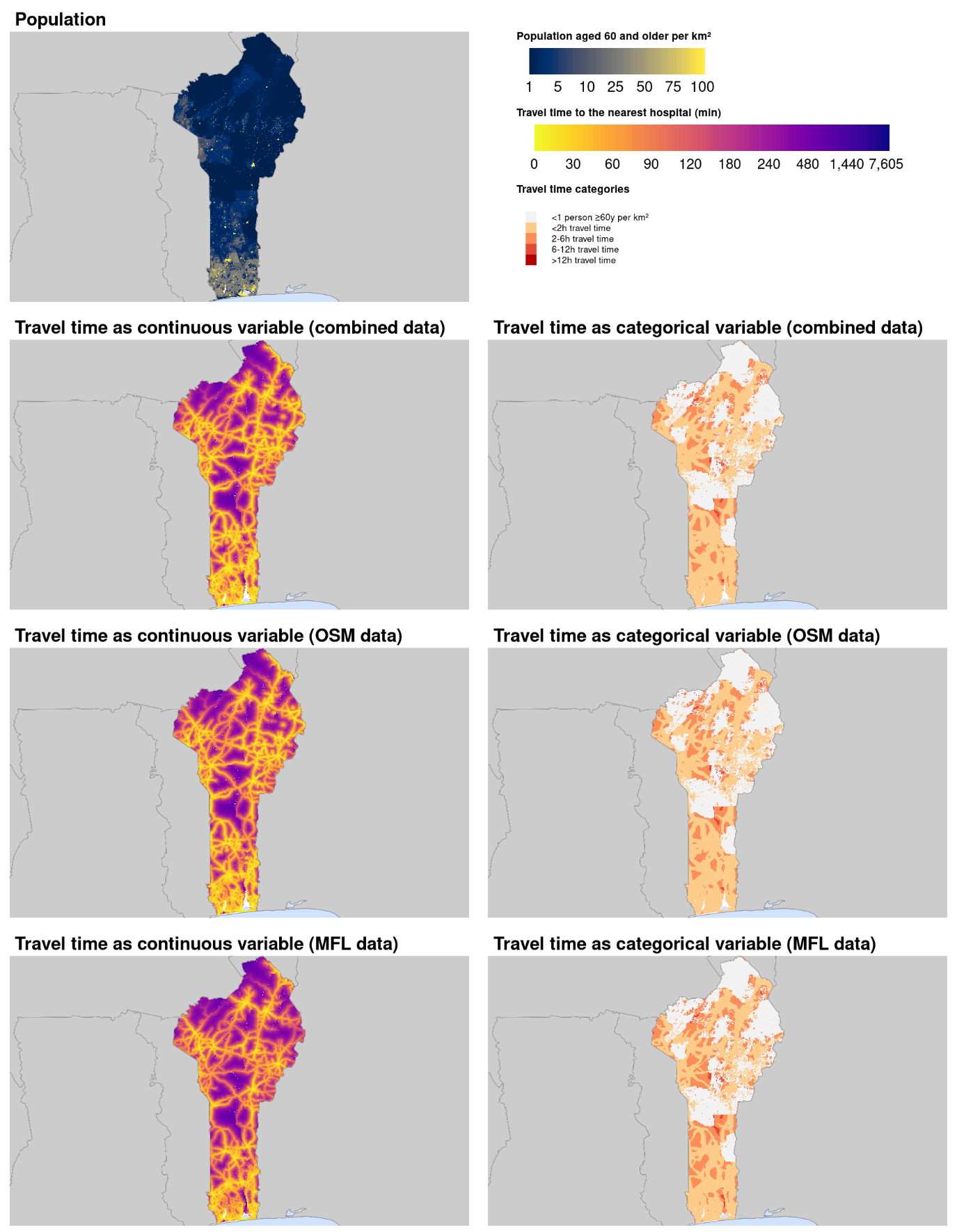


# **Figure S9. Botswana map of travel time to the nearest hospital for adults aged ≥ 60 years**


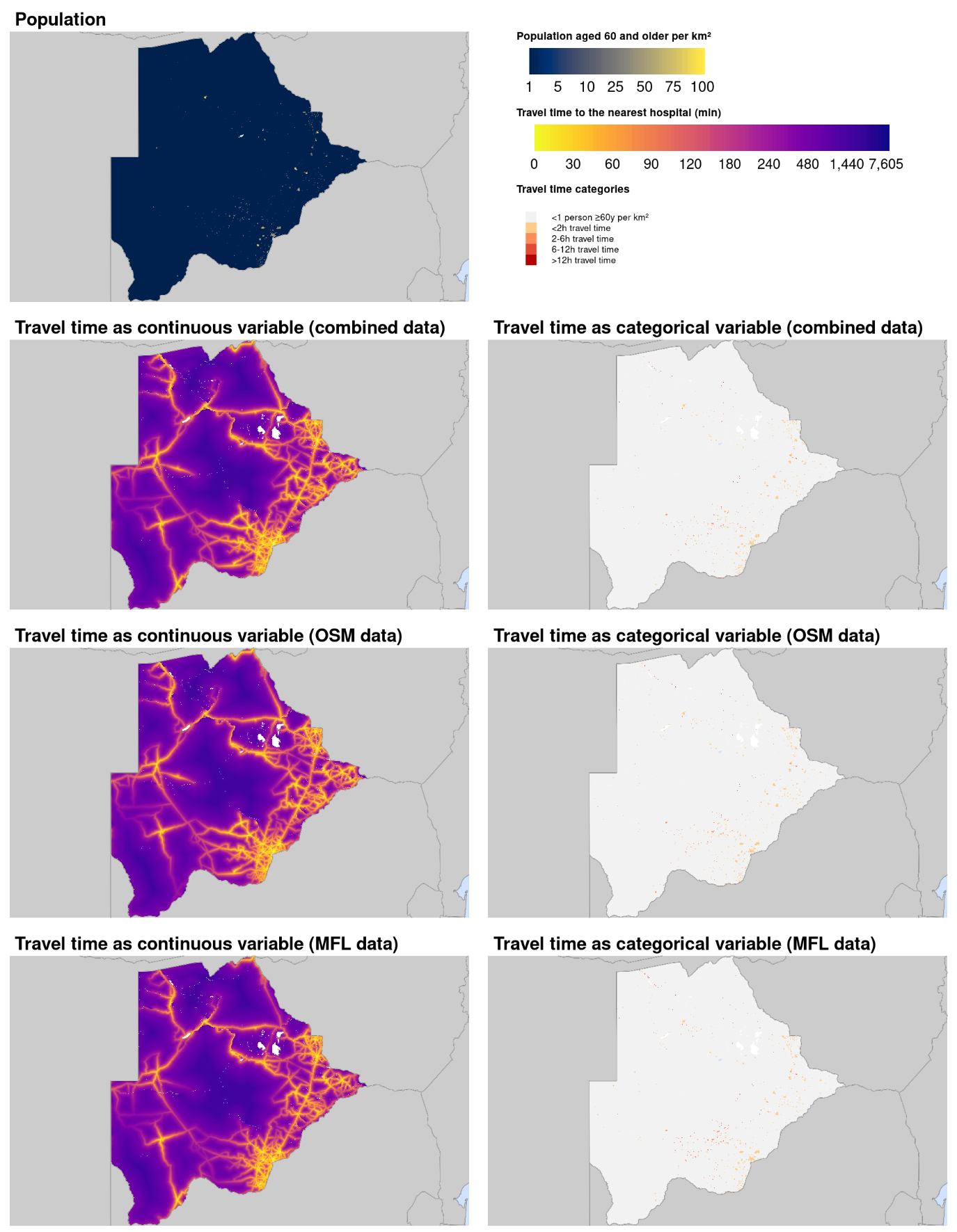


# **Figure S10. Burkina Faso map of travel time to the nearest hospital for adults aged ≥ 60 years**


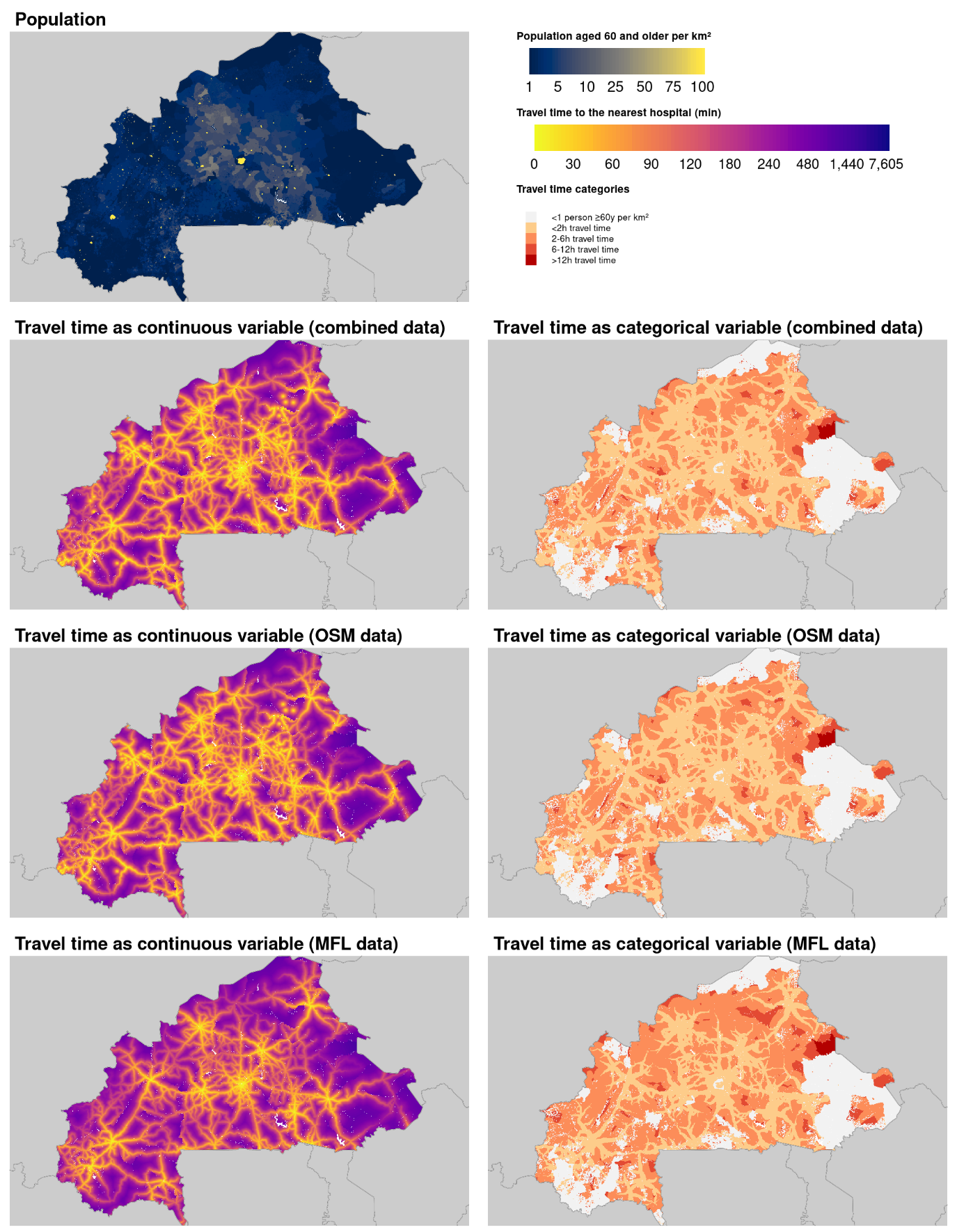


# **Figure S11. Burundi map of travel time to the nearest hospital for adults aged ≥ 60 years**


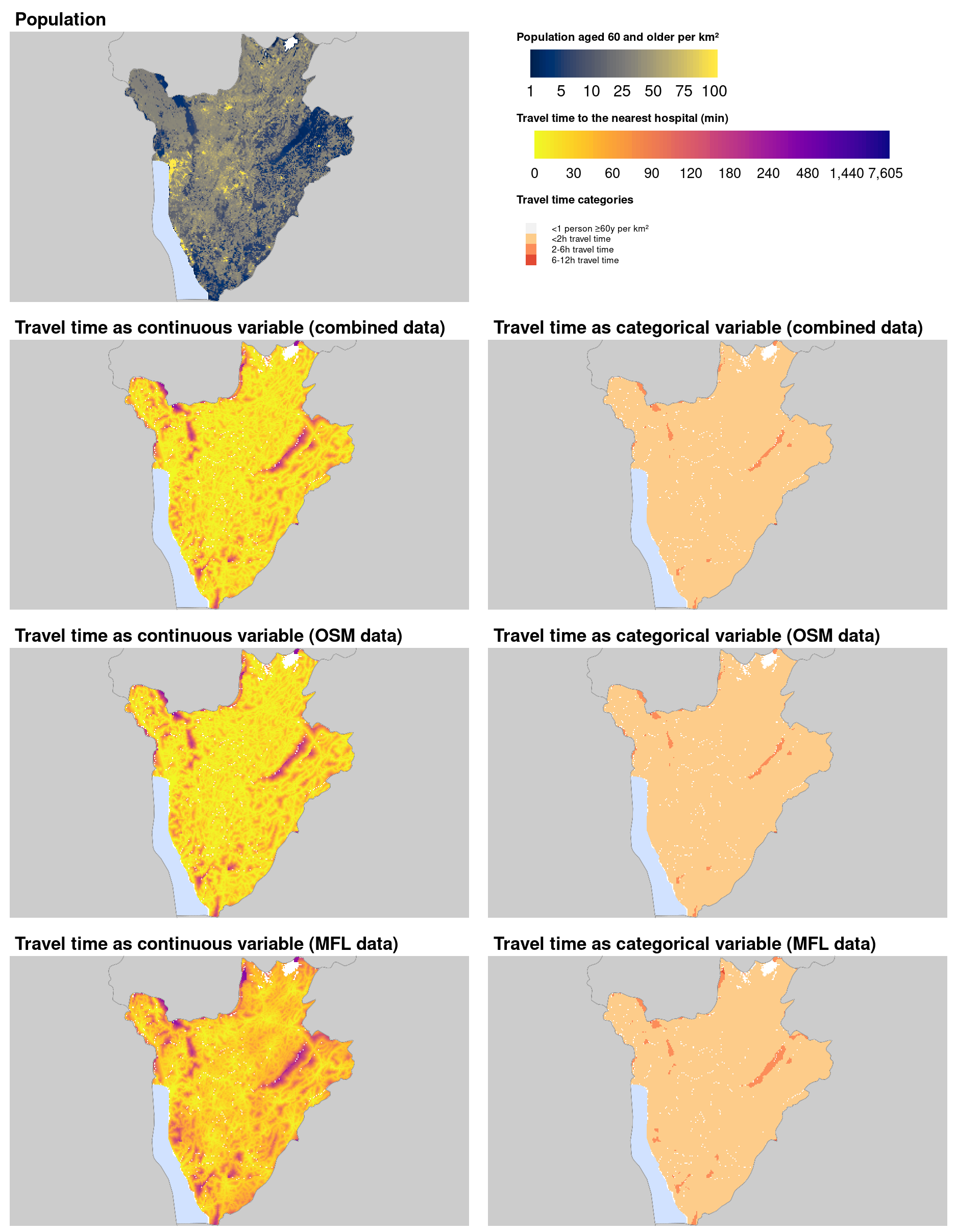


# **Figure S12. Cameroon map of travel time to the nearest hospital for adults aged ≥ 60 years**


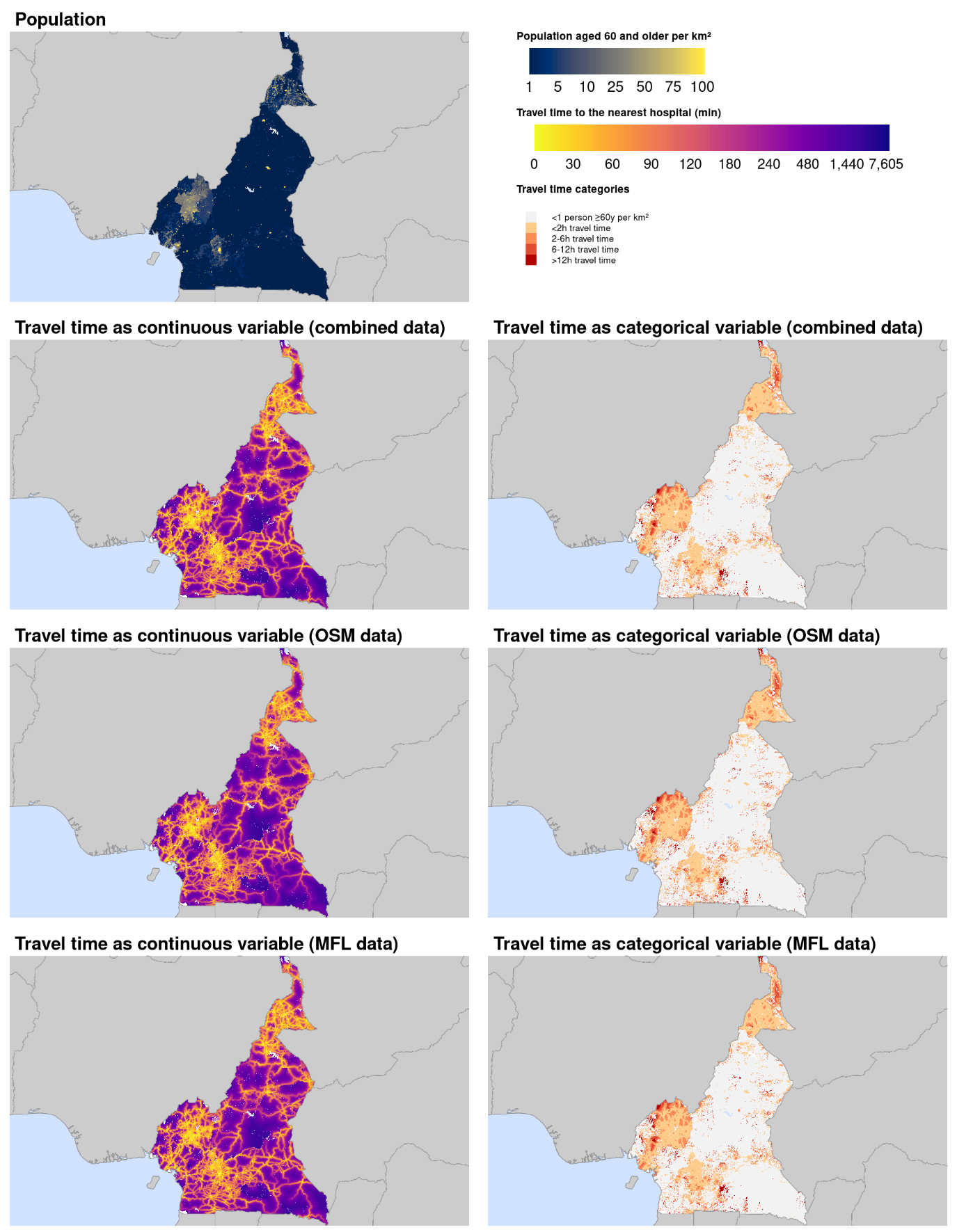


# **Figure S13. Central African Republic map of travel time to the nearest hospital for adults aged ≥ 60 years**


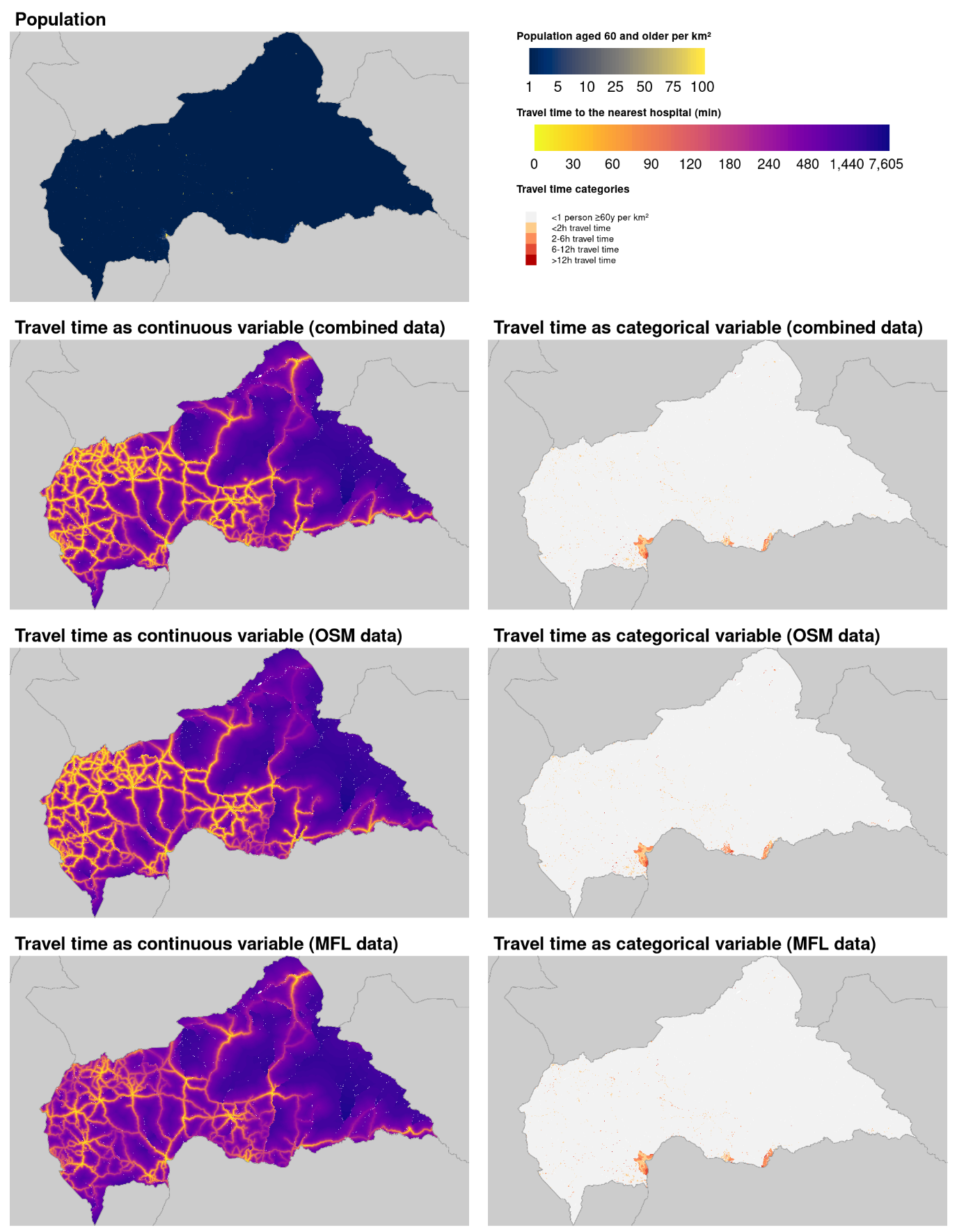


# **Figure S14. Chad map of travel time to the nearest hospital for adults aged ≥ 60 years**


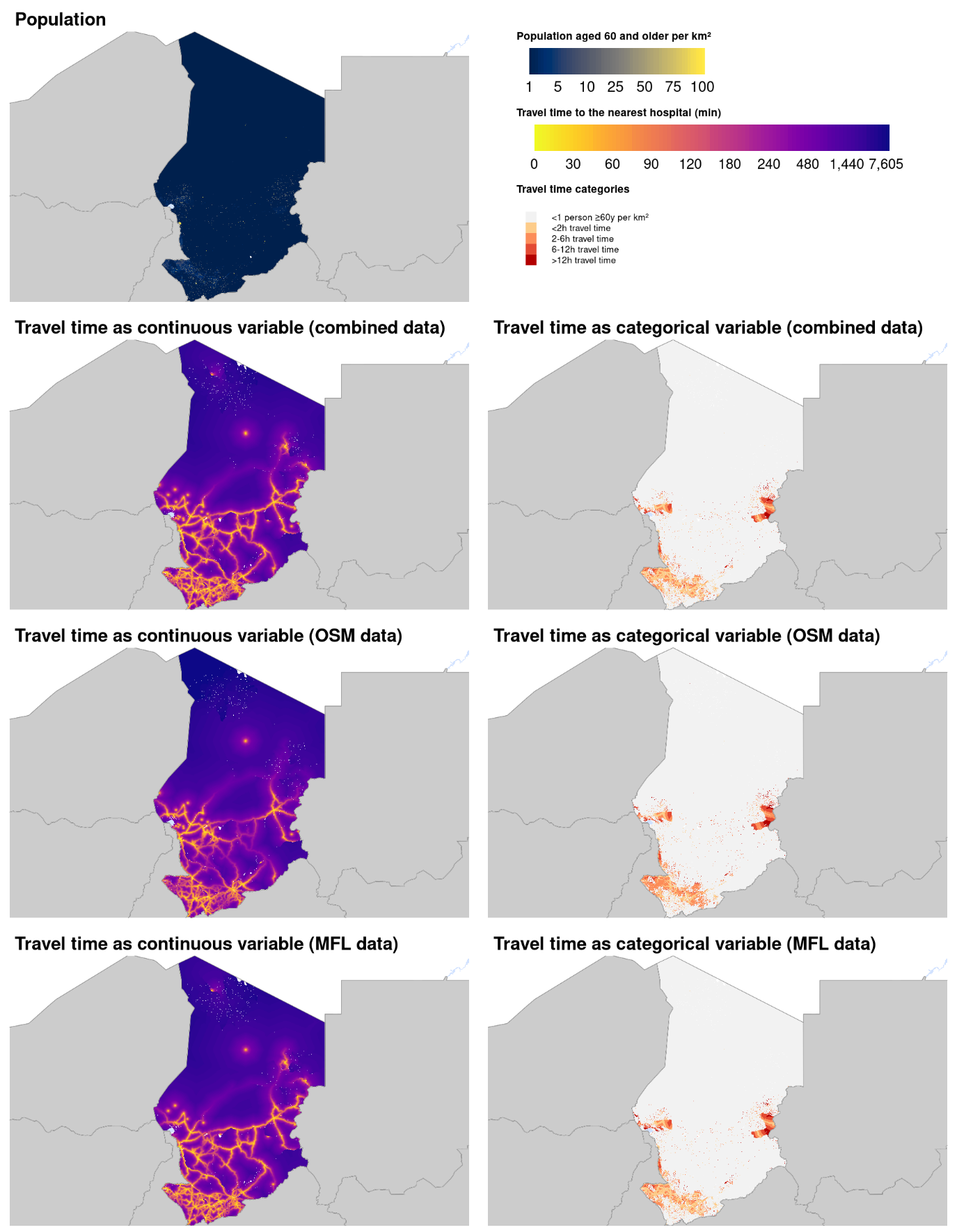


# **Figure S15. Djibouti map of travel time to the nearest hospital for adults aged ≥ 60 years**


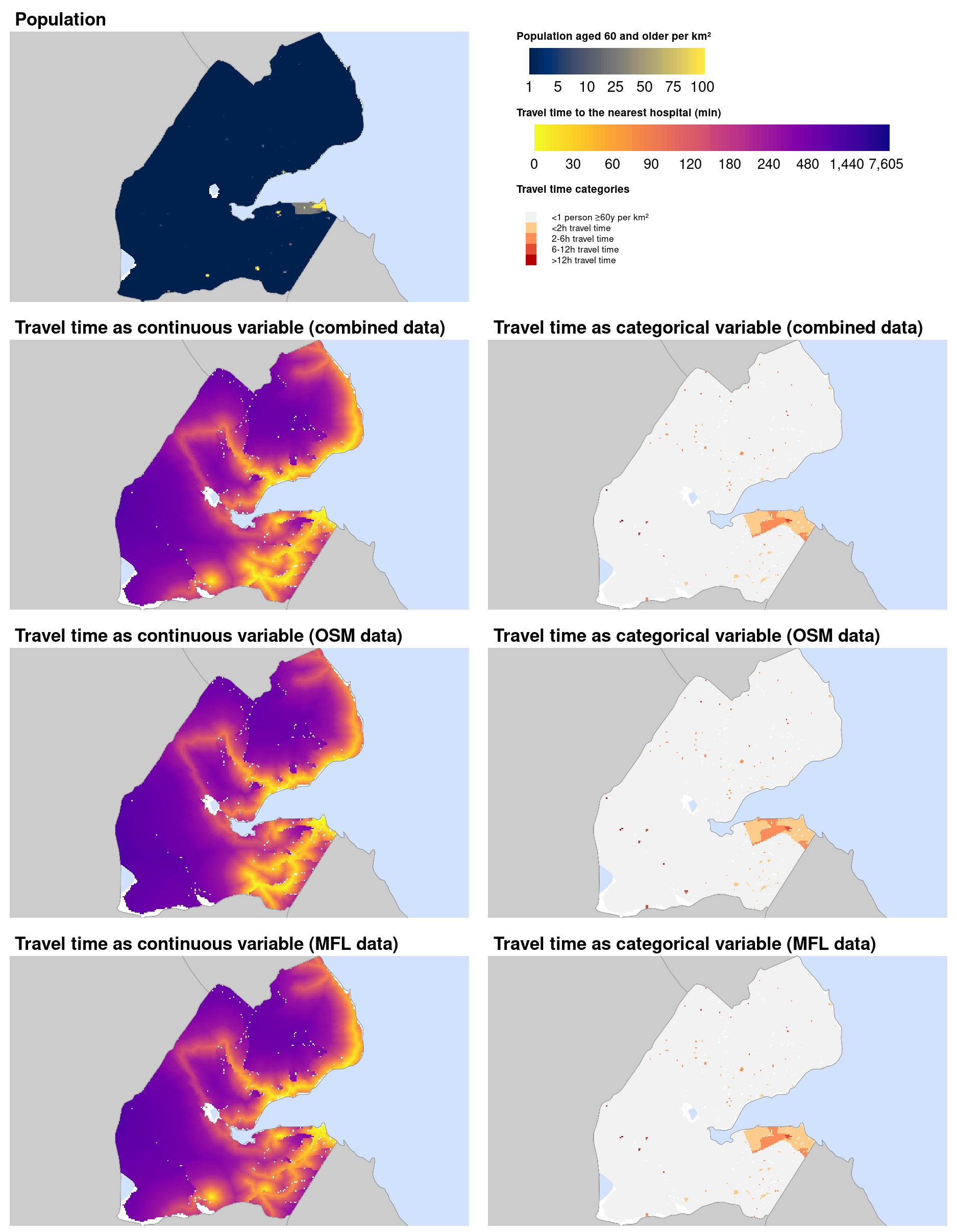


# **Figure S16. DRC map of travel time to the nearest hospital for adults aged ≥ 60 years**


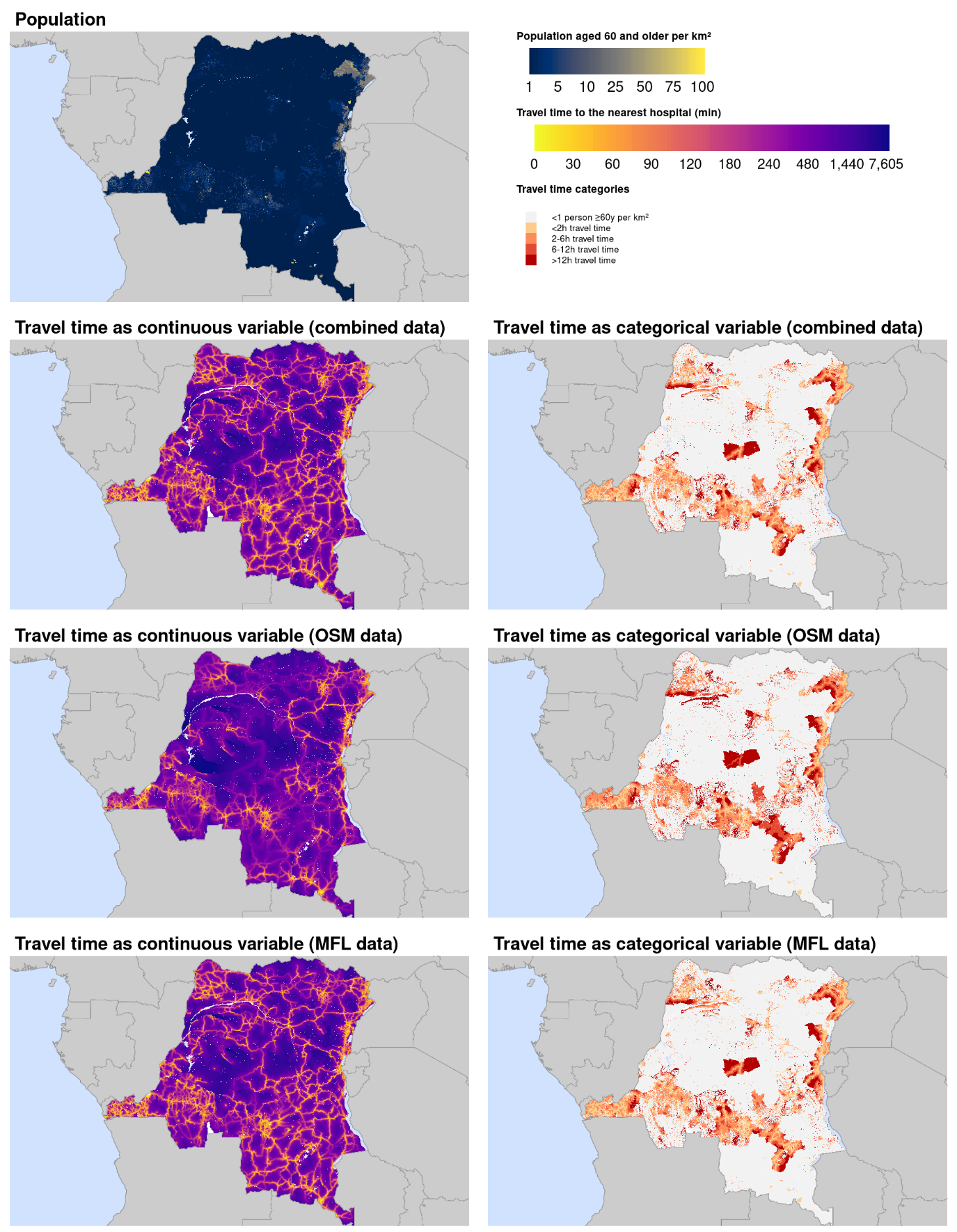


# **Figure S17. Equatorial Guinea map of travel time to the nearest hospital for adults aged ≥ 60 years**


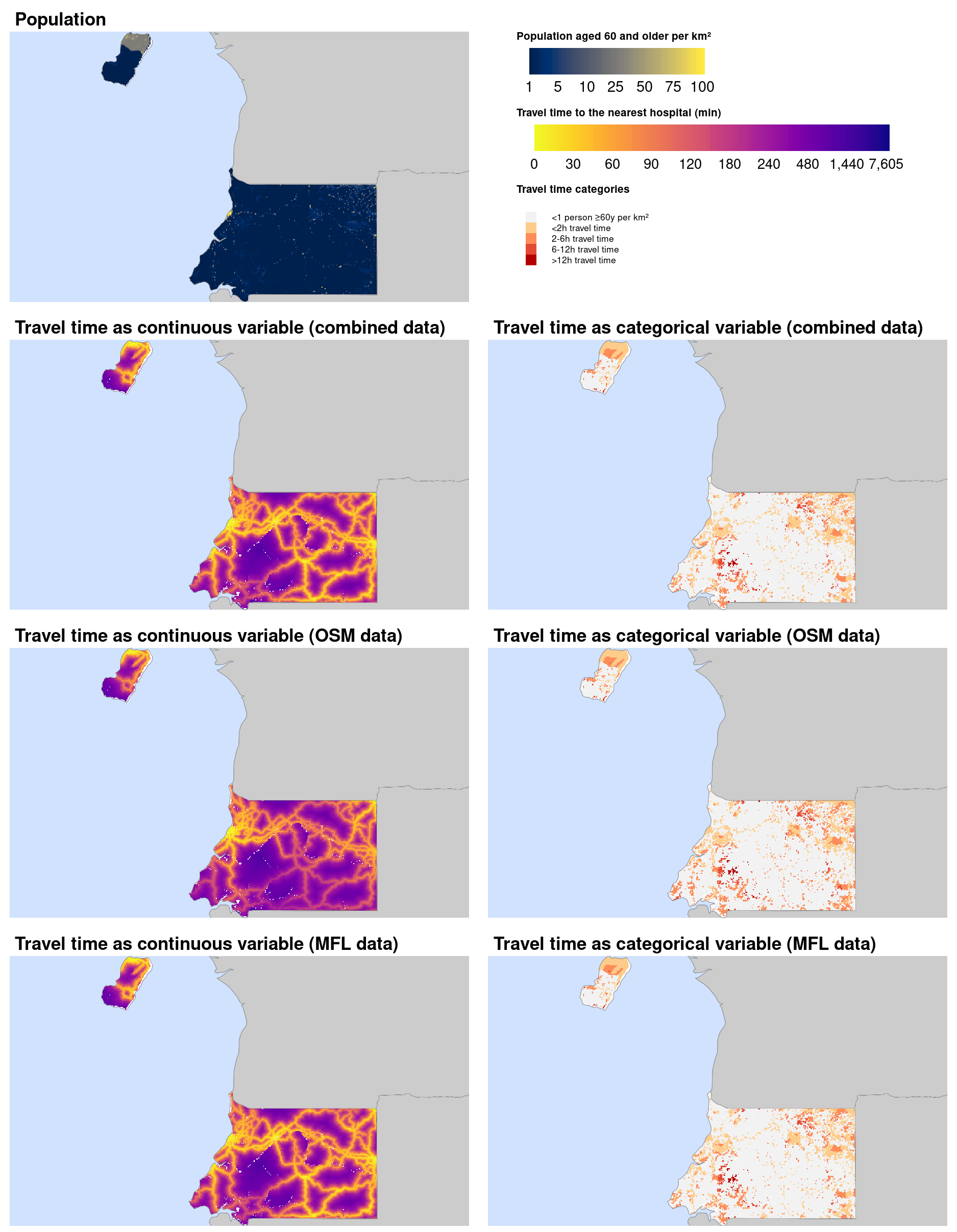


# **Figure S18. Eritrea map of travel time to the nearest hospital for adults aged ≥ 60 years**


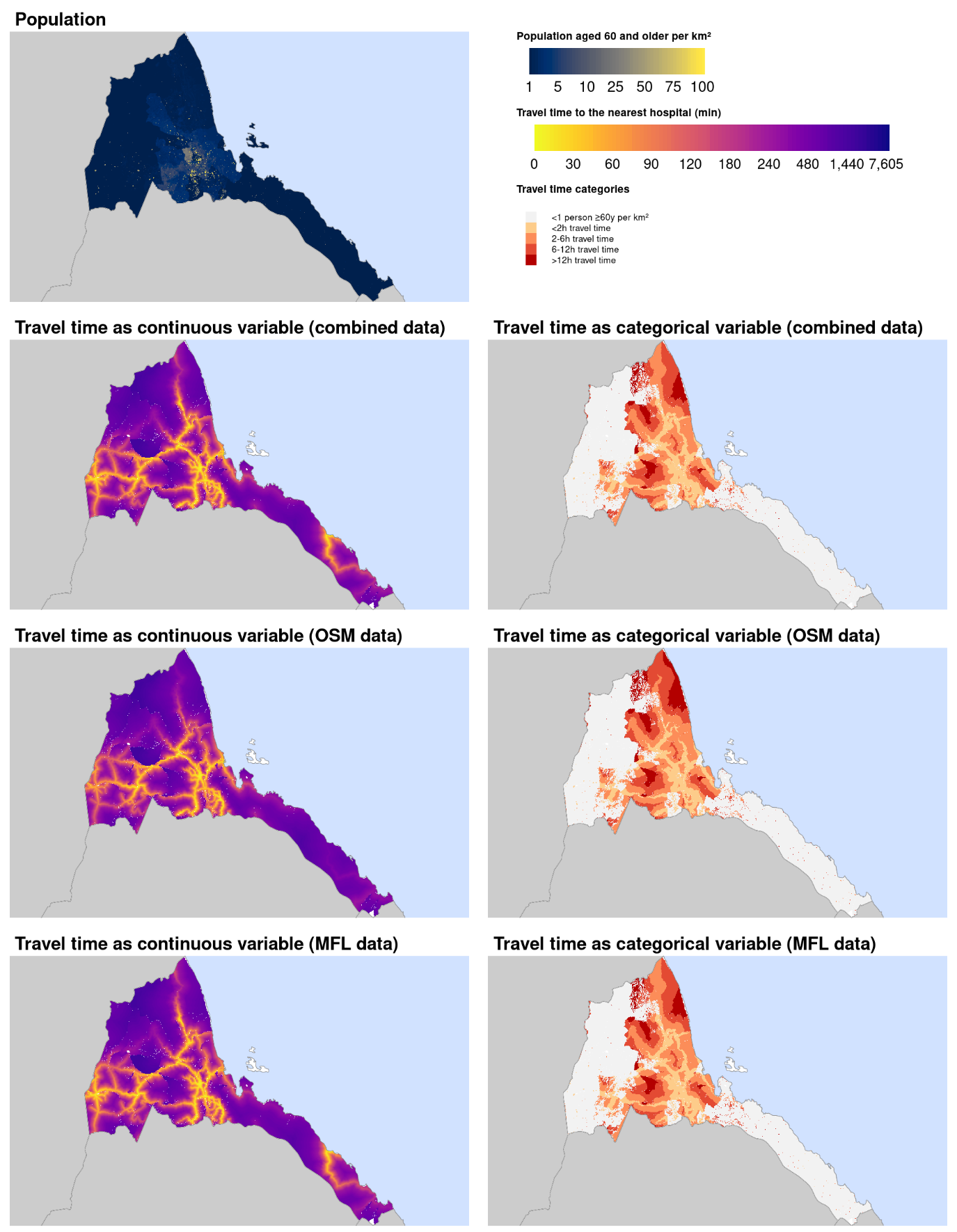


# **Figure S19. eSwatini map of travel time to the nearest hospital for adults aged ≥ 60 years**


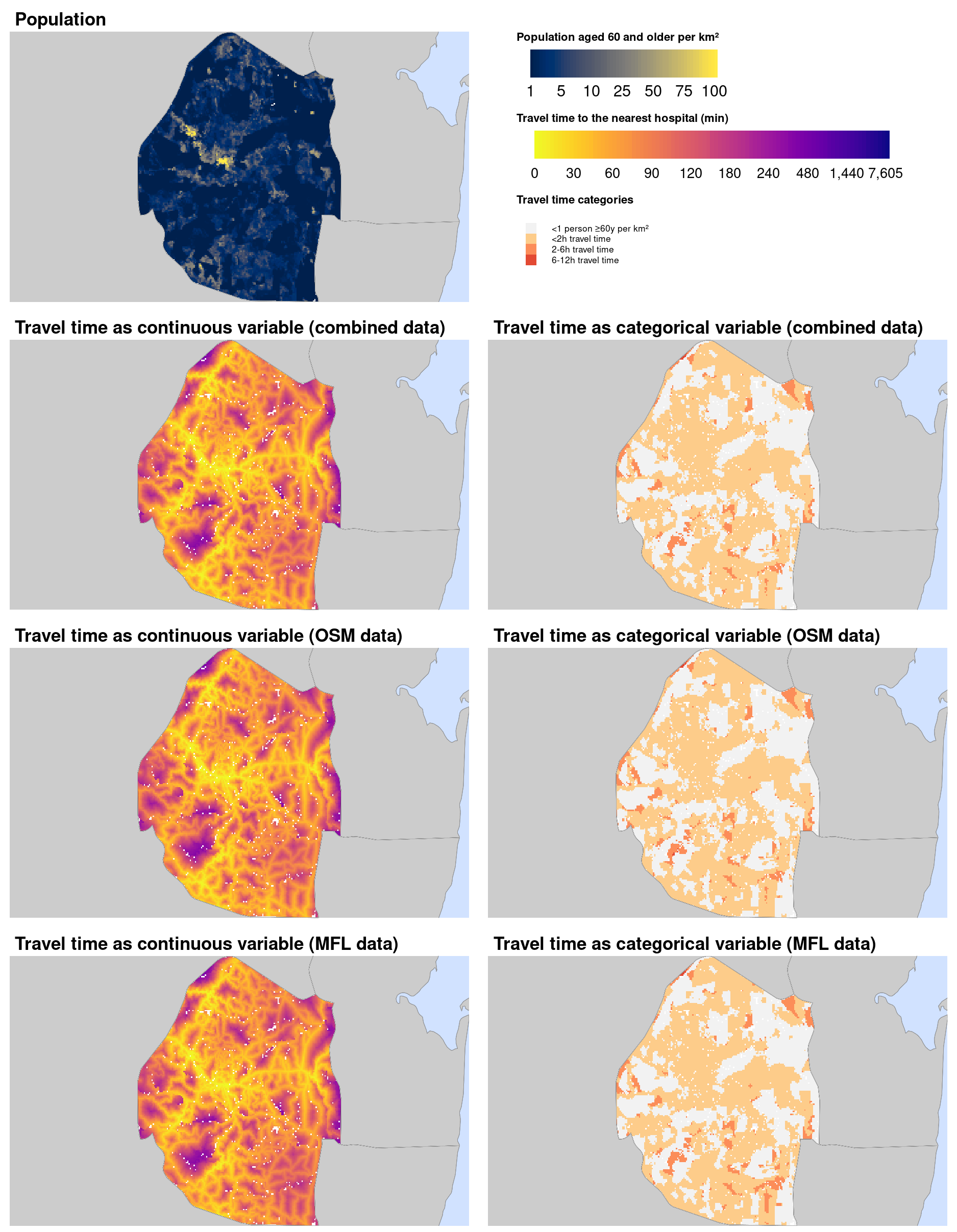


# **Figure S20. Ethiopia map of travel time to the nearest hospital for adults aged ≥ 60 years**


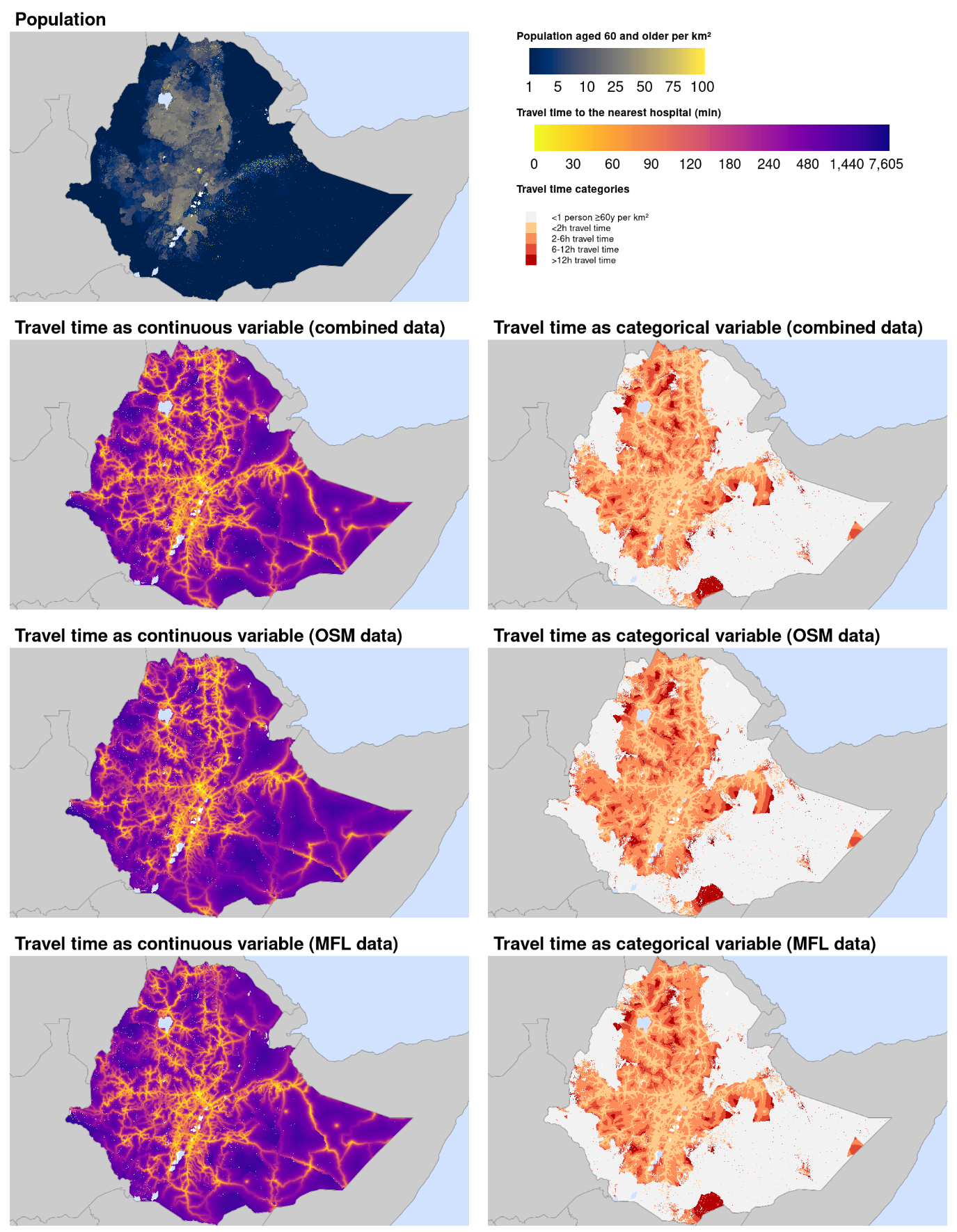


# **Figure S21. Gabon map of travel time to the nearest hospital for adults aged ≥ 60 years**


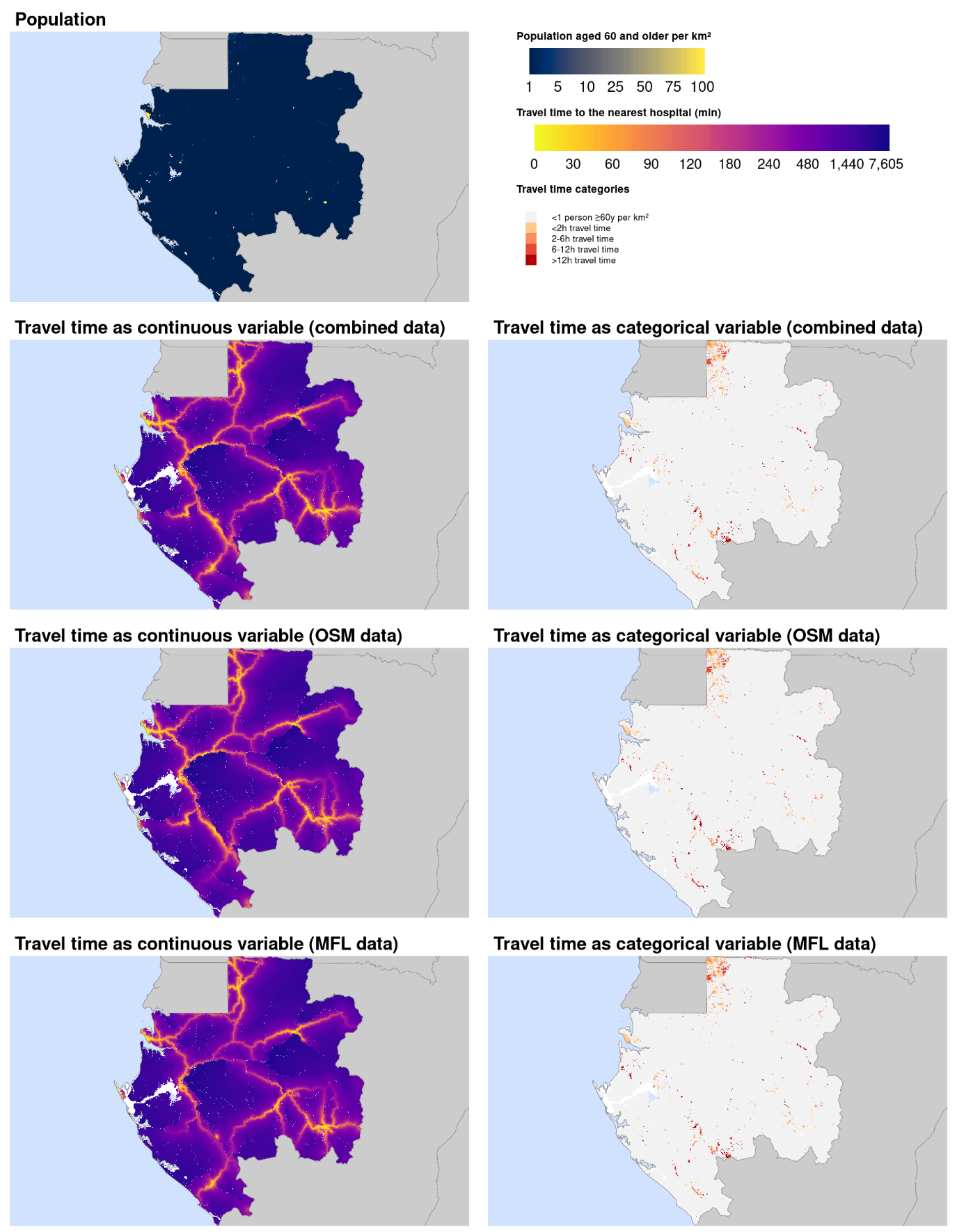


# **Figure S22. Ghana map of travel time to the nearest hospital for adults aged ≥ 60 years**


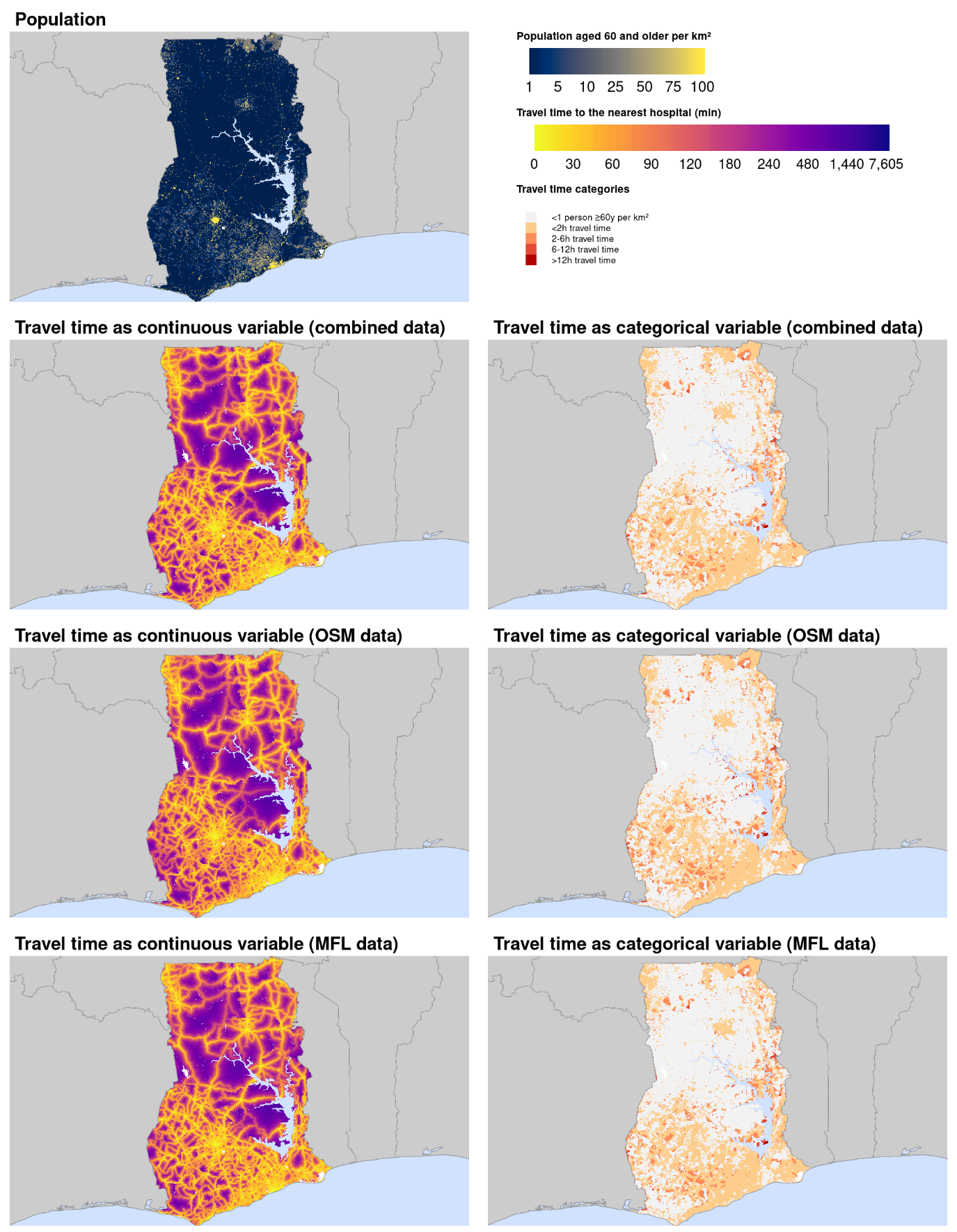


# **Figure S23. Guinea map of travel time to the nearest hospital for adults aged ≥ 60 years**


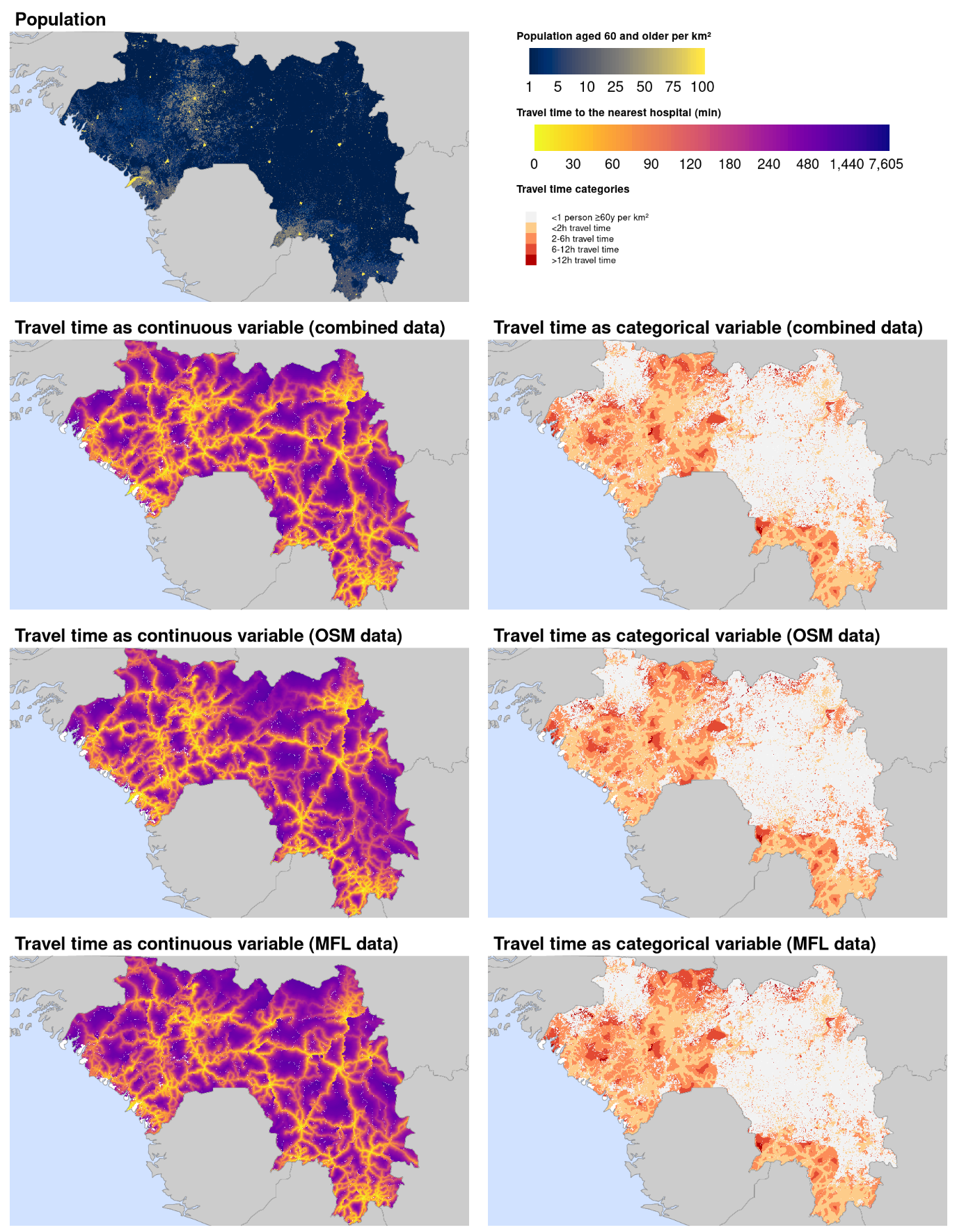


# **Figure S24. Guinea-Bissau map of travel time to the nearest hospital for adults aged ≥ 60 years**


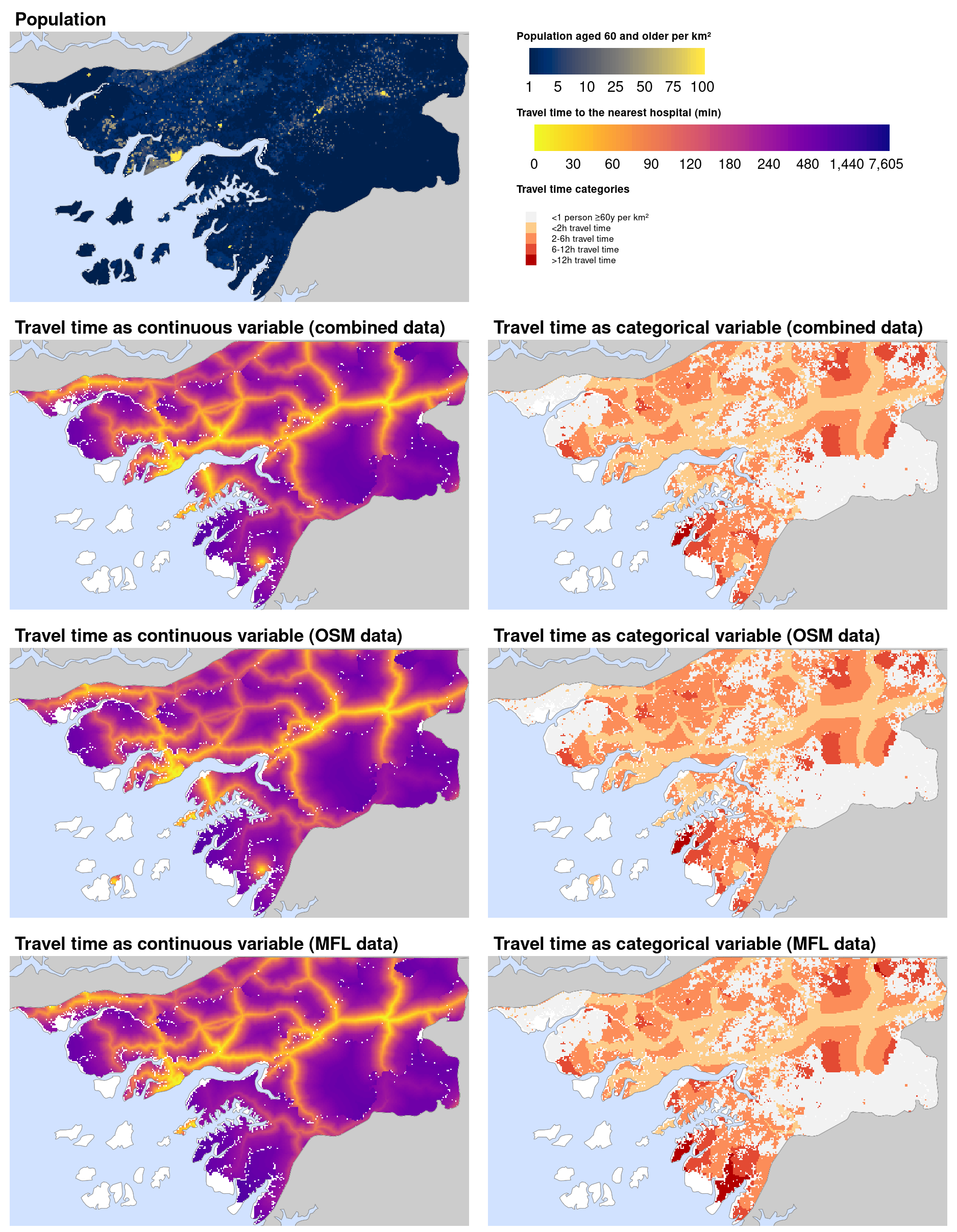


# **Figure S25. Ivory Coast map of travel time to the nearest hospital for adults aged ≥ 60 years**


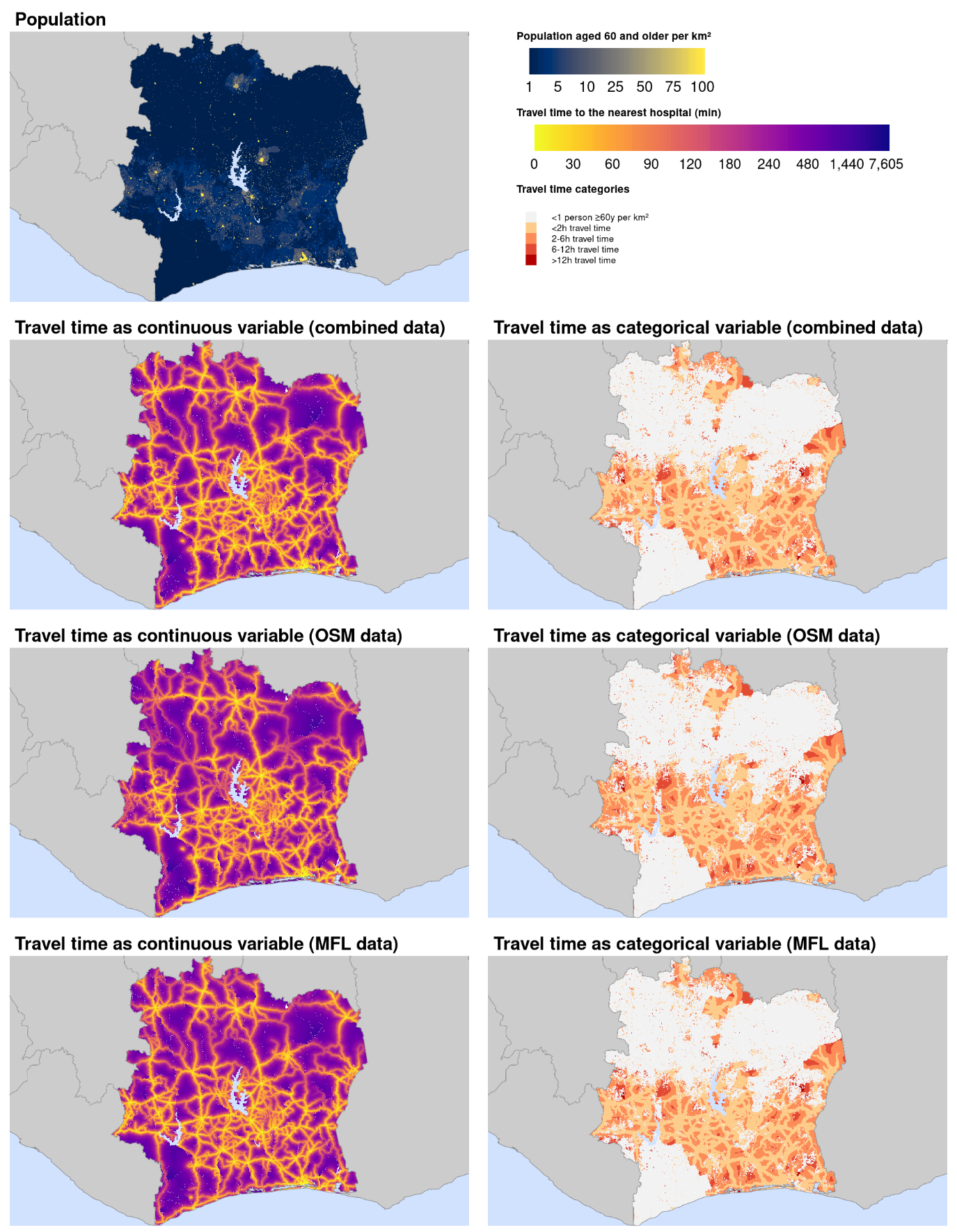


# **Figure S26. Kenya map of travel time to the nearest hospital for adults aged ≥ 60 years**


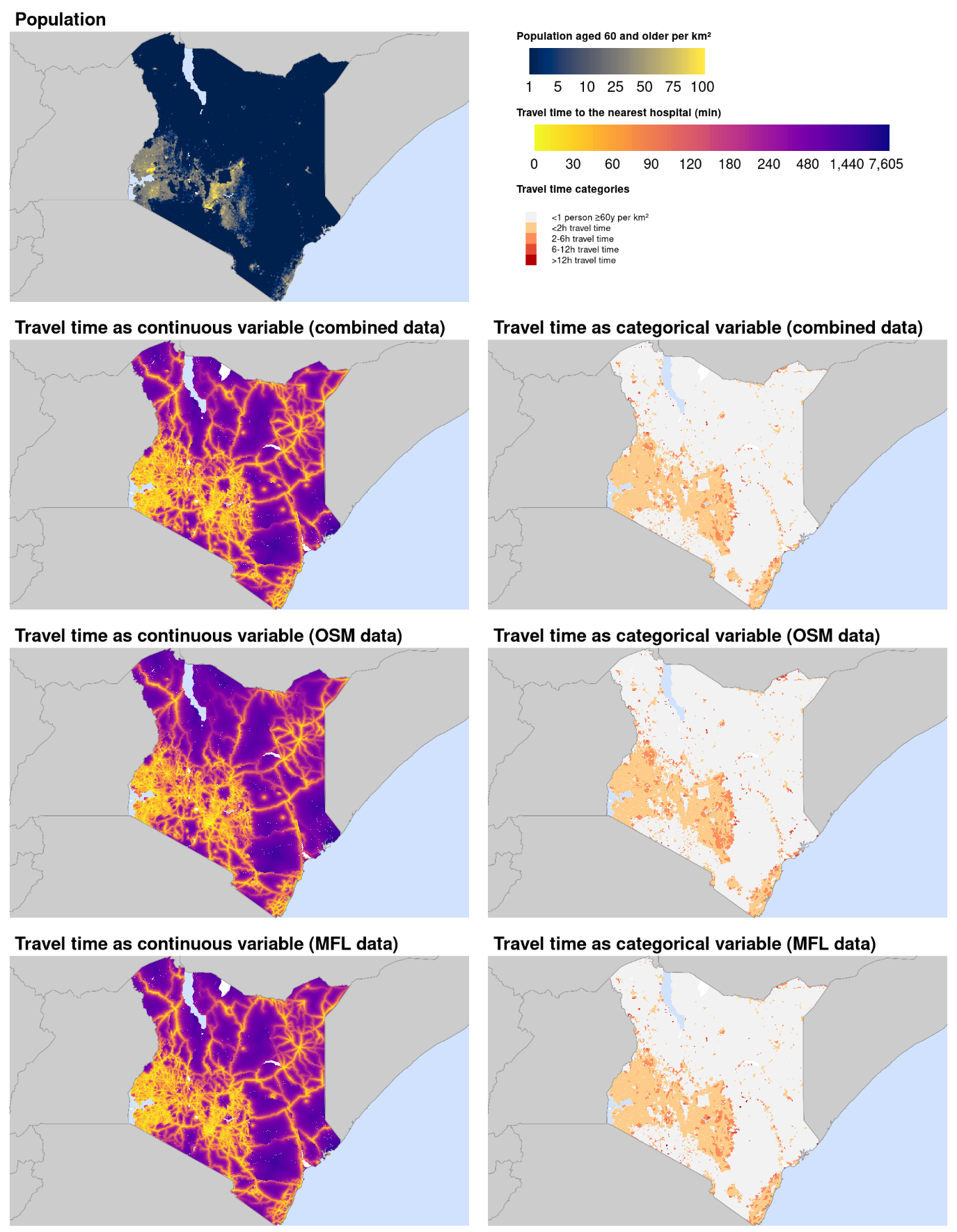


# **Figure S27. Lesotho map of travel time to the nearest hospital for adults aged ≥ 60 years**


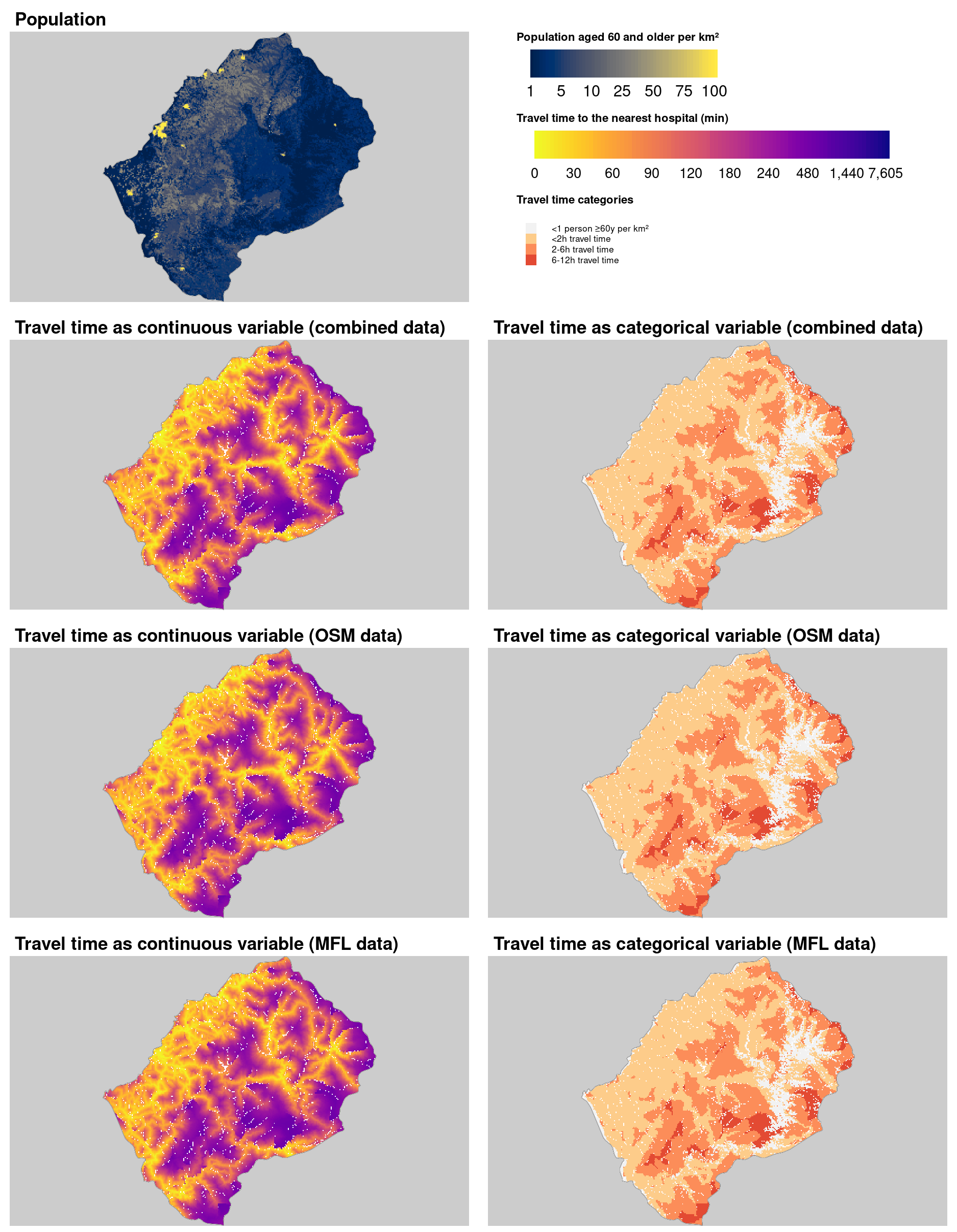


# **Figure S28. Liberia map of travel time to the nearest hospital for adults aged ≥ 60 years**


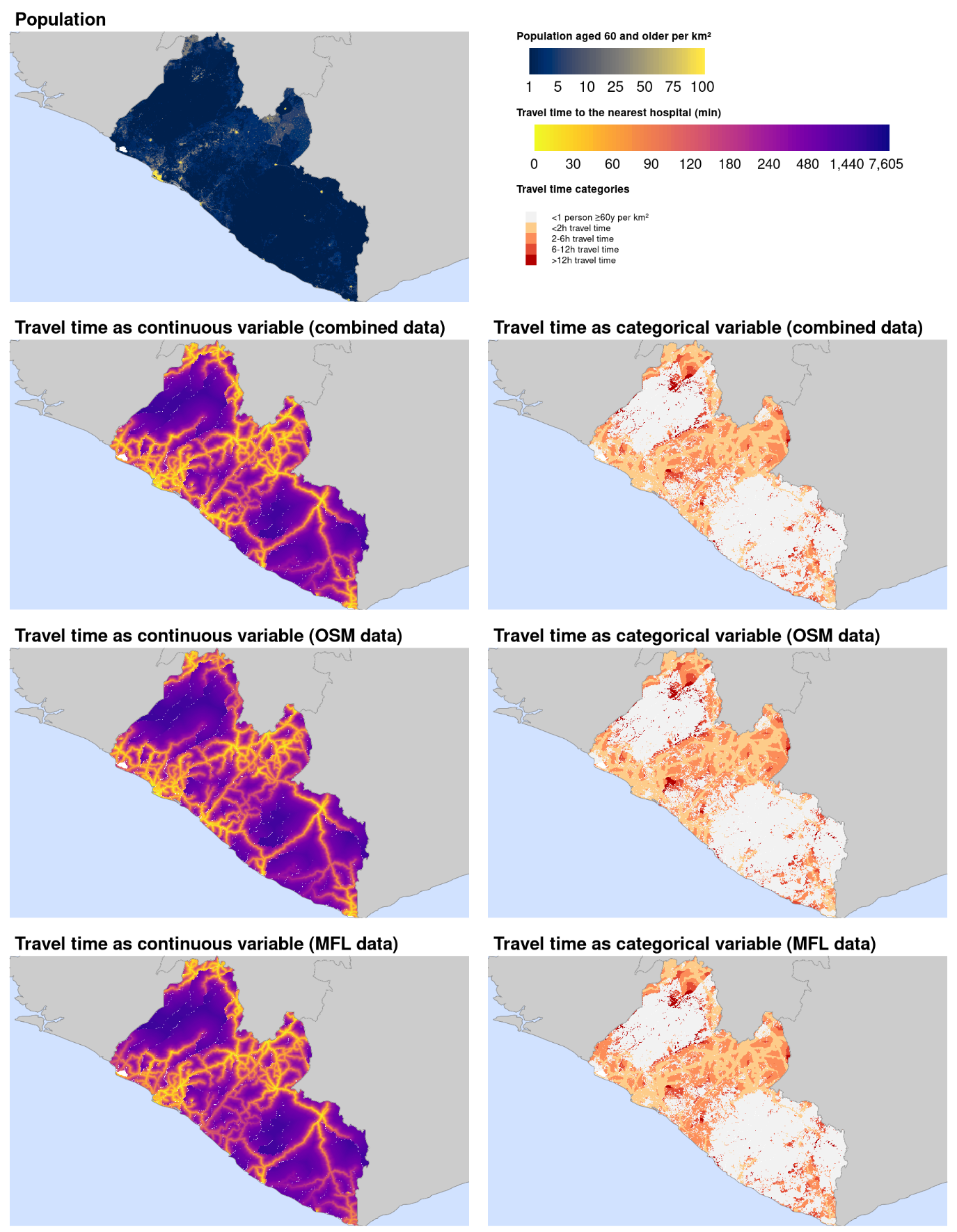


# **Figure S29. Madagascar map of travel time to the nearest hospital for adults aged ≥ 60 years**


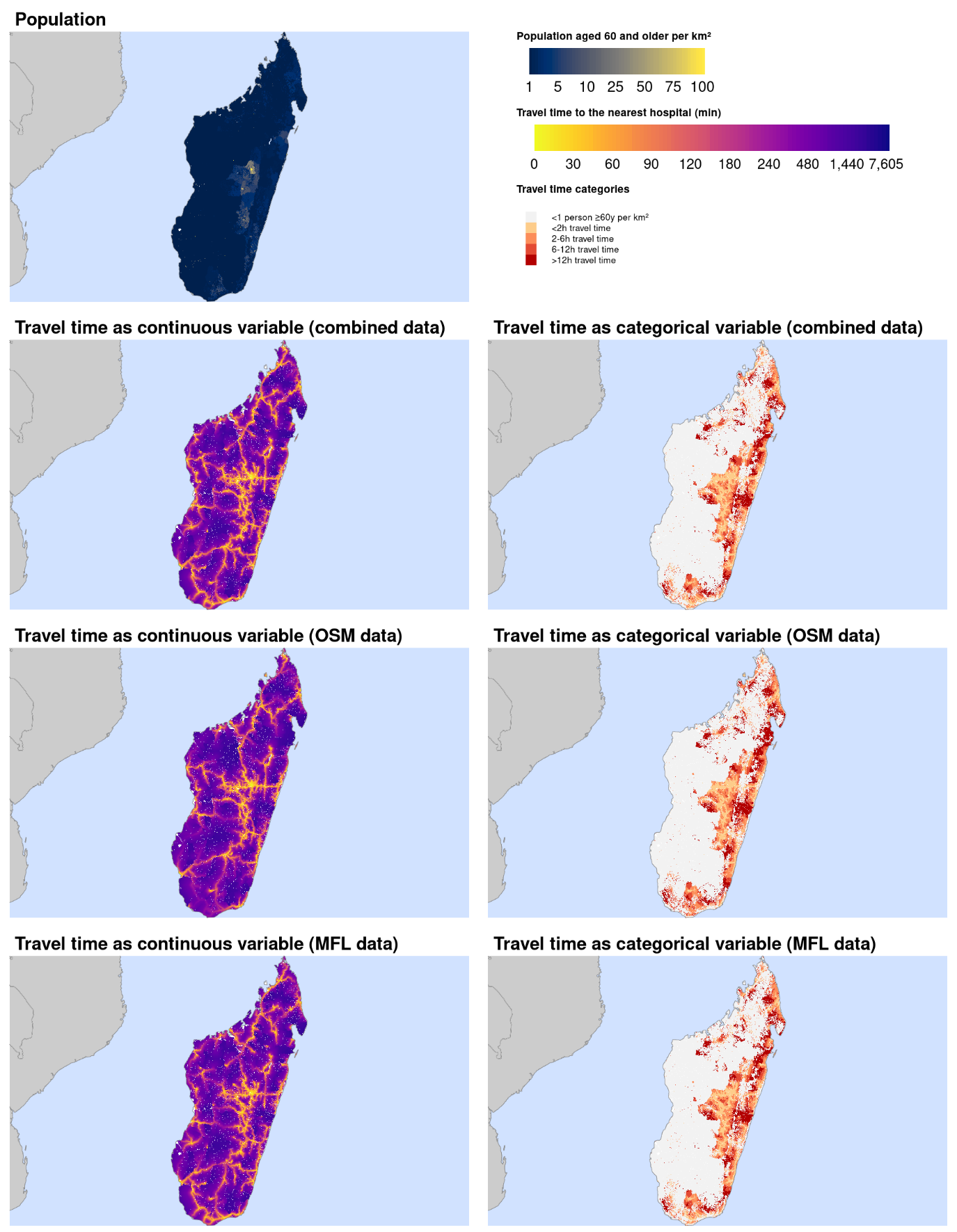


# **Figure S30. Malawi map of travel time to the nearest hospital for adults aged ≥ 60 years**


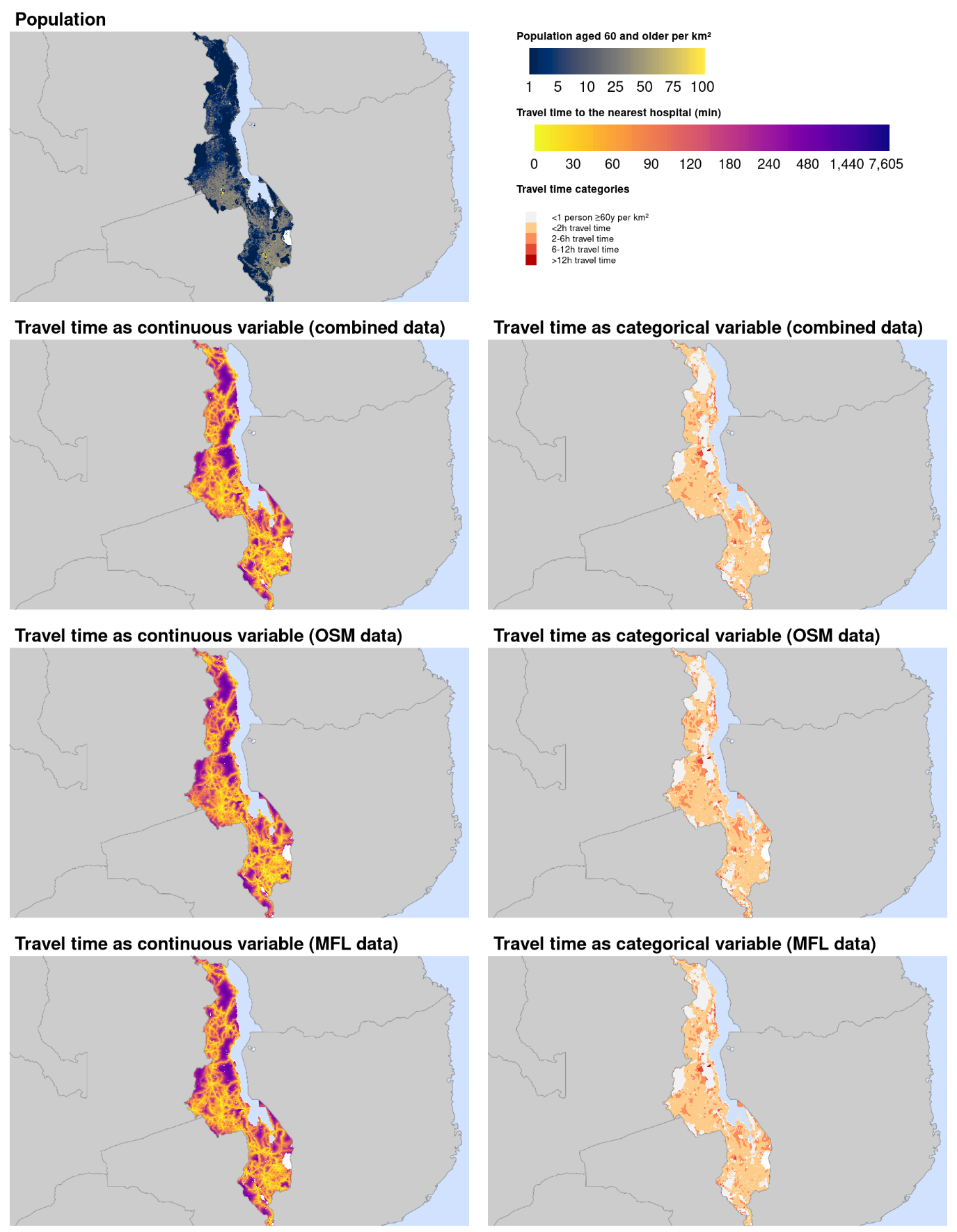


# **Figure S31. Mali map of travel time to the nearest hospital for adults aged ≥ 60 years**


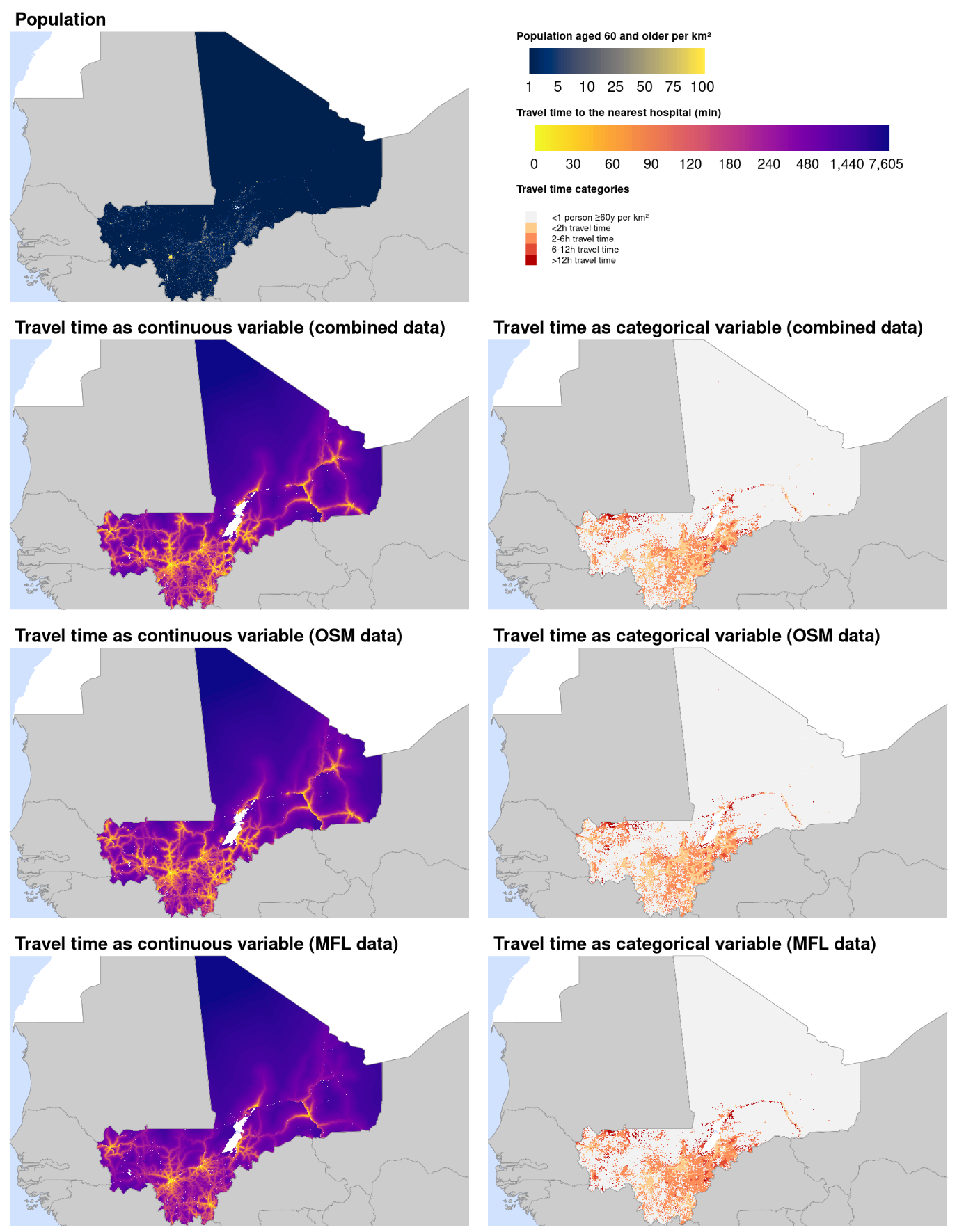


# **Figure S32. Mauritania map of travel time to the nearest hospital for adults aged ≥ 60 years**


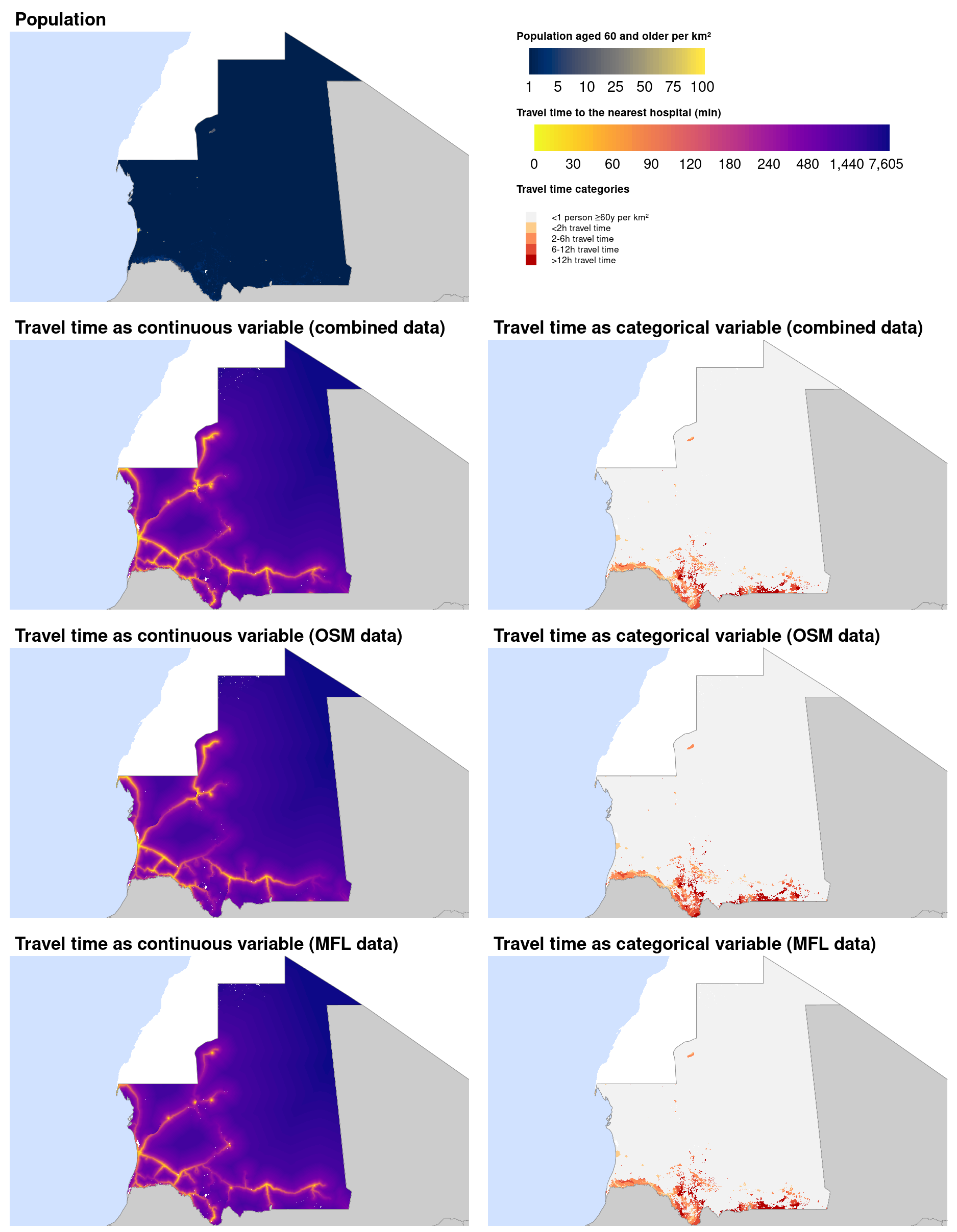


# **Figure S33. Mozambique map of travel time to the nearest hospital for adults aged ≥ 60 years**


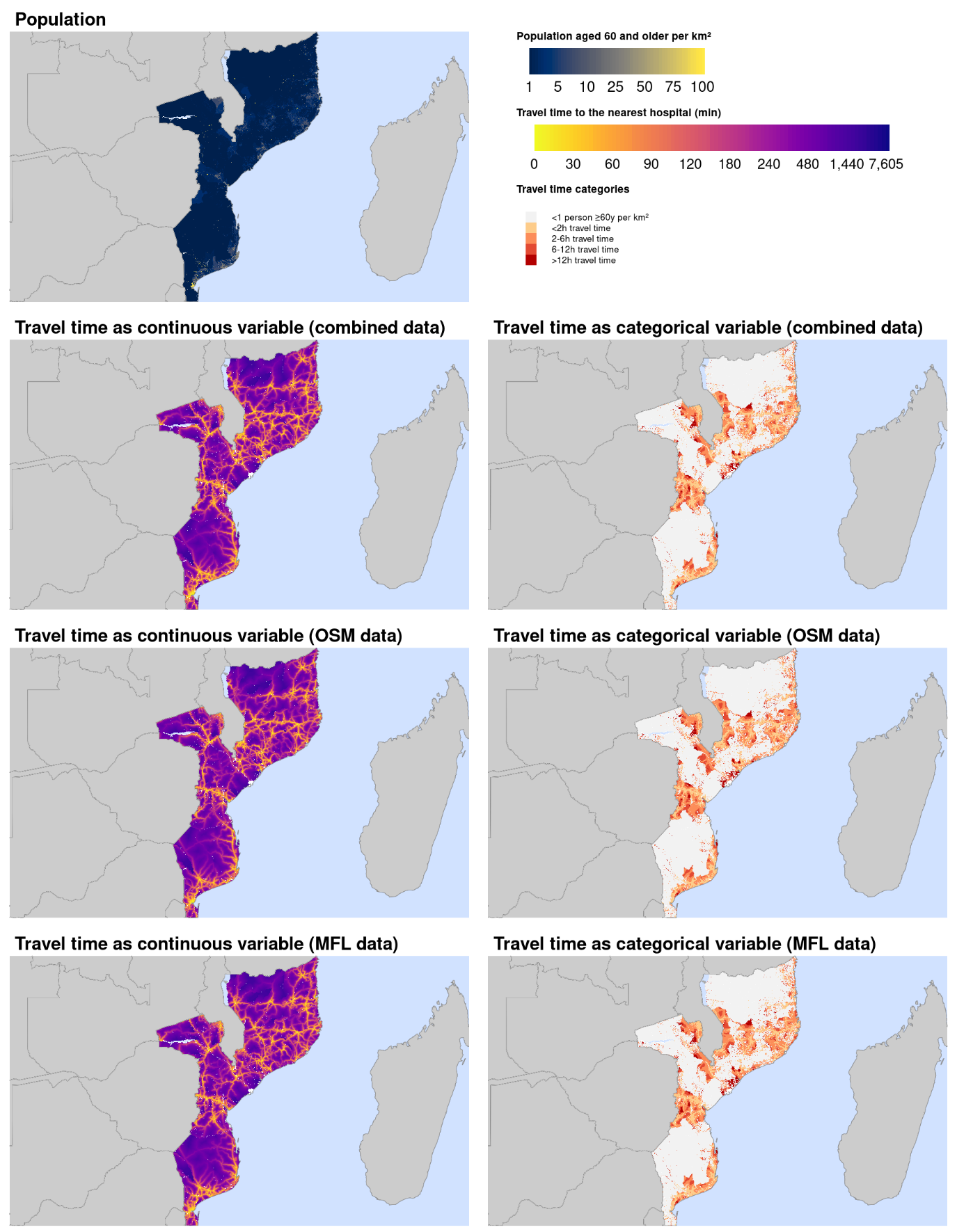


# **Figure S34. Namibia map of travel time to the nearest hospital for adults aged ≥ 60 years**


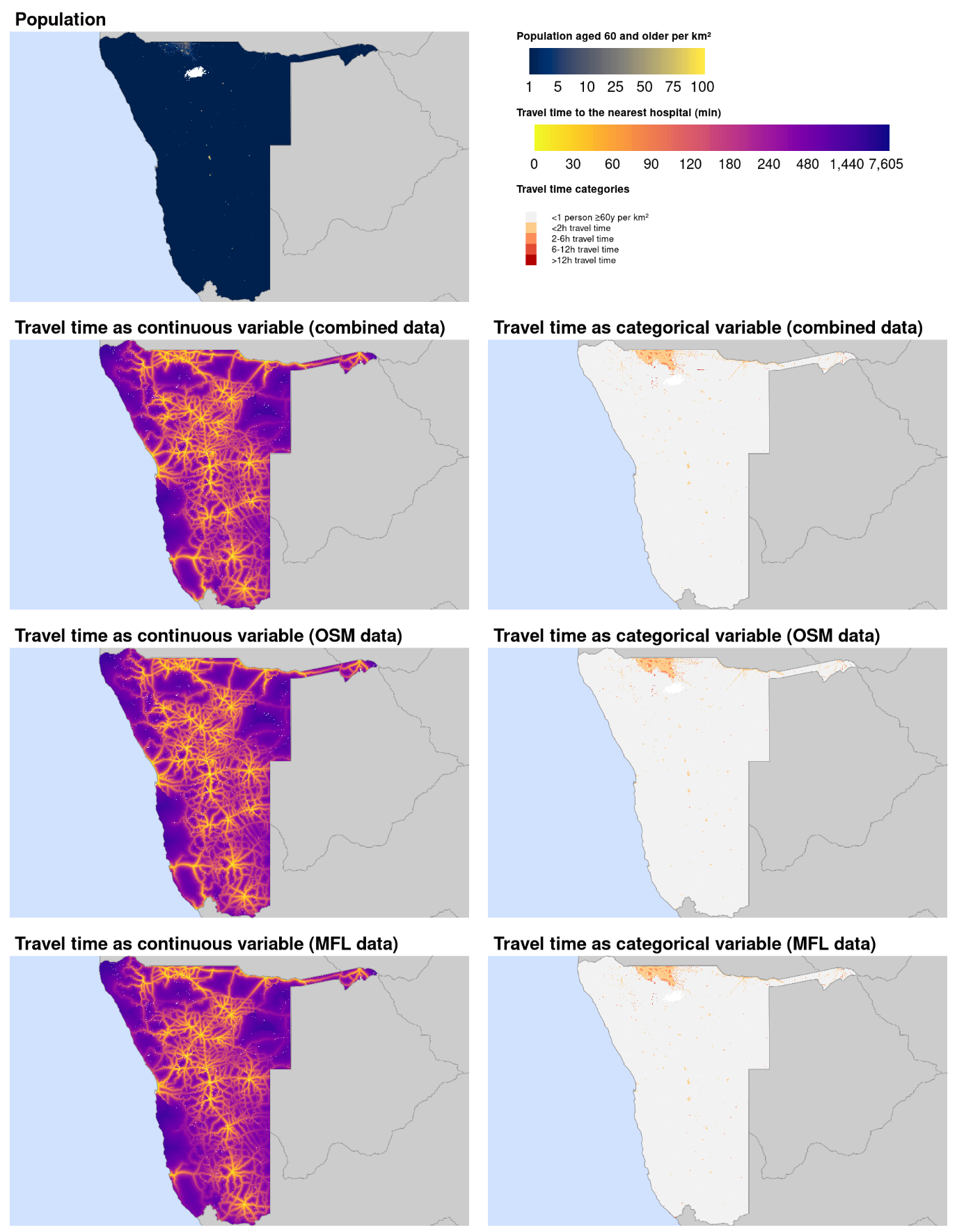


# **Figure S35. Niger map of travel time to the nearest hospital for adults aged ≥ 60 years**


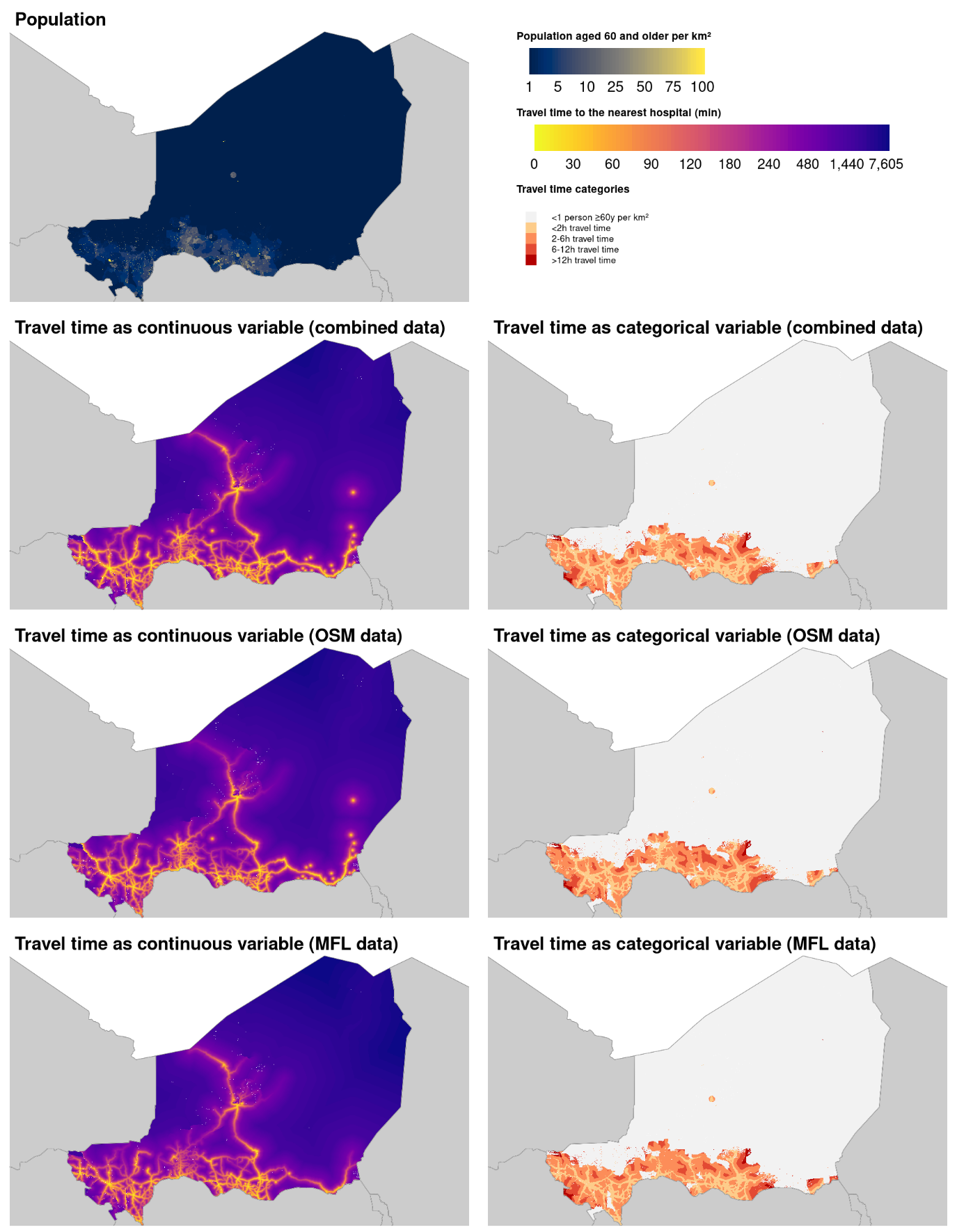


# **Figure S36. Nigeria map of travel time to the nearest hospital for adults aged ≥ 60 years**


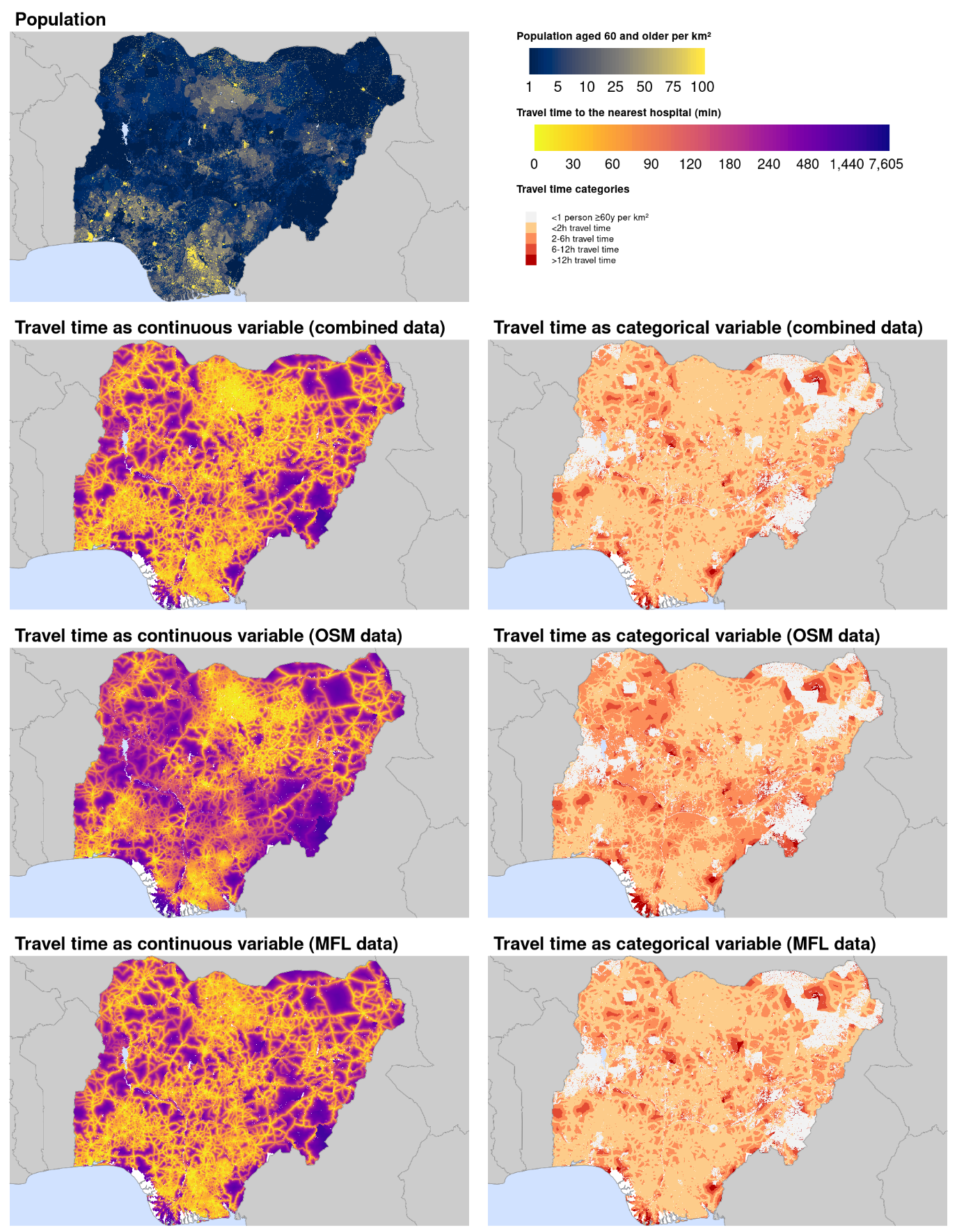


# **Figure S37. Republic of the Congo map of travel time to the nearest hospital for adults aged ≥ 60 years**


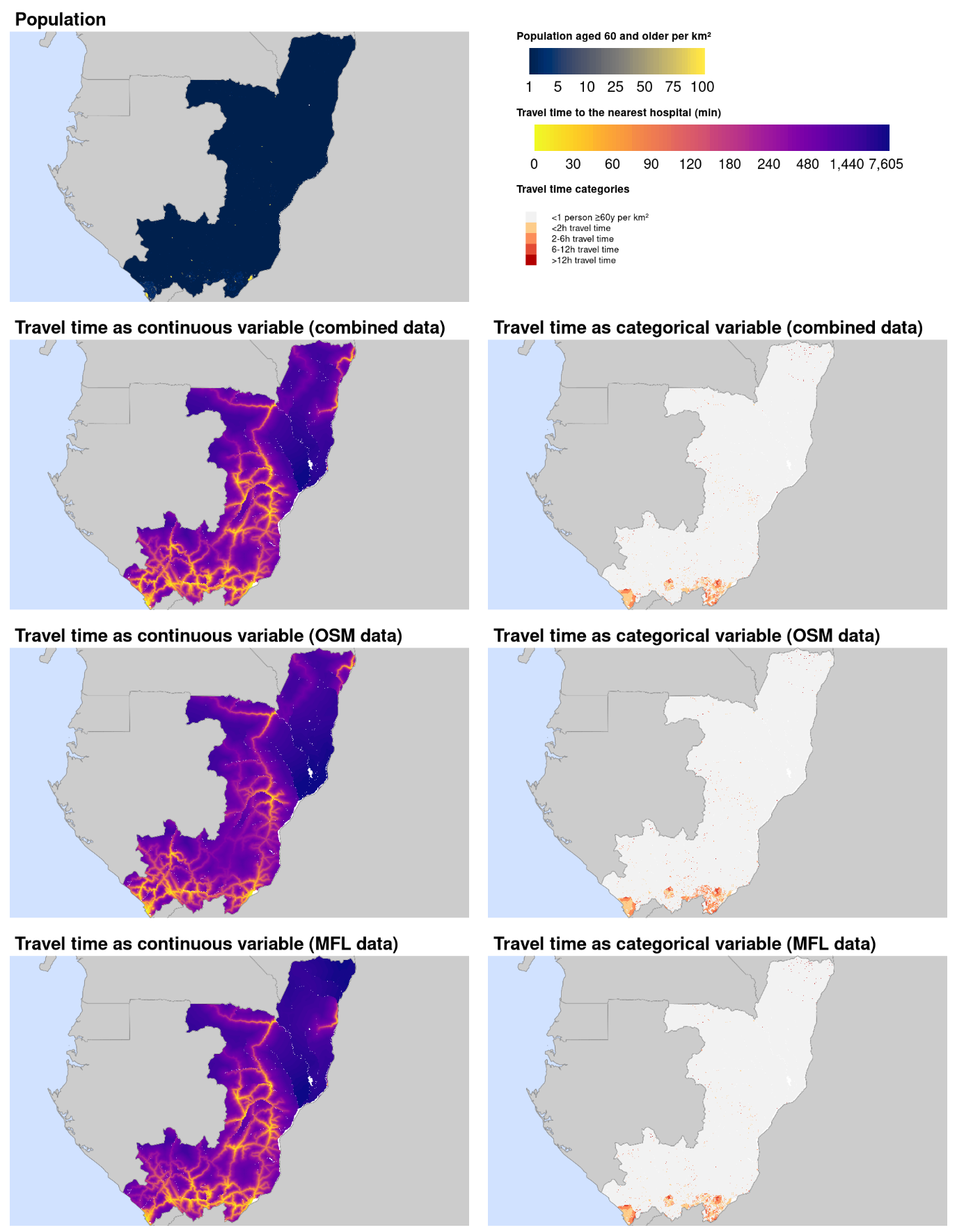


# **Figure S38. Rwanda map of travel time to the nearest hospital for adults aged ≥ 60 years**


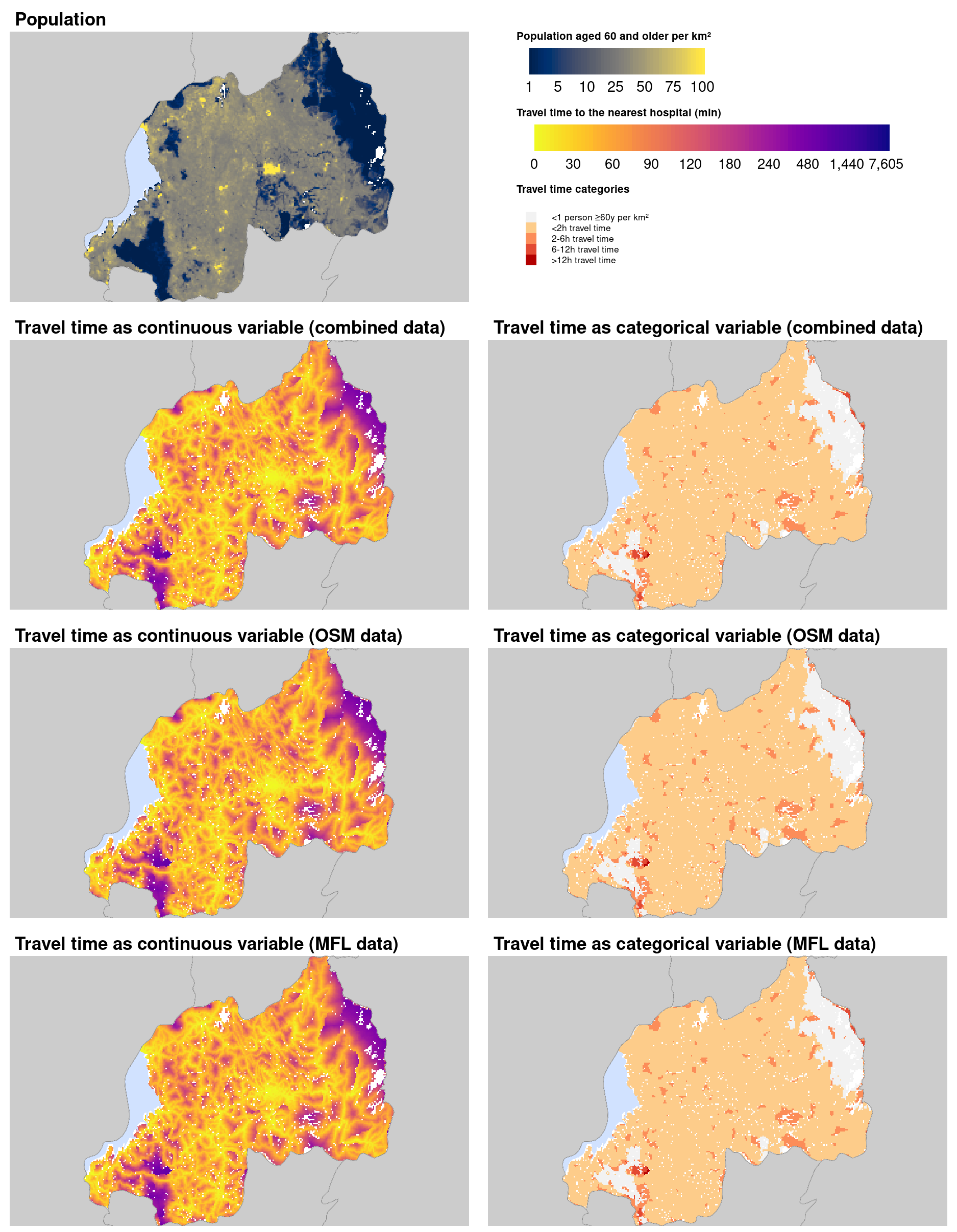


# **Figure S39. Senegal map of travel time to the nearest hospital for adults aged ≥ 60 years**


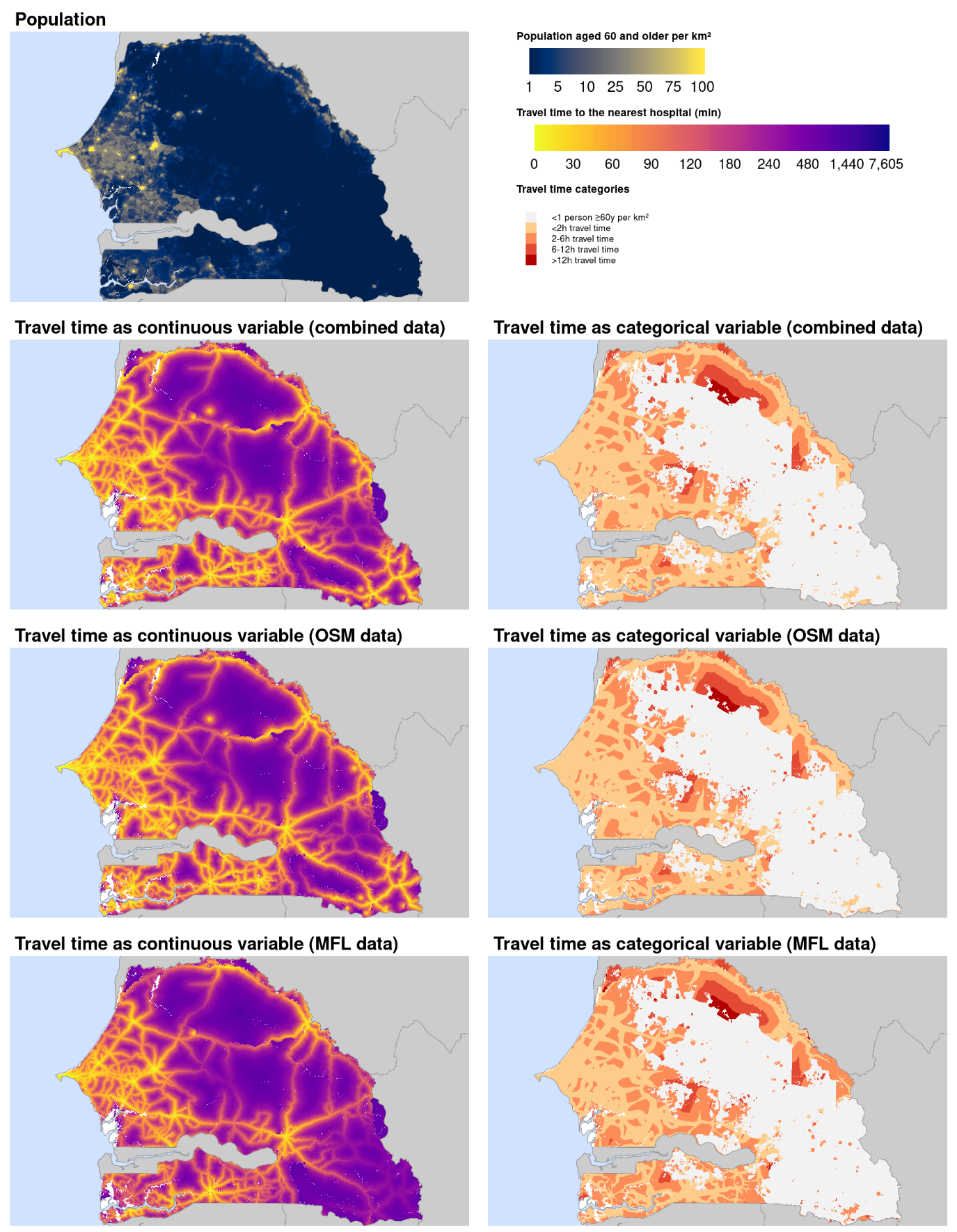


# **Figure S40. Sierra Leone map of travel time to the nearest hospital for adults aged ≥ 60 years**


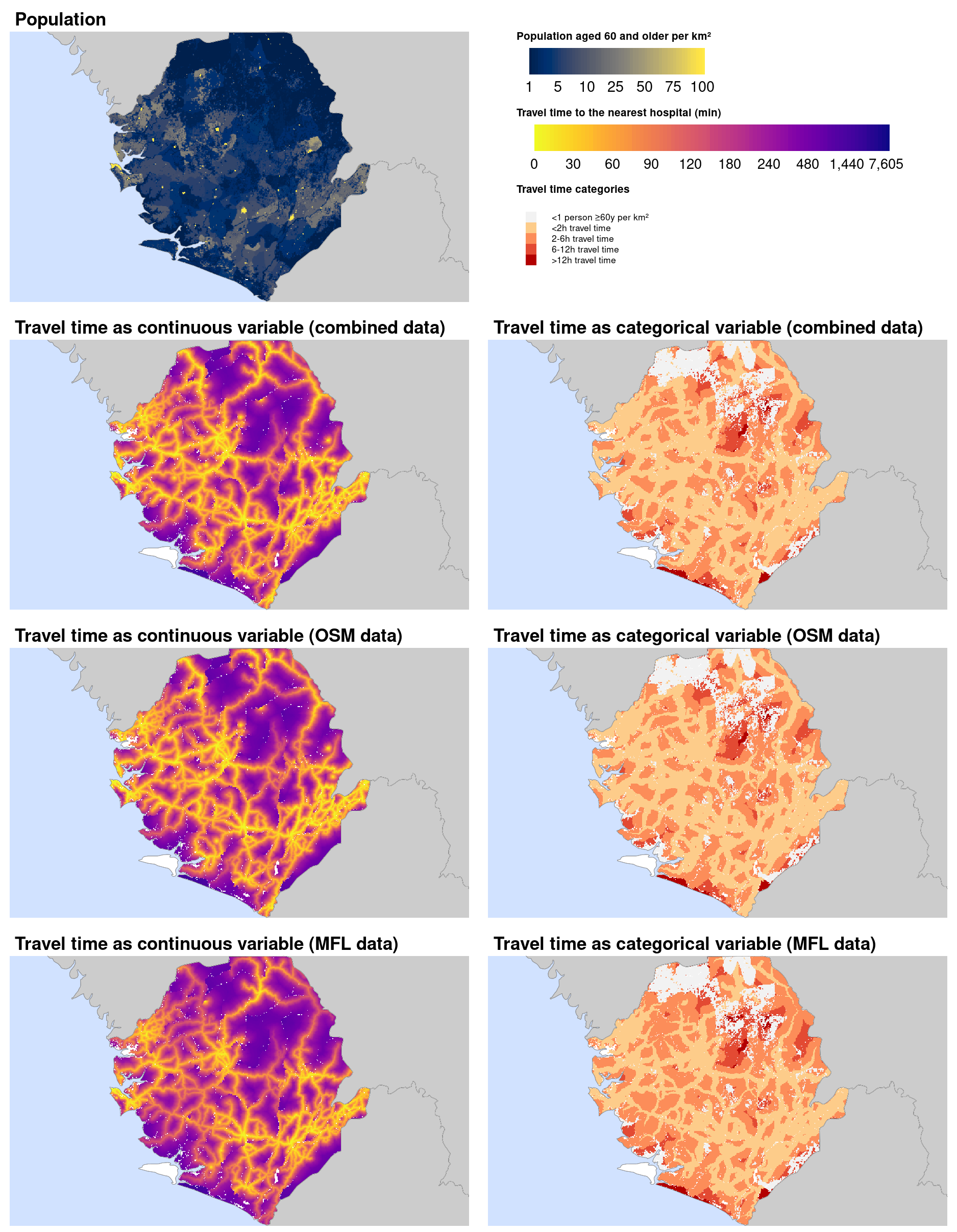


# **Figure S41. Somalia map of travel time to the nearest hospital for adults aged ≥ 60 years**


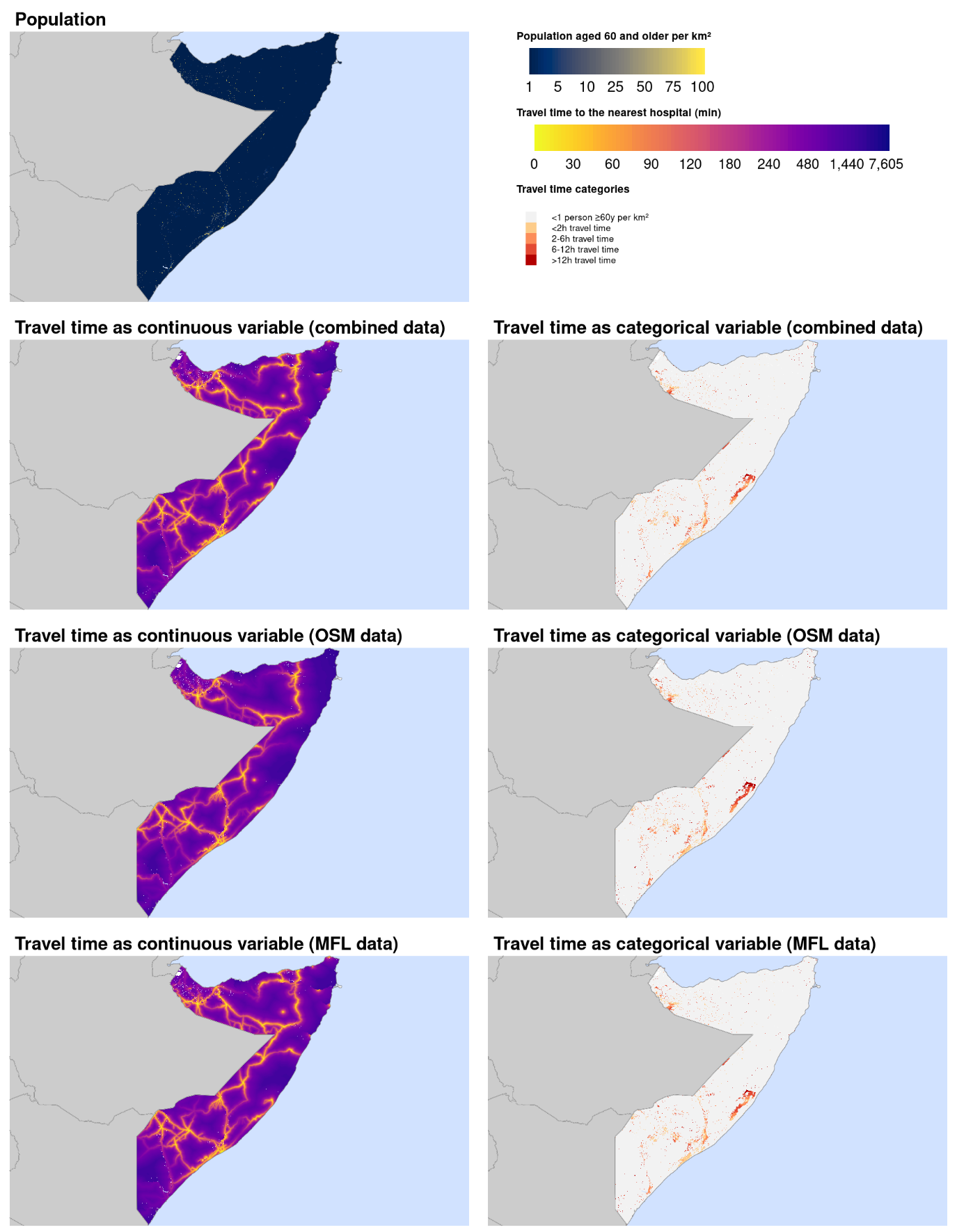


# **Figure S42. South Africa map of travel time to the nearest hospital for adults aged ≥ 60 years**


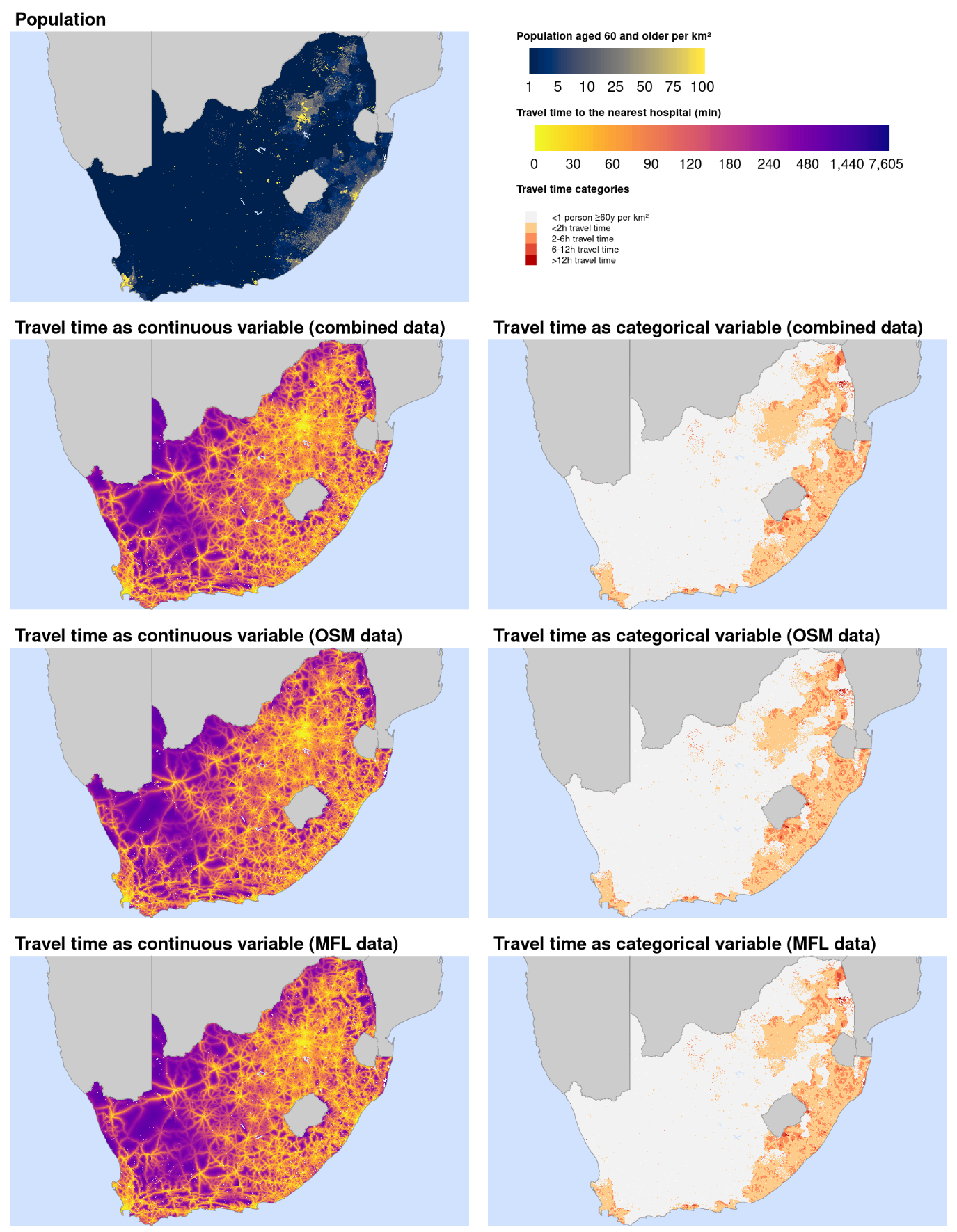


# **Figure S43. South Sudan map of travel time to the nearest hospital for adults aged ≥ 60 years**


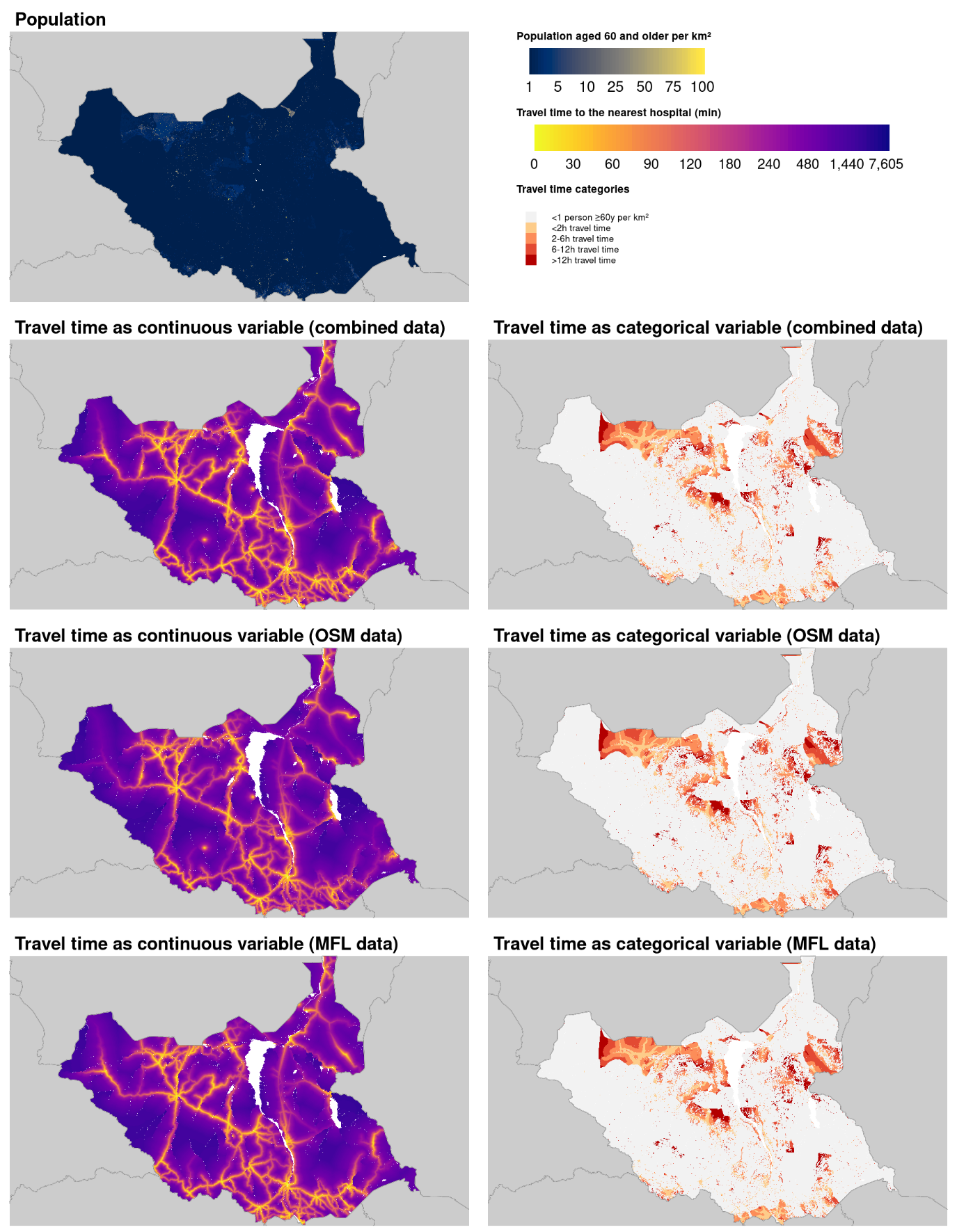


# **Figure S44. Sudan map of travel time to the nearest hospital for adults aged ≥ 60 years**


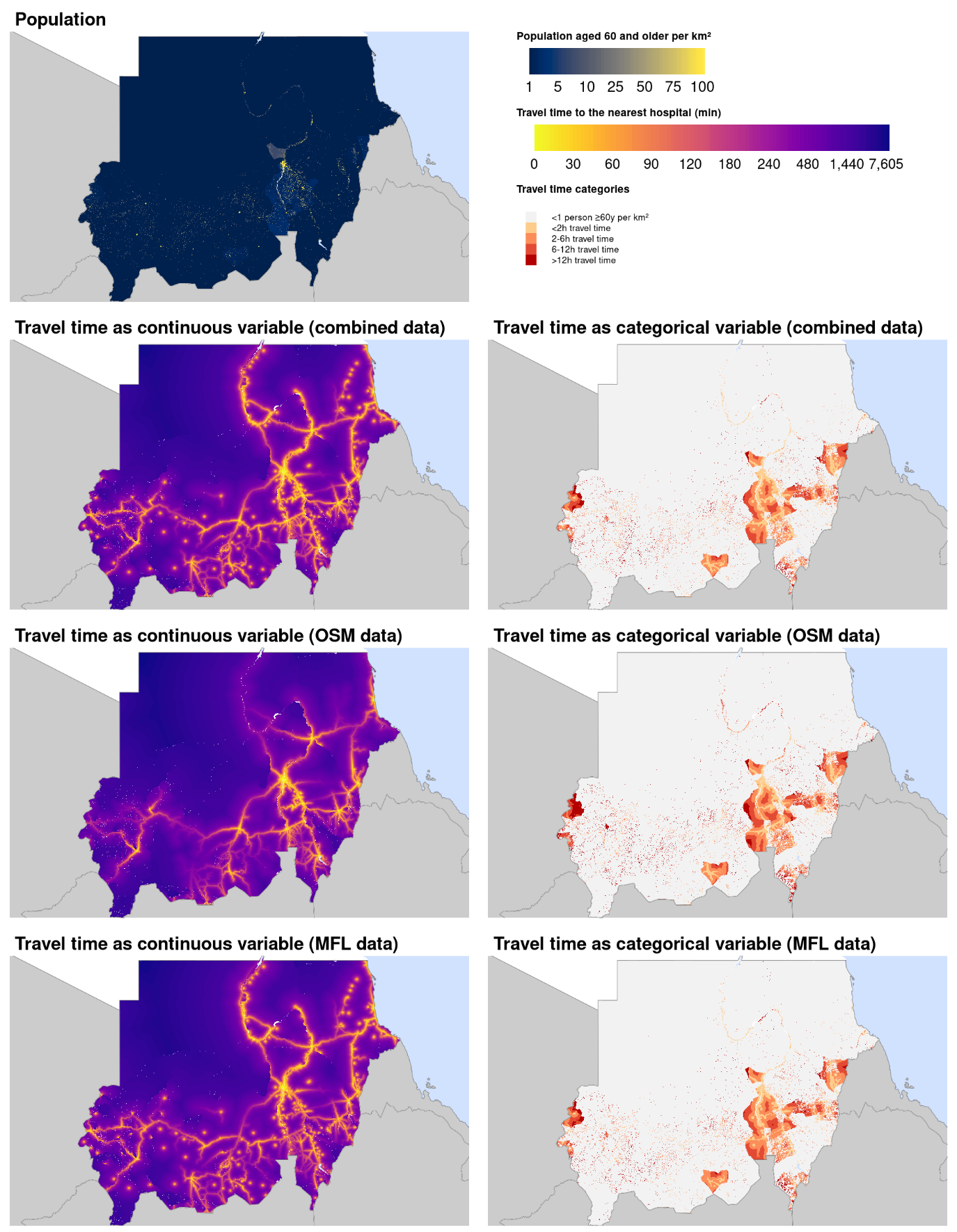


# **Figure S45. Tanzania map of travel time to the nearest hospital for adults aged ≥ 60 years**


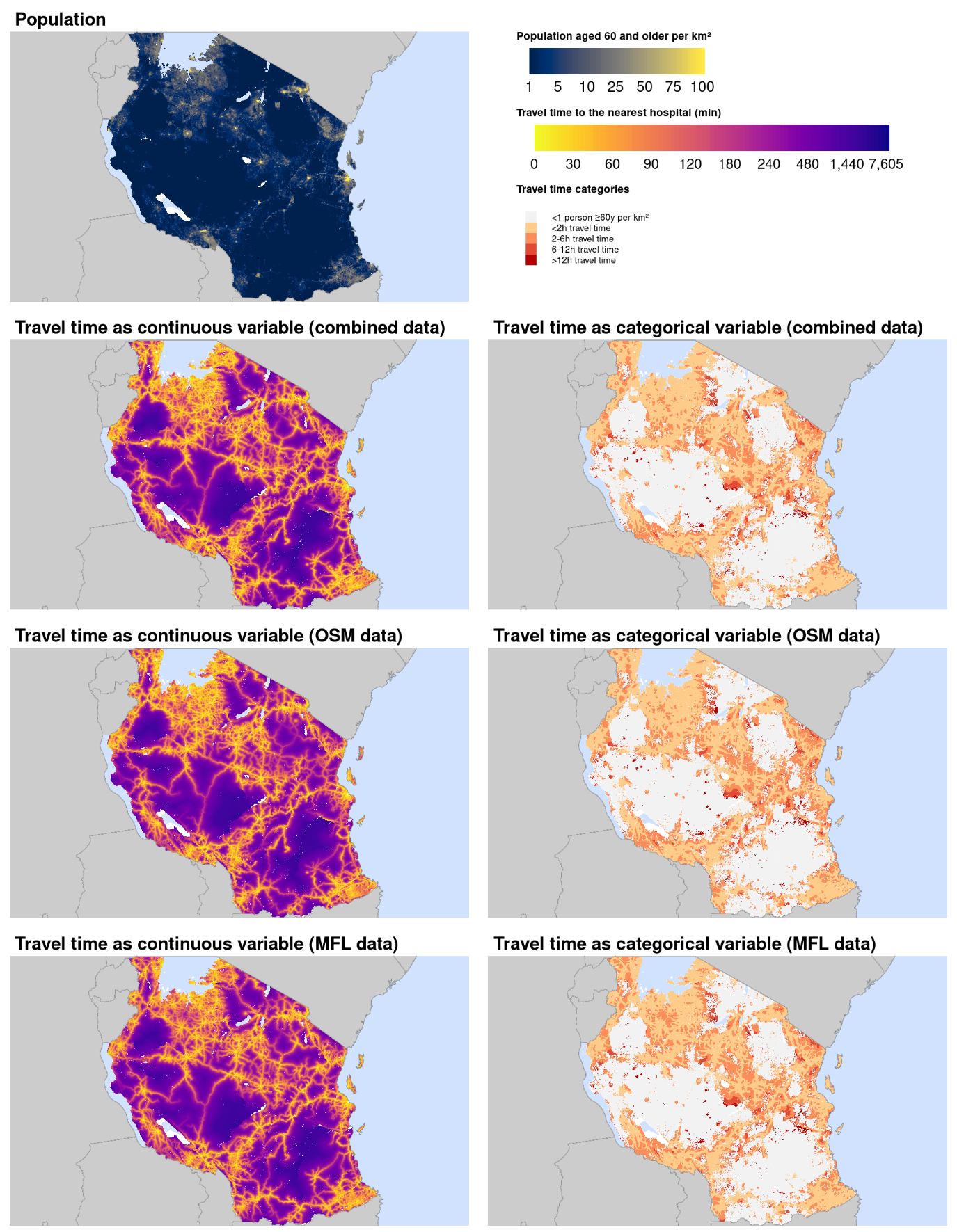


# **Figure S46. The Gambia map of travel time to the nearest hospital for adults aged ≥ 60 years**


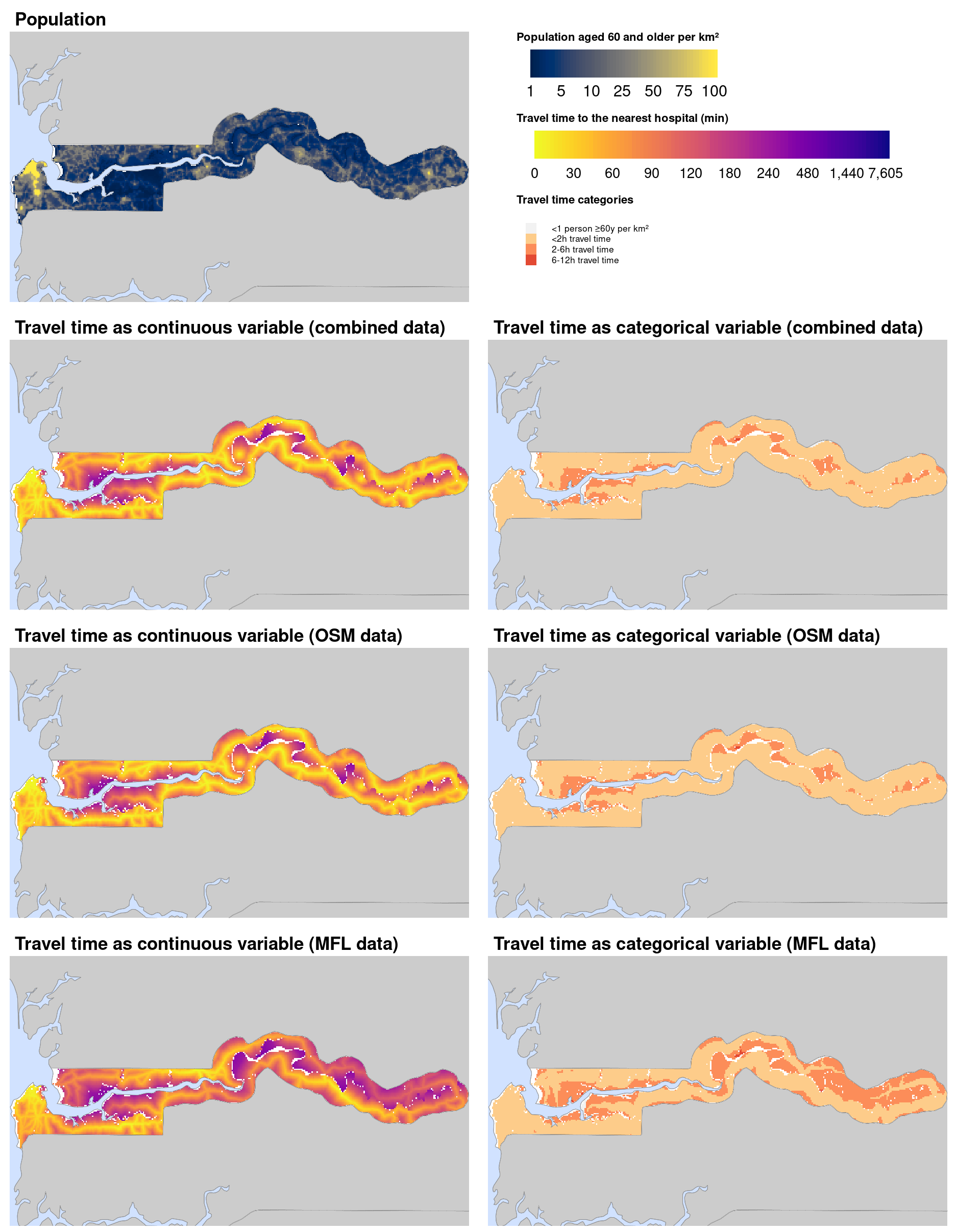


# **Figure S47. Togo map of travel time to the nearest hospital for adults aged ≥ 60 years**


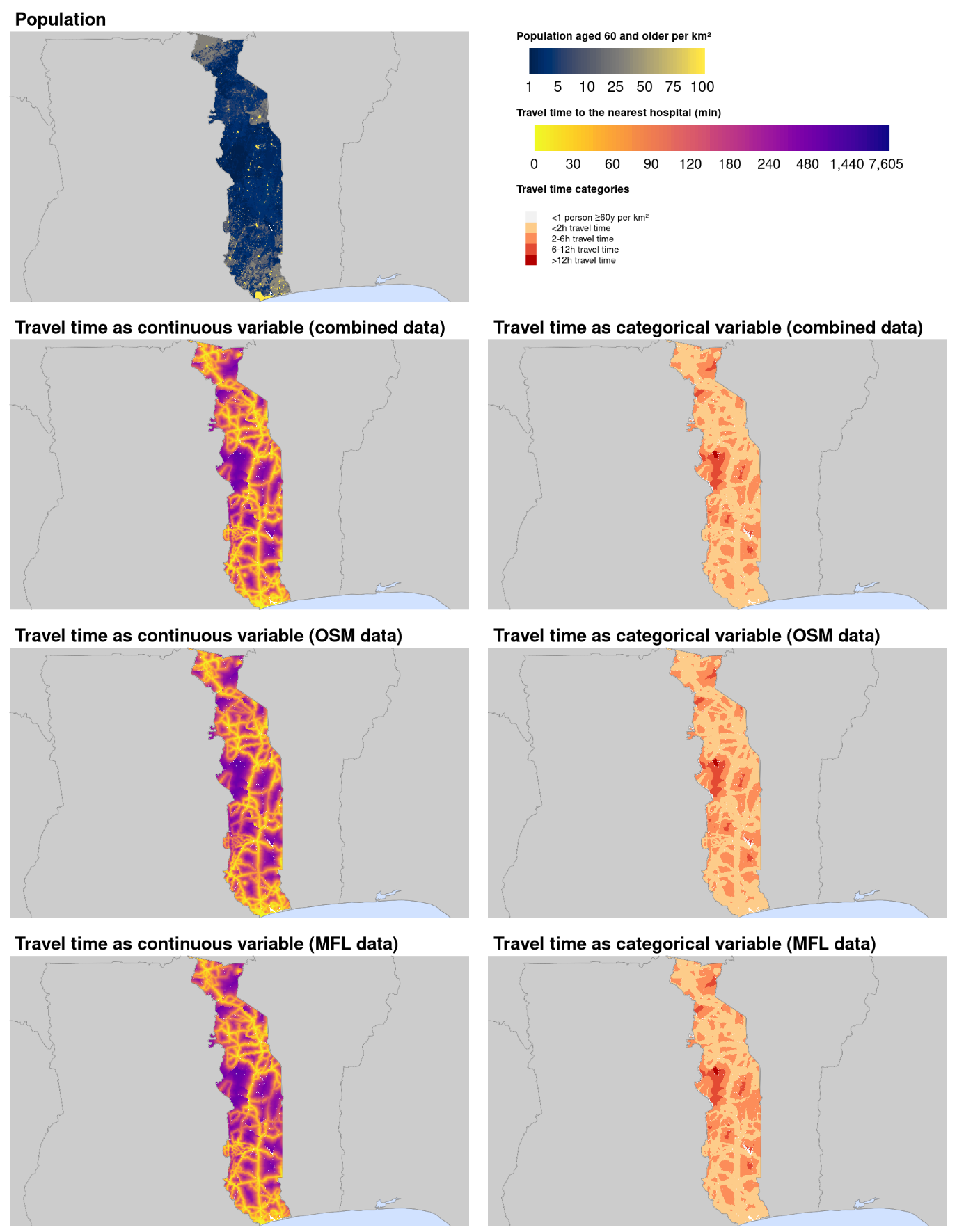


# **Figure S48. Uganda map of travel time to the nearest hospital for adults aged ≥ 60 years**


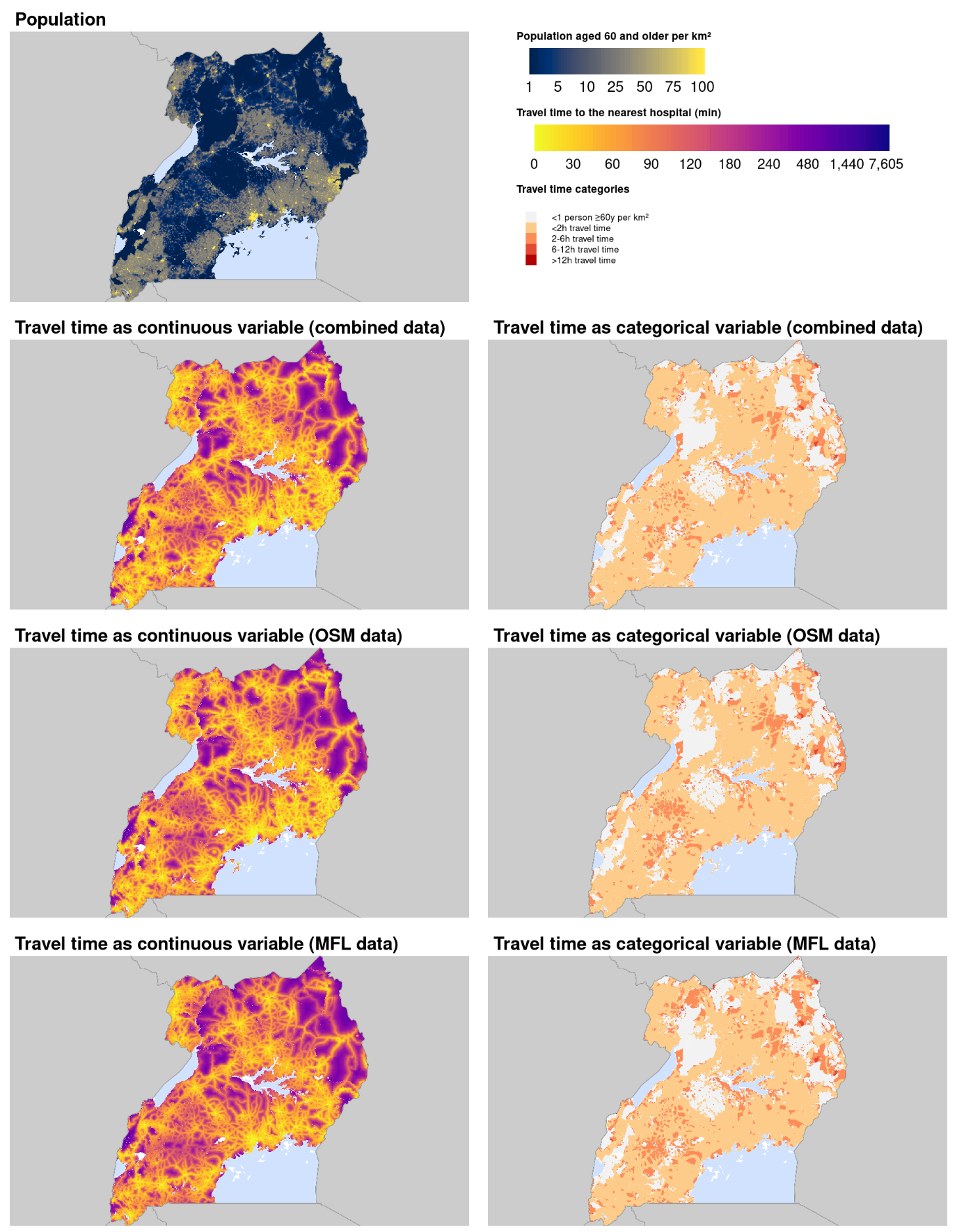


# **Figure S49. Zambia map of travel time to the nearest hospital for adults aged ≥ 60 years**


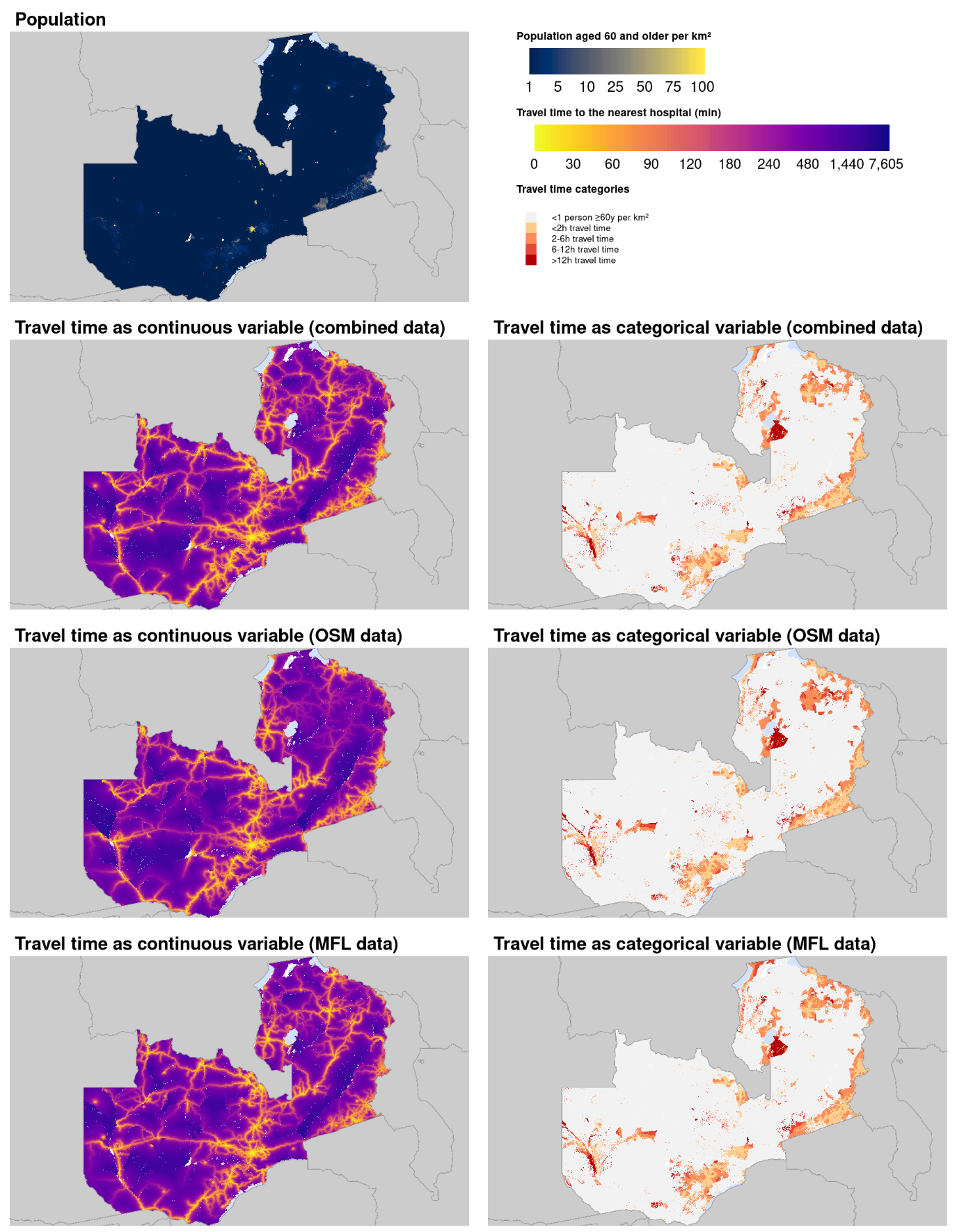


# **Figure S50. Zimbabwe map of travel time to the nearest hospital for adults aged ≥ 60 years**


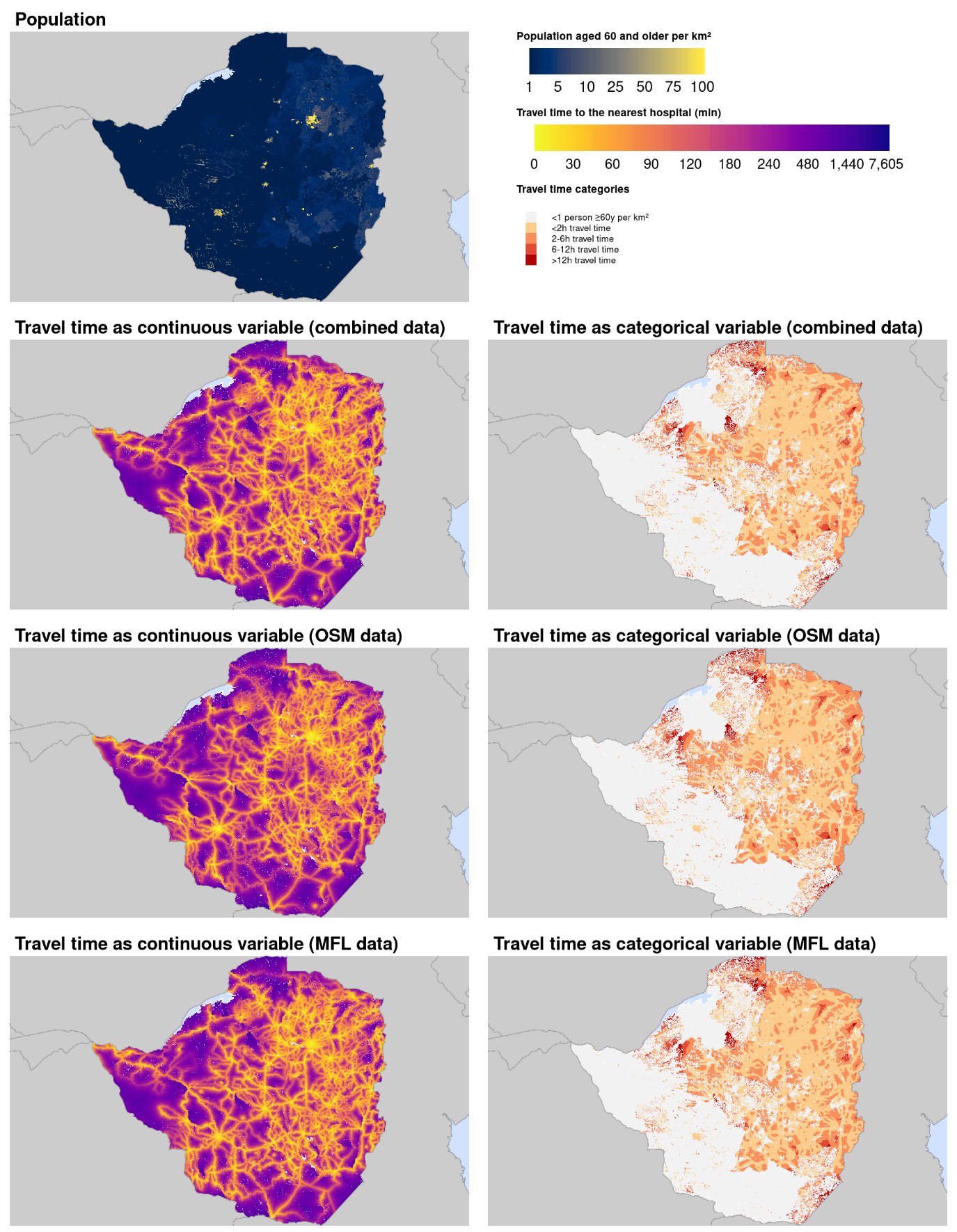


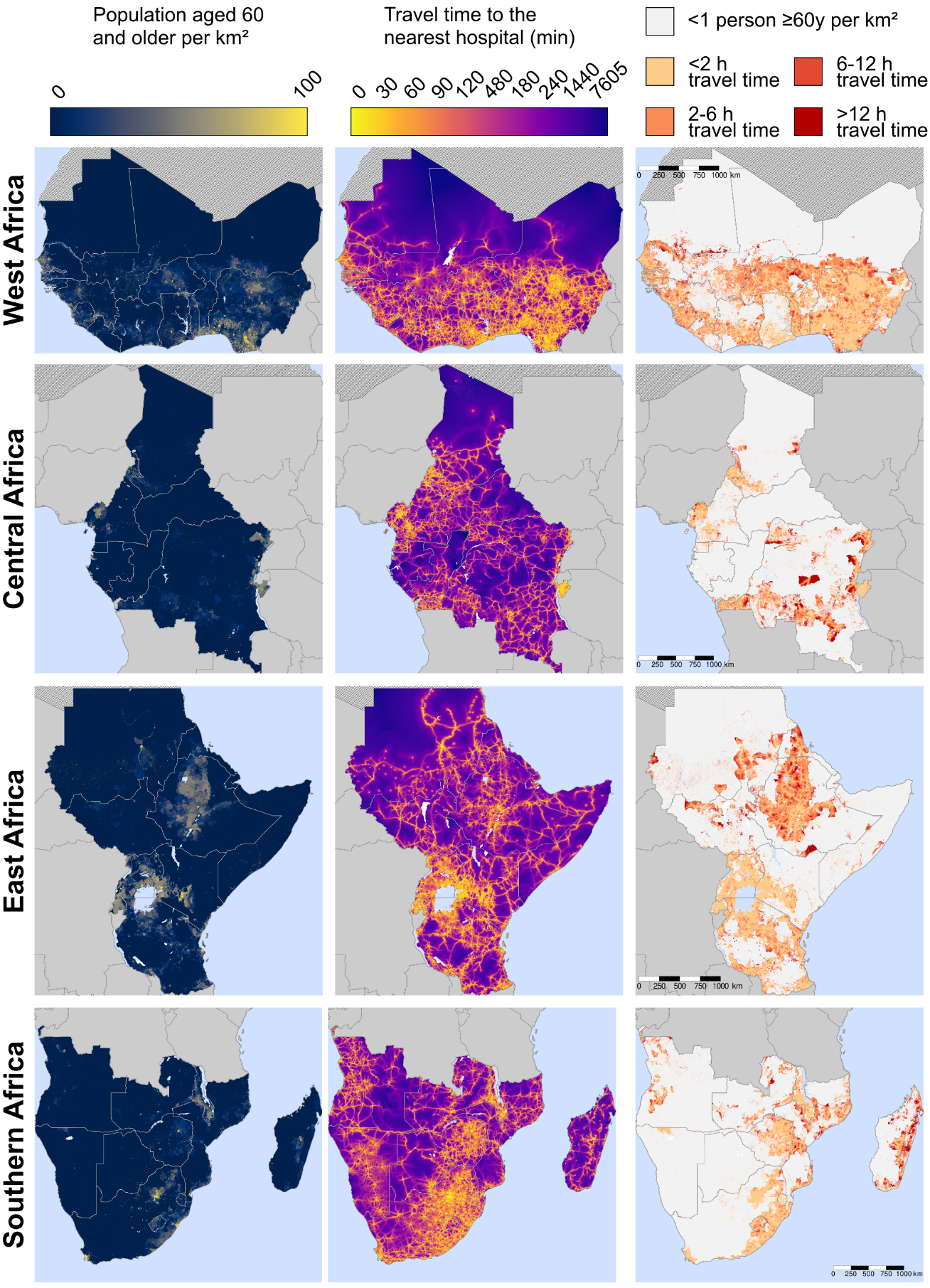


# **Figure S51. Maps of travel time to the nearest hospital for adults ≥ 60 years, by region based on the MFL dataset**


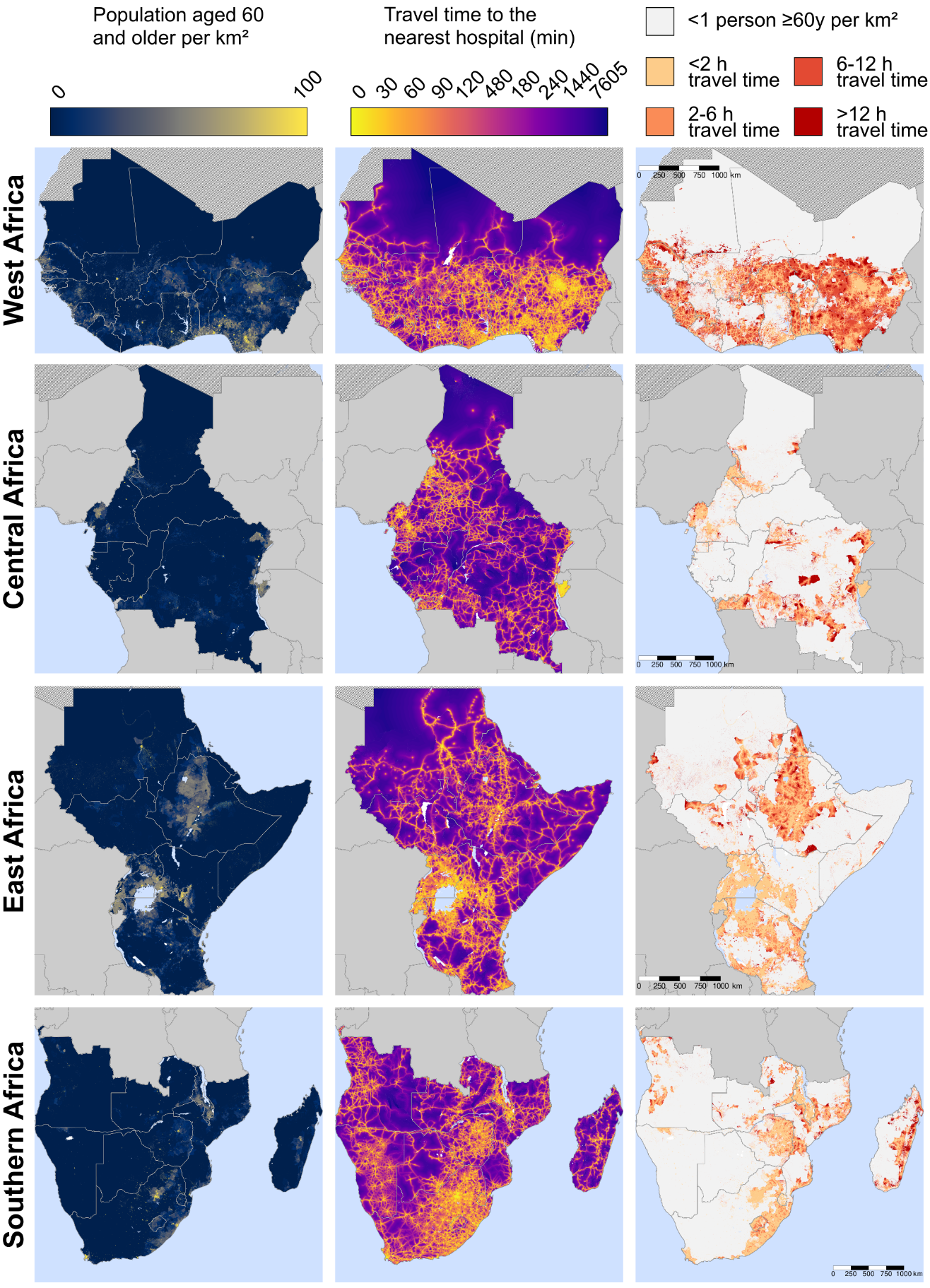


# **Figure S52. Maps of travel time to the nearest hospital for adults ≥ 60 years, by region based on the OSM dataset**

# **Figure S53. Angola map of travel time to the nearest healthcare facility for adults aged ≥ 60 years**


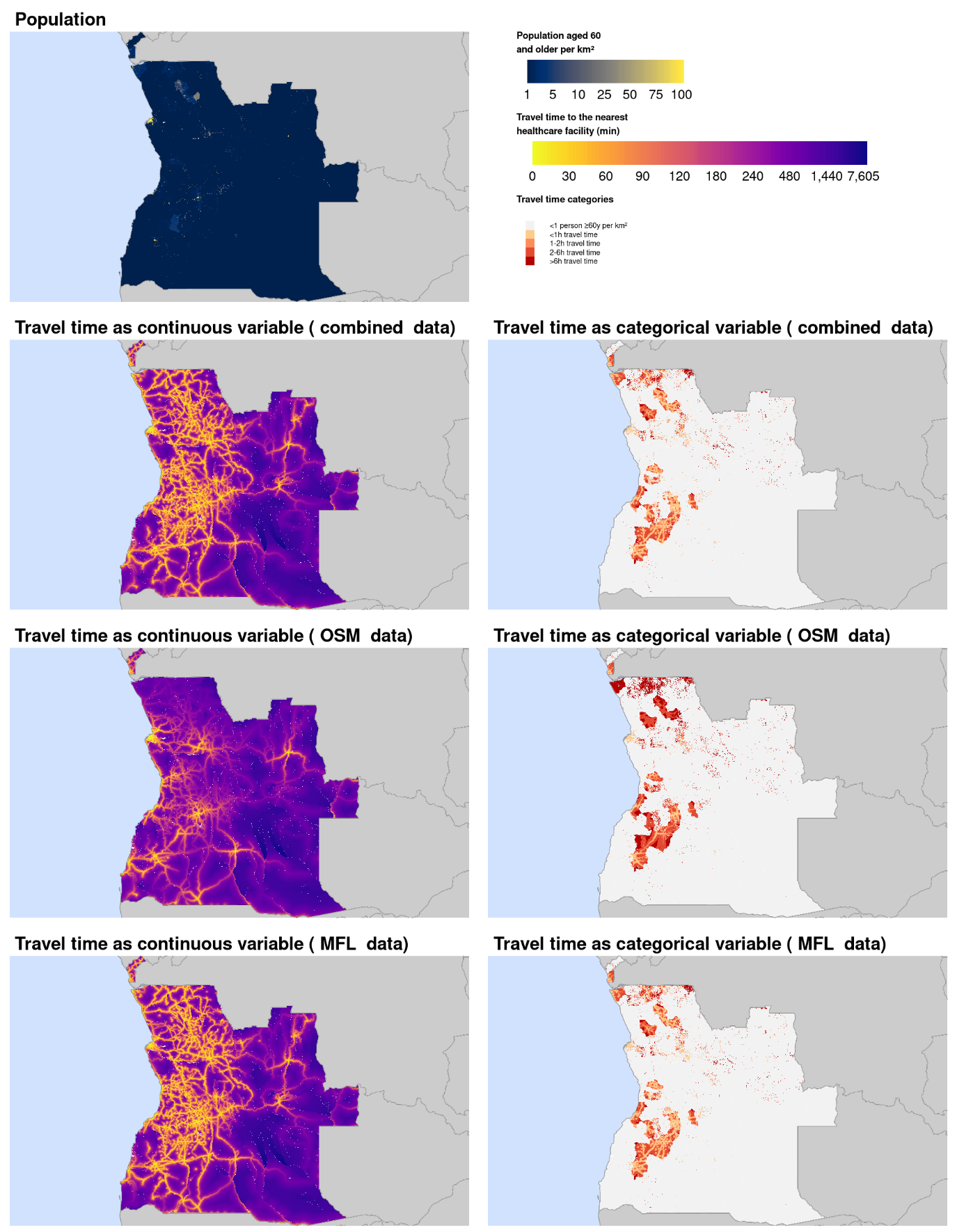


# **Figure S54. Benin map of travel time to the nearest healthcare facility for adults aged ≥ 60 years**


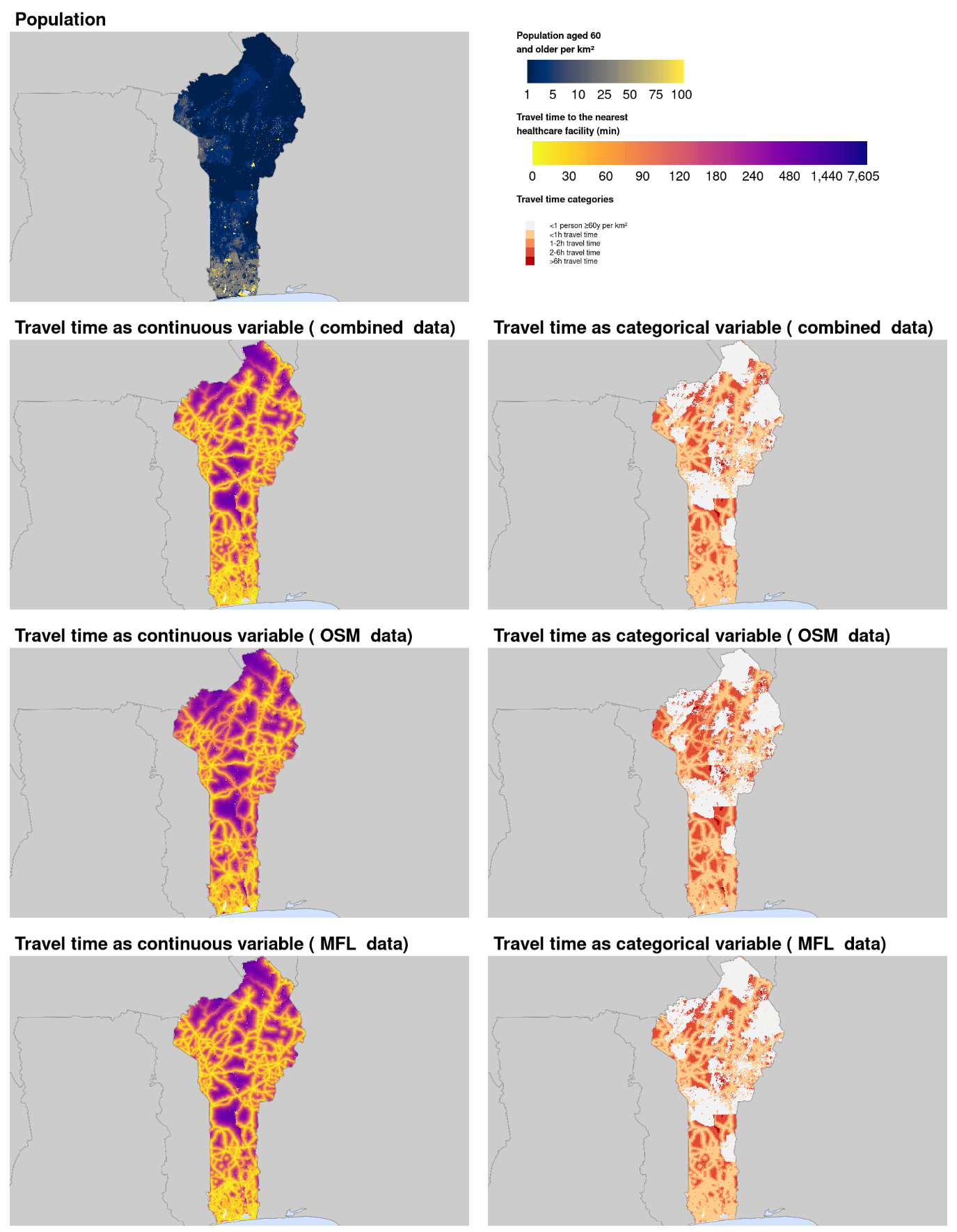


# **Figure S55. Botswana map of travel time to the nearest healthcare facility for adults aged ≥ 60 years**


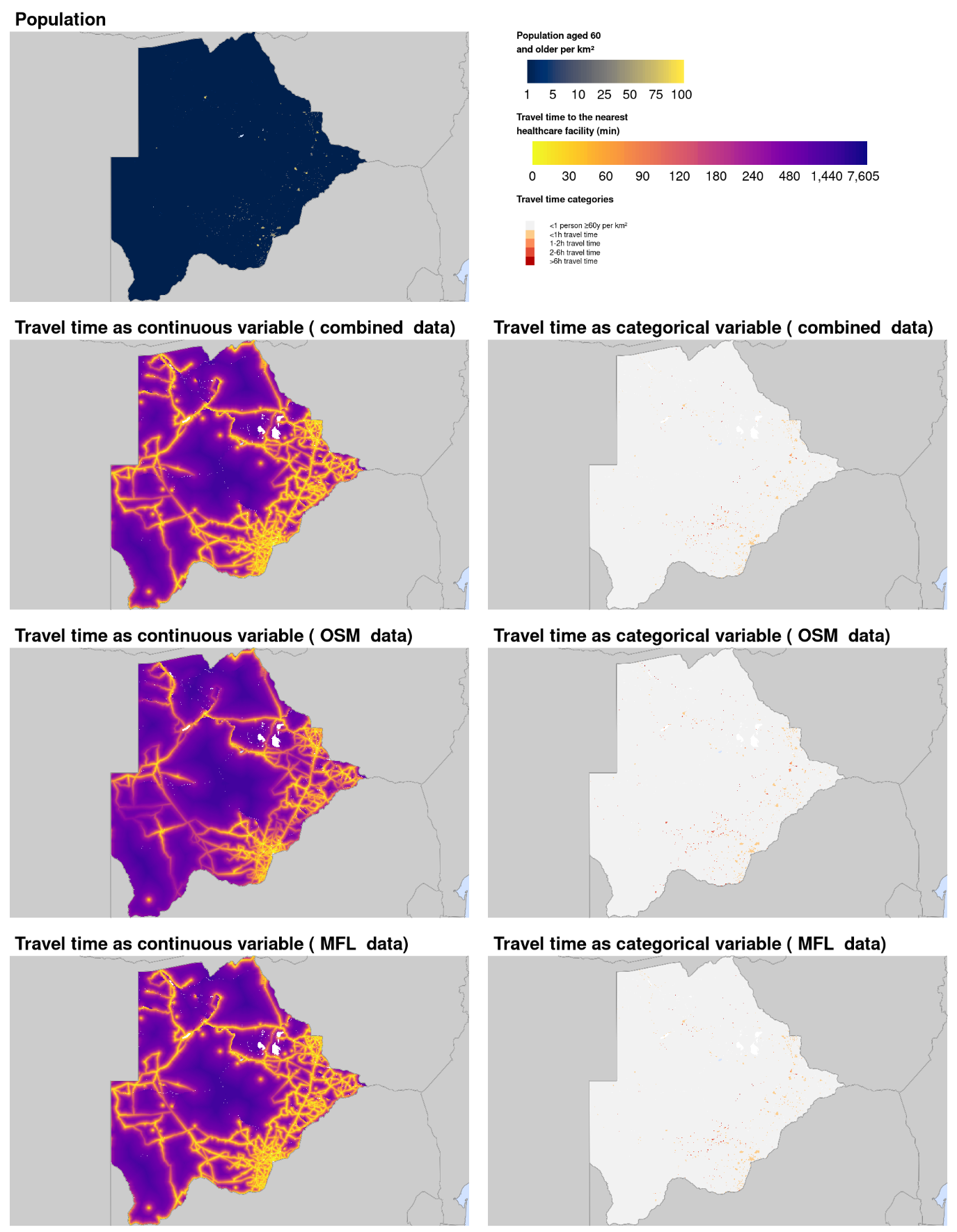


# **Figure S56. Burkina Faso map of travel time to the nearest healthcare facility for adults aged ≥ 60 years**


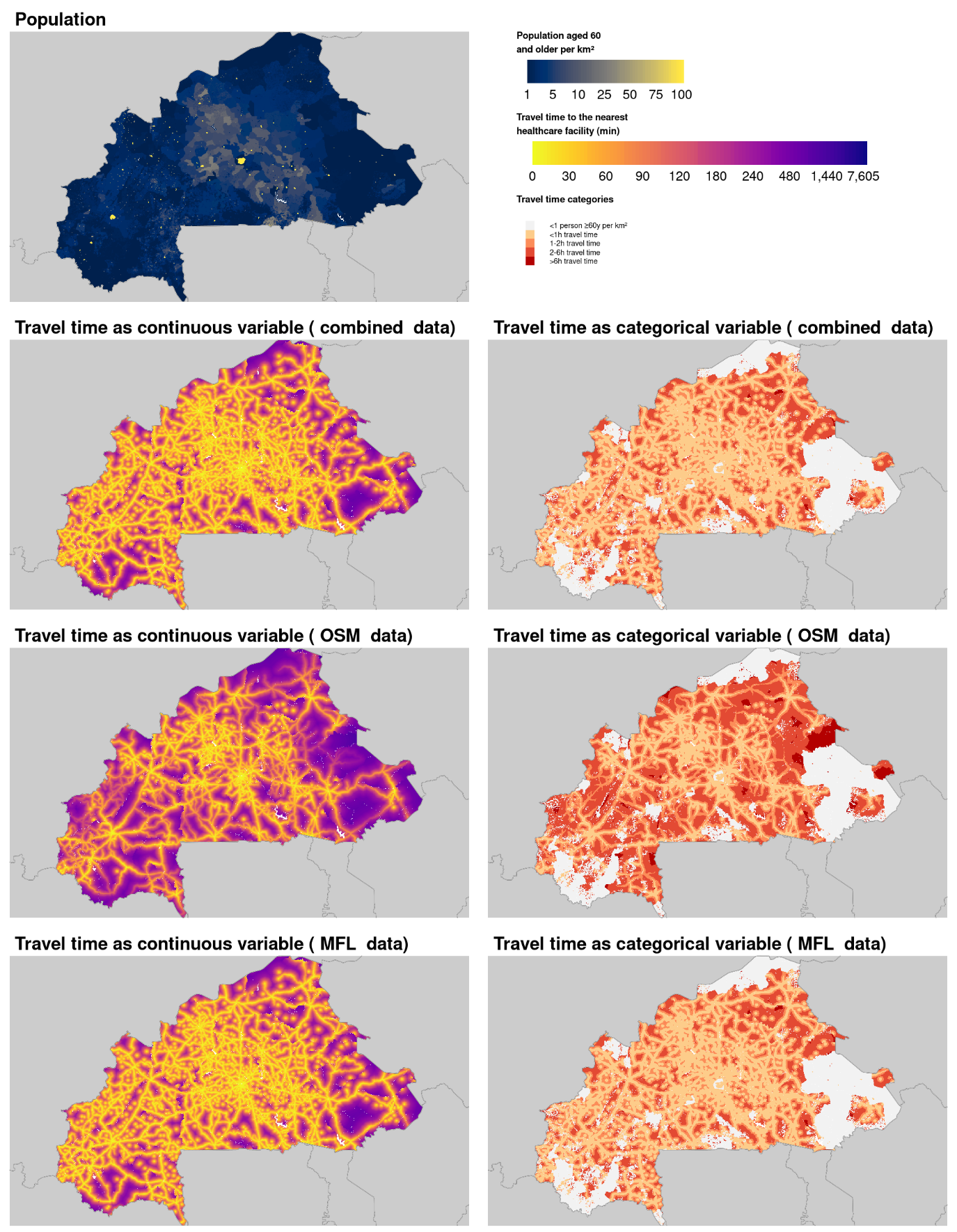


# **Figure S57. Burundi map of travel time to the nearest healthcare facility for adults aged ≥ 60 years**


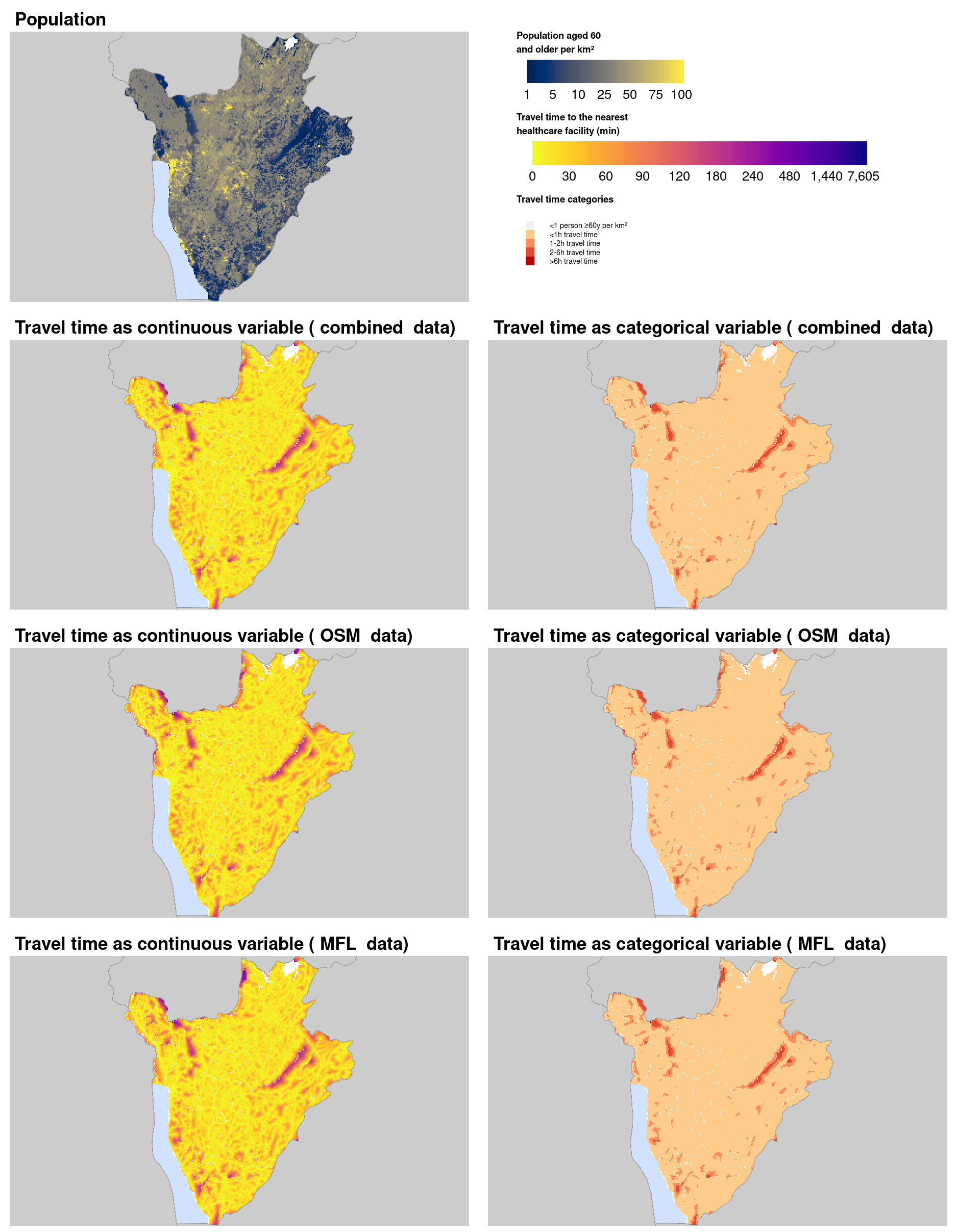


# **Figure S58. Cameroon map of travel time to the nearest healthcare facility for adults aged ≥ 60 years**


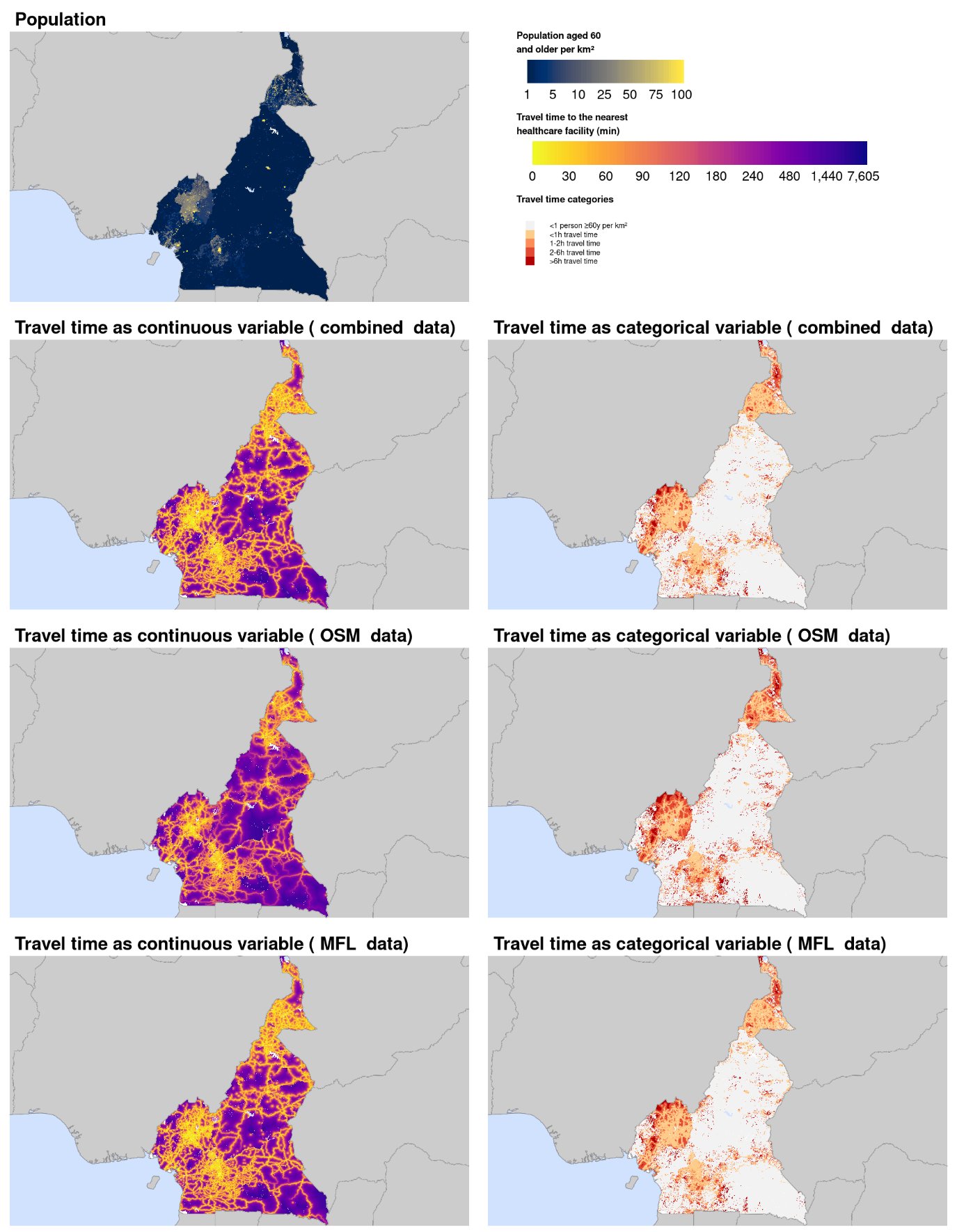


# **Figure S59. Central African Republic map of travel time to the nearest healthcare facility for adults aged ≥ 60 years**


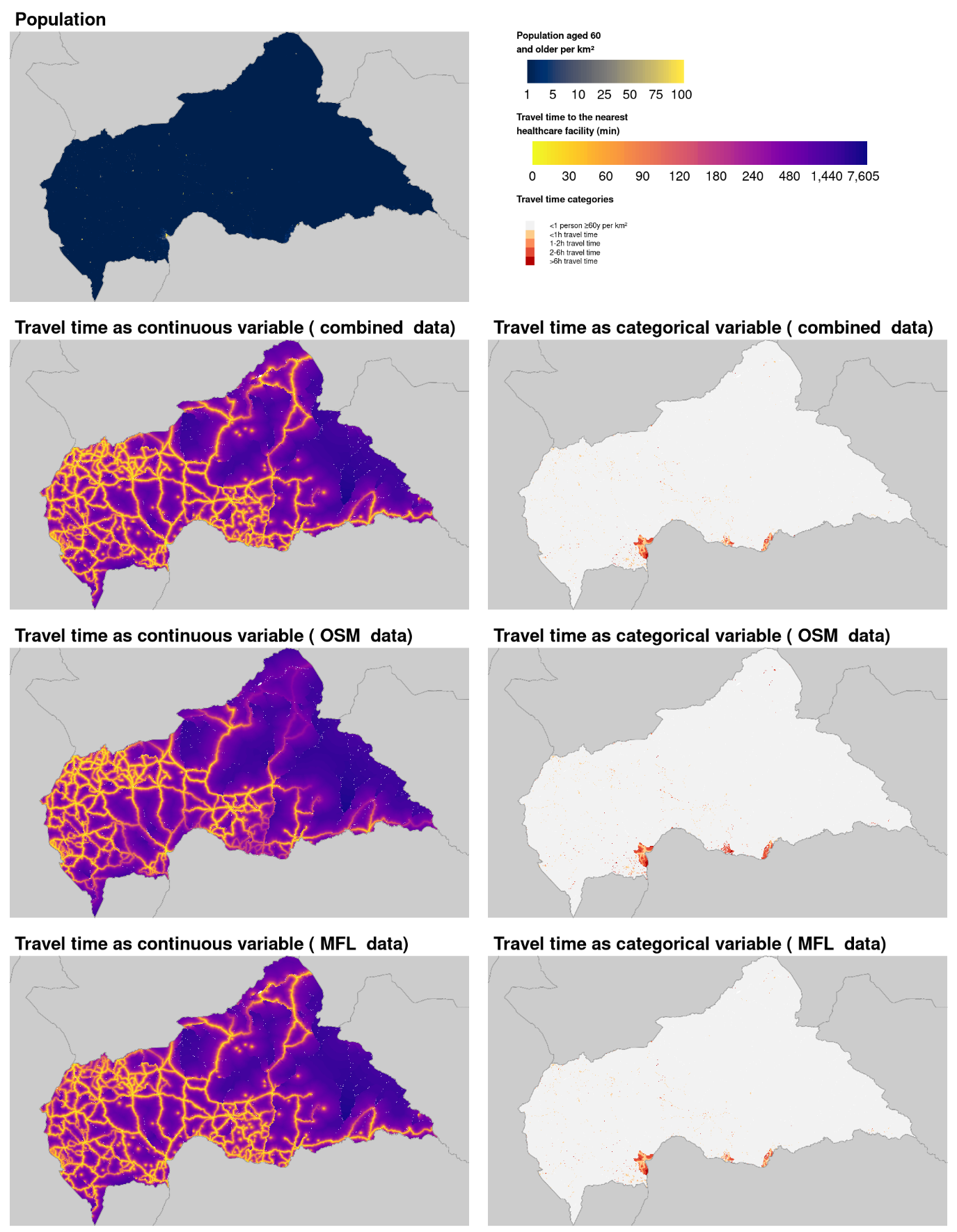


# **Figure S60. Chad map of travel time to the nearest healthcare facility for adults aged ≥ 60 years**


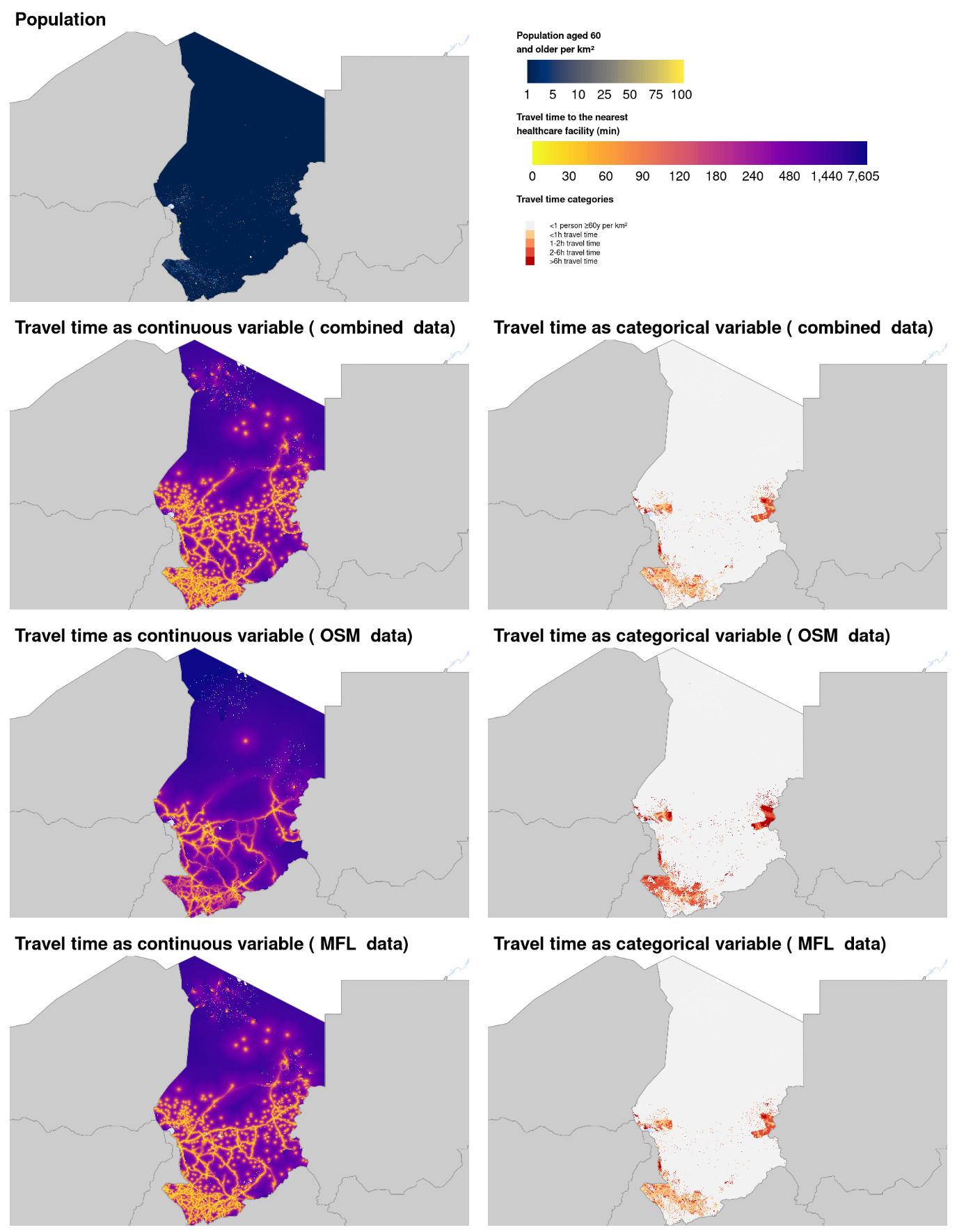


# **Figure S61. Djibouti map of travel time to the nearest healthcare facility for adults aged ≥ 60 years**


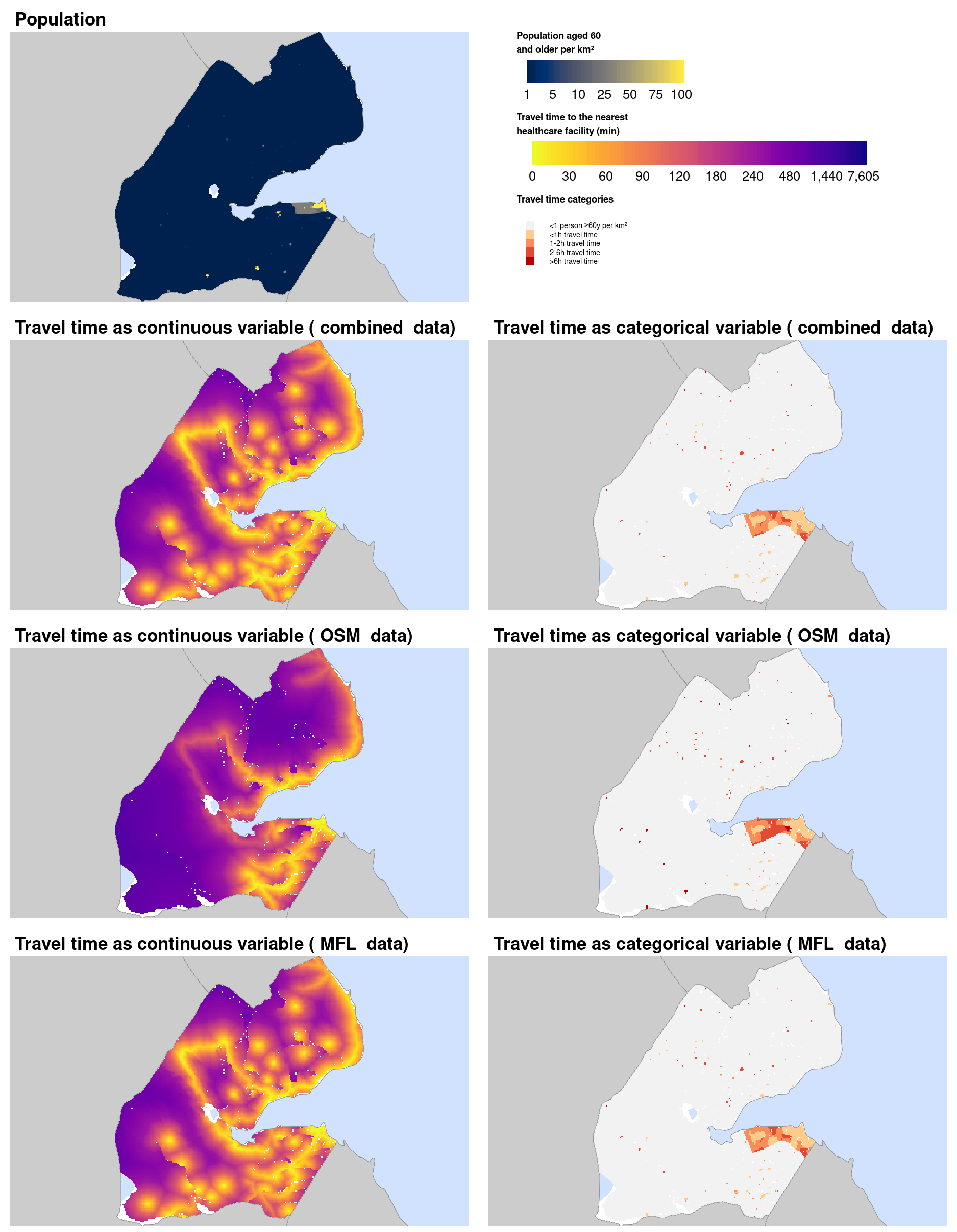


# **Figure S62. DRC map of travel time to the nearest healthcare facility for adults aged ≥ 60 years**


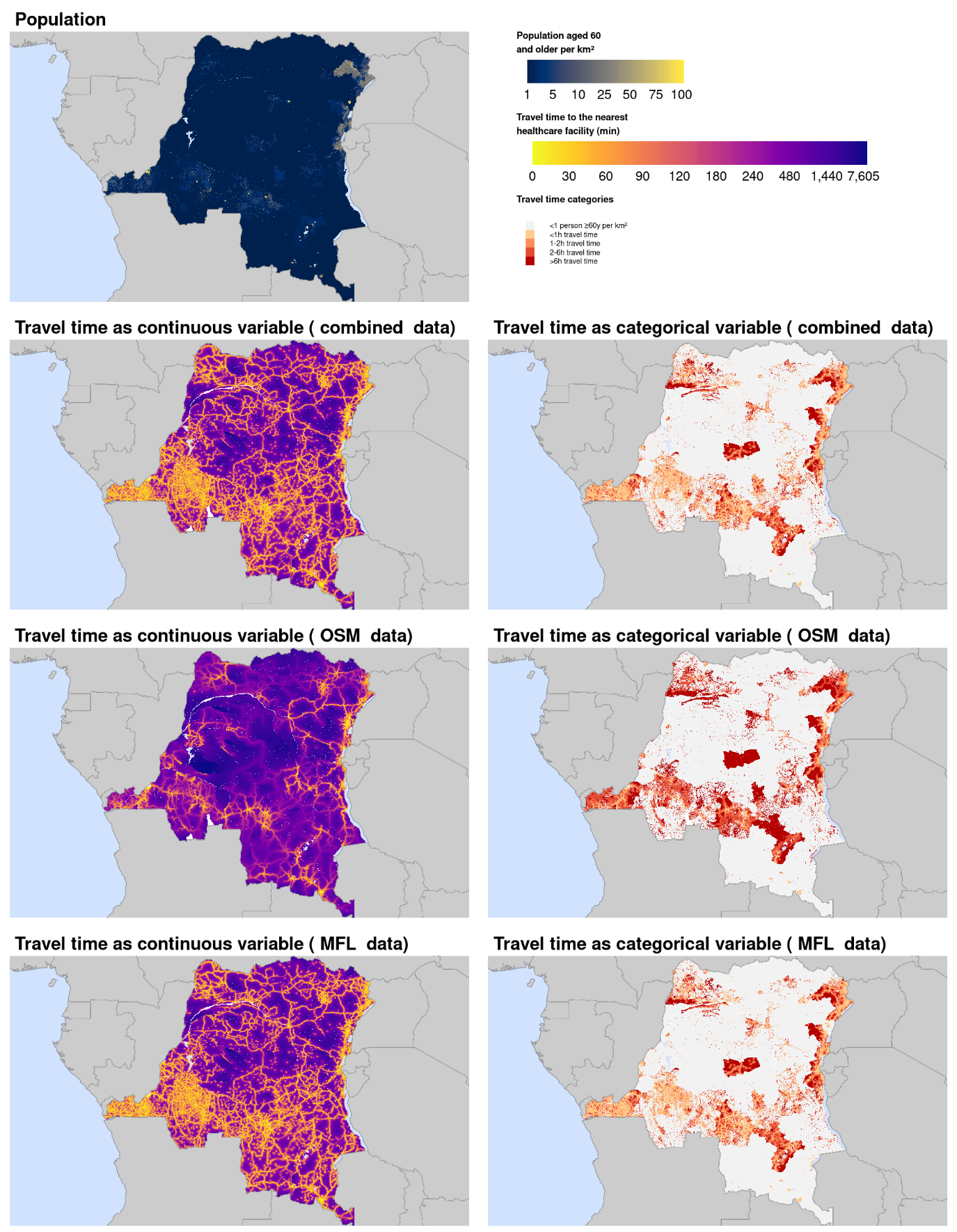


# **Figure S63. Equatorial Guinea map of travel time to the nearest healthcare facility for adults aged ≥ 60 years**


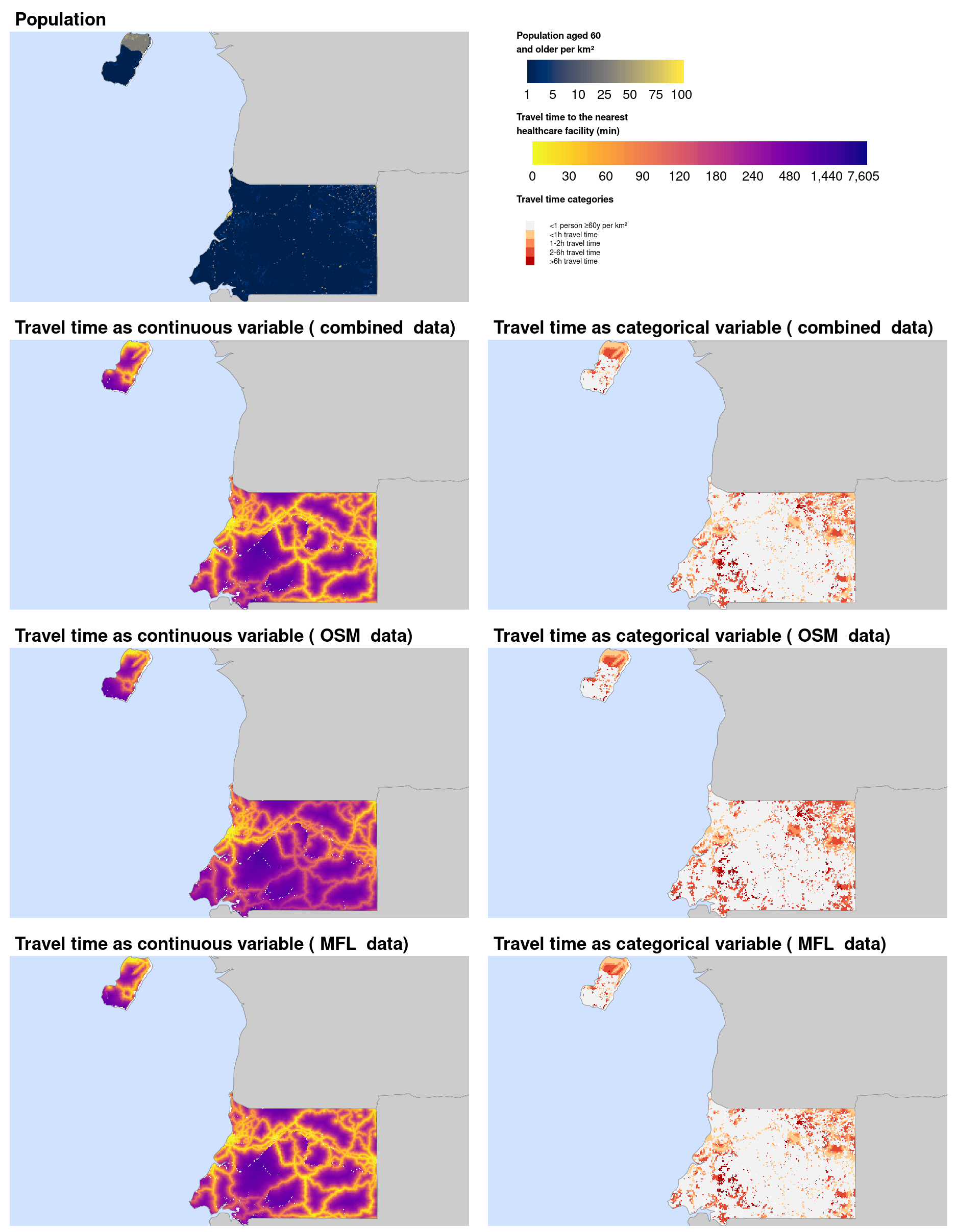


# **Figure S64. Eritrea map of travel time to the nearest healthcare facility for adults aged ≥ 60 years**


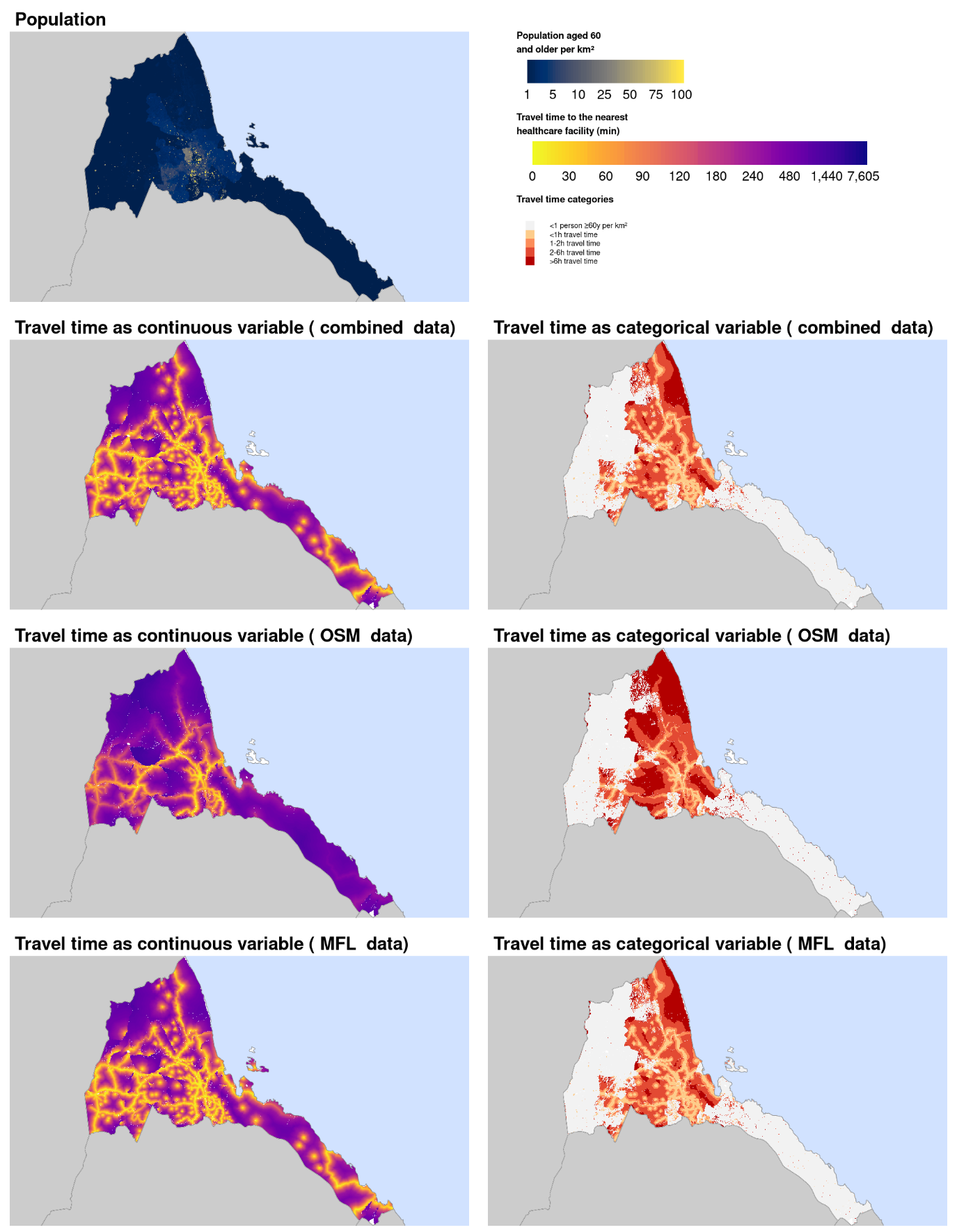


# **Figure S65. eSwatini map of travel time to the nearest healthcare facility for adults aged ≥ 60 years**


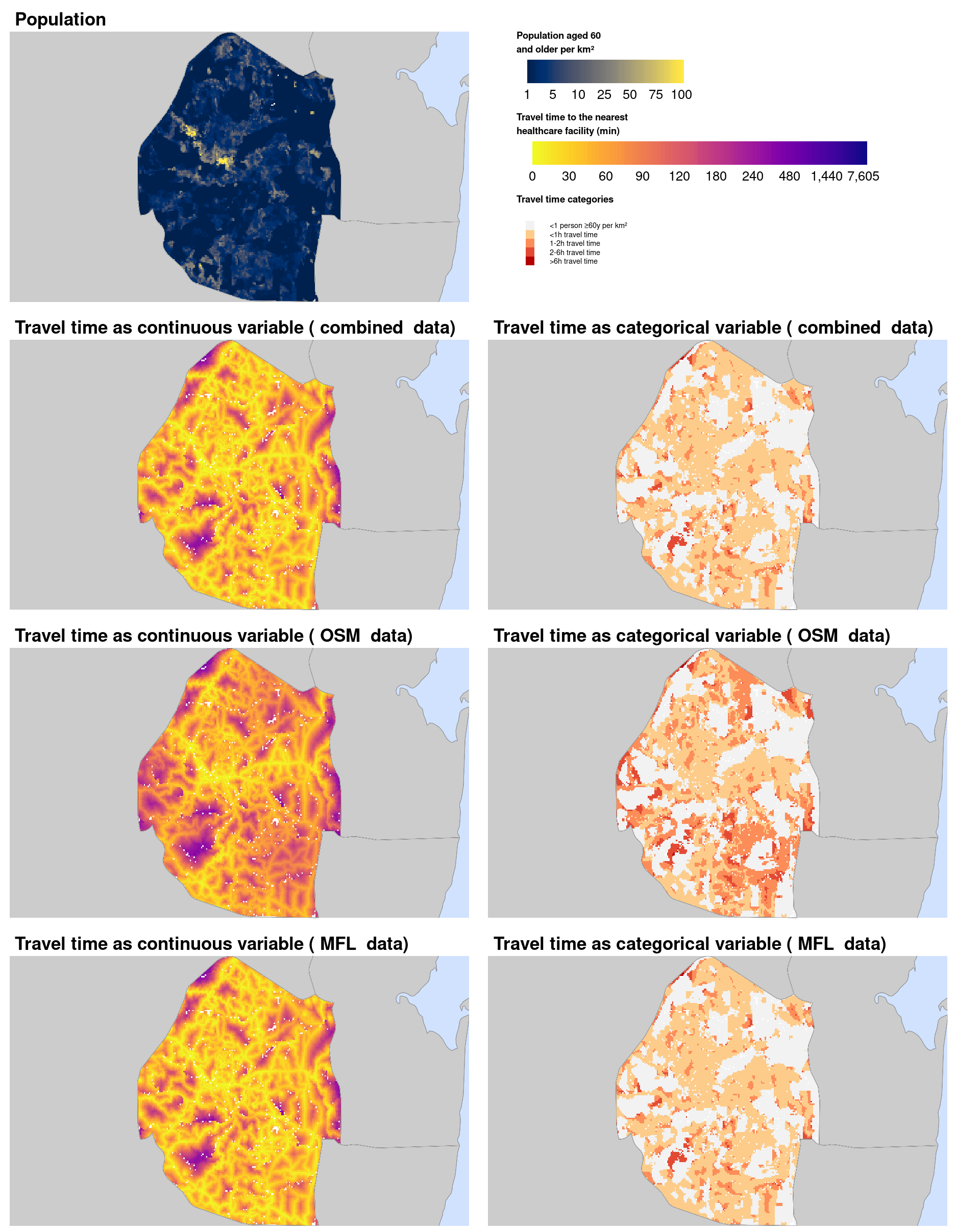


# **Figure S66. Ethiopia map of travel time to the nearest healthcare facility for adults aged ≥ 60 years**


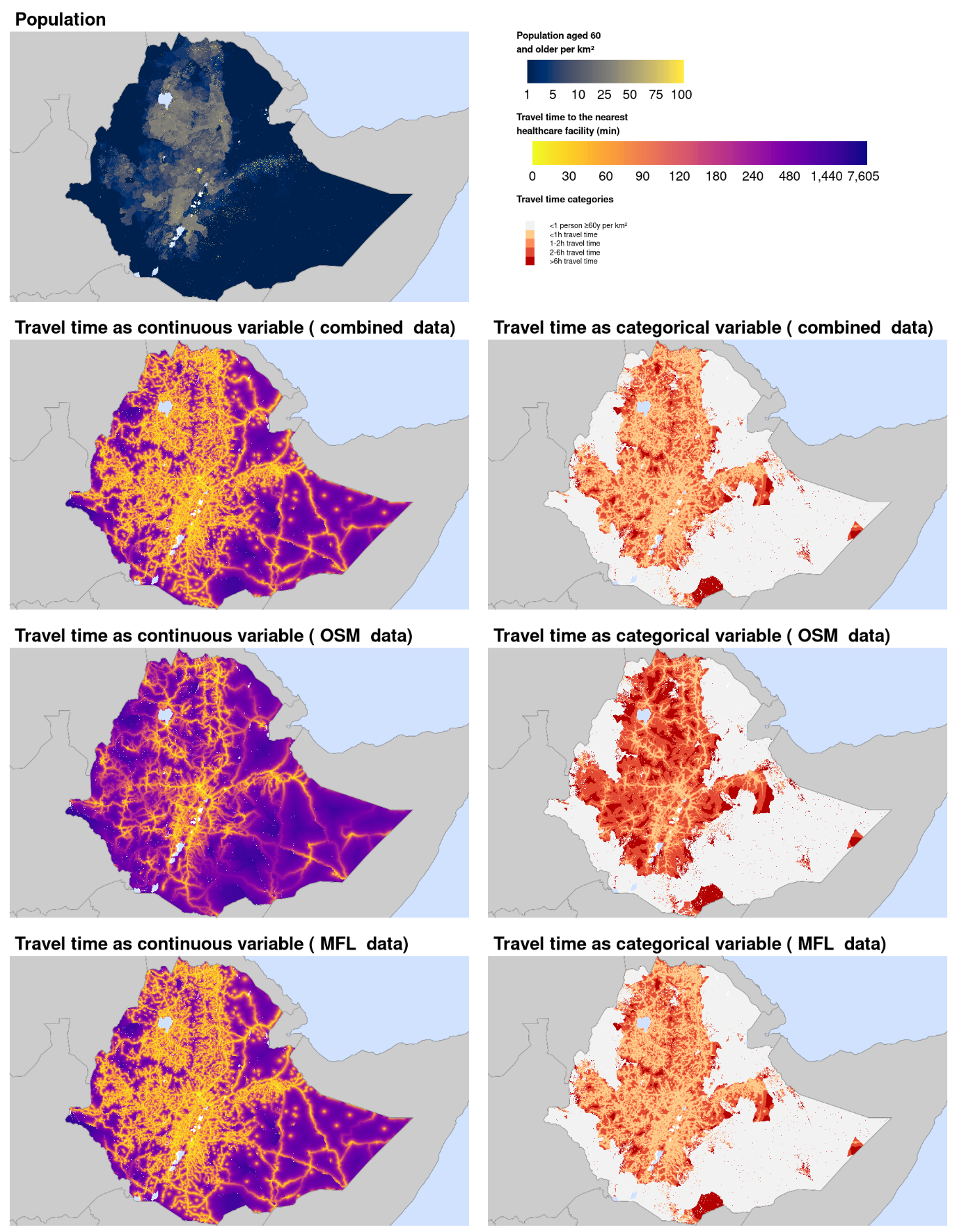


# **Figure S67. Gabon map of travel time to the nearest healthcare facility for adults aged ≥ 60 years**


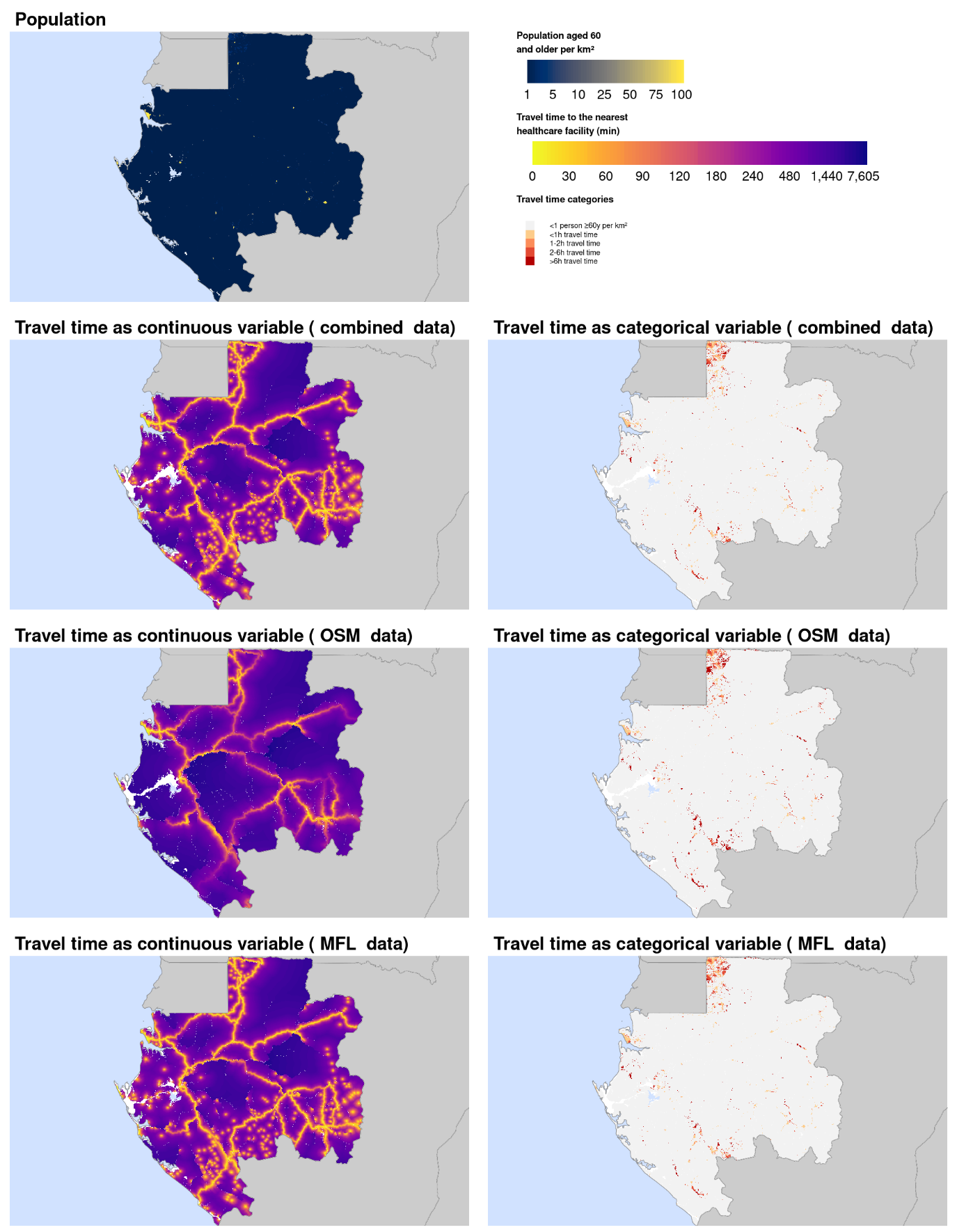


# **Figure S68. Ghana map of travel time to the nearest healthcare facility for adults aged ≥ 60 years**


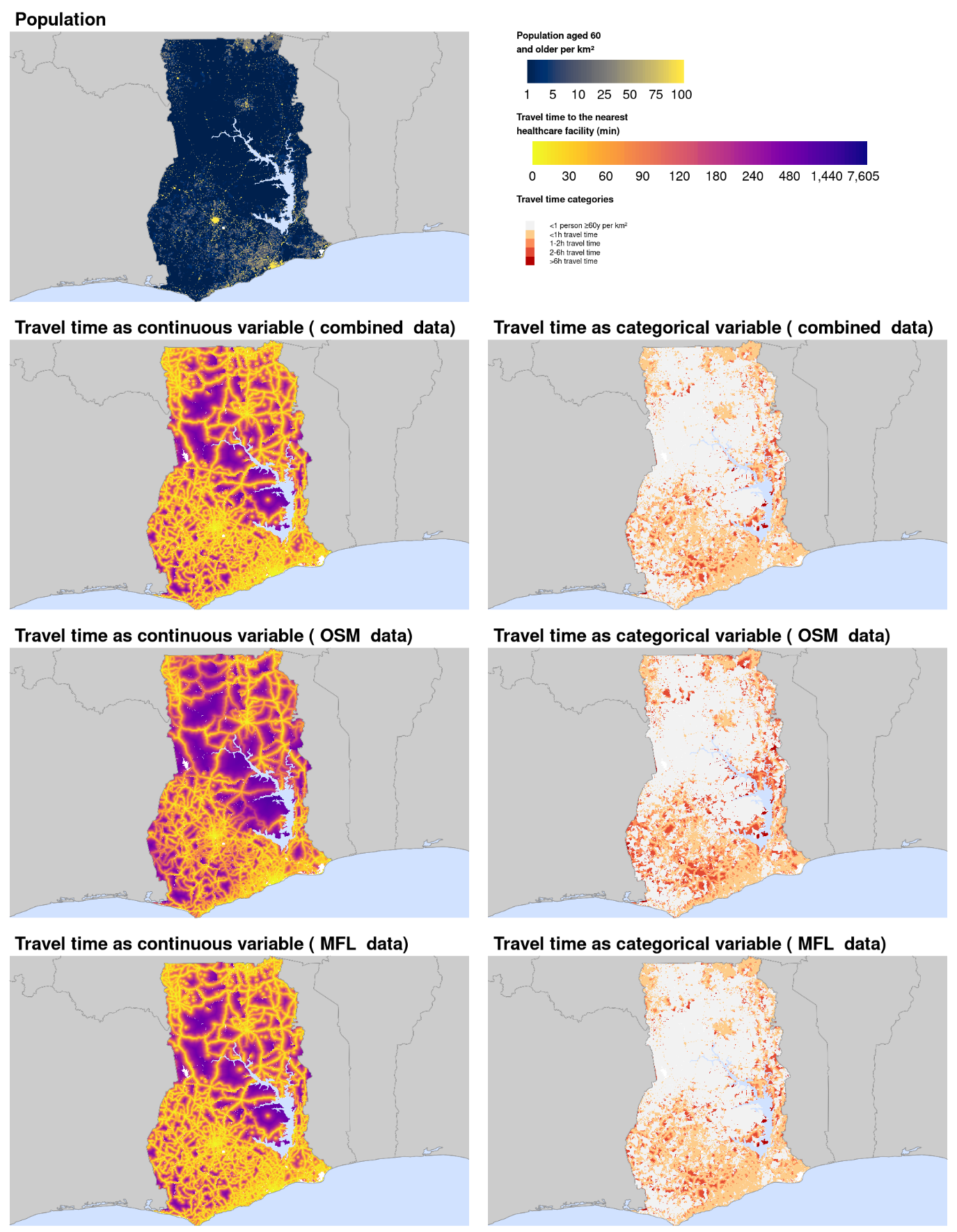


# **Figure S69. Guinea map of travel time to the nearest healthcare facility for adults aged ≥ 60 years**


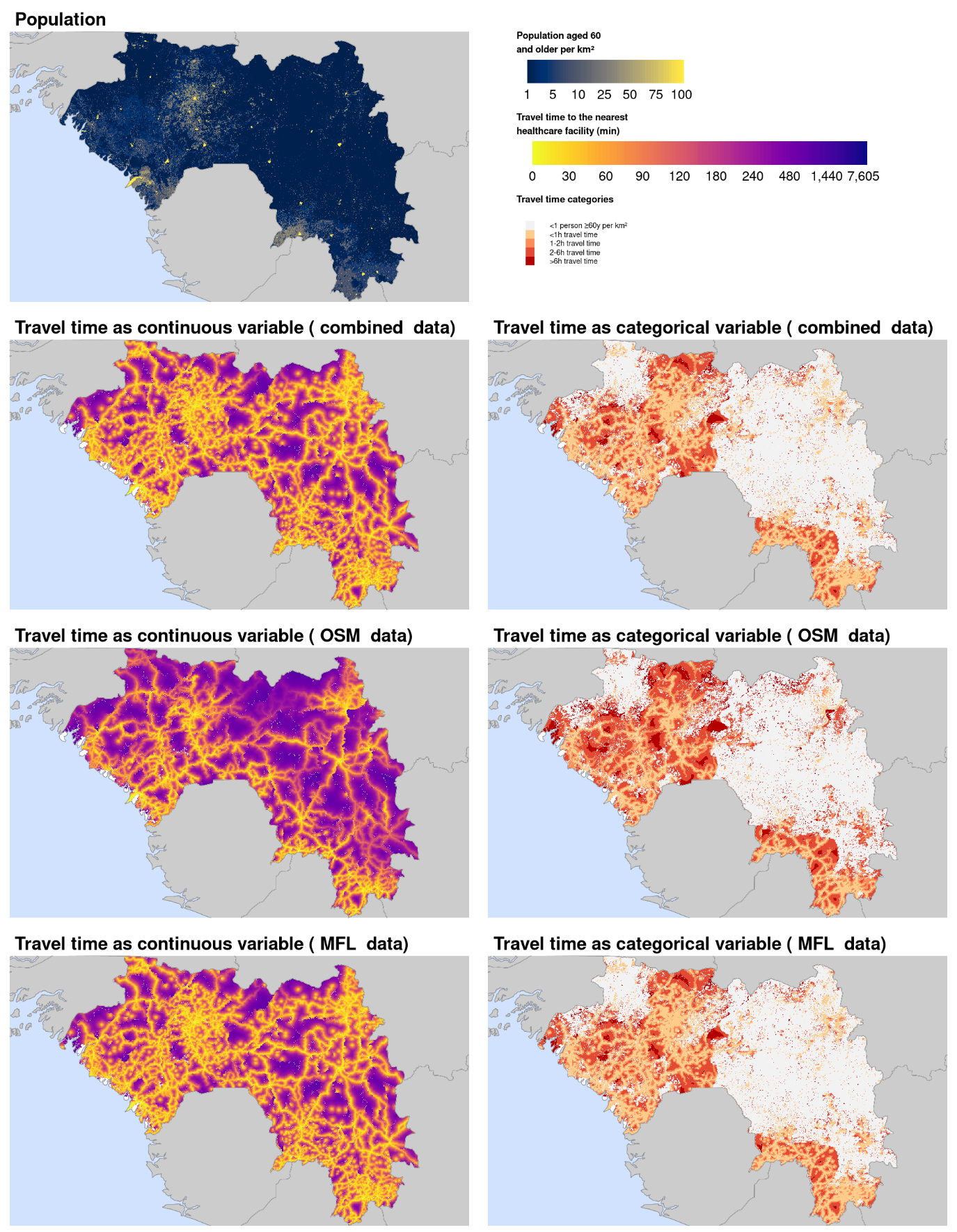


# **Figure S70. Guinea-Bissau map of travel time to the nearest healthcare facility for adults aged ≥ 60 years**


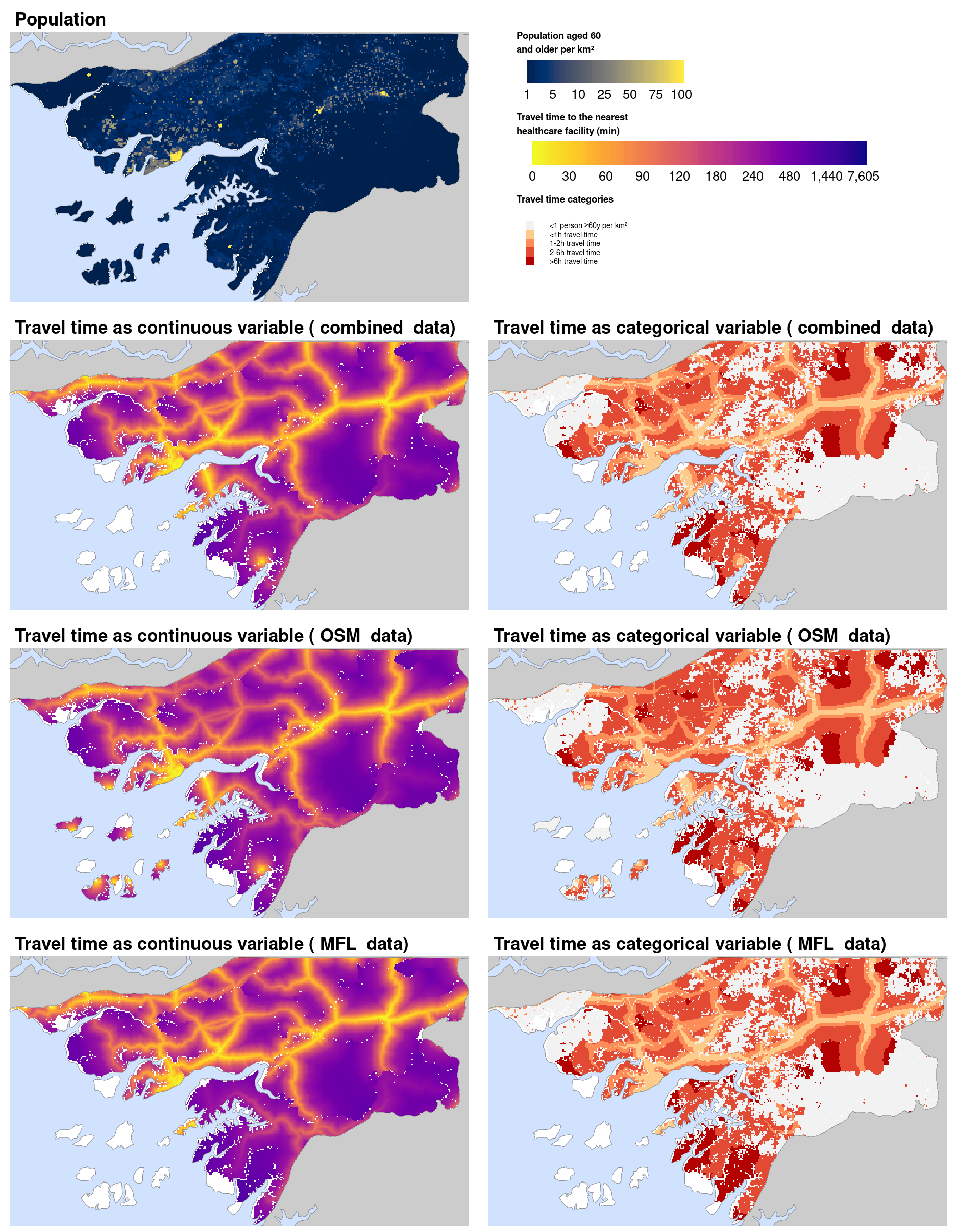


# **Figure S71. Ivory Coast map of travel time to the nearest healthcare facility for adults aged ≥ 60 years**


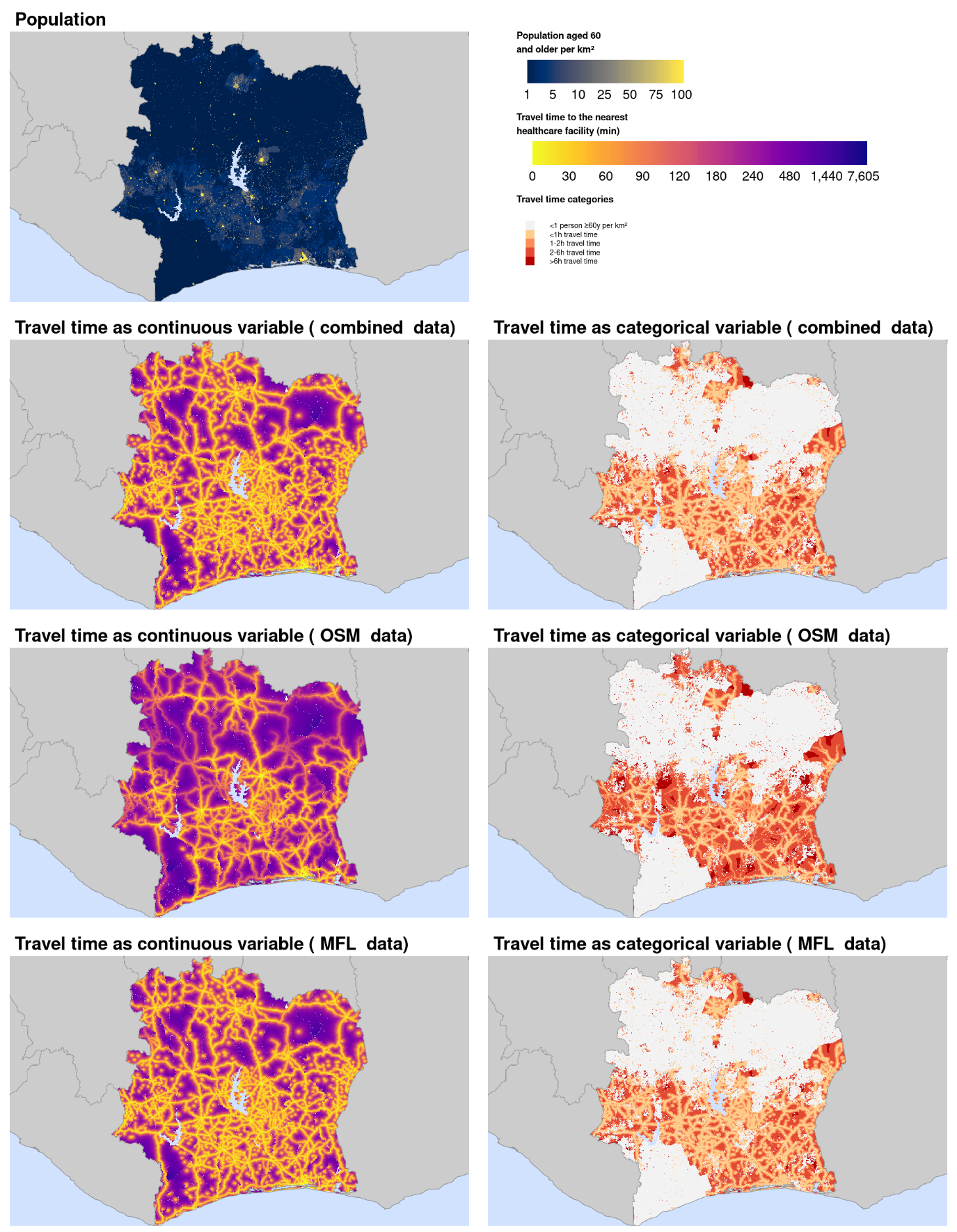


# **Figure S72. Kenya map of travel time to the nearest healthcare facility for adults aged ≥ 60 years**


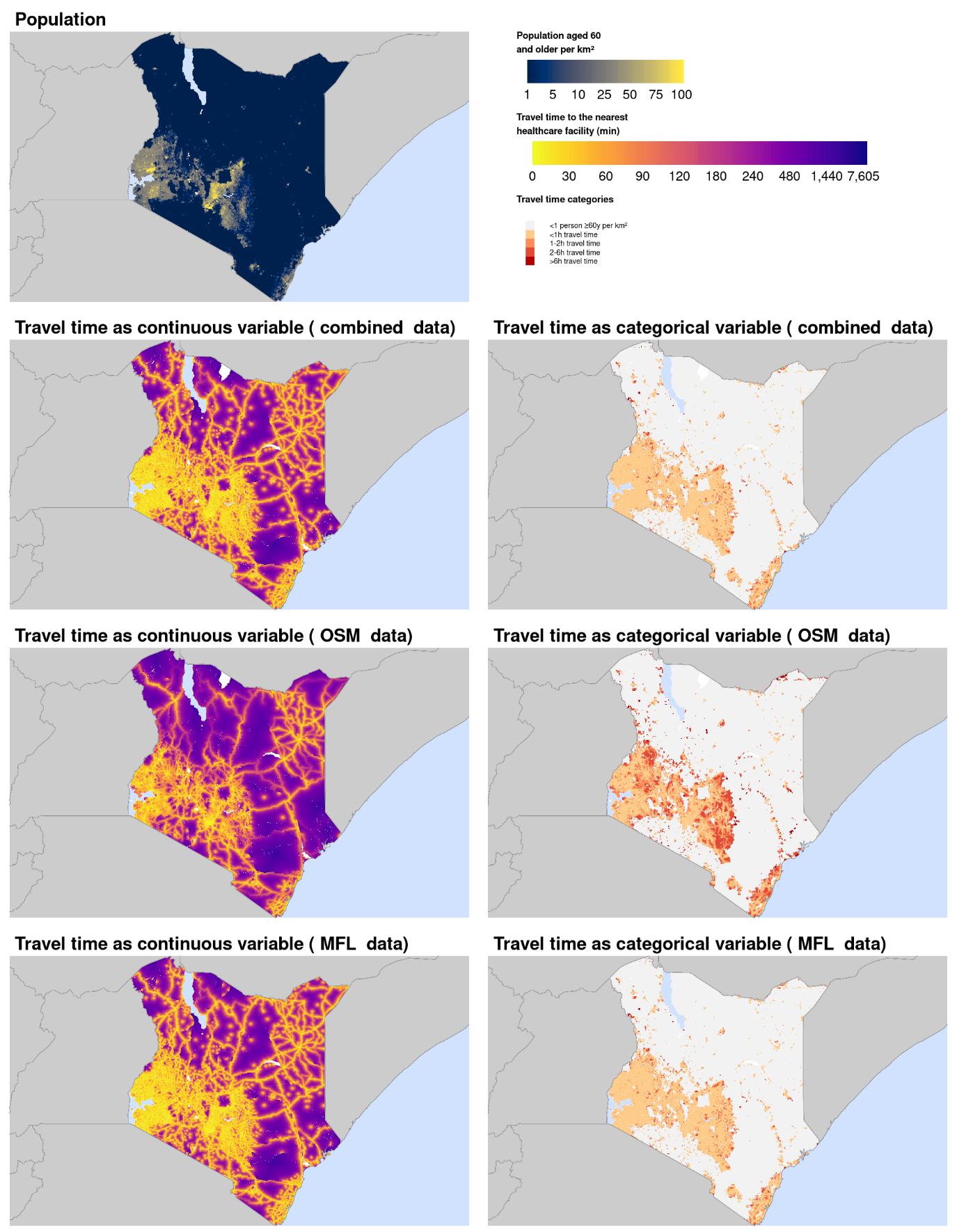


# **Figure S73. Lesotho map of travel time to the nearest healthcare facility for adults aged ≥ 60 years**


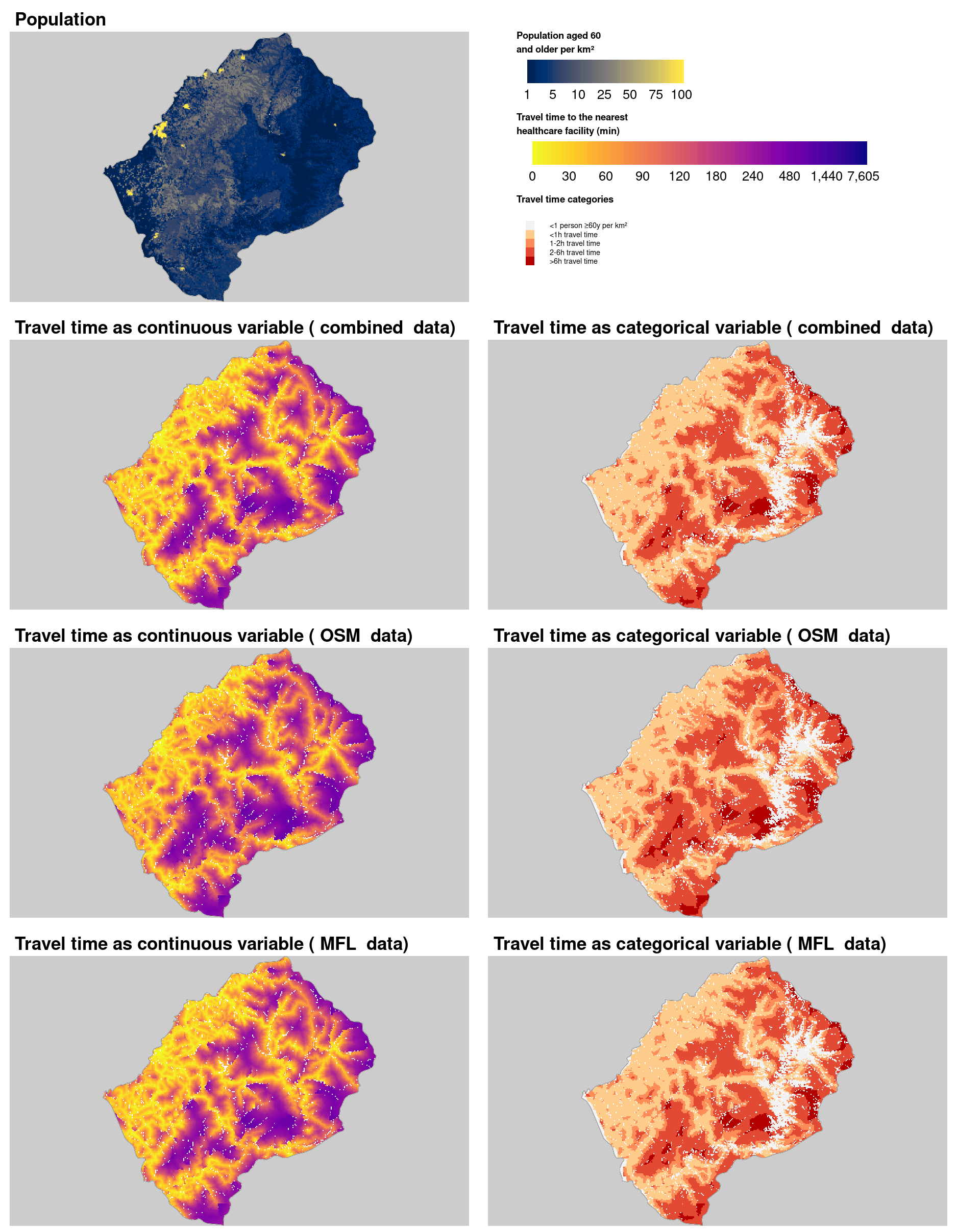


# **Figure S74. Liberia map of travel time to the nearest healthcare facility for adults aged ≥ 60 years**


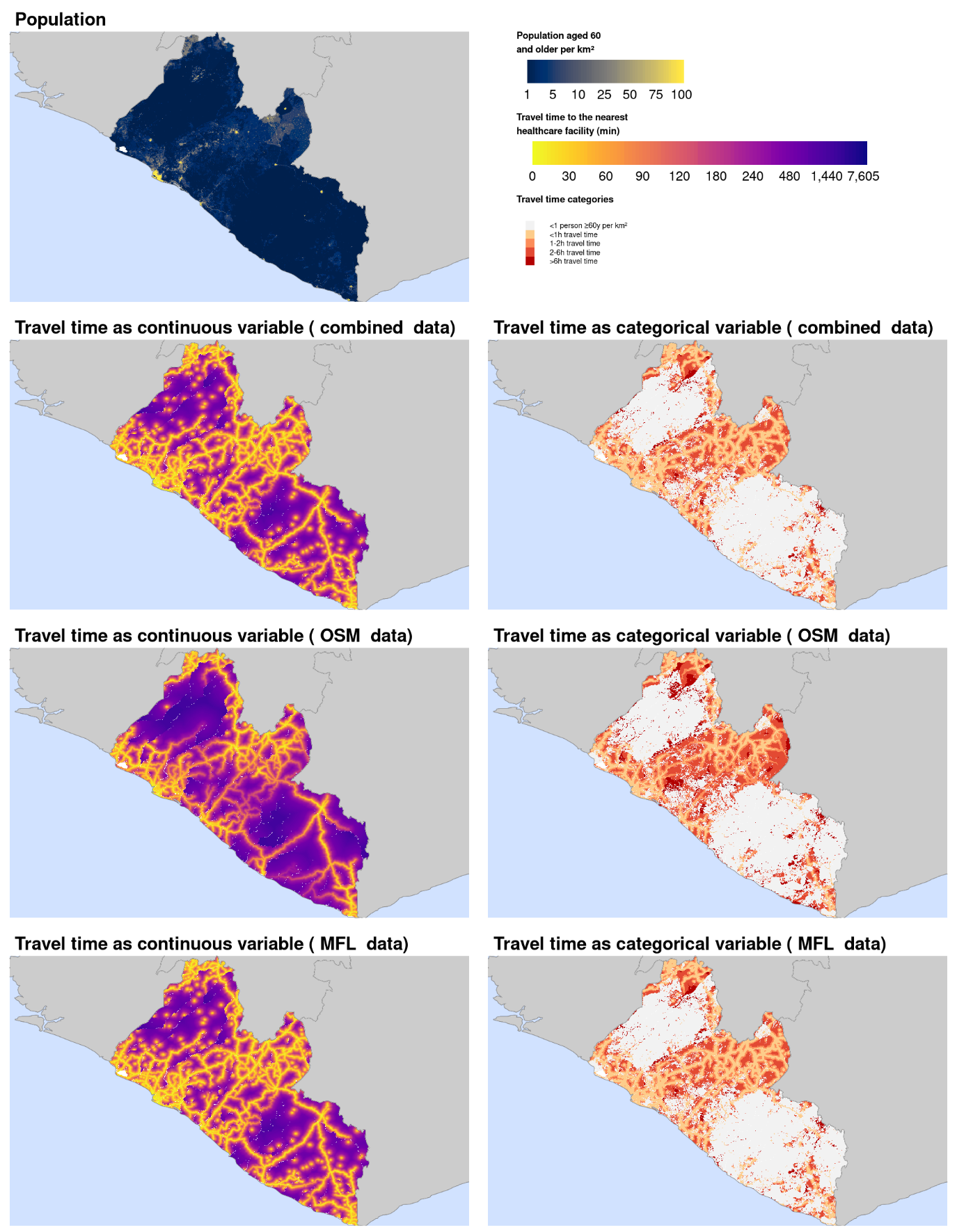


# **Figure S75. Madagascar map of travel time to the nearest healthcare facility for adults aged ≥ 60 years**


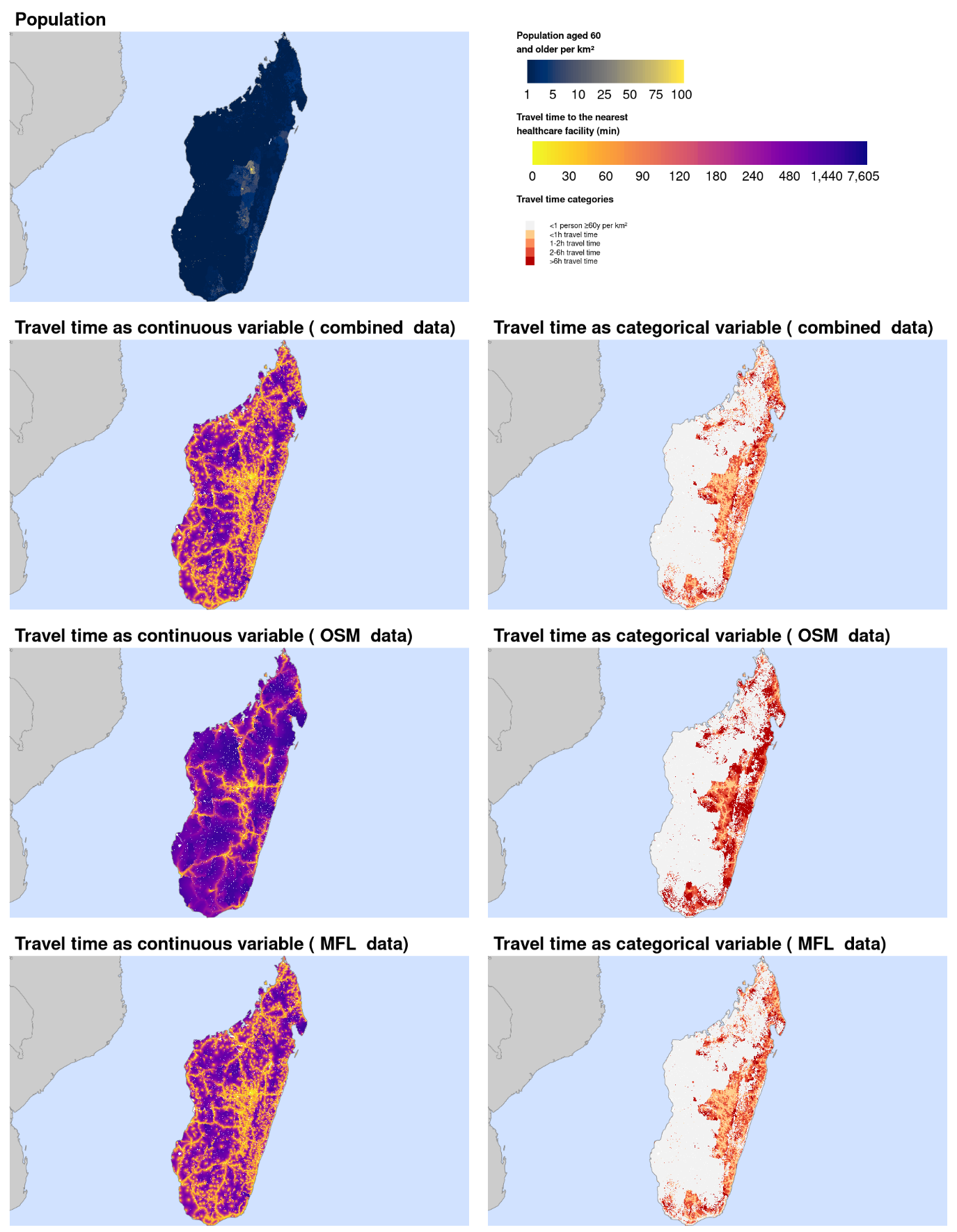


# **Figure S76. Malawi map of travel time to the nearest healthcare facility for adults aged ≥ 60 years**


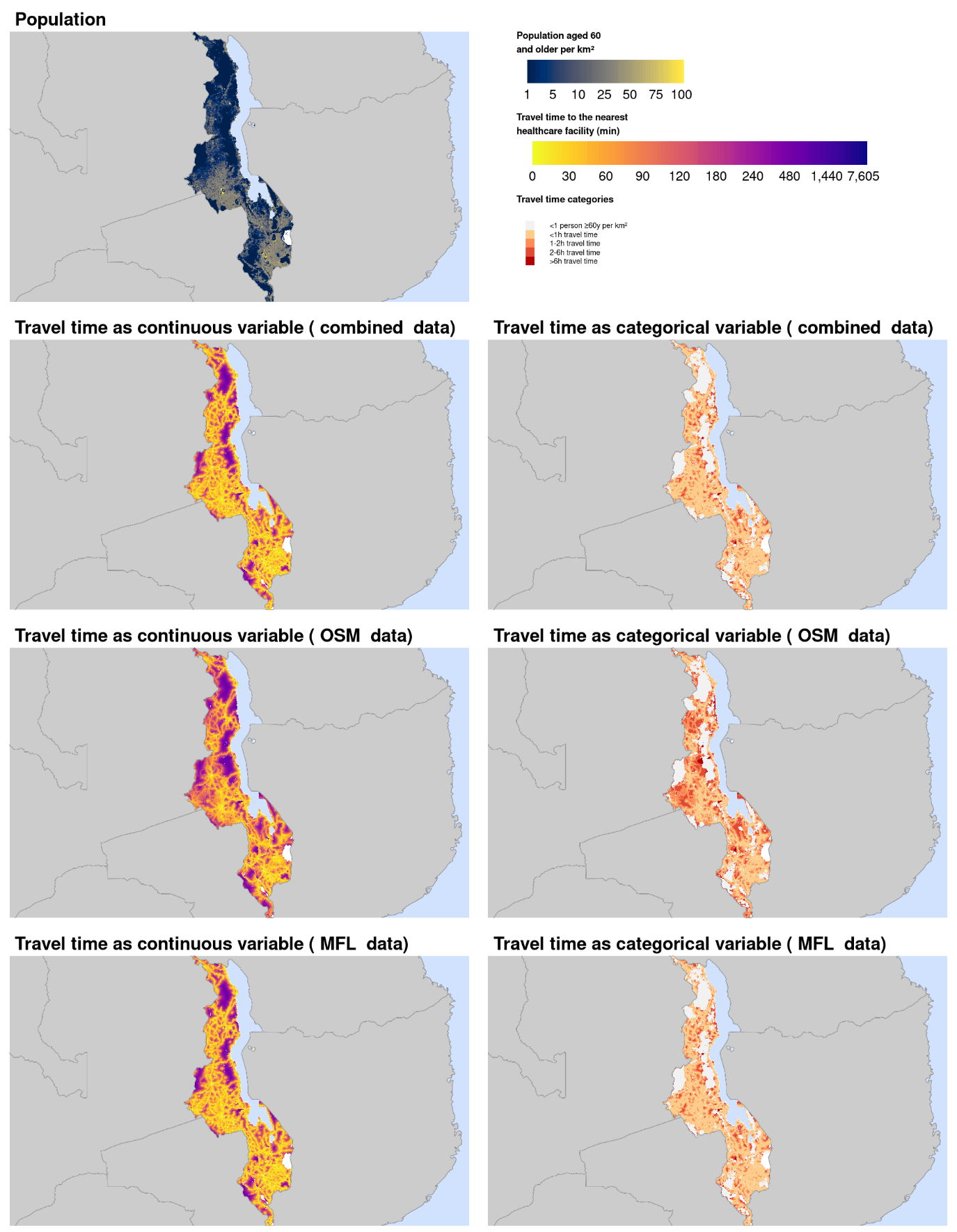


# **Figure S77. Mali map of travel time to the nearest healthcare facility for adults aged ≥ 60 years**


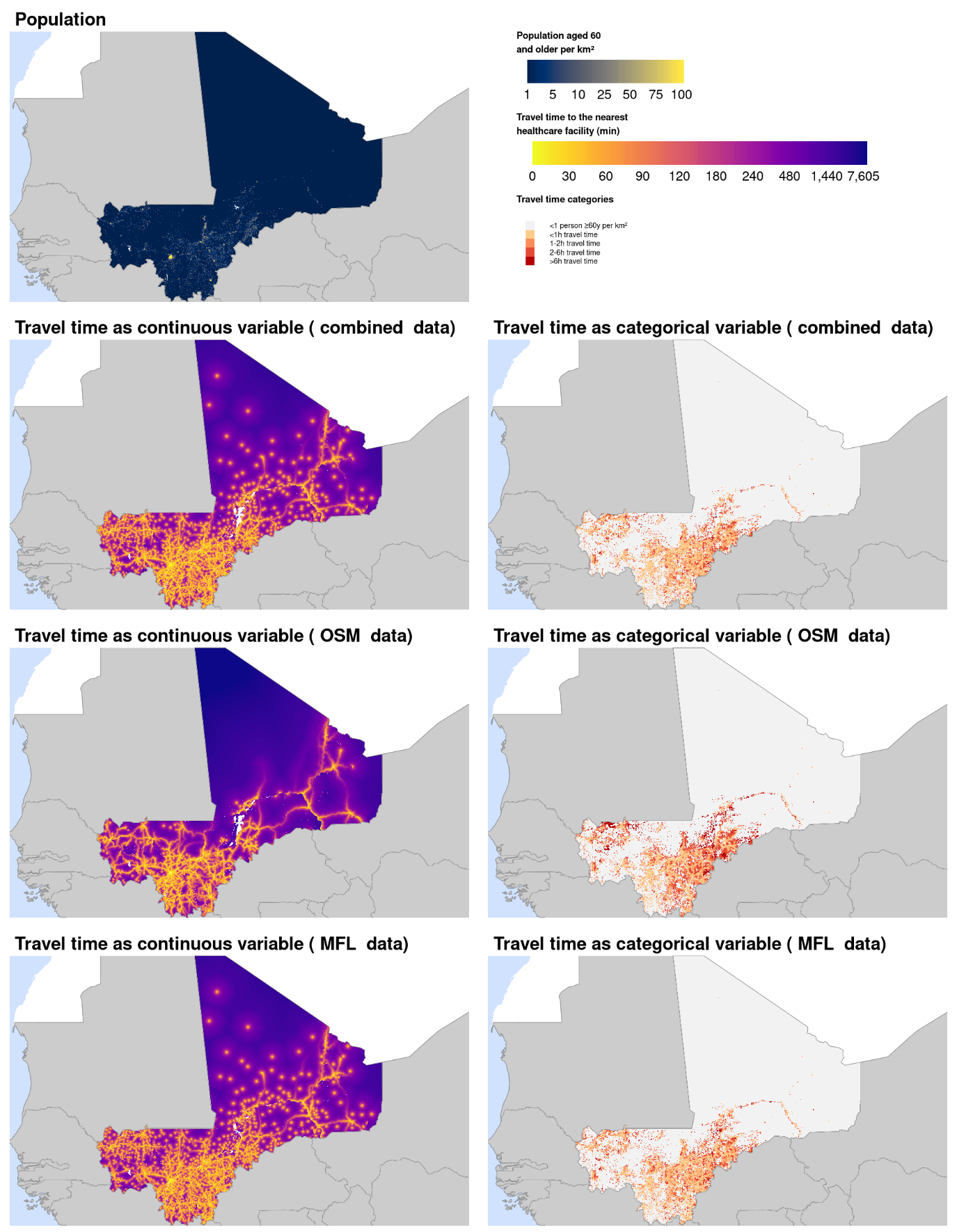


# **Figure S78. Mauritania map of travel time to the nearest healthcare facility for adults aged ≥ 60 years**


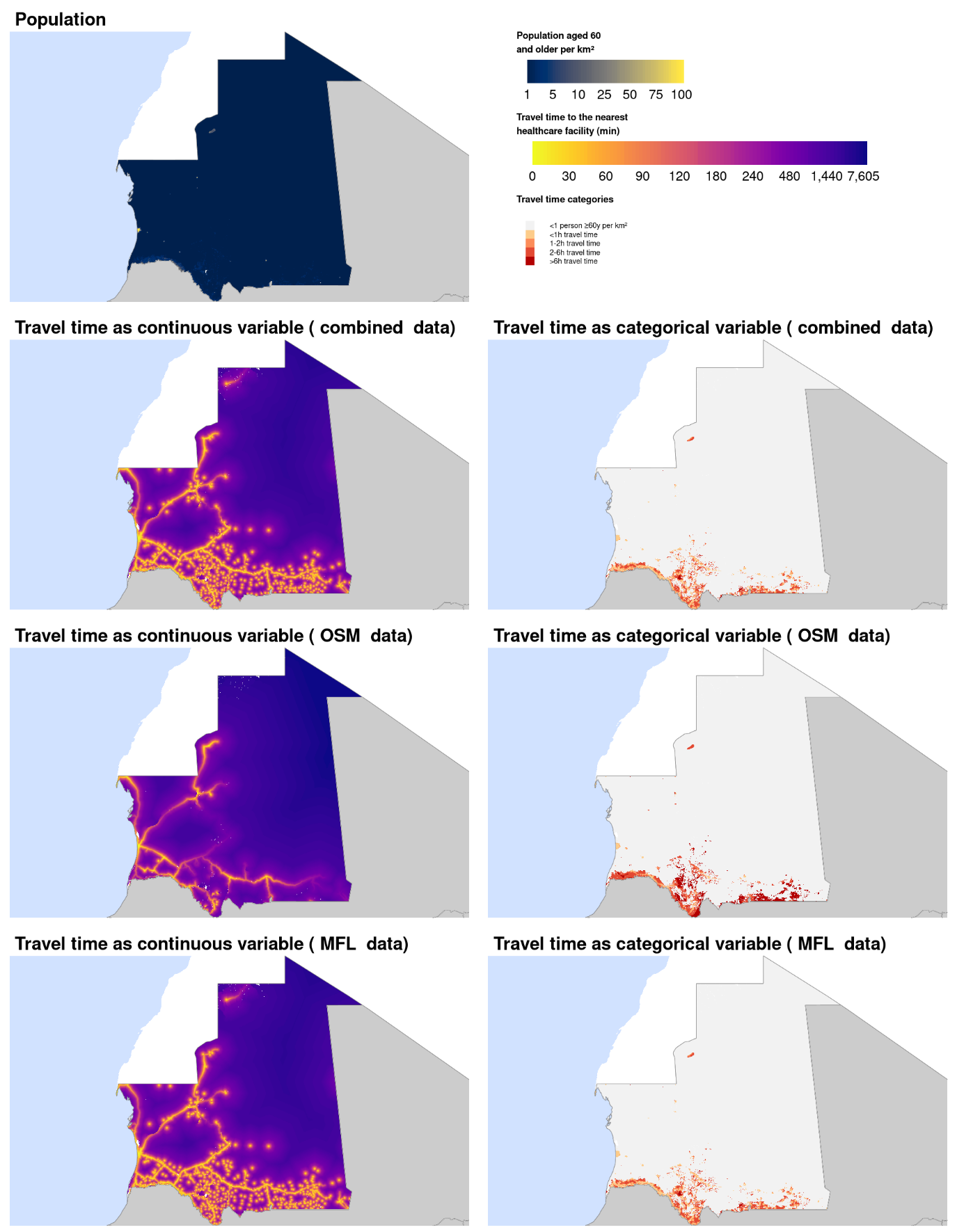


# **Figure S79. Mozambique map of travel time to the nearest healthcare facility for adults aged ≥ 60 years**


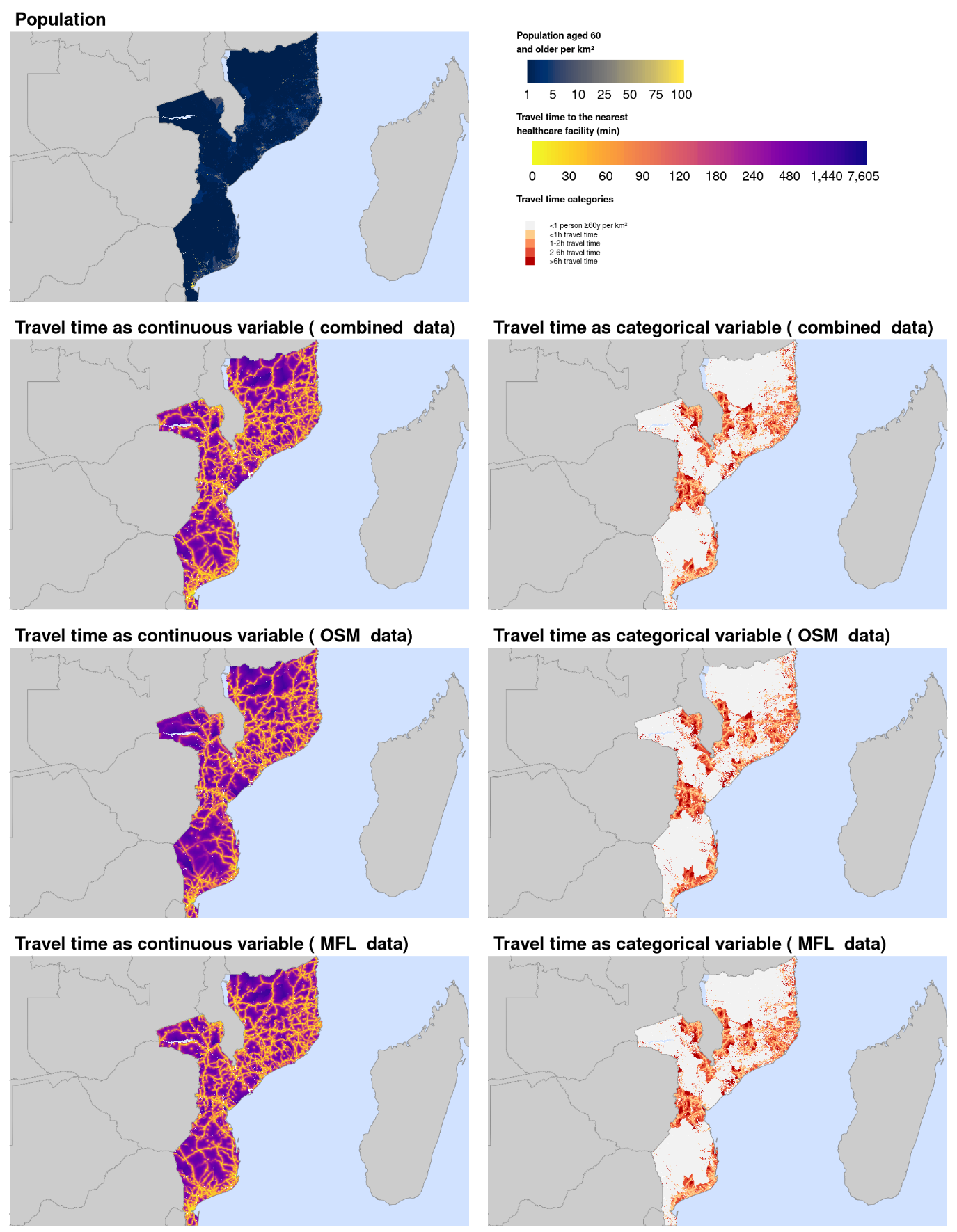


# **Figure S80. Namibia map of travel time to the nearest healthcare facility for adults aged ≥ 60 years**


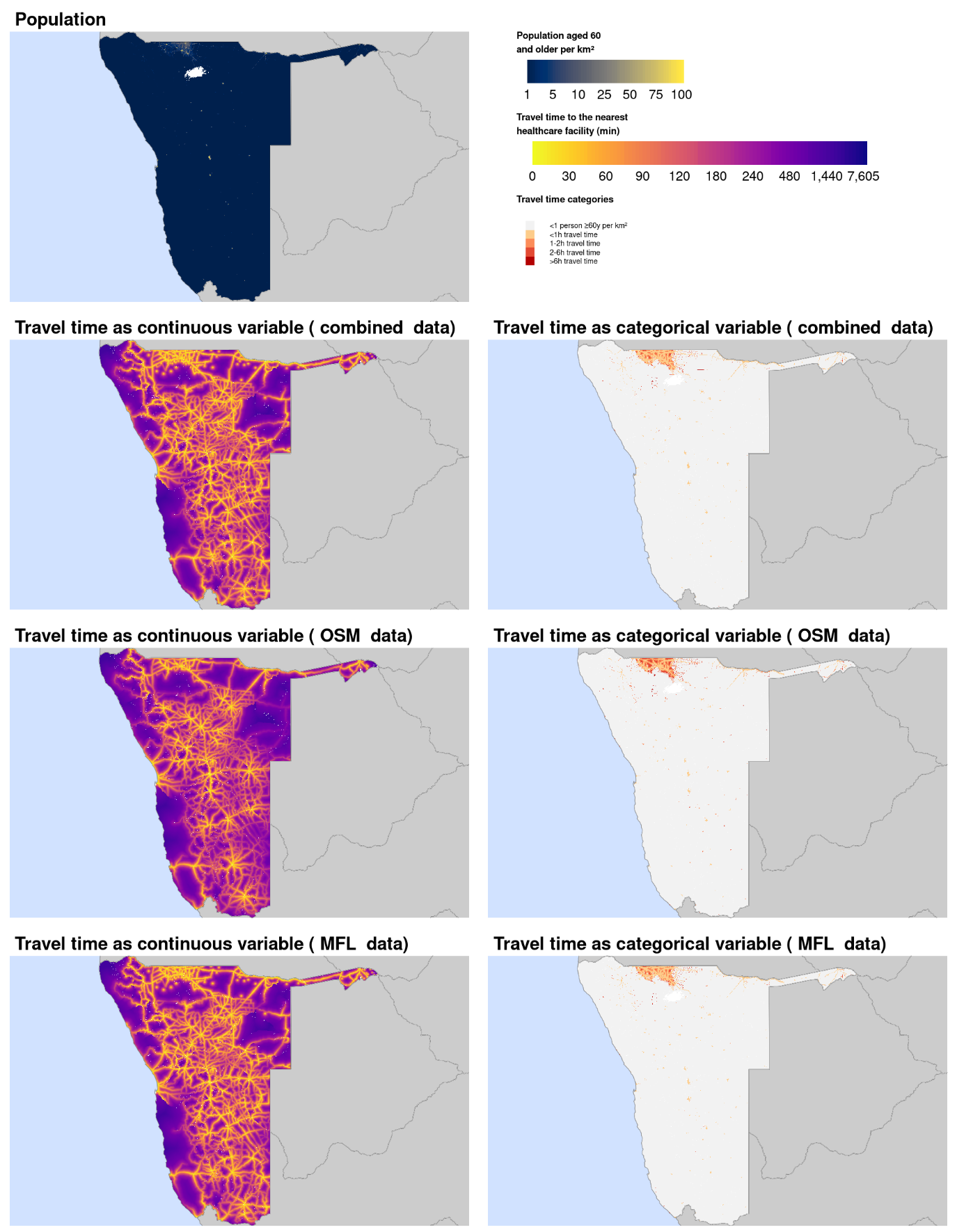


# **Figure S81. Niger map of travel time to the nearest healthcare facility for adults aged ≥ 60 years**


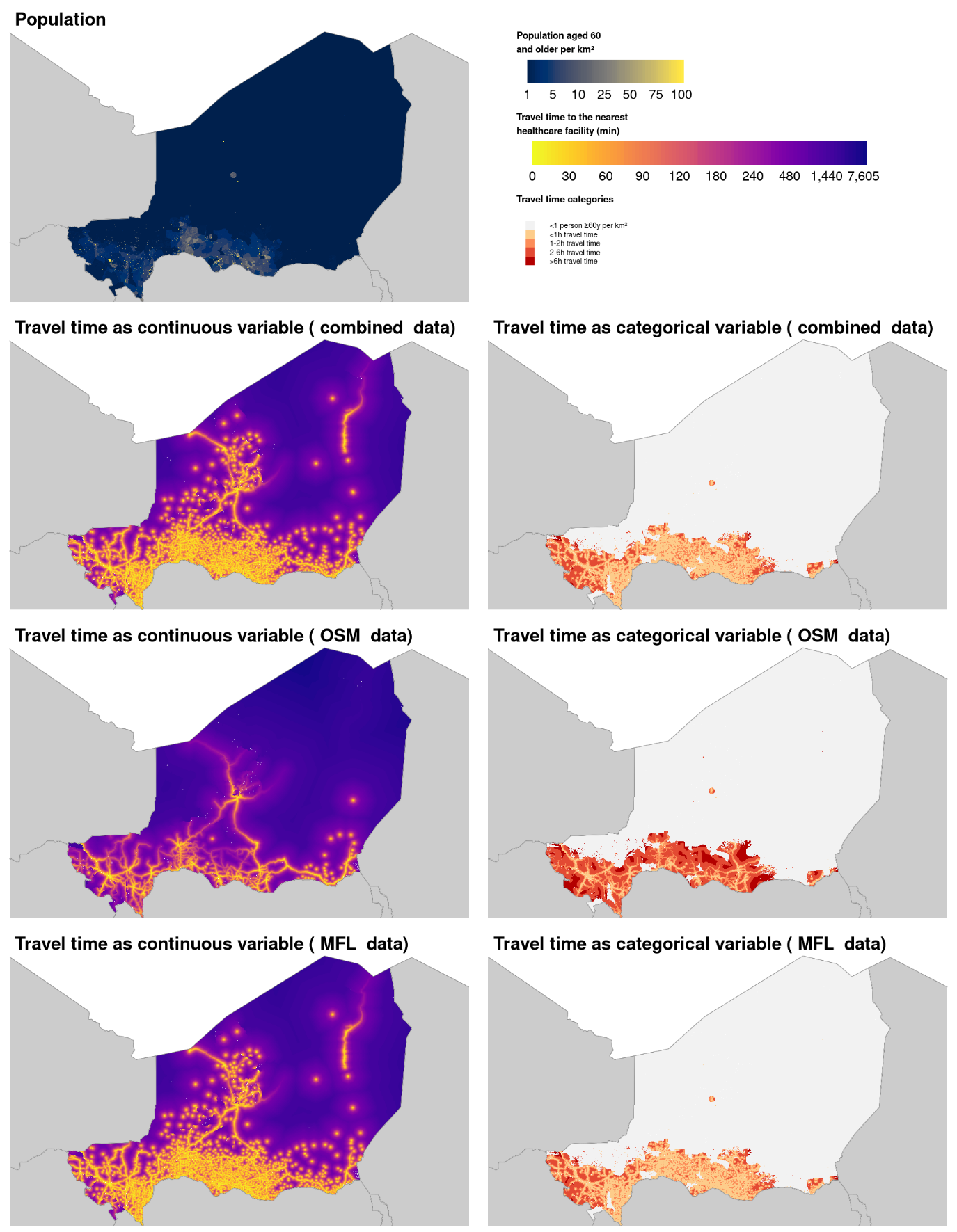


# **Figure S82. Nigeria map of travel time to the nearest healthcare facility for adults aged ≥ 60 years**


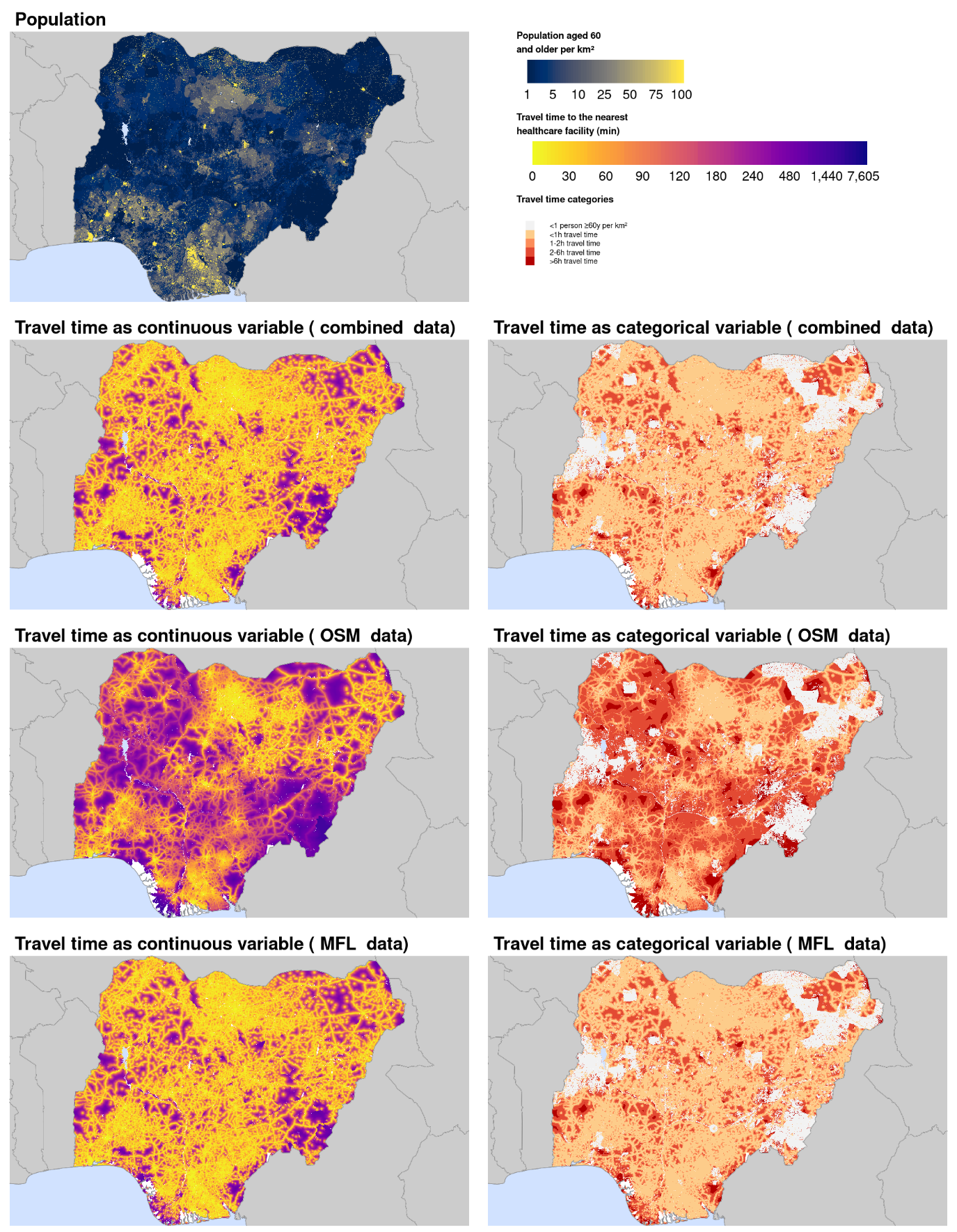


# **Figure S83. Republic of the Congo map of travel time to the nearest healthcare facility for adults aged ≥ 60 years**


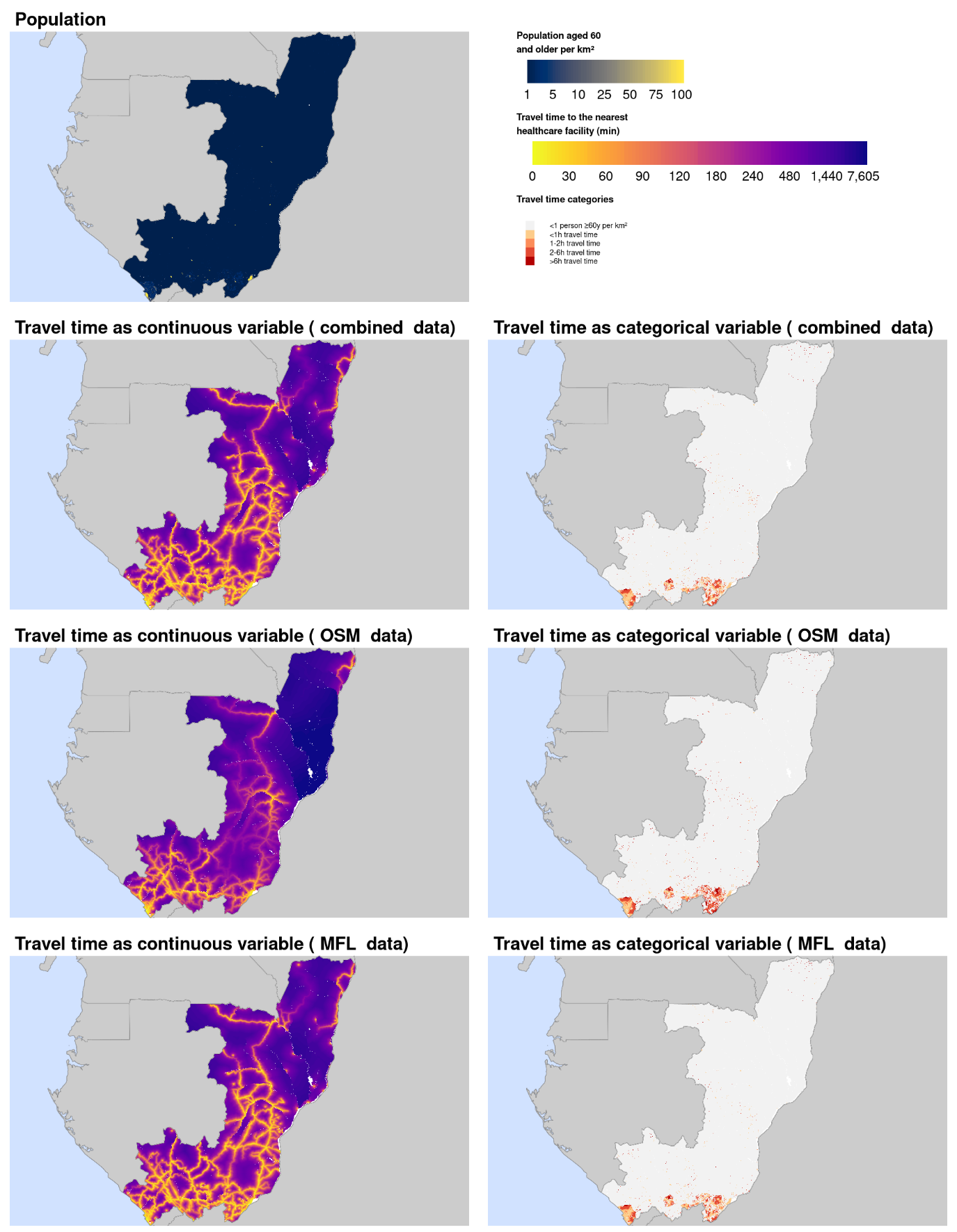


# **Figure S84. Rwanda map of travel time to the nearest healthcare facility for adults aged ≥ 60 years**


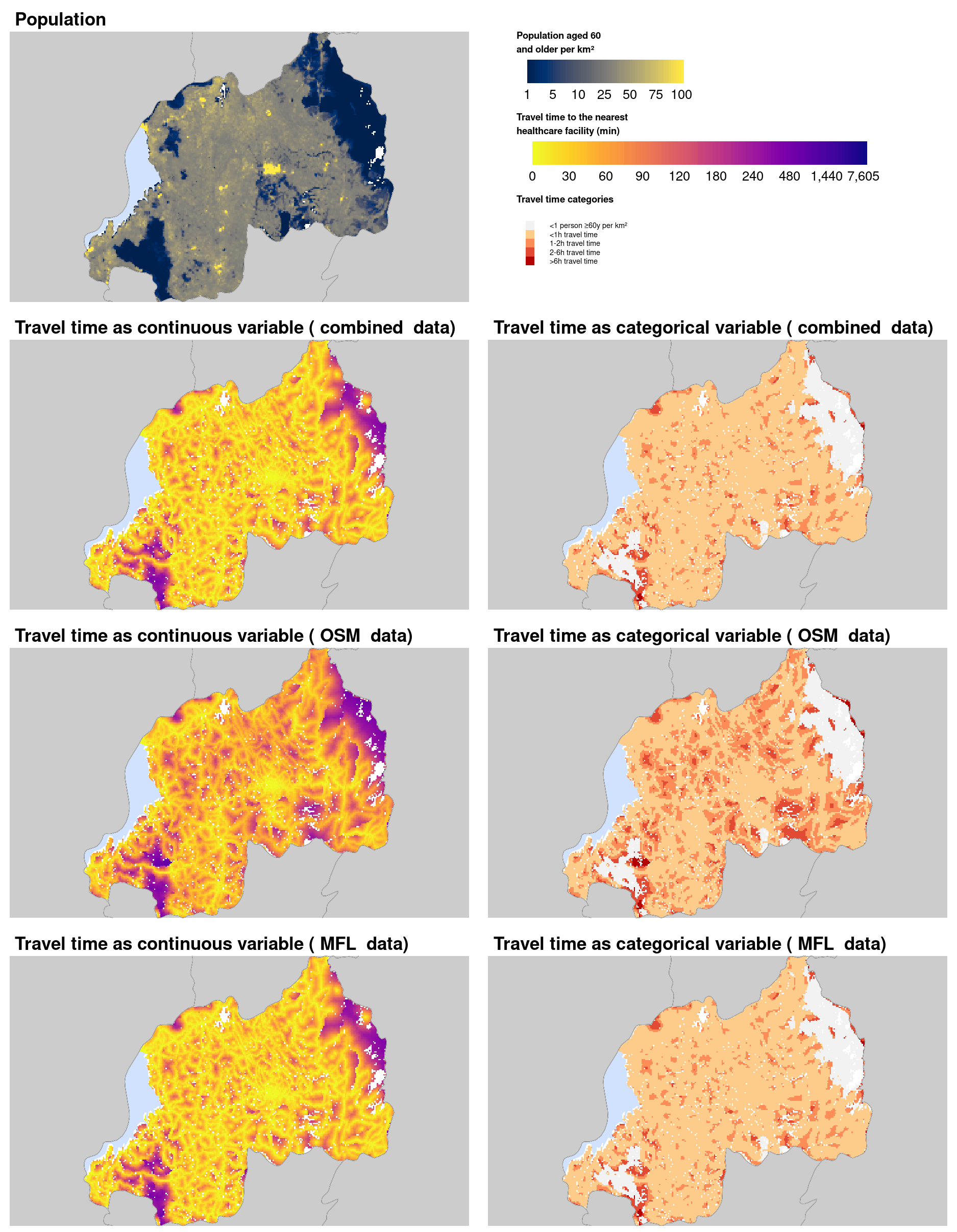


# **Figure S85. Senegal map of travel time to the nearest healthcare facility for adults aged ≥ 60 years**


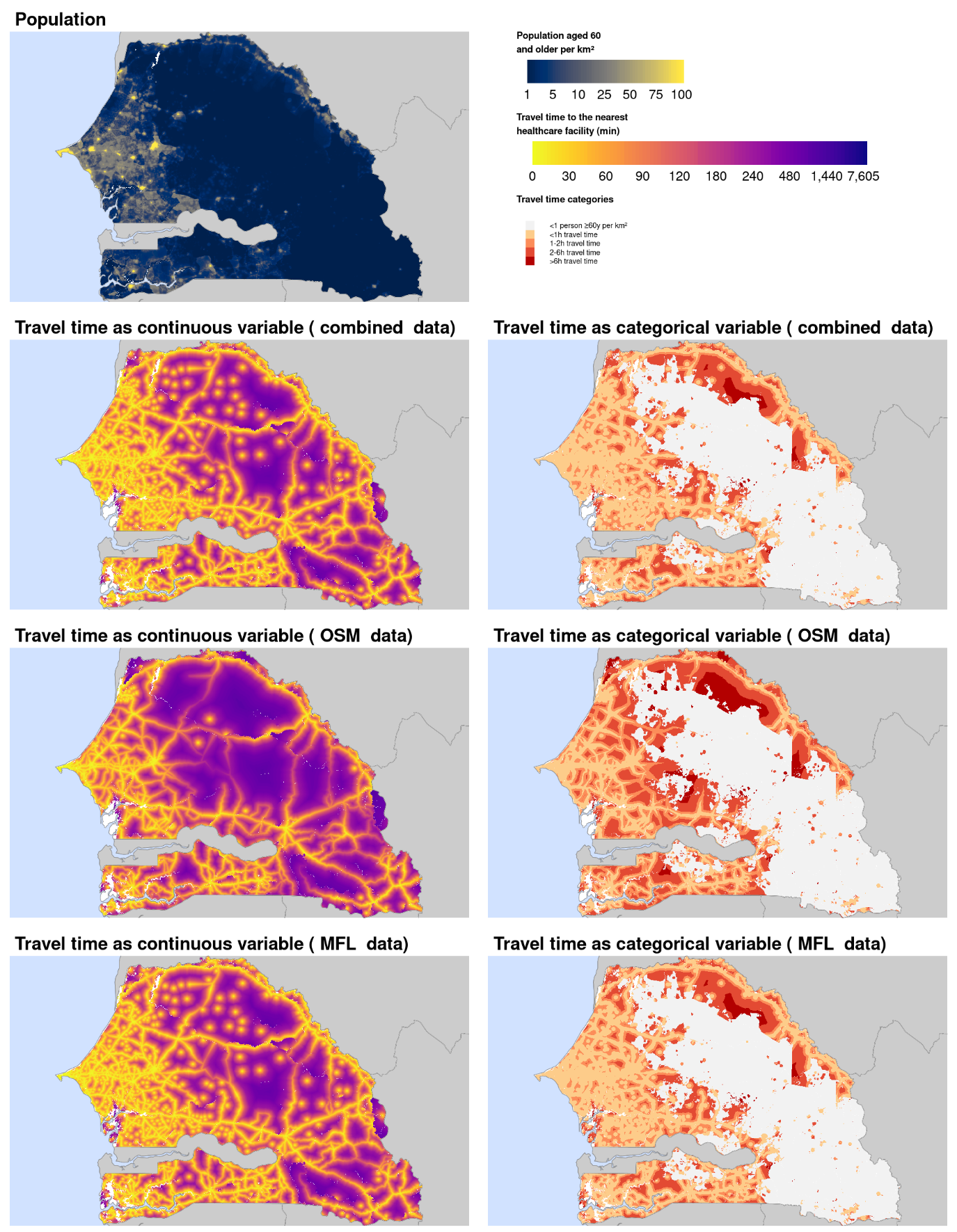


# **Figure S86. Sierra Leone map of travel time to the nearest healthcare facility for adults aged ≥ 60 years**


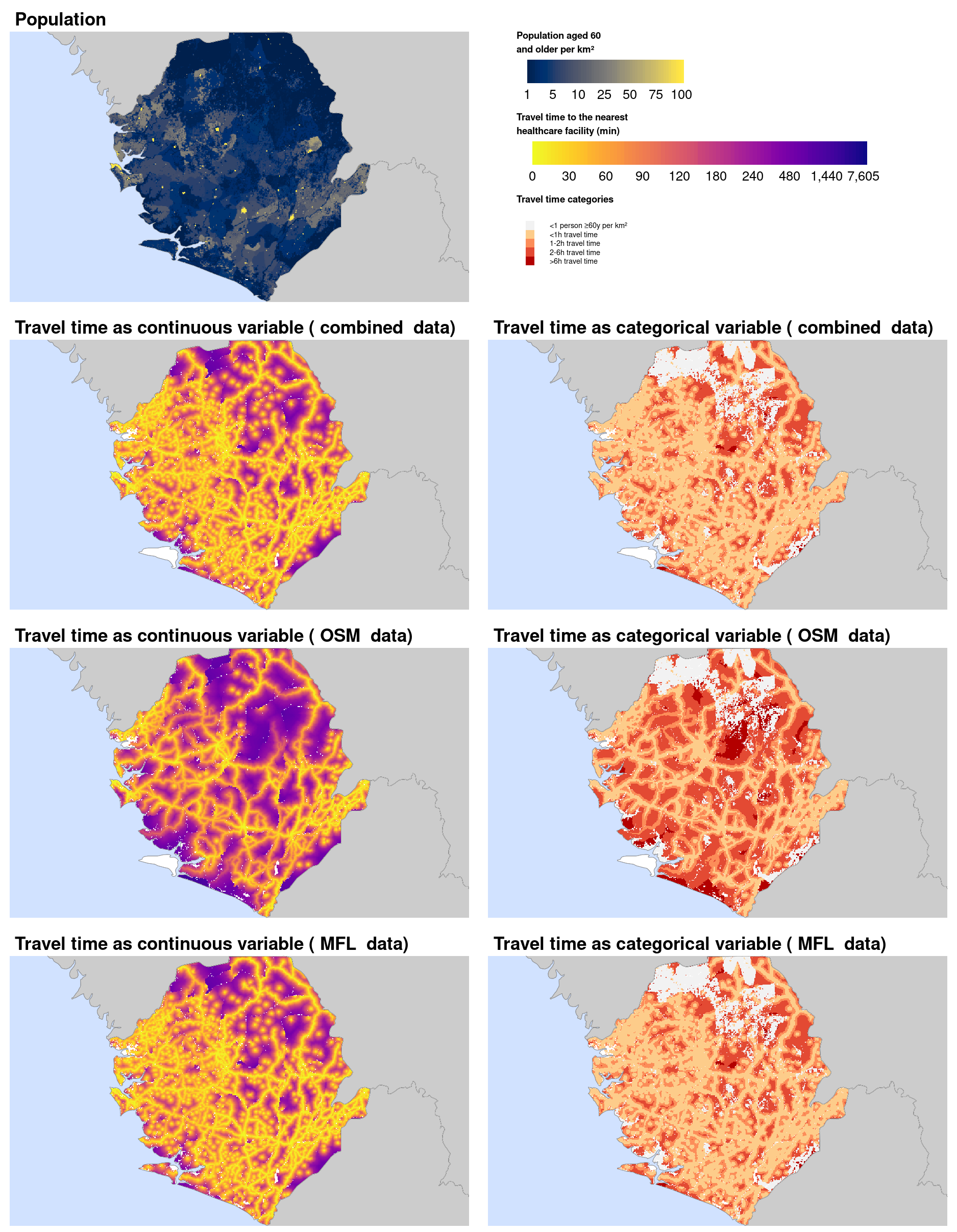


# **Figure S87. Somalia map of travel time to the nearest healthcare facility for adults aged ≥ 60 years**


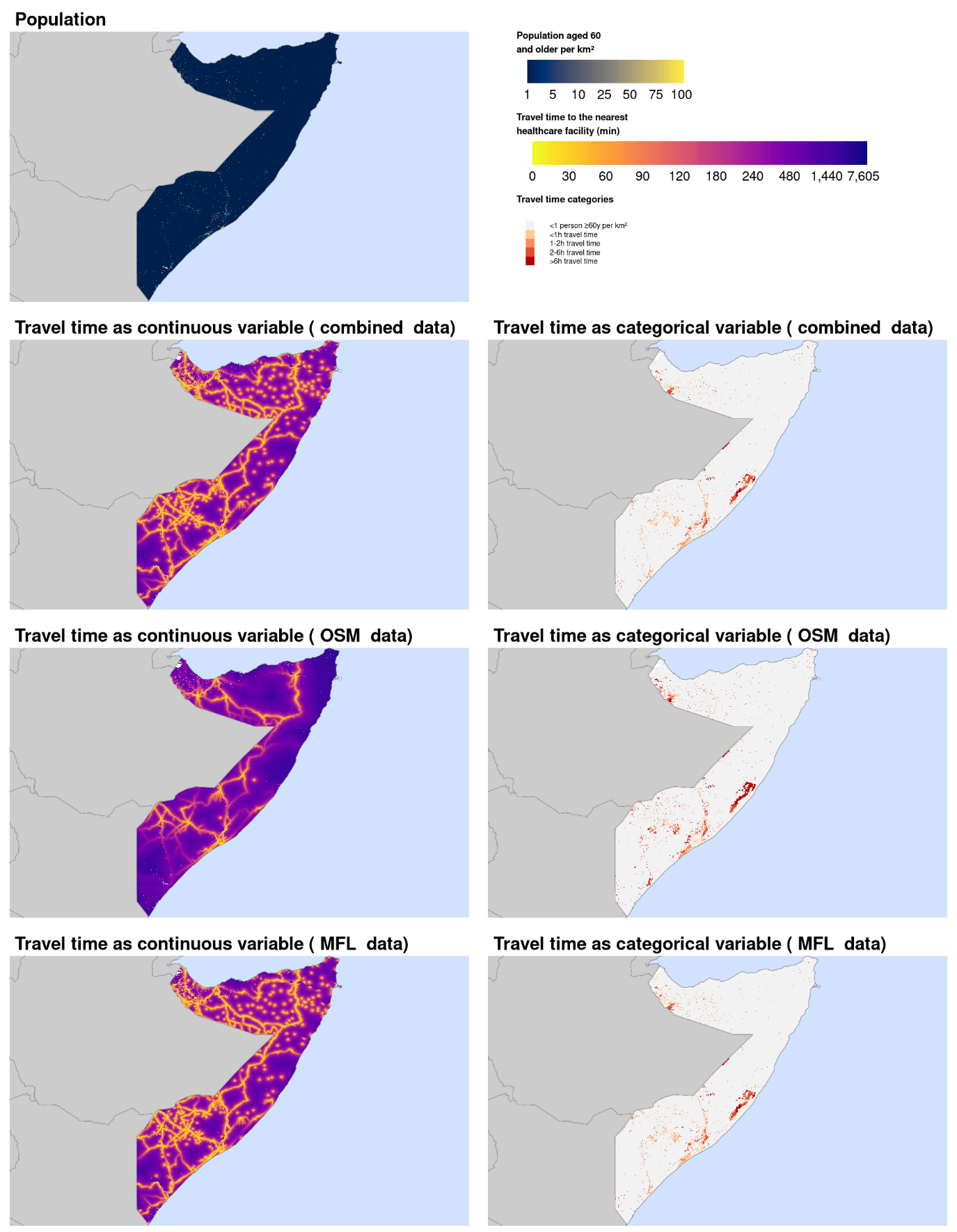


# **Figure S88. South Africa map of travel time to the nearest healthcare facility for adults aged ≥ 60 years**


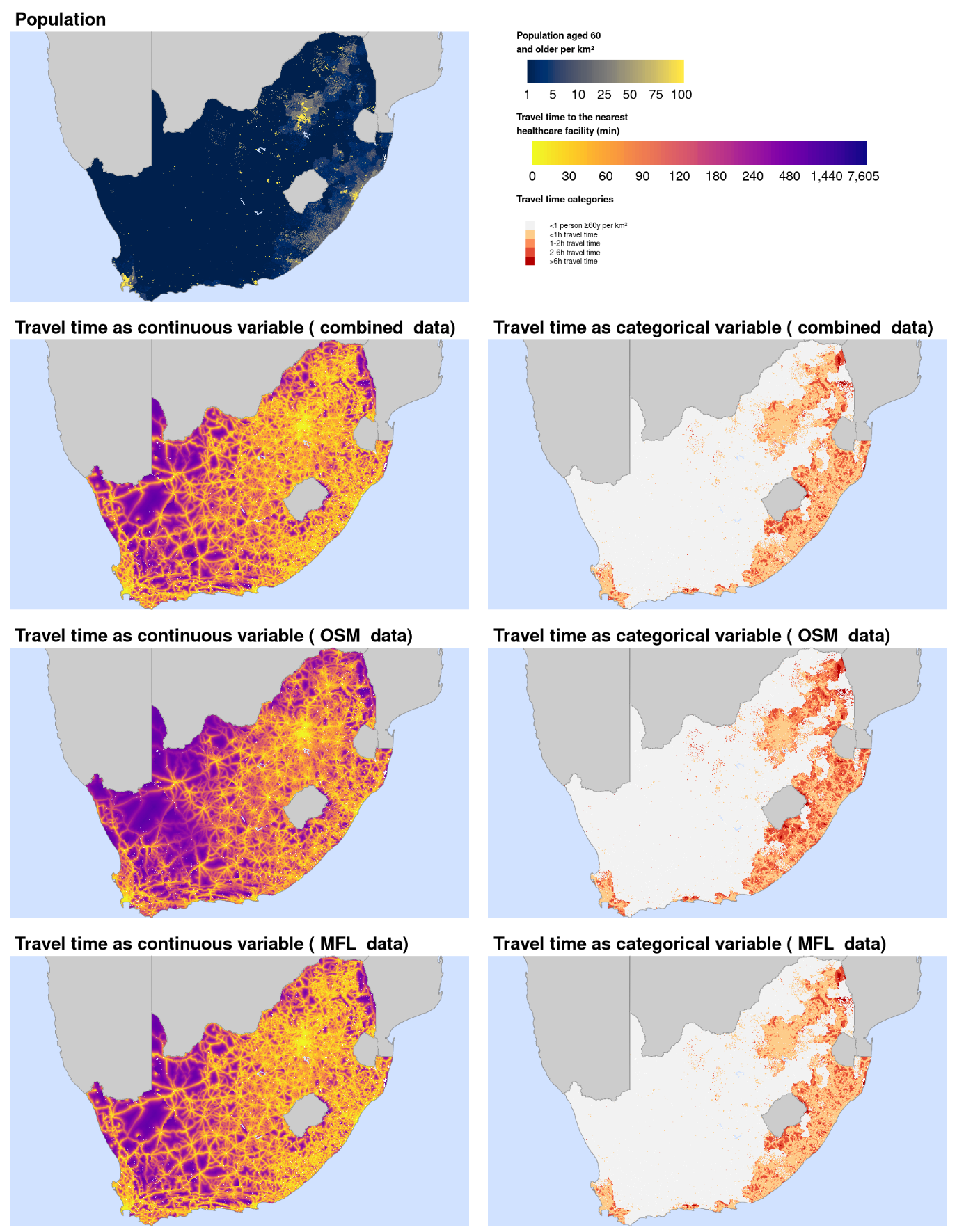


# **Figure S89. South Sudan map of travel time to the nearest healthcare facility for adults aged ≥ 60 years**

**
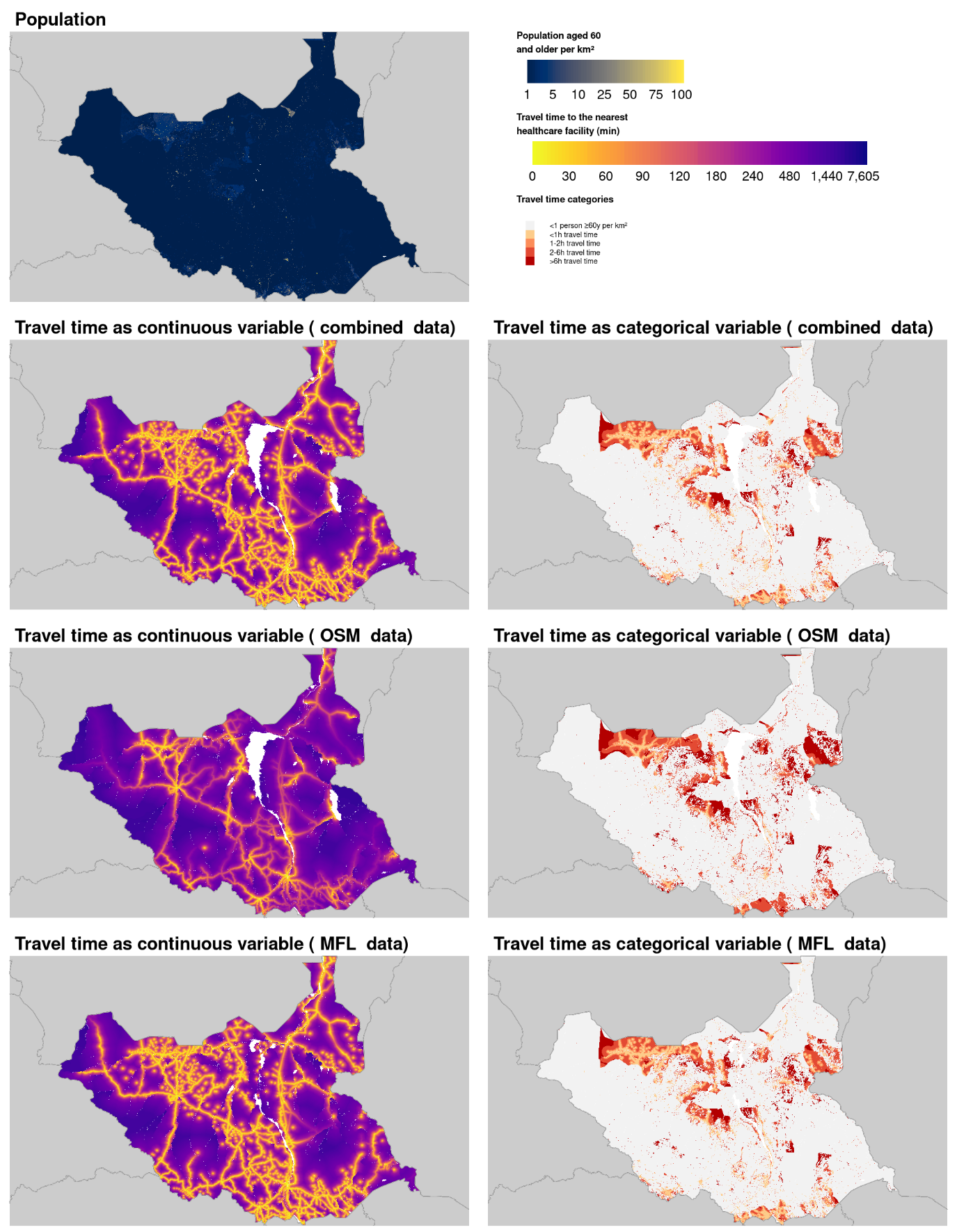
**

# **Figure S90. Sudan map of travel time to the nearest healthcare facility for adults aged ≥ 60 years**


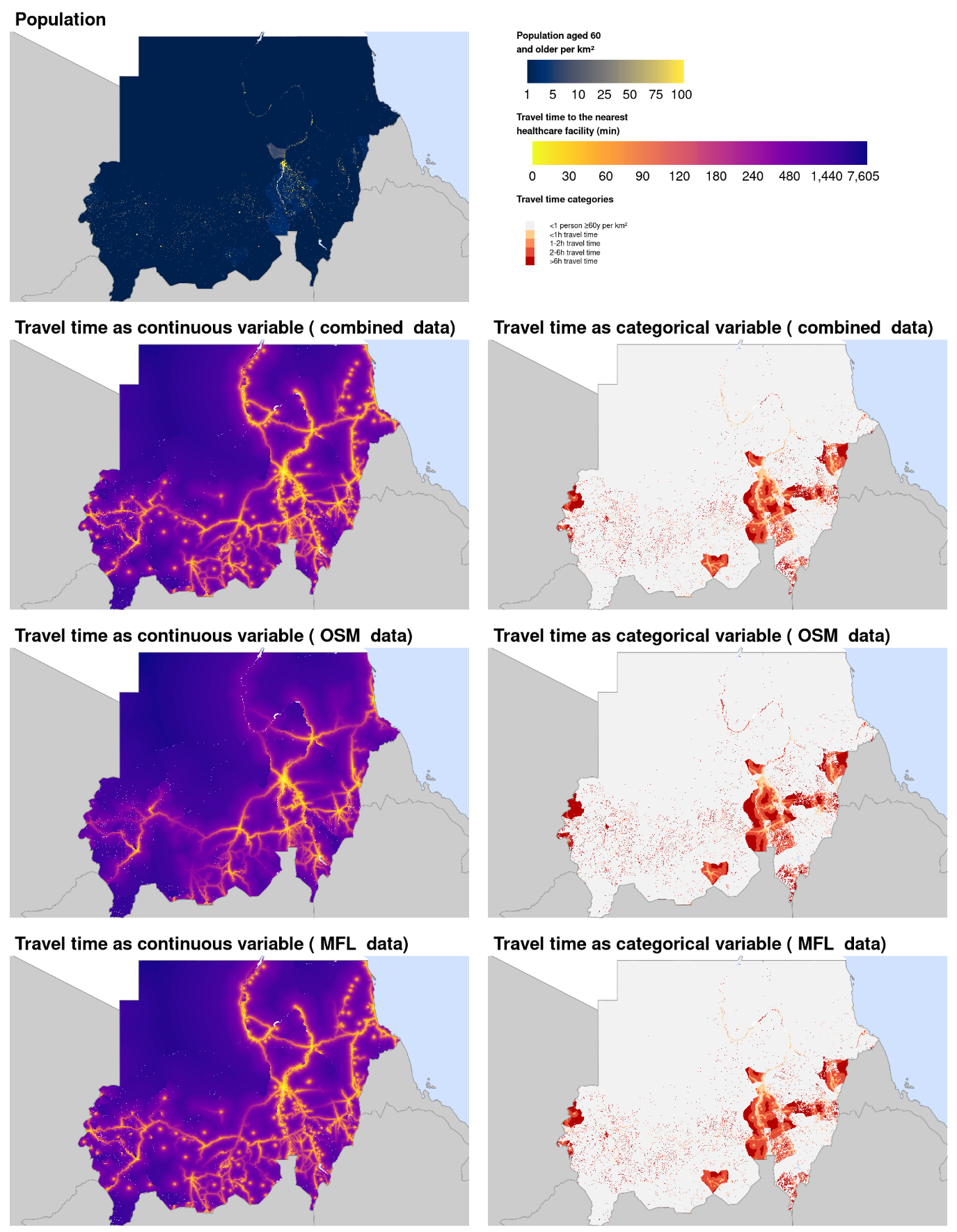


# **Figure S91. Tanzania map of travel time to the nearest healthcare facility for adults aged ≥ 60 years**

**
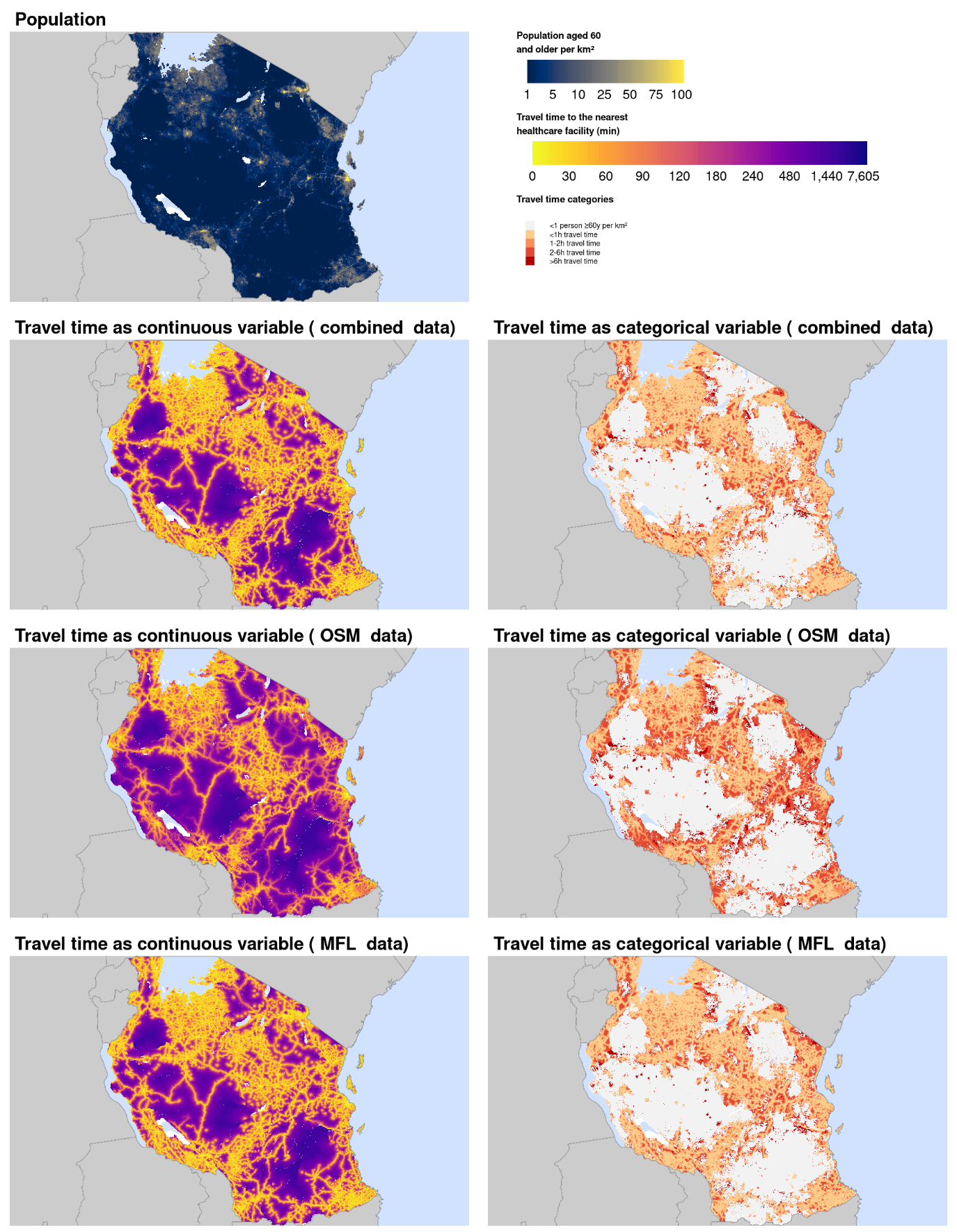
**

# **Figure S92. The Gambia map of travel time to the nearest healthcare facility for adults aged ≥ 60 years**


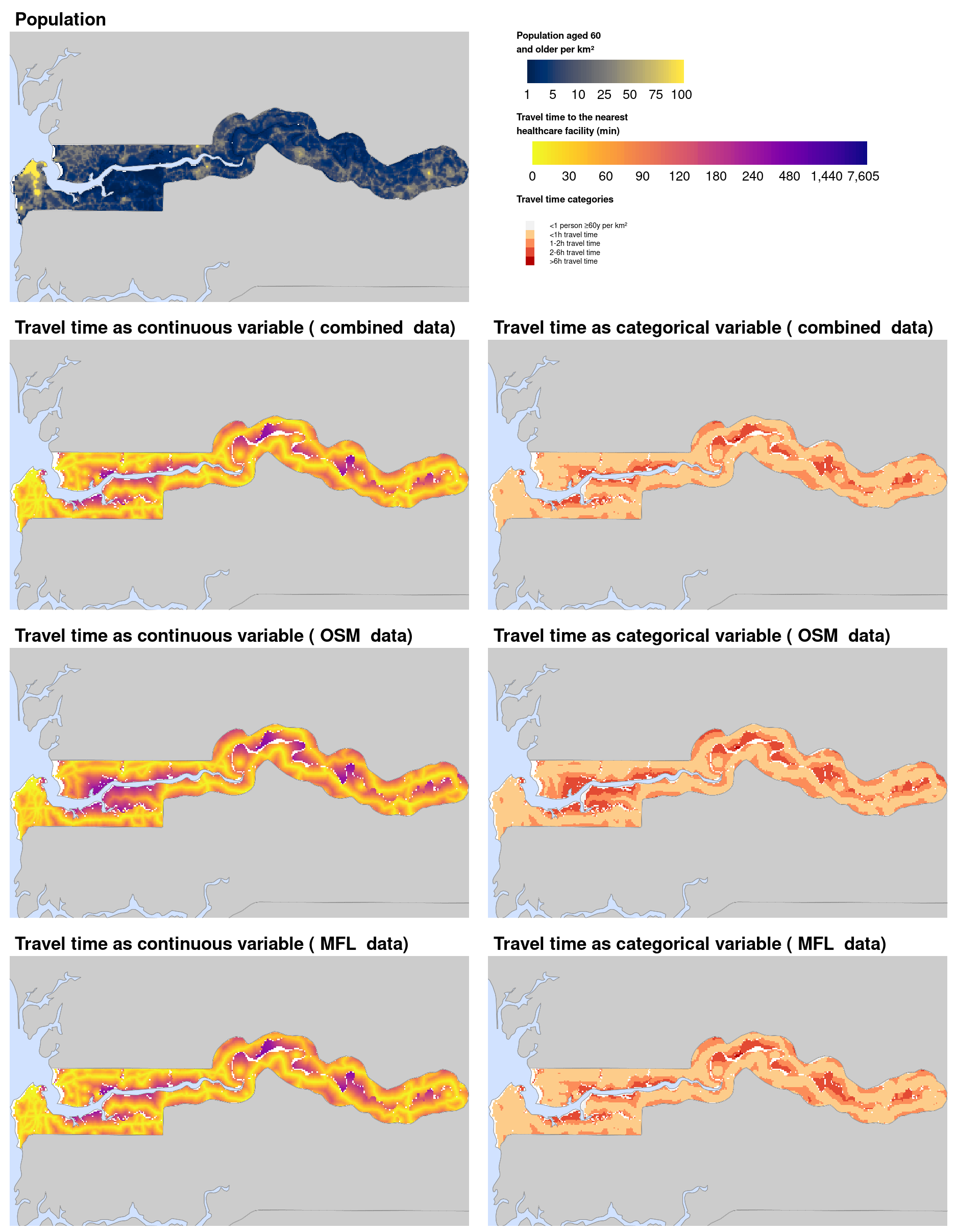


# **Figure S93. Togo map of travel time to the nearest healthcare facility for adults aged ≥ 60 years**

**
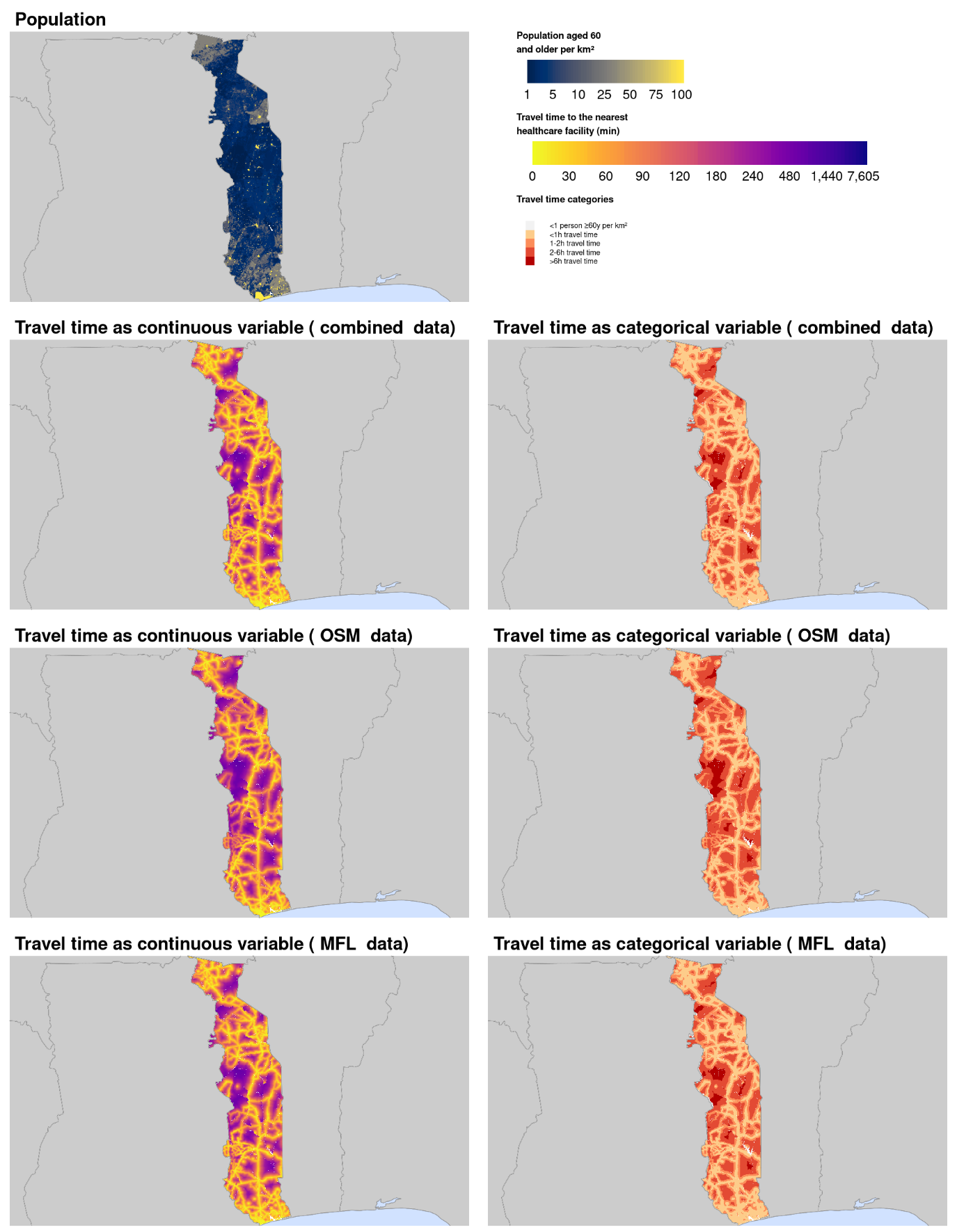
**

# **Figure S94. Uganda map of travel time to the nearest healthcare facility for adults aged ≥ 60 years**


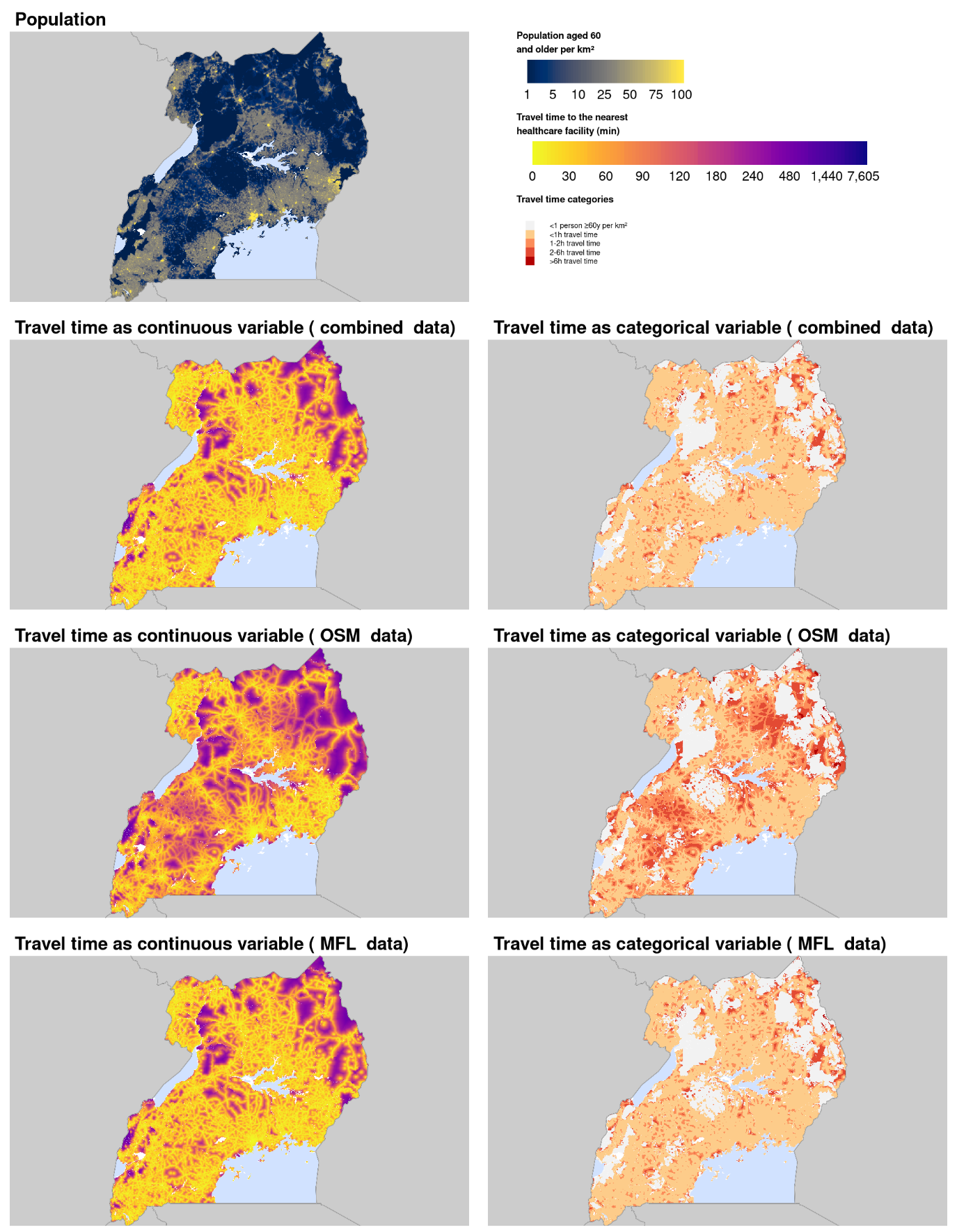


# **Figure S95. Zambia map of travel time to the nearest healthcare facility for adults aged ≥ 60 years**


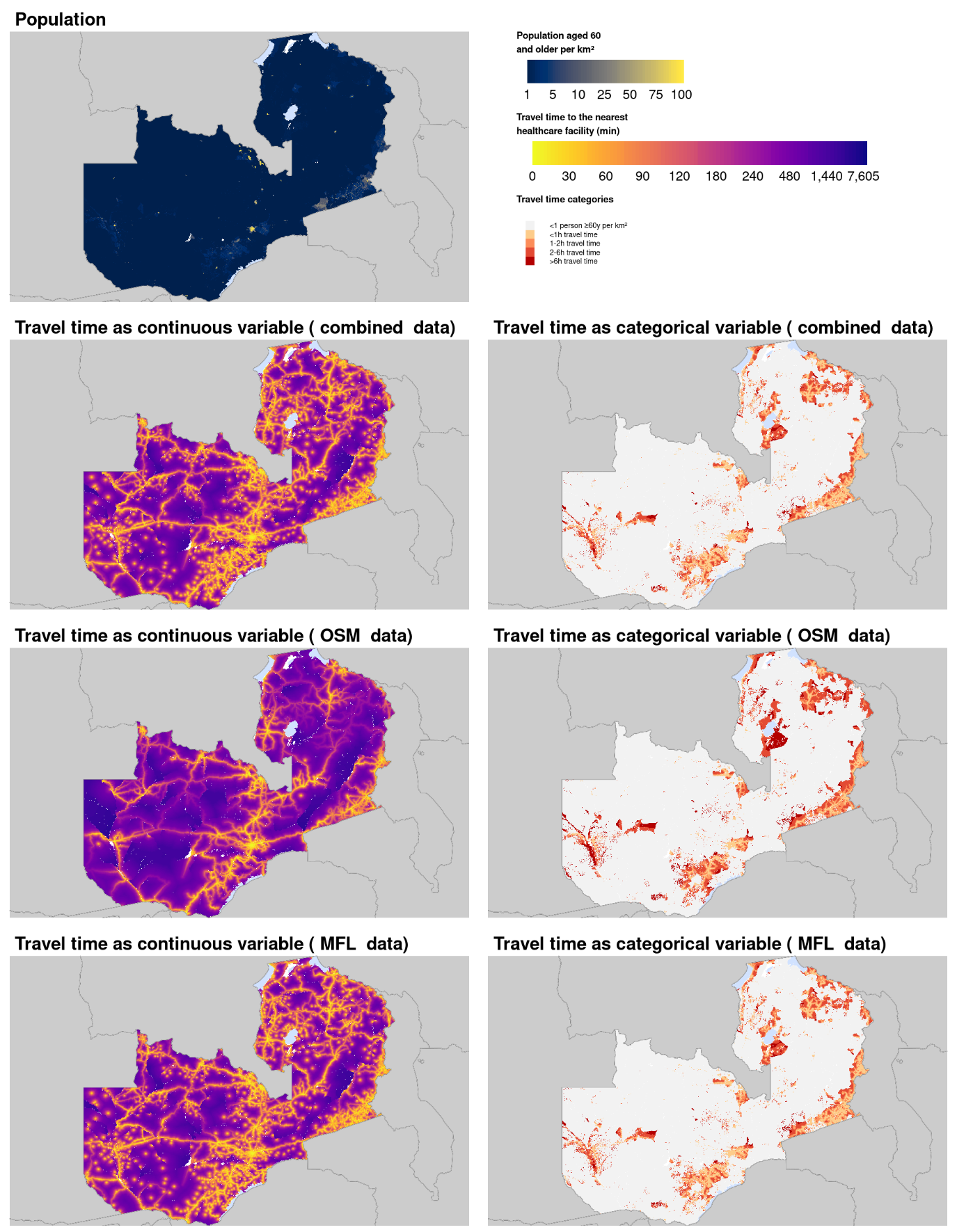


# **Figure S96. Zimbabwe map of travel time to the nearest healthcare facility for adults aged ≥ 60 years**


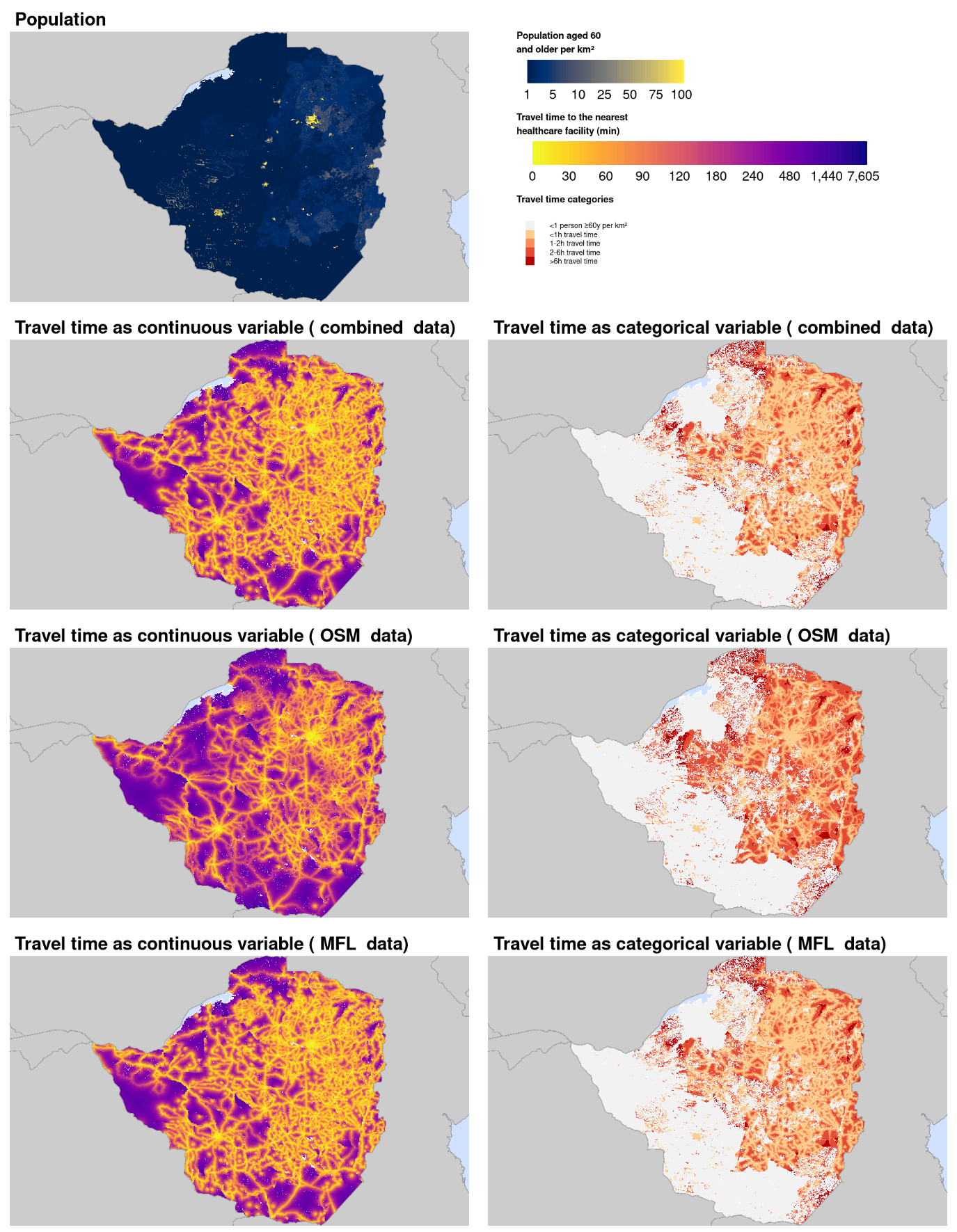


# **Figure S97. Maps of travel time to the nearest healthcare facility for adults ≥ 60 years, by region based on the MFL dataset**


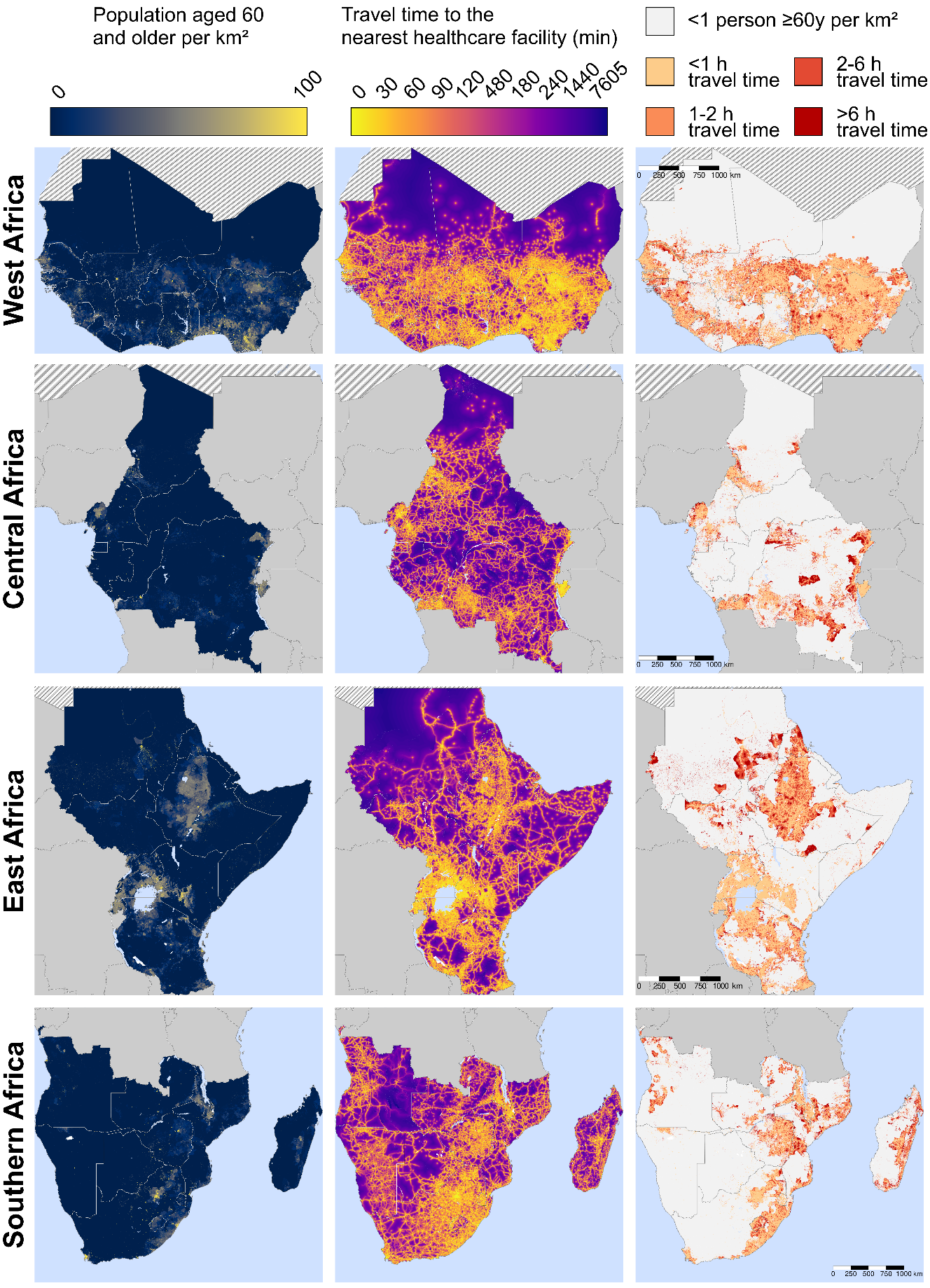


# **Figure S98. Maps of travel time to the nearest healthcare facility for adults ≥ 60 years, by region based on the OSM dataset**


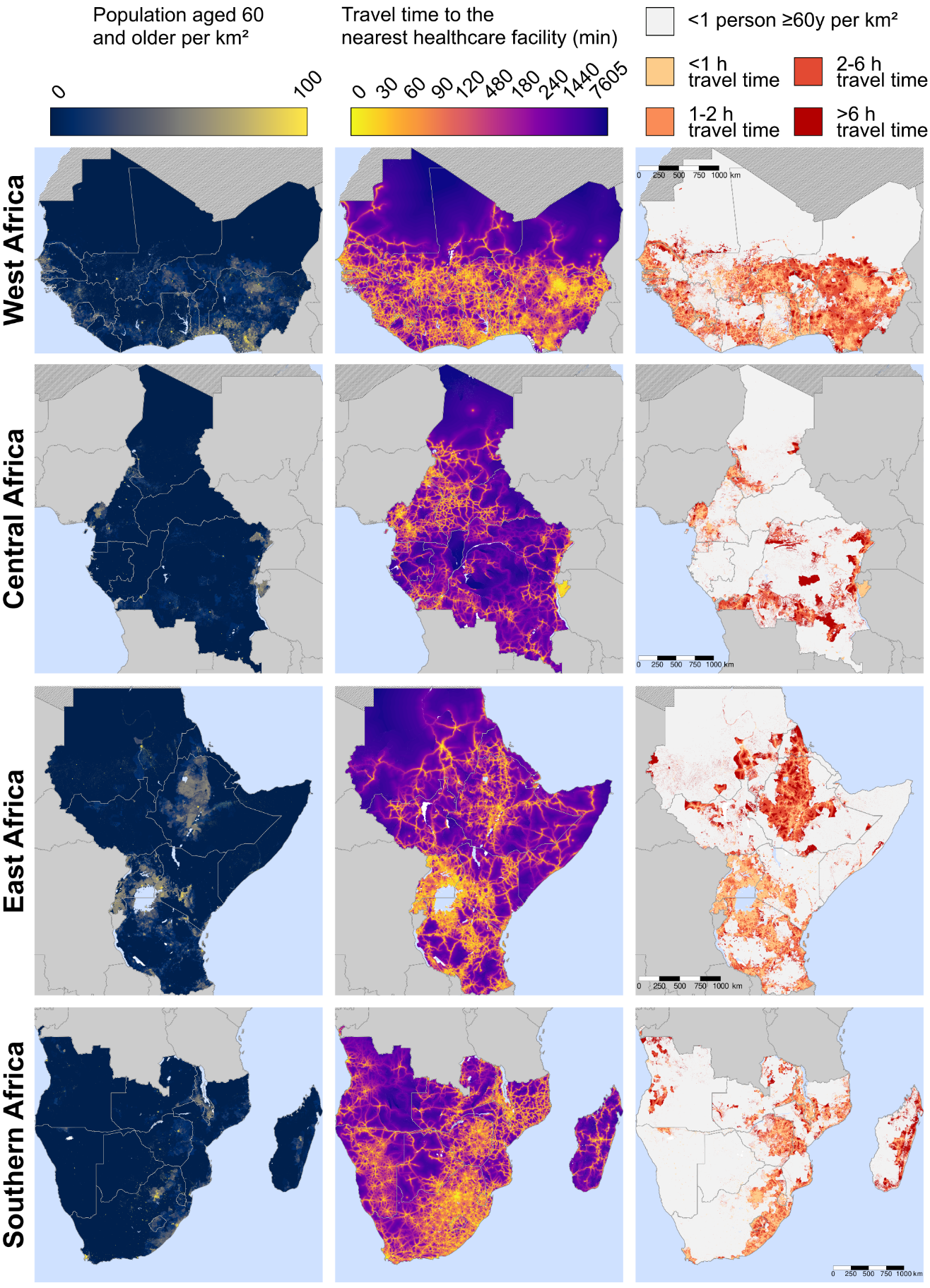


# **Figure S99. Location of healthcare facilities in OSM and MFL data for Angola**


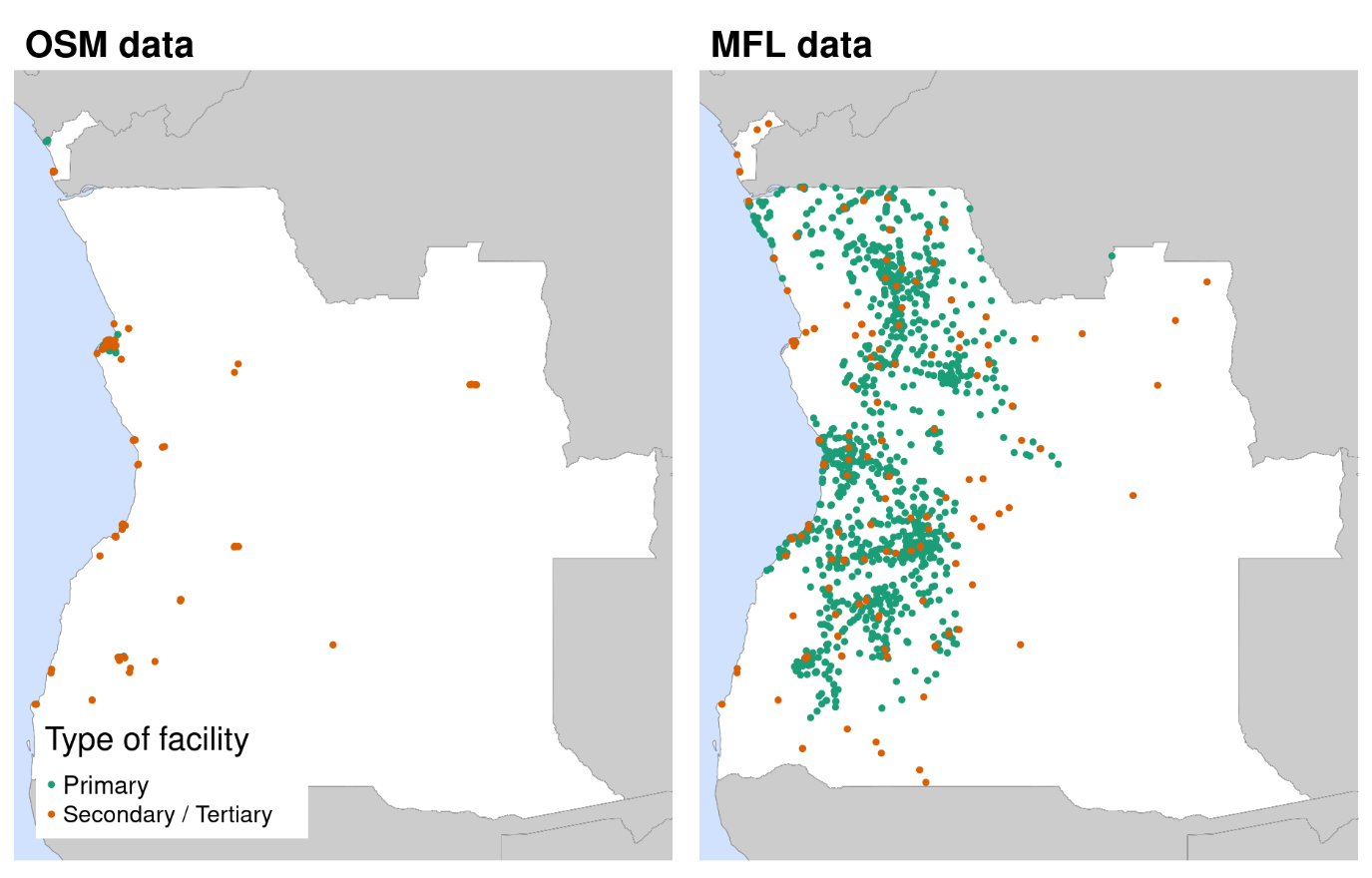


# **Figure S100. Location of healthcare facilities in OSM and MFL data for Benin**

# **Figure S101. Location of healthcare facilities in OSM and MFL data for Botswana**

# **Figure S102. Location of healthcare facilities in OSM and MFL data for Burkina Faso**

# **Figure S103. Location of healthcare facilities in OSM and MFL data for Burundi**

# **Figure S104. Location of healthcare facilities in OSM and MFL data for Cameroon**

# **Figure S105. Location of healthcare facilities in OSM and MFL data for Central African Republic**

# **Figure S106. Location of healthcare facilities in OSM and MFL data for Chad**

# **Figure S107. Location of healthcare facilities in OSM and MFL data for Djibouti**

# **Figure S108. Location of healthcare facilities in OSM and MFL data for DRC**

# **Figure S109. Location of healthcare facilities in OSM and MFL data for Equatorial Guinea**

# **Figure S110. Location of healthcare facilities in OSM and MFL data for Eritrea**

# **Figure S111. Location of healthcare facilities in OSM and MFL data for eSwatini**

# **Figure S112. Location of healthcare facilities in OSM and MFL data for Ethiopia**

# **Figure S113. Location of healthcare facilities in OSM and MFL data for Gabon**

# **Figure S114. Location of healthcare facilities in OSM and MFL data for Ghana**

# **Figure S115. Location of healthcare facilities in OSM and MFL data for Guinea**

# **Figure S116. Location of healthcare facilities in OSM and MFL data for Guinea-Bissau**

# **Figure S117. Location of healthcare facilities in OSM and MFL data for Ivory Coast**

# **Figure S118. Location of healthcare facilities in OSM and MFL data for Kenya**

# **Figure S119. Location of healthcare facilities in OSM and MFL data for Lesotho**

# **Figure S120. Location of healthcare facilities in OSM and MFL data for Liberia**

# **Figure S121. Location of healthcare facilities in OSM and MFL data for Madagascar**

# **Figure S122. Location of healthcare facilities in OSM and MFL data for Malawi**

# **Figure S123. Location of healthcare facilities in OSM and MFL data for Mali**

# **Figure S124. Location of healthcare facilities in OSM and MFL data for Mauritania**

# **Figure S125. Location of healthcare facilities in OSM and MFL data for Mozambique**

# **Figure S126. Location of healthcare facilities in OSM and MFL data for Namibia**

# **Figure S127. Location of healthcare facilities in OSM and MFL data for Niger**

# **Figure S128. Location of healthcare facilities in OSM and MFL data for Nigeria**

# **Figure S129. Location of healthcare facilities in OSM and MFL data for Republic of the Congo**

# **Figure S130. Location of healthcare facilities in OSM and MFL data for Rwanda**

# **Figure S131. Location of healthcare facilities in OSM and MFL data for Senegal**

# **Figure S132. Location of healthcare facilities in OSM and MFL data for Sierra Leone**

# **Figure S133. Location of healthcare facilities in OSM and MFL data for Somalia**

# **Figure S134. Location of healthcare facilities in OSM and MFL data for South Africa**

# **Figure S135. Location of healthcare facilities in OSM and MFL data for South Sudan**

# **Figure S136. Location of healthcare facilities in OSM and MFL data for Sudan**

# **Figure S137. Location of healthcare facilities in OSM and MFL data for Tanzania**

# **Figure S138. Location of healthcare facilities in OSM and MFL data for The Gambia**

# **Figure S139. Location of healthcare facilities in OSM and MFL data for Togo**

# **Figure S140. Location of healthcare facilities in OSM and MFL data for Uganda**

# **Figure S141. Location of healthcare facilities in OSM and MFL data for Zambia**

# **Figure S142. Location of healthcare facilities in OSM and MFL data for Zimbabwe**

# **Table S1. Overlap of the GPS location of healthcare facilities with a building footprint and settlement locations in Bing satellite imagery**

| **Dataset** | **Facility type** | **Facilities examined** | **Overlap^1^ with building footprint** | | **No overlap**  **but near**  **to a settlement^2^** | | **No overlap**  **and not near**  **to a settlement^2^** | |
| --- | --- | --- | --- | --- | --- | --- | --- | --- |
|  |  |  | *N* | *%* | *N* | *%* | *N* | *%* |
| OSM | Primary care | 80 | 66 | 82.5% | 13 | 16.3% | 1 | 1.3% |
| OSM | Hospital | 80 | 68 | 85.0% | 10 | 12.5% | 2 | 2.5% |
| MFL | Primary care | 80^3^ | 47 | 60.3% | 20 | 25.6% | 11 | 14.1% |
| MFL | Hospital | 80^3^ | 53 | 68.0% | 16 | 20.1% | 9 | 11.5% |
| *Total* | *-* | *320* | *234* | *74.1%* | *59* | *18.7%* | *23* | *7.3%* |

^1^ Overlap with a building footprint was defined as a building being visible within a five meter radius around the GPS location of the healthcare facility.

^2^ “Near to a settlement” was defined as being within 1,000m of a settlement.

^3^ We were unable to conduct this validity exercise for four healthcare facilities (two primary care facilities and two hospitals in MFL data) because the satellite image was either covered by clouds or of insufficient resolution.
